# Engineering metazoan fatty acid synthase to control chain length applied in yeast

**DOI:** 10.1038/s41589-025-02105-w

**Published:** 2026-01-07

**Authors:** Damian L. Ludig, Xiaoxin Zhai, Alexander Rittner, Christian Gusenda, Maximilian Heinz, Svenja Berlage, Ning Gao, Adrian J. Jervis, Yongjin J. Zhou, Martin Grininger

**Affiliations:** 1 Institute of Organic Chemistry and Chemical Biology, Buchmann Institute for Molecular Life Sciences, Goethe Universität Frankfurt am Main, Frankfurt, Germany; 2 Division of Biotechnology, Dalian Institute of Chemical Physics, Chinese Academy of Sciences, Dalian, PR China; 3 State Key Laboratory of Phytochemistry and Natural Medicines, Dalian, PR China; 4 University of Chinese Academy of Sciences, Beijing, PR China; 5 Unilever Research & Development, Port Sunlight, Wirral, UK

**Keywords:** Biocatalysis, Enzyme mechanisms

## Abstract

Metazoan fatty acid (FA) synthases (mFASs) facilitate the de novo synthesis of C16- and C18-FAs through iterative extensions within the FA cycle and hydrolytic release. Here we re-engineer mFAS to fine-tune the interplay between FA extension and FA hydrolytic release for the targeted production of short- and medium-chain fatty acids. Single amino acid exchanges in the ketosynthase domain can redirect FA product profiles from predominantly C8 (G113W) to C8/C10 (G113F) and C12/C14 (G113M). Integration of a thioreductase domain enables the production of medium-chain fatty aldehydes and alcohols. We apply our approach for controlling chain length in FA biosynthesis to the microbial production of C10- and C12-FAs, translate it into a yeast cell factory and achieve C10/C12-FAs titers of 674 mg l^−1^ and 67% purity of total free FAs. Our work demonstrates a modular platform for programmable FA synthesis and paves the way toward sustainable bioproduction of valuable oleochemicals.

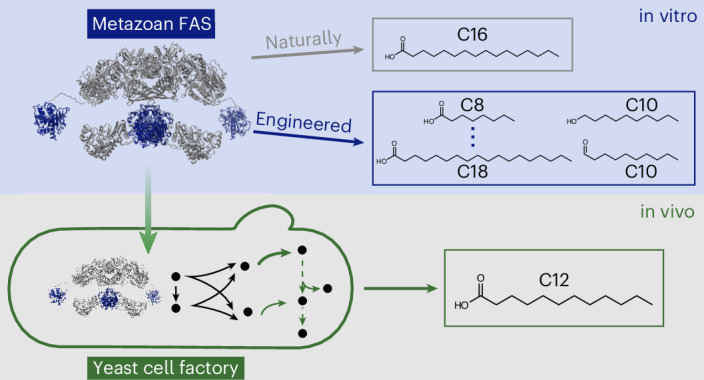

## Abstract (web summary)

The authors report on engineering metazoan fatty acid synthase variants with tunable selectivity to obtain short- and medium-chain fatty acids, alcohols and aldehydes. Pairing these optimized enzymes with a yeast strain designed for efficient β-oxidation yields high production levels of medium-chain fatty acids.

## Main

The biosynthesis of FAs with specific chain lengths is of high interest to the chemical industry^1,2^. For example, short-chain FAs (SCFAs) (≤C8) find widespread use in the food, pharmaceutical and cosmetic industries^3,4^, whereas medium-chain FAs (MCFAs) (C10–C14) can be directly used as lubricants, fragrances, paint additives and pharmaceuticals^5^. The biotechnological production of short- and medium-chain FAs (SMCFAs) offers a potential sustainable alternative to land-dependent coconut and palm oil extraction. To date, approaches for the microbial production of SMCFAs have been based on the eukaryotic multienzyme FA synthases (FASs)^6–11^ and the bacterial FAS system^12–16^ composed of separate enzymes. Many approaches harness the substrate-tolerant truncated version of the thioesterase TesA (‘TesA) from *Escherichia* *coli*, which is able to hydrolyze short and medium chains^17–20^; however, beyond titers lagging behind industrial demands, approaches to date have suffered from a lack of specificity in the production of the desired chain length^1,2^.

**Fig. 1:**
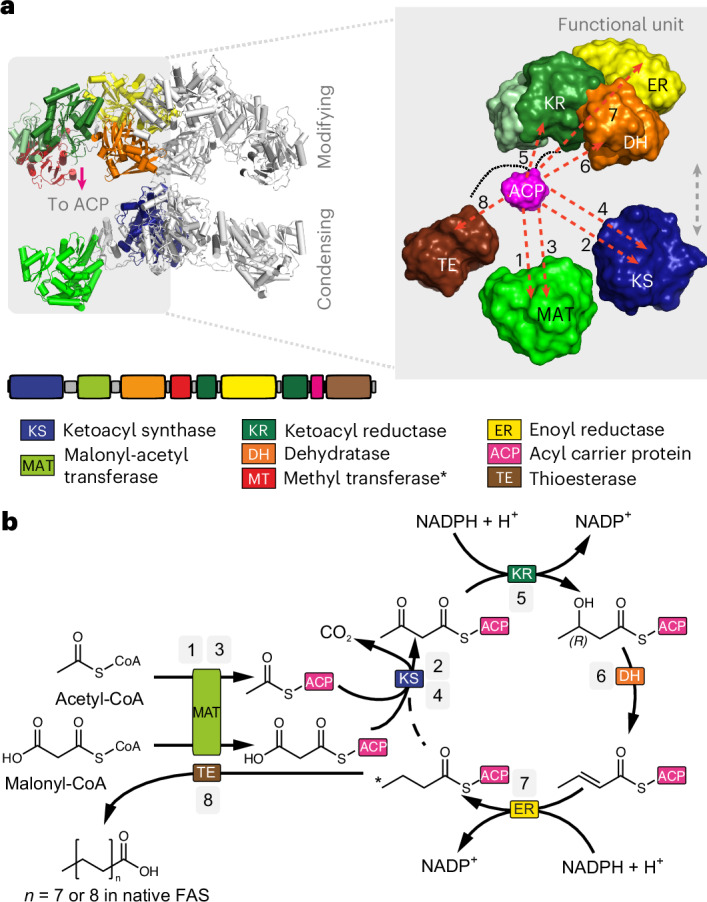
Structure of metazoan FAS, FA synthesis. **a**, Crystallographic structure of porcine mFAS (PDB ID: 2VZ8). The homodimeric mFAS adopts an extended X-shaped conformation. Condensing and modifying parts are connected by a short linker and form two lateral reaction clefts. The TE and ACP domains are attached flexibly such that they cannot be traced in electron density. Zoom in shows that one reaction cleft is attached to depict substrate shuttling by ACP. Numbers indicate the sequence of reactions (the path of ACP in shuttling substrates and intermediates to the catalytic domains). **b**, Cycle of metazoan FA biosynthesis. At defined length, TE releases the acyl chain as free FA. Numbers indicate the sequence of reactions as shown in **a**.

The metazoan FAS (mFAS) forms an open 550 kDa homodimeric X-shaped fold with two reaction clefts (Fig. 1a)^21–24^. mFAS naturally synthesizes C16- and C18-FAs through an iterative process, typically starting with acetyl-coenzyme A (Ac-CoA) as the priming substrate and using malonyl-CoA (Mal-CoA) for elongation, with each cycle adding two carbons from malonyl to the growing fatty acyl chain^25,26^. Each carbon–carbon bond formation by the ketoacyl synthase (KS) domain is followed by reduction of β-ketoacyl by the β-ketoacyl reductase (KR), dehydration to enoyl by the dehydratase (DH) and further reduction by the enoyl reductase (ER) to the saturated fatty acyl. To serve this action, the enzymatic domains KS, KR, DH and ER are substrate-tolerant to the growing chain length of FAs. In contrast, the FA-releasing thioesterase (TE) domain of mFAS is substrate specific, and very precisely intercepts synthesis after seven or eight cycles to release free C16- or C18-FAs, respectively (Fig. 1b)^27^. FA biosynthesis is assisted by substrate shuttling mediated by the compact four-helical bundle domain ACP, which is flexibly linked to the multienzyme FAS^28,29^. The ACP covalently binds substrates and intermediates through a post-translationally introduced phosphopantetheine moiety. Their covalent attachment prevents substrates and intermediates from diffusing out of the catalytic compartment, thereby maintaining high local concentrations that enhance enzymatic efficiency^30,31^. Notably, fungi possess a distinct FAS multienzyme that has evolved along a separate evolutionary trajectory, while following similar synthetic principles (Supplementary Fig. 1a, b)^23,24,32^.

We recently demonstrated that the turnover rate of the KS domain is chain length dependent, with medium-chain substrates being processed more efficiently than longer ones. As the KS domain also serves as the gatekeeper of the FA cycle, the progression of an acyl chain through the cycle is inherently dependent on its length^33,34^. The following describes the iterative elongation pathway leading to the formation of a C16-FA: the initial elongation of the acetyl moiety, which primes the FA synthesis, proceeds with relatively low efficiency. KS efficiency then increases with chain length, showing a 6.7-fold enhancement for hexanoyl moieties during the third cycle passage, and a further 2.5-fold increase for decanoyl moieties in the fifth cycle. These increases correspond to lowered transition state energies by 2 and 4 kJ mol^−1^, respectively. As the growing acyl chain approaches the target chain length, the overall rate of the FA cycle declines. At this point, the substrate-specific TE domain terminates the iterative process, releasing the C16-FA^27,35,36^.

In this work, we engineer mFAS variants that are capable of producing SMCFAs by decreasing the efficiency of the KS in processing acyl moieties of longer chain lengths and installing a TE with broad chain length specificity. In such mFAS variants, the promiscuous TE would preferentially hydrolyze short- and medium-chain length substrates into free FAs, as they are poor substrates for KS. To implement this strategy in mFAS, we generate an mFAS variant that has lost its stringent control over chain length. As a next step, we adjust the kinetic properties of KS and ‘TesA by targeted mutations to decrease efficiency of KS for longer substrates, while enhancing the efficiency of ‘TesA for short and medium chain lengths. This dual optimization enables efficient synthesis of SMCFAs, with tunable enzymatic properties to enable the selective production of specific chain length subsets. We also create an acyl reducing mFAS hybrid by replacing the mFAS TE with a terminating thioreductase domain (TR). By re-tuning KS-mediated elongation versus TR-mediated reductive release, we achieve direct enzymatic synthesis of medium-chain alcohols and aldehydes. Finally, we engineer an *Ogataea* *polymorpha* yeast cell factory with tuned β-oxidation for the selective reduction of long-chain FAs (LCFAs) to serve as a chassis for mFAS/‘TesA hybrids. Fed-batch fermentation enables the production of 674 mg l^−1^ MCFAs (190 mg l^−1^ C10-FA and 484 mg l^−1^ C12-FA), highlighting the potential of this host system for sustainable oleochemical production from low-cost feedstocks.

## Results

### Engineering a generalist variant as production platform

We performed experiments in vitro with full-length mFAS and truncated versions, purified from *E.* *coli*^37^, which allowed us to evaluate the effects of scaffold remodeling and mutations in their impact on protein quality, enzyme kinetics and product output.

**Fig. 2:**
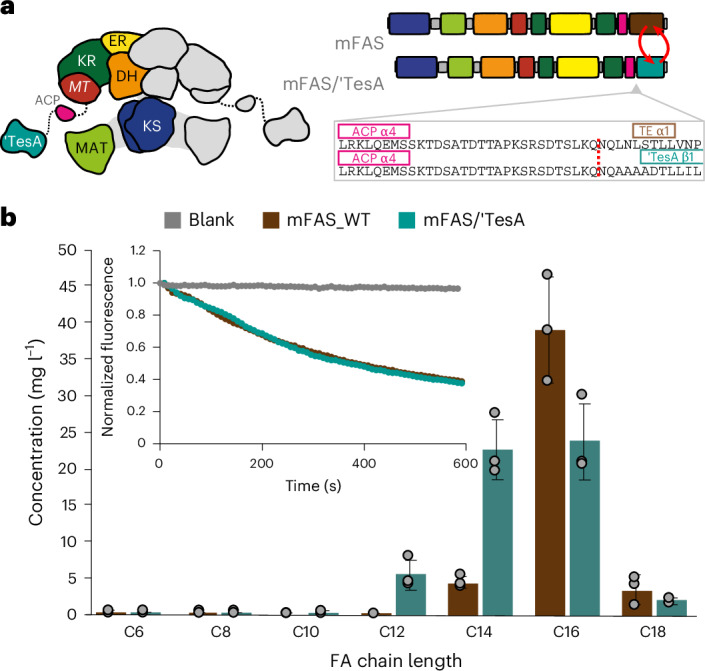
Design of a generalist mFAS. **a**, Scheme and domain borders of the TE/‘TesA swap. A cartoon of mFAS is shown for clarity with one protomer colored. **b**, Exemplary activity assay of mFAS and the mFAS/‘TesA hybrid, monitored by NADPH consumption. The specific activities of WT mFAS and mFAS/‘TesA were 460 and 458 nmol min^−1^ mg^−1^, respectively. Inset shows product distributions of mFAS and the mFAS/‘TesA hybrid. The bars represent the means of three biological replicates and the error bars show s.d. Source data

As reported earlier, the FA chain length in metazoan FA synthesis is largely determined by the TE domain, which is specific for C16 acyl chains^27^. To generate a platform for the production of FAs of various chain length, we replaced the native TE domain with ‘TesA from *E.* *coli* (Fig. 2a and Supplementary Fig. 2a–d). ‘TesA exhibits broad substrate specificity with a preference for medium-chain acyl moieties (Supplementary Fig. 2a–d)^19^ and has been widely used for producing SMCFAs^12,15,20,38^. The replacement of TE^mFAS^ with ‘TesA was informed by previous work swapping mFAS TE with mammalian TEII and *Cuphea* *palustris* TE^10^. We received an mFAS/‘TesA hybrid with rates of FA synthesis that remained essentially unchanged compared to wild-type (WT) mFAS (Fig. 2b), indicating that the replacement of the native TE with ‘TesA does not per se impose a kinetic penalty. At the same time, the TE/‘TesA swap caused a shift in the FA product spectrum toward including medium chain lengths (C12–C18). We refer to this construct as the generalist mFAS, reflecting the loss of stringent chain length control and its suitability as an engineering platform for tailoring FA output.

### Engineering substrate specificity of KS

**Fig. 3:**
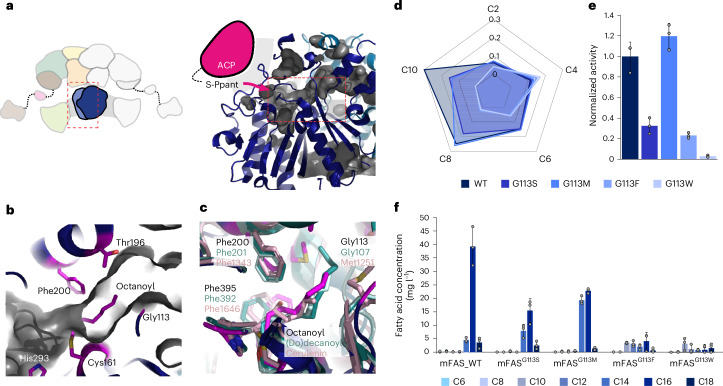
Engineering the KS domain. **a**, Cross section of the KS dimer with the binding tunnel represented by its surface in gray. The ACP binding site is indicated. An mFAS model is attached for overview. **b**, Active site and acyl binding cavity of the KS domain. Key amino acids and the Cys161-bound octanoyl moiety are highlighted. Gly113 is positioned centrally in the binding tunnel near atom C8 of the substrate. Murine FAS numbering. **c**, Superposition with *E.* *coli* FabB with bound decanoyl and dodecanoyl moieties (PDB IDs 1F91 (light cyan) and 1EK4 (dark cyan)) and *S.* *cerevisiae* FAS with bound cerulenin (PDB ID 2VKZ, light pink). **d**, Radar chart of KS elongation rates for substrates of various chain lengths (C2–C10) of five different KS mutants at position 113. The rates represent three biological replicates and are given in 1 s^−1^. WT, wild type. **e**, Normalized activity of mFAS_WT and four different KS mutants at position 113 as monitored by NADPH consumption. The mean enzymatic activity of WT mFAS was 373 nmol min^−1^ mg^−1^ (data collected in biological triplicates with three technical replicates each, error bars indicate s.d.). **f**, Product distributions of mFAS_WT carrying mutated KS (data represents the mean ± s.d. of biological triplicates with three technical replicates each). FAs were monitored as methyl esters (FAMEs) by gas chromatography (GC) after methylation. Source data

The KS performs a two-step reaction comprising the loading of the acyl chain to the active cysteine of KS (transacylation) and the elongation of the bound acyl moiety by a C2-unit (decarboxylative Claisen condensation) (Supplementary Fig. 3). The specificity of the KS-mediated elongation reaction originates from the transacylation of the acyl chain to the active cysteine (Cys161 in murine mFAS)^34^, such that acyl:KS complex structures provide valuable information for engineering. We utilized structural data of murine mFAS with a KS-bound octanoyl moiety as a basis for engineering (Fig. 3a, b)^39^. Since acyl chain binding is largely conserved across species, we also harnessed related structural information (Fig. 3c)^40,41^. Finally, the design of KS variants was further guided by homologous KSs from FAS/PKS systems that are known to produce mainly hexanoyl during olivetol biosynthesis (Supplementary Fig. 4)^42,43^.

We hypothesized that sterically demanding residues replacing Gly113, Ser117, Met132, Ala160 and Thr196 have an influence on chain length control. A second line of evidence for Gly113 as a hotspot for engineering chain length originated from our own work on yeast^6^ and corynebacterial FAS^44^ (Fig. 3c). Overall, we generated a library of 14 binding tunnel mutants of the KS (Supplementary Figs. 5–8), which we evaluated in elongation kinetics (Supplementary Fig. 9). Among these variants, Gly113 demonstrated exceptional plasticity to exchanges with sterically demanding residues Ser<Met<Phe<Trp. Low rates for hexanoyl and octanoyl substrates agree with the steric restrictions imposed by phenylalanine and tryptophan, whereas the turnover rates received for acetyl and butyryl substrates remained essentially unchanged in Gly113 mutants (Fig. 3d and Supplementary Fig. 10a, b).

KS mutations (G113S/M/F/W) were then integrated into full-length mFAS and subjected to in vitro activity and product assays. Except for the G113M variant, the activity of the KS-mutated variants decreased as the size of the amino acid at position 113 increased (Fig. 3e). Further, as amino acid size increased, the FA product spectra shifted toward shorter FAs, and yields of free FAs dropped in line with decreased activity (Fig. 3f and Supplementary Fig. 11a, b).

### Chain length modulation by combined engineering of KS and ‘TesA

**Fig. 4:**
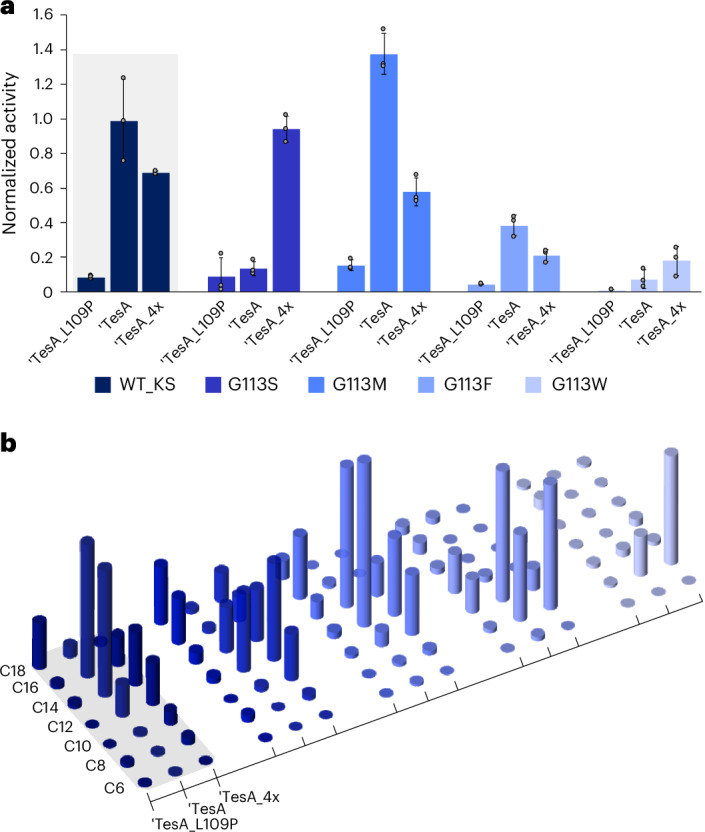
In vitro analysis of 12 mFAS/‘TesA hybrids engineered in KS and ‘TesA. **a**, Normalized activity monitored by NADPH consumption. The assay was performed in 20 µl volume. Components were used in final concentrations of 100 μM Acetyl-CoA, 100 μM Malonyl-CoA, 50 μM NADPH and 20–80 nM enzyme. Data collected in biological triplicates with three technical replicates each, and normalized to mFAS_WT activity. Error bars represent s.d. **b**, Product distributions collected in three biological replicates with three technical replicates each. Data collected for hybrid constructs with nonmutated KS highlighted by a gray background. Source data

As a next step, we introduced selected KS mutations into the mFAS/‘TesA generalist variant, and additionally modified ‘TesA according to variants with known enzymatic properties from previous studies: ‘TesA_L109P hydrolyzes C8-ACP ten times slower than WT, but retains specificity for shorter chains^18,19^, and ‘TesA_M141L/E142D/Y145G/L146K (‘TesA_4x) exhibits higher specificity for short chains and a tenfold increased rate for C8-ACP cleavage compared to WT (Supplementary Fig. 10b)^18^. In the background of WT KS, mFAS/‘TesA_4x and mFAS/‘TesA_L109P had a different impact on the FA synthesis. The mFAS/‘TesA_4x hybrid operated with similar activity to the mFAS/‘TesA (nonmutated ‘TesA) and further broadened the spectrum to release C8 to C16-FAs (Fig. 4a,b; data highlighted by gray background). In contrast, the mFAS/‘TesA_L109P exhibited substantially decreased activity in line with low hydrolysis activities of this ‘TesA variant. The shift in the product spectrum toward C18-FAs correlates with ‘TesA_L109P being ineffective in intercepting the FA cycle driven by the nonmutated KS. Thus, ‘TesA_L109P can only cleave FAs at chain lengths beyond C16, when KS rates have sufficiently dropped (Supplementary Fig. 11c,d).

We then combined the three ‘TesA variants (‘TesA, ‘TesA_L109P and ‘TesA_4x) with the four selected KS mutations (G113S/M/F/W) (Supplementary Figs. 12 and 13), yielding 12 mFAS/‘TesA hybrids, and recorded catalytic efficiency and product output spectra (Fig. 4a,b and Supplementary Fig. 14a,b). While mFAS/‘TesA hybrids with aromatic amino acids substituting Gly113 (G113F/W) showed consistently decreased activities, as a result of the throttled KS elongation rates, hybrids with KS mutations G113S and G113M varied in activity depending on the ‘TesA variant. In particular, they showed high NADPH consumption rates with ‘TesA (G113S-variant) and ‘TesA_4x (G113S & G113M). The hybrids mFAS^G113S^/‘TesA_4x and mFAS^G113M^/‘TesA even surpassed rates of WT mFAS, which can be attributed to the high elongation rates of G113S and G113M for short acyl chains that excellently align with the specificity profile of ‘TesA and ‘TesA_4x. The data indicate that the produced chain lengths are primarily dictated by the KS mutation and are further fine-tuned by the matching ‘TesA variant.

### Production of fatty aldehydes and alcohols

**Fig. 5:**
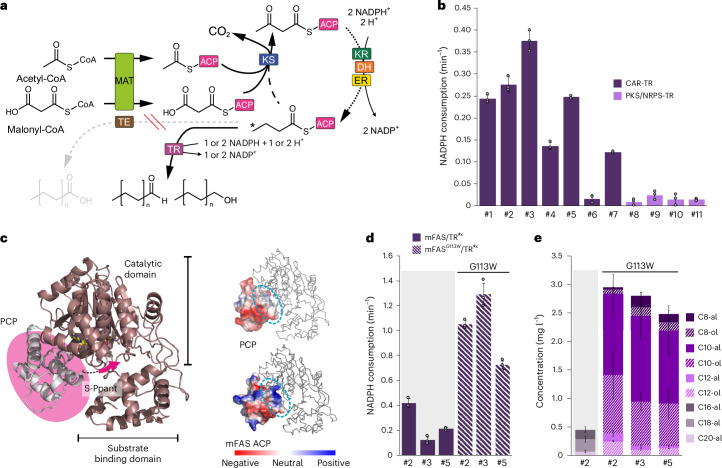
Construction of mFAS/TR hybrids for the direct production of short and medium chain length aldehydes/alcohols. **a**, Scheme of aldehyde and alcohol production with mFAS/TR. **b**, Activity screening of seven TR^CAR^ and four TR^PKS/NRPS^ for the reductive cleavage of the decanoyl group from C10-ACP as decanal and/or decanol. Reductive domains are derived from: *Mycobacterium* *marinum* (#1), *M.* *phlei* (#2), *M.* *smegmatis* (#3), *Nocardia* *iowensis* (#4), *N.* *otitidiscaviarum* (#5), *Tsukamurella* *paurometabola* (#6), *Neurospora* *crassa* (#7), *Stigmatella* *aurantiaca* (#8), *M.* *tuberculosis* (#9), *M.* *smegmatis* (#10) and *Methanobrevibacter* *ruminantium* (#11). Data represent the mean ± s.d. of technical triplicates. **c**, AlphaFold model of PCP-TR^CAR^ from *M.* *smegmatis* with docked PCP^60^. NADPH is not part of the PCP-TR^CAR^ *M.* *smegmatis* model, but it was superimposed from the PCP-TR^CAR^ structure from *Segniliparus* *rugosus* (PDB ID: 5MSV). Selected active site residues in stick representation. Vacuum electrostatics indicated for PCP of modeled *M.* *smegmatis* PCP-TR^CAR^ (right/top), and for mFAS ACP modeled to the PCP atomic coordinates with Modeller^61^ (ACP 24% sequence identical to PCP of *M.* *smegmatis*) (right/bottom). **d**, Activity of mFAS/TR hybrids with WT KS (gray background) and G113W-mutated KS, respectively. NADPH consumption (by KR, ER and TR) during fatty alcohol/aldehyde synthesis was monitored in three technical replicates, error bars represent s.d. **e**, In vitro production of fatty aldehydes/alcohols with mFAS/TR hybrids with WT KS (gray background) and G113W-mutated KS, respectively. Data from the mFAS/TR hybrids were collected in technical triplicates. Data from the hybrids with G113W mutation were collected in three biological and technical replicates. Error bars represent s.d. Source data

To extend the mFAS product spectrum beyond FAs, we replaced the TE domain with a thioester reductase (TR) domain for production of fatty alcohols and/or aldehydes (Fig. 5a). We focused on two classes of TRs: TRs from carboxylic acid reductases (TRs^CAR^) and TRs from PKS/NRPS systems (TRs^PKS/NRPS^) (Supplementary Fig. 15a–c)^45–50^. TRs^PKS/NRPS^ catalyze either 2-electron or nonprocessive (2 + 2) electron reduction to the aldehyde or alcohol, respectively^51,52^. While earlier studies characterized TRs^CAR^ as exclusively performing two-electron reductions to generate aldehydes^48,53^, more recent evidence indicates the potential for (2 + 2) electron reductions as well (Supplementary Fig. 16)^49^. To select a suitable TR to replace the TE^mFAS^ domain, we screened seven TRs^CAR^ and four TRs^PKS/NRPS^, which represent a broad phylogenetic diversity and are characterized in function and partly in structure (Supplementary Fig. 17)^45–49,54^.

Standalone TRs were tested in their ability to reduce and release saturated C10 acyl chains from mFAS ACP (Fig. 5b and Supplementary Fig. 18). The activities of TRs^PKS/NRPS^ were at least one order of magnitude lower than that of TRs^CAR^, which also naturally process saturated FAs. Further, we observed that the activity of both TR types remains at least two orders of magnitude lower than the overall mFAS activity, indicating that they will impose a kinetic bottleneck when harnessed in mFAS for fatty alcohol/aldehyde production. Based on structural models, PCP and TR^CAR^ physically interact for the reductive release (Supplementary Fig. 19a)^48^. An AlphaFold3-modeled *Mycobacterium* *smegmatis* PCP:TR complex indicates that the interface is overall little charged, whereas, in contrast, the mFAS ACP displays a more positively charged surface (Fig. 5c and Supplementary Fig. 19b). Thus, we hypothesize that suboptimal, noncomplementary domain–domain interactions at least partially account for the low efficiency of the reductive release of the acyl chain bound to mFAS.

Based on results from the analysis of standalone TR domains, we exchanged the TE^mFAS^ domain with the three best performing TRs, namely the TRs^CAR^ from *Mycobacterium* *phlei*, *M.* *smegmatis* and *Nocardia* *otitidiscaviarum*, termed in the following TR^#2^, TR^#3^ and TR^#5^, respectively (Supplementary Figs. 20–22). For all constructs, the NADPH consumption rates were low (Fig. 5d), and alcohol/aldehyde production could not be detected, except for traces of long-chain fatty aldehydes hexadecanal, octadecanal and eicosanal for the hybrid mFAS/TR^#2^ (Fig. 5e). In an attempt to match the kinetics of KS and TR, as we did for the kinetic interplay of KS and ‘TesA in SCFAs and MCFAs production, we eventually decreased the flux in the FA cycle with the help of the G113W mutation in KS. In doing so, for all three mFAS/TR hybrids, the activity increased threefold (Fig. 5d), and product amounts for hybrid mFAS^G113W^/TR^#2^ increased up to eightfold (Fig. 5e).

### In vivo production in yeast

**Fig. 6:**
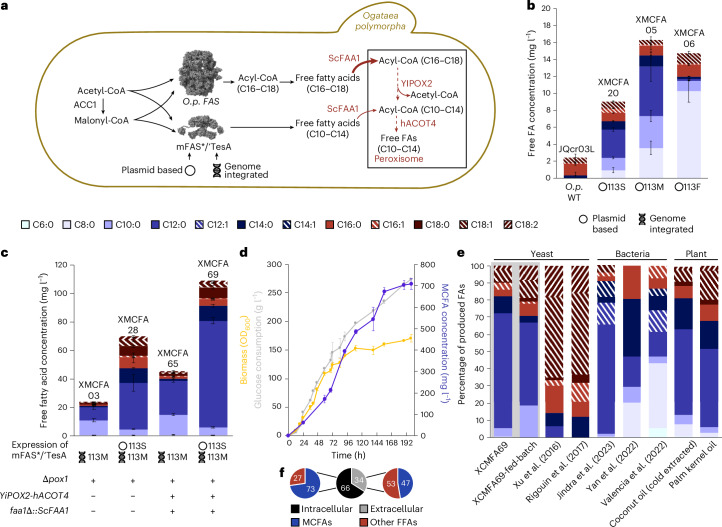
In vivo production of free FAs and fatty alcohols by using mFAS/‘TesA hybrids. **a**, Schematic depiction of the production of free FAs in *O.* *polymorpha (O.* *p.)*. The mFAS/‘TesA hybrids directly synthesize free FAs, whereas the *O.* *p*. FAS generates fatty acyl-CoAs. The produced free FAs can be coupled to CoAs by Faa1 and then transported into the peroxisome where Pox1 degrades them to acetyl-CoA during β-oxidation. The conversion of LCFAs to MCFAs can be achieved by remodeling the β-oxidation pathway. **b**, Production of free FAs by engineered *O.* *polymorpha* strains without mFAS hybrids (*O.p*. WT) or one of three mFAS/‘TesA hybrids with different KS mutations. mFAS/‘TesA hybrids were encoded from plasmid. Values shown represent the mean of three biologically independent samples, error bars depict the s.d. of the mean. **c**, Remodeling β-oxidation for enhancing the production of MCFAs. The latter two strains (XMCFA65 and 69) overexpressed *ScFAA1* and *YlPOX2-hACOT4* without and with an additional plasmid-based expression of mFAS^G113S^/‘TesA, respectively. Values shown represent the mean of three biologically independent samples, error bars depict the s.d. of the mean. **d**, Fed-batch fermentation of strain XMCFA69 in 1.5-l bioreactors. Biomass, glucose consumption and MCFA concentration over time in Delft minimal medium containing 20 g l^−1^ glucose. Values shown represent the mean of three biologically independent samples, error bars depict the s.d. of the mean. **e**, Free FA distribution in various strains and plants. XCMFA and XCMFA-fed-batch, this study; Xu et al. (*Y.* *lipolytica CnF*atB2)^9^; Rigouin et al. (*Y.* *lipolytica* JMY1233_I220W)^8^; Jindra et al. (*E.* *coli* BTE)^16^; Yan et al. (*E.* *coli* PhaG variant Q45R G142V)^62^; Valencia et al. (*P.* *putida* 3KO)^13^; coconut oil^63^ and palm kernel oil^58^. **f**, Distribution and content of free FAs at the end of fed-batch fermentation. Source data

We then introduced selected mFAS/‘TesA hybrids into yeast cell factories for the precise biosynthesis of FAs and their derivatives with controlled chain lengths. First, we overexpressed three mFAS/‘TesA constructs carrying KS mutations G113S, G113M and G113F in the thermotolerant yeast *Ogataea* *polymorpha*, which has the ability to utilize a wide range of substrates including methanol, glucose and xylose (Fig. 6a)^55^. Without additional metabolic engineering, the plasmid-based expression enabled the biosynthesis of up to 14.5 mg l^−1^ SMCFAs (Fig. 6b). For all mFAS/‘TesA hybrids, a slight shift toward shorter chains is observed compared to in vitro results. Since chain length output is substantially influenced by concentrations of acetyl-CoA and malonyl-CoA, shifted spectra may reflect cellular substrate concentrations that do not match our chosen in vitro conditions^56^.

To further assess the applicability of our chain length control strategy, and given its industrial relevance, we selected C12-FA (lauric acid) as the target chain length for yeast based in vivo production. MCFAs, particularly C12-FA, are widely used in the production of surfactants, detergents, lubricants and personal care products due to their favorable physicochemical properties and antimicrobial activity^57^. Currently, C12-FA is predominantly obtained from palm kernel oil, which only makes up below 10% of the oil in the fruit and contains about 50% C12-FA^58^. As a result, large-scale palm cultivation is required to meet demand, contributing considerably to biodiversity loss and greenhouse gas emissions in Southeast Asia^59^. To this end, the gene encoding mFAS^G113M^/‘TesA was integrated into the genome under the control of the strong constitutive promoter P_*GAP*_ (promoter of glyceraldehyde-3-phosphate dehydrogenase), which resulted in MCFAs titers comparable to those achieved with the plasmid-based expression (Supplementary Fig. 23). To further enhance free FA titers, we sought to block β-oxidation by deleting the FA oxidase gene *POX1* (strain XCMFA03), which increased MCFAs titers by 3.0-fold and improved MCFA selectivity compared to the control strain XCMFA38 (Supplementary Fig. 24). We further aimed at increasing the copy number of mFAS/‘TesA hybrids by parallel expression from plasmids and genome. For variant mFAS^G113M^/‘TesA (XCMFA27), this led to elevated titers of free C12 and C14-FAs by 3.2 and 6.1-fold, respectively. Combining the genomic expression of mFAS^G113M^/‘TesA with the plasmid-based expression of mFAS^G113S^/‘TesA (XCMFA28) increased the production of C12 and C14-FAs by 3.5 and 13.0-fold, respectively (Supplementary Fig. 24).

Additionally, rather than merely blocking β-oxidation, we considered specifically targeting the degradation of LCFAs toward MCFAs through precisely regulating β-oxidation. Feeding experiments revealed the consumption of C12- and C14-FAs by the WT strain (Supplementary Fig. 25a). Deletion of the acyl-CoA ligase encoding gene *OpFAA1* led to improved MCFA/LCFA titers (Supplementary Fig. 25b), suggesting that OpFaa1 acts on LCFAs as well as MCFAs. Replacing *OpFAA1* with *Saccharomyces* *cerevisiae ScFAA1* reduced LCFA levels while increasing MCFA levels (Supplementary Fig. 25b), demonstrating the selective activity of ScFaa1 toward LCFAs. Notably, a strategy based on differences in acyl-CoA ligase substrate specificity for modulating the FA chain length spectrum has been leveraged earlier for a *Pseudomonas* *putida* strain^13^. For the limited β-oxidation approach, we tested combinations of long-chain acyl-CoA oxidases and medium-chain acyl-CoA thioesterases to enhance the production of free MCFAs. The combinatorial expression of long-chain acyl-CoA oxidase encoding gene *YlPOX2* from *Yarrowia* *lipolytica* and human thioesterase encoding gene *hACOT4* showed the most promising results (Supplementary Fig. 26). We then proceeded to incorporate the limited β-oxidation cascade into the strain with the *OpPOX1* deletion expressing the mFAS^G113M^/‘TesA variant from the genome. This resulted in a strain XMCFA65 with a 96% increase in MCFA production and 52% C12 fraction of total free FAs, which is a 33% increase compared to parent strain XMCFA03 (Fig. 6c). Again, increasing the mFAS expression levels through additional plasmid-based expression of mFAS^G113S^/‘TesA (strain XMCFA69) further increased the MCFA titer to 90.8 mg l^−1^, corresponding to a 4.5-fold increase compared to the control strain XMCFA03 (Fig. 6c). Of note, in strain XMCFA69, the C12-FA fraction accounts for 69% of the total free FAs, which is 77% higher than that of XMCFA03. Furthermore, the strain had a 1.9-fold increase in MCFA production and a 50% increase in the C12 fraction compared to XMCFA28 (Fig. 6c). Thus, remodeling the β-oxidation pathway of LCFAs, combined with multicopy expression of mFAS/‘TesA hybrids in yeast, is an effective strategy to enhance the specific biosynthesis of MCFAs and C12-FA. Finally, to assess the in vivo potential of mFAS/‘TesA hybrids for MCFAs biosynthesis, we performed fed-batch fermentation with strain XMCFA69 in a 1.5-l bioreactor. This approach yielded a total of 708.6 mg l^−1^ MCFAs, consisting of 189.5 mg l^−1^ C10-FA, 484.1 mg l^−1^ C12-FA and 34.0 mg l^−1^ C14-FA (Fig. 6d and Supplementary Fig. 27). Of the total free FAs produced, 70% are MCFAs, and C12-FA alone accounts for 48% (Fig. 6e). Of note, a portion of 23% of MCFAs was detected in the extracellular medium (Fig. 6f), suggesting partial secretion or a form of FA-leakage. Thus, with a strain subjected to only limited metabolic engineering, we achieved the highest titers of MCFAs reported in yeast to date^8,9^, with C12-FA produced at a specificity comparable to that found in palm or coconut oil and previously engineered *E.* *coli* (Fig. 6e)^16^.

The expression of mFAS hybrids capable of producing fatty alcohols in vitro did not lead to the desired products in *O.* *polymorpha*. In contrast, expression of mFAS^G113W^/TR^#2^ and mFAS^G113W^/TR^#3^ in *S.* *cerevisiae* resulted in the successful in vivo production of C10 and C12 alcohols (Supplementary Fig. 28).

## Discussion

In metazoan de novo FA biosynthesis, the chain length of products is defined by the interplay of the mFAS domains KS and TE, with a saturated acyl chain of a given length being either elongated by the KS or hydrolyzed by the TE. Given equal concentrations of the two enzymatic domains within mFAS, it is ultimately their relative catalytic efficiency that dictates which pathway dominates. In the native biosynthesis, accurate chain length regulation at C16 is enabled by a pronounced increase in the TE’s relative catalytic efficiency at this chain length^27^, accompanied by a simultaneous efficiency loss of the KS^34,39^. In this work, we aimed at harnessing this molecular foundation of chain length control of FA biosynthesis. Specifically, we hypothesized that tuning the relative catalytic efficiencies of KS versus TE could serve as a design principle to access distinct FA chain length ranges through just modest mutational impact. As a first step, we constructed a generalist mFAS variant by replacing the highly substrate-specific native TE with the broadly active *E.* *coli* thioesterase ‘TesA. Precise kinetic data for ‘TesA hydrolysis of acyl-ACP substrates are lacking; however, we expected that its pronounced substrate promiscuity reduces chain-length selectivity in the mFAS/‘TesA hybrid, yielding a wider and less selective product distribution (for a conceptual representation of the putative kinetic behavior, see Supplementary Fig. 29a–c). Indeed, as documented by the FA output spectra (Fig. 2b), the generalist mFAS only imposed loose chain length control. Using the generalist mFAS as a platform, we subsequently modulated the kinetics of KS and ‘TesA, creating specialized variants for SMCFAs production. Mutations such as G113W, G113F or G113M substantially shifted the product spectrum toward C8 to C14 (Fig. 4b). Our mFAS engineering strategy represents an innovative engineering approach to chain length control. Rather than being dictated by a single dominant factor, chain length regulation emerges from a finely balanced interplay between two catalytic activities, representing a system-level phenomenon rather than the isolated selectivity of a single domain. The broader applicability of this principle is demonstrated by its successful implementation in the production of short and medium-chain fatty aldehydes and alcohols using engineered mFAS/TR hybrids. In these systems, product formation was only observed when the catalytic efficiency of the KS was reduced in the medium chain length range, allowing the terminating TR to effectively compete with the KS for reductive release (Fig. 5e).

For SMCFA production in yeast, three mFAS/‘TesA hybrids were initially selected for integration in *O.* *polymorpha* (Fig. 6a). The product spectra of the in vitro measurements are well reflected in the in vivo production, characterizing the mFAS/‘TesA hybrids as robust producers of SMCFAs. The robustness of the mFAS-based chain length regulation in the complex environment of a yeast cytoplasm is remarkable, and can largely be attributed to compartmentalization, which enables synthesis to proceed in a highly autonomous manner (Fig. 6b). We then focused on producing C12-FA in the industrially relevant strain *O.* *polymorpha* with the goal of enhancing both, product specificity and titer. To this end, we implemented a strategy involving targeted modulation of β-oxidation as a central concept in our engineering strategy. Specifically, we deleted the *POX1* gene, which encodes the rate-limiting acyl-CoA oxidase in peroxisomal β-oxidation, and redirected the degradation of LCFAs toward MCFAs through targeted regulation of β-oxidation. Furthermore, to refine metabolic re-routing, we replaced the endogenous *OpFAA1*, which acts on both LCFAs and MCFAs, with *ScFAA1* from *S.* *cerevisiae*. ScFaa1 preferentially activates LCFA and allows MCFA accumulation. Eventually, the strain (XMCFA69) produced 90.8 mg l^−1^ of MCFAs in high purity of C12 over other chain lengths (Fig. 6c). While β-oxidation is typically viewed as a catabolic process and often suppressed in metabolic engineering strategies for SMCFA production, we demonstrate its utility as a tunable mechanism for refining product chain length profiles in yeast.

MCFAs, particularly C12-FA, are currently primarily derived from palm kernel oil. Microbial production platforms must meet several key criteria to serve as a viable alternative to existing plant-based oleochemical technologies: selective synthesis of short- and medium-chain oleochemicals, industrially relevant titers with low land-use requirements, and the ability to utilize cost-effective, sustainable feedstocks^59^. In this work, we focused on product specificity with the goal of establishing a robust, broadly applicable and tunable platform for the de novo biosynthesis of SMCFAs. The mFAS/‘TesA hybrid system, presented here, fulfills this role. Without additional metabolic engineering, plasmid-based expression of the mFAS/‘TesA construct enabled selective production of C12-FA (37%, strain XMCFA20) and C8-FA (70%, strain XMCFA06) (Fig. 6b). To explore the potential of our approach, we further optimized C12-FA biosynthesis through metabolic engineering. Under fed-batch fermentation conditions, a minimally engineered *O.* *polymorpha* strain yielded 0.674 g l^−1^ C10/C12-FAs (29% C10-FA, 71% C12-FA), which account for 67% of total FAs (Fig. 6d). Given the industrial relevance of *O.* *polymorpha*^55^, these findings underscore the strong potential of mFAS/‘TesA hybrids for developing efficient cell factories for sustainable SMCFA production.

## Methods

### Heterologous expression of mFAS constructs

Plasmids containing full-length mFAS constructs and mFAS hybrids were transformed into electrically competent *E.* *coli* BAP1 cells and plasmids containing KS-MAT^0^ were transformed into chemically competent *E.* *coli* BL21 gold (DE3). After transformation cells were plated on LB-Agar plates (100 µg ml^−1^ ampicillin (amp) and 1% (w/v) glucose). Overall, 3–6 colonies were picked and cells were grown overnight at 37 °C in 20 ml LB medium (100 µg ml^−1^ amp and 1% (w/v) glucose). Pre-cultures were used to inoculate 1 l TB medium (100 µg ml^−1^ amp). Cultures were grown at 37 °C and 150 rpm until they reached the desired optical density (OD_600_) of 0.6–0.8. After cooling at 4 °C, cultures were induced with 250 µM IPTG and grown for an additional 16 h at 20 °C and 150 rpm. Cells were collected by centrifugation (5,000*g* for 15 min). After the supernatant was discarded, the remaining cell pellet was resuspended in 30 ml lysis buffer (200 mM KCl, 30 mM imidazole, 50 mM potassium phosphate, 10% (v/v) glycerol, 1 mM EDTA, pH 7.0), a small amount DNase I (Sigma Aldrich) was added. A French Pressure Cell Press (Amico) was used to disrupt the cells. The resulting suspension was centrifuged using the JA 25.500 rotor at 40,000*g* for 1 h. The supernatant was mixed with 1 M MgCl_2_ to a final concentration of 2 mM and then transferred to Ni-NTA columns and washed with five column volumes (CVs) of His-wash buffer (lysis buffer without EDTA). Bound protein was eluted with 5 ml elution buffer (200 mM KCl, 300 mM imidazole, 50 mM potassium phosphate and 10% (v/v) glycerol, pH 7.0). The eluted protein was then transferred to a Strep-Tactin column. After being washed with five CVs of strep-wash buffer (250 mM potassium phosphate, 1 mM EDTA and 10% (v/v) glycerol, pH 7.0), bound protein was eluted with 5 ml strep-elution buffer (strep-wash buffer + 40 mM biotin). Representative SDS–PAGE images can be found in Supplementary Figs. 12 and 15. Afterwards proteins were concentrated to 5–10 mg ml^−1^, frozen in liquid nitrogen and stored at −80 °C. Next, proteins were thawed at 37 °C for 60 min and further purified by size-exclusion chromatography (SEC). A Superdex 200 GL 10/300 column and strep-wash buffer were used. Representative SEC profiles can be found in Supplementary Figs. 13 and 16. Fractions with dimeric protein were pooled and subsequently concentrated to 2–5 mg ml^−1^. Aliquots were frozen in liquid nitrogen and stored at −80 °C.

### Heterologous expression of mFAS ACP and TR domains

Plasmids containing mFAS ACP and TR domains were transformed into chemically competent *E.* *coli* BL21 gold (DE3). They were expressed as the mFAS constructs but no Strep-Tactin purification was performed. Representative SDS–PAGE images can be found in Supplementary Fig. 14. SEC was conducted with a Superdex 200 GL 10/300 column and His-wash buffer was used. Protein fractions containing the correct size were pooled and concentrated to 1–10 mg ml^−1^ for the TR domains and 40–60 mg ml^−1^ for the mFAS ACP. Aliquots were frozen in liquid nitrogen and stored at −80 °C.

### MabA assay

The KS kinetics were determined as described previously^34^. In short, condensation activity of the KS domain was monitored indirectly with an enzyme coupled assay using a reductase MabA from *Mycobacterium* *tuberculosis*. All solutions were prepared in an assay buffer (50 mM sodium phosphate and 10% glycerol, pH 7.0) as 8× stock or 2× stock (Mal-ACP) and pipetted immediately before monitoring. The components were added to a final concentration of 500 nM enzyme, 20 µM Mal-ACP, 100 µM Acyl-ACP, 50 µM NADPH and 5 µM MabA. The NADPH fluorescence (excited 348–320 nm, detected 476–420 nm) was monitored during the reaction at 25 °C.

### mFAS activity assays

Measurements of the mFAS-based constructs were carried out using a CLARIOstar Plus Microplate Reader and 386-well-plates (Greiner). NADPH fluorescence was measured (excited 348–320 nm, detected 470–420 nm) at 25 °C. The assay was performed in 20 µl assay volume. All solutions were prepared in an assay buffer (50 mM potassium phosphate, 10% glycerol (v/v), 5% PEG 400 (v/v), 0.03 mg ml^−1^ BSA and 1 mM dithiothreitol (DTT), pH 7,0) as 4× stock and pipetted immediately before monitoring. The components were added to a final concentration of 100 μM Acetyl-CoA, 100 μM Malonyl-CoA, 50 μM NADPH and 20–80 nM enzyme (WT and chain length constructs) or 400–800 nM enzyme (TR hybrids). Before the measurements each protein was thawed for 5 min at 37 °C and stored on ice until the measurement. Blank measurements were conducted without acetyl- and malonyl-CoA.

### TR activity screening

Measurements of the standalone TR domains were carried out using a CLARIOstar Plus Microplate Reader and 386-well plates (Greiner). NADPH fluorescence was measured (excited 348–320 nm, detected 470–420 nm) at 25 °C. The assay was performed in 21 µl assay volume. All solutions were prepared in an assay buffer (50 mM potassium phosphate, 10 mM MgCl_2_ and 10% glycerol (v/v), pH 7.0) as 3× stock and pipetted immediately before monitoring. The components were added to a final concentration of 10 μM NADPH, 460 µM C10-mFAS ACP and 1 µM TR. Before the measurements each protein was thawed for 5 min at 37 °C and stored on ice until the measurement. Blank measurements were conducted with apo-mFAS ACP instead of C10-mFAS ACP.

### In vitro FA production assay

The in vitro production of FAs was performed similar to the mFAS activity assays. The assay was performed in 500 µl assay volume. All solutions were prepared in an assay buffer (50 mM potassium phosphate, 10% glycerol (v/v), 5% PEG 400 (v/v), 0.03 mg ml^−1^ BSA and 1 mM DTT, pH 7.0) as 4× stock and added to final concentrations of 500 μM Acetyl-CoA, 500 μM Malonyl-CoA, 500 μM NADPH and 40 nM enzyme concentration. The assay solution was then incubated overnight at room temperature. After incubation 5 µl of heptanoic acid (2 g l^−1^ in methanol:CHCl_3_, 3:1) was added as internal standard for the GC measurements.

### FA extraction and derivatization

To increase the vapor pressure and decrease the boiling point of the FAs produced in vitro, the FAs were converted to FA methyl esters (FAMEs). The procedure was adapted from rom Bligh and Dyer^64^. Therefore, the 500 µl test solution was acidified with 50 µl of 8% HCl in methanol to protonate the FAs and make them less polar. Next, 125 µl methanol:CHCl_3_ (1:1) was added and the solution was vortexed to extract the FAs to the nonpolar phase. The mixture was then centrifuged at 3,000*g* for 10 min to separate the phases. The lower nonpolar phase was transferred to a fresh Eppendorf tube and the solvent was evaporated under reduced pressure. Then, 20 µl toluene was added and transferred to Duran glass tubes containing 200 µl solution of 8% HCl in methanol and 500 µl methanol. The tubes were sealed tightly and shaken to mix the solutions. The reaction mixture was then heated to 100 °C for 3 h. The reaction was then cooled on ice for 10 min and 100 µl of hexane and 300 µl of H_2_O were added. The mixture was then vortexed and returned to ice to allow the phases to separate. 70 µl of the hexane phase was transferred to a GC vial with micro inlet.

### In vitro fatty aldehyde/alcohol production assay

The in vitro production of FAs was performed similarly to the mFAS activity assays. The assay was performed in 500 µl assay volume. All solutions were prepared in an assay buffer (50 mM potassium phosphate, 10% glycerol (v/v), 5% PEG 400 (v/v), 0.03 mg ml^−1^ BSA and 1 mM DTT, pH 7.0) as 4× stock and added to final concentrations of 300 μM acetyl-CoA, 300 μM malonyl-CoA, 500 μM NADPH and 1 µM enzyme concentration. The assay solution overlaid with 100 µl hexane and then incubated overnight at room temperature. After incubation 10 µl of heptanoic acid (0.4 g l^−1^ in hexane) was added as internal standard for the GC measurements.

### Fatty aldehyde/alcohol extraction

Extraction was carried out by adding another 50 µl hexane and mixing. The mixture was centrifuged for 10 min at 5,000*g* for phase separation. Finally, 50 µl of the organic phase was transferred to a GC vial with micro inlet.

### Gas chromatography measurements

The measurements of the FAMEs have been conducted with two different devices: a PerkinElmer Clarus 400 gas chromatograph equipped with an Elite 5MS capillary column (30 m × 0.25 mm, film thickness, 0.25 μm) and a flame ionization detector (Software, TotalChom 6.3); And an Agilent 7890B gas chromatograph equipped with a HP-5ms Ultra Inert capillary column (30 m × 0.25 mm, film thickness, 0.25 μm) and an Agilent 5977B MSD (Software MassHunter 12). The injector temperature was 200 °C and the detector temperature was 250 °C. The temperature program used was heating to 50 °C for 5 min; increase of 10 °C min^−1^ until 120 °C was reached, then hold for 5 min; increase of 15 °C min^−1^ until 220 °C was reached and then hold for 10 min.

The measurements of FA aldehydes/alcohols have been conducted with an Agilent 7890B GC equipped with an HP-5ms Ultra Inert capillary column (30 m × 0.25 mm, film thickness, 0.25 μm) and an Agilent 5977B MSD. The temperature program used was heating to 50 °C for 7 min; increase of 15 °C min^−1^ until 220 °C was reached, then hold for 5 min; increase of 20 °C min^−1^ until 280 °C was reached and then hold for 5 min.

### GC data analysis

The peaks at the previously obtained retention times of each FAME were integrated using the program ‘totalchrom’ for the PerkinElmer data, ‘OpenChrom’ was used for the Agilent data. To take losses during extraction and derivatization into account the signals was normalized to the signal of the internal standard. The final concentrations of the FAMEs were calculated using equation (1).

1 $${{\rm{c}}}_{\mathrm{sample}}=\frac{{{\rm{A}}}_{\mathrm{sample}}}{{{\rm{m}}}_{\mathrm{sample}}}\times \left(\left(\frac{{{\rm{m}}}_{\mathrm{standard}}}{{{\rm{A}}}_{\mathrm{standard}}}\times {{\rm{C}}}_{\mathrm{standard}}\right)\times 1,000\right)$$

With A_sample_ being the integrated peak area of the respective sample peak, A_standard_ being the integrated peak area of the standard peak, m_sample_ being the slope of the respective sample calibration curve, m_standard_ being the slope of the standards calibration curve and c_sample_ and c_standard_ the corresponding concentrations.

### Strains and cell cultivation

The plasmids, donor DNAs, and strains used to construct the yeast cell factories are detailed in Supplementary Tables 2–4, respectively. All yeast strains for FA production were derived from *O.* *polymorpha NCYC 495*, whereas those for fatty alcohol production were derived from *S.* *cerevisiae CEN.PK 113-11* *C*.

The formulations of LB, SD, YPD and Delft minimal medium with 20 g l^−1^ glucose are the same as those described by Zhai et al.^65^, whereas the culture conditions for FA-producing strains are consistent with Zhai et al.^65^ and the culture conditions for fatty alcohol-producing strains align with Gao et al.^66^. For the fermentation in a shake bottle, the yeast cells were cultivated in Delft minimal medium at 220 rpm for 96 h, at 37 °C for *O.* *polymorpha* and 30 °C for *S.* *cerevisiae*, with supplementation of 60 mg l^−1^ uracil or 20 mg l^−1^ methionine, respectively, where necessary.

### Genetic manipulation by CRISPR/Cas9 system

The oligonucleotides used in construction of donor DNAs shown in Table S3. All guide RNA plasmids refer to the previous descriptions^66,67,68^. Related reagents and the methods for construction of gRNA expression plasmids and donor DNAs are consistent with Zhai et al.^65^. DNA transformation into *O.* *polymorpha* and *S.* *cerevisiae* was conducted according to previous methods^66,65^.

### C12/C14-FA feeding experiment

Following cultivation of the WT *O.* *polymorpha* strain JQcr03L in 20 ml of Delft minimal medium with 20 g l^−1^ glucose to an OD_600_ of 5, C12-FA and C14-FA were added to concentrations of 800 mg l^−1^ and 600 mg l^−1^, respectively. The initial concentrations of these FAs were quantized upon addition and after a 72-h fermentation period by gas chromatography–mass spectrometry (GC–MS).

### Qualitative and quantitative analysis of FAs

The total FA extraction procedure was adapted from established methodologies with minor modifications^69^. In brief, 200 μl of appropriately diluted cell cultures was combined with 10 μl of 40% tetrabutylammonium hydroxide (Sigma, cat. no. 86854). Subsequently, 200 μl of 200 mM iodomethane (Sigma, cat. no. I8507) in dichloromethane (Sigma, cat. no. 650463) was added immediately. The dichloromethane solution contained 50 mg l^−1^ each of pentacanic acid, tridecanoic acid and nonanoic acid (Sigma, cat. no. P6125, T0502 and 75190) as internal standards. The mixture was vortexed at 1,200 rpm for 30 min and then centrifuged at 1,000*g* for 10 min to achieve phase separation. About 150 μl of the dichloromethane layer was transferred to a GC vial equipped with a glass insert and evaporated to dryness. The resulting methyl esters were reconstituted in pure hexane and analyzed using a GC–MS system (Thermo Fisher Scientific, ISQ 7610) equipped with a TG-5MS capillary column (30 m × 0.25 mm × 0.25 μm; Thermo Fisher Scientific, cat. no. 26098-1420). The GC–MS temperature program was set as initial temperature of 40 °C, held for 2 min; increased to 180 °C at 30 °C min^−1^, then raised to 200 °C at 4 °C min^−1^ and held for 1 min, followed by an increase to 240 °C at 2 °C min^−1^ and a final hold for 10 min. The injection volume was 1 μl, and the helium carrier gas flow rate was maintained at 1.0 ml min^−1^. Data were processed using Xcalibur software (v.4.1).

### Qualitative analysis of fatty alcohol

Fatty alcohol was directly extracted from the fermentation broth using n-hexane at a solvent-to-broth ratio of 1:4 (v/v). The mixture was shaken at 1,500 rpm for 30 min, followed by centrifugation at 1,000*g* for 10 min. Subsequently, 200 μl of the n-hexane layer was transferred into a GC vial equipped with a glass insert. Fatty alcohol quantification was conducted on a GC–MS system (Thermo Fisher Scientific, ISQ 7610) fitted with a TG-5MS capillary column (30 m × 0.25 mm × 0.25 μm; Thermo Fisher Scientific, cat. no. 26098-1420). The temperature program for fatty alcohol analysis was set as initial temperature held at 45 °C for 2.5 min, increased to 220 °C at 20 °C min^−1^ and held for 2 min, then raised to 300 °C at 20 °C min^−1^ and maintained for 5 min. Data were processed using Xcalibur v.4.1 software.

### Fed-batch fermentation

Fed-batch fermentations were conducted in CloudReady Bioreactors System (T&J Bio-engineering) with a 1.5-l bioreactor and 0.5-l working volume. The initial batch phase began by inoculating the pre-cultured strain XMCFA69 (OD_600_ = 4–5) into a Delft minimal medium with 20 g l^−1^ glucose, achieving a starting OD_600_ of 0.5. The process parameters were maintained at 37 °C, pH 5.6 and 30% dissolved oxygen. Agitation started at 400 rpm and aeration at 0.5 VVM, both automatically adjusted (max up to 1,000 rpm and 2 VVM, respectively) to sustain the dissolved oxygen level. Upon glucose depletion, a feeding solution of 5× Delft minimal medium with 500 g l^−1^ glucose was introduced at 1–3 ml h^−1^, adjusted according to residual glucose and ethanol concentrations.

### Reporting summary

Further information on research design is available in the Nature Portfolio Reporting Summary linked to this article.

## Online content

Any methods, additional references, Nature Portfolio reporting summaries, source data, extended data, supplementary information, acknowledgements, peer review information; details of author contributions and competing interests; and statements of data and code availability are available at 10.1038/s41589-025-02105-w.

## Supplementary information

Supplementary Information Supplementary Figs. 1–33, Methods and Tables 1–7.

Reporting Summary

Supplementary Table 1 Lists of oligonucleotides used in this study.

## Source data

Source Data Fig. 2 Statistical source data.

Source Data Fig. 3 Statistical source data.

Source Data Fig. 4 Statistical source data.

Source Data Fig. 5 Statistical source data.

Source Data Fig. 6 Statistical source data.

## Data Availability

The data generated in this study are provided within the article and in the Supplementary Information. Further data are available from the corresponding authors upon request. Source data are provided with this paper.
