# Supplementary material for: Engineering metazoan fatty acid synthase to control chain length applied in yeast
Source: Nat Chem Biol. 2026 Jan 7;22(7):1078–86. doi: 10.1038/s41589-025-02105-w (PMC13303079; doi:10.1038/s41589-025-02105-w)
Supplement: Supplementary file 1 — Supplementary Figs. 1–33, Methods and Tables 1–7. [file 41589_2025_2105_MOESM1_ESM.pdf]

nature chemical biology

Supplementary information https://doi.org/10.1038/s41589-025-02105-w

Engineering metazoan fatty acid synthase to control chain length applied in yeast

In the format provided by the authors and unedited

Table of Contents

Supplementary Figures

Supplementary Figure 1: Structure of the fungal FAS and fungal FA cycle.
Supplementary Figure 2: TE-mediated hydrolysis and structural comparison of mFAS TE domain vs. E. coli TesA.
Supplementary Figure 3: Schematic depiction of the KS Ping-Pong mechanism.
Supplementary Figure 4: Comparison of selected KS sequences.
Supplementary Figure 5: The KS binding tunnel.
Supplementary Figure 6: Representative SDS-PAGE analysis of the KS^{G113X}-MAT^{S581A} didomain constructs.
Supplementary Figure 7: Representative size-exclusion chromatograms of KS^{G113X}-MAT^{S581A} mutants.
Supplementary Figure 8: Thermal shift assay of KS^{G113X}-MAT^{S581A} mutants, as well as other KS mutants.
Supplementary Figure 9: Screening of KS mutants.
Supplementary Figure 10: KS elongation rates.
Supplementary Figure 11: *In vitro* assays of mFAS KS-mutants and mFAS/'TesA hybrids.
Supplementary Figure 12: Representative analysis of protein purity by SDS-PAGE for selected mFAS/'TesA constructs.
Supplementary Figure 13: Analysis of protein purity by SEC for the 20 mFAS/'TesA constructs used in this study.
Supplementary Figure 14: Normalized activity and product spectra of mFAS-hybrids.
Supplementary Figure 15: Thioreductase domains used in mFAS/TR engineering.
Supplementary Figure 16: Mechanism of TR-mediated reduction and product release.
Supplementary Figure 17: Phylogenetic tree of TRs^{CAR} and $TRs^{PKS/NRPS}$.
Supplementary Figure 18: SDS-PAGE of TR-domains.
Supplementary Figure 19: Interaction of carrier protein with TR^{CAR}.
Supplementary Figure 20: Sequence alignment of linkers in mFAS_WT and mFAS/TR hybrids.
Supplementary Figure 21: SDS-PAGE for mFAS/TR hybrids.
Supplementary Figure 22: Analysis of protein purity by SEC for the mFAS/TR hybrids.
Supplementary Figure 23: Production of free FAs by using $mFAS^{G113M}$/'TesA hybrids with different overexpression forms.
Supplementary Figure 24: Blocking β-oxidation for production of free fatty acids by *O. polymorpha* strains with mFASG113M/'TesA integrated in the genome.
Supplementary Figure 25: Screening long-chain fatty acyl-CoA synthases.
Supplementary Figure 26: Optimizing the combination of various fatty acyl-CoA oxidases and thioesterases.
Supplementary Figure 27: Fed-batch fermentation of strain XMCFA69 in 1.5 L bioreactors.
Supplementary Figure 28: Production of fatty alcohols by S. cerevisiae strains.
Supplementary Figure 29: Conceptual representation of the putative kinetic behavior of the interplay between KS and TE.
Supplementary Figure 30: Uncropped SDS-Page gels from supplementary figure 6.
Supplementary Figure 31: Uncropped SDS-Page gels from supplementary figure 6.
Supplementary Figure 32: Uncropped SDS-Page gels from supplementary figure 18.
Supplementary Figure 33: Uncropped SDS-Page gels from supplementary figure 23.

Supplementary Methods
Synthesis of decanoyl-CoA ester
Synthesis of C10-ACP

Supplementary Tables
Supplementary Table 1: Table of TR domains screened for this work. Given is the internal number, the enzyme class, the molecular weight, the type of reduction and their native organism.
Supplementary Table2: Cloning strategies and primers for plasmids created in this study. Supplementary Table2: Cloning strategies and primers for plasmids created in this study.
Supplementary Table 3: Cloning strategies and primers for expression cassettes created in this study.
Supplementary Table 4: Table of strains and their genotypes used in this study.
Supplementary Table 5: Amino acid and DNA sequences of the constructs used in the FA chain length experiments.
Supplementary Table 6: Amino acid and DNA sequences of the reductase domains screened for their capability to use C10-mACP as substrates.
Supplementary Table 7: Amino acid and DNA sequences of the constructs used in the fatty aldehydes/alcohol experiments.

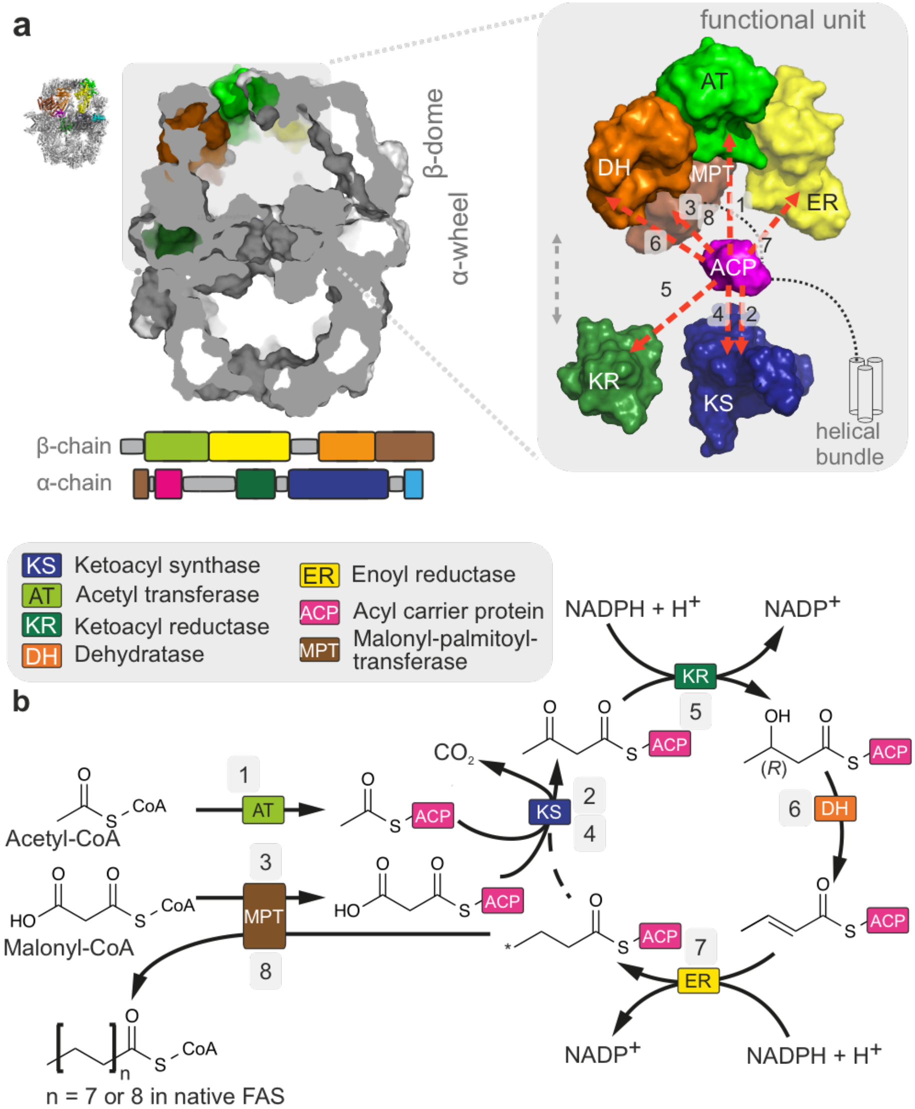

Supplementary Figure 1: Structure of the fungal FAS and fungal FA cycle. (**a**) Structure of S. cerevisiae FAS (PDB-code: 3hmj). The barrel-shaped structure (see inset, domain of one α- and one β-chain in color code of legend) is sliced along the three-fold axis to depict the two inner reaction chambers that each harbor three sets of domains. A functional unit, defined on the basis of domains in shortest distances between both ACP anchor points, is highlighted by domains in surface representation. Numbers indicate the sequence of reactions; i.e., the path of ACP in shuttling the substrates and intermediates to the catalytic domains. (**b**) Cycle of FA biosynthesis. An acetyl unit is used for priming, and the subsequently growing chain is elongated by a two-carbon (C2) unit, originating from the malonyl unit, and then processed in a cascade of three reactions (reduction–dehydration–reduction). At defined length, the MPT domain releases the acyl chain as CoA-ester. Numbers indicate the sequence of reaction as shown in (a).

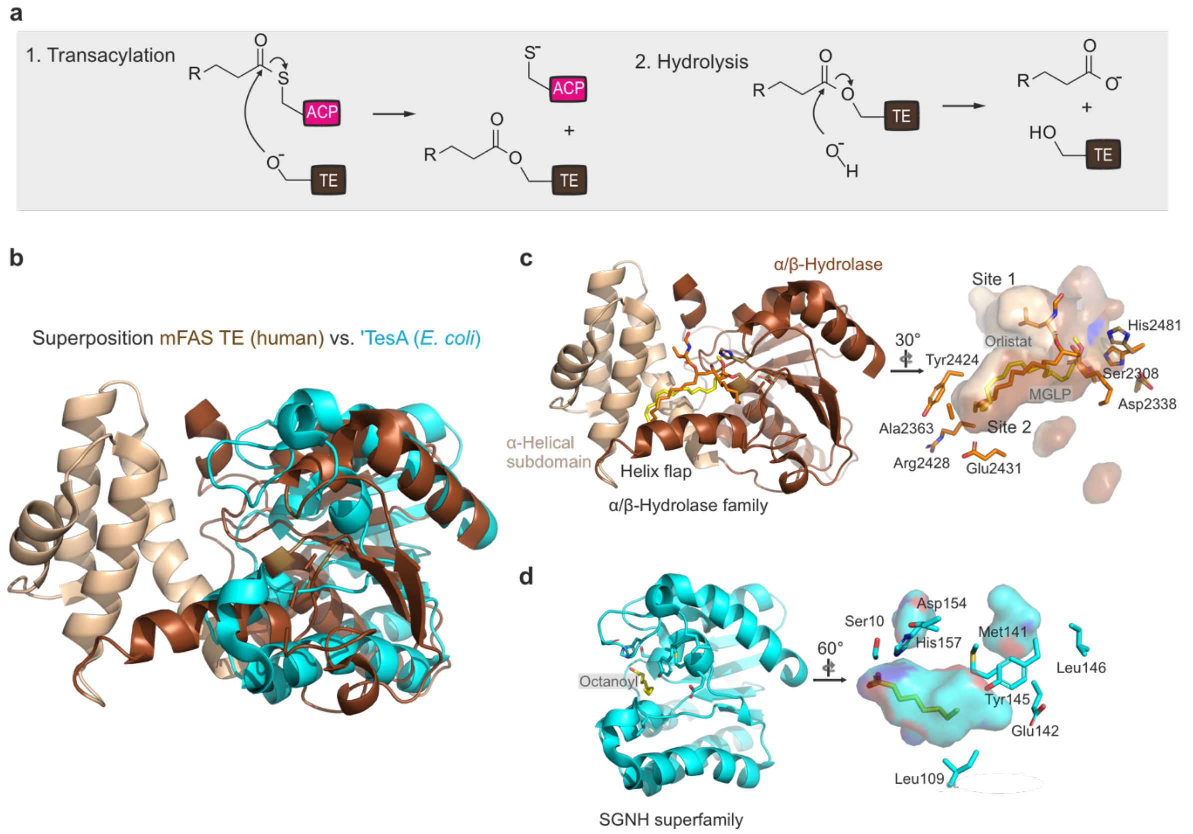

Supplementary Figure 2: TE-mediated hydrolysis and structural comparison of mFAS TE domain vs. E. coli TesA. (a) The ACP-bound acyl moiety is transacylated onto the active serine of the TE domain. The acyl chain is then released by hydrolysis. (b) Superposition of mFAS TE (human) (PDB ID: 3tjm) colored in brown and E. coli TesA (PDB ID: 1u8u) in cyan, both in cartoon representation. (c) Structure of the mFAS TE (human) with a covalently bound methyl γ-linolenylphosphonate (MGLP, PDB ID: 3tjm) in yellow and bound orlistat (PDB ID:2px6) in orange. The TE domain consists of an α/β-hydrolase fold and an inserted helical subdomain, depicted in light brown for clarity. Active site and binding site residues are highlighted in stick representation. The binding pocket surface shows the two binding sites, both at the interface to the subdomains. Binding site 2 is occupied by acyl chains of ligands orlistat and MGLP, of which MGLP causes the structuring of an amphiphilic α-helix, termed “helix flap”. Amino acid residues, suggested to be involved in chain length specificity, are highlighted in stick representation (for orlistat bound structure). (d) TesA adopts a typical α/β-hydrolase fold in which a hydrophobic groove is formed at the protein surface. The absence of a lid or capping subdomain contributes to its broader substrate specificity. The binding pocket surface is shown with an octanoyl ligand (in green), and residues, mutated in this study, are in stick representation (PDB ID: 1u8u).

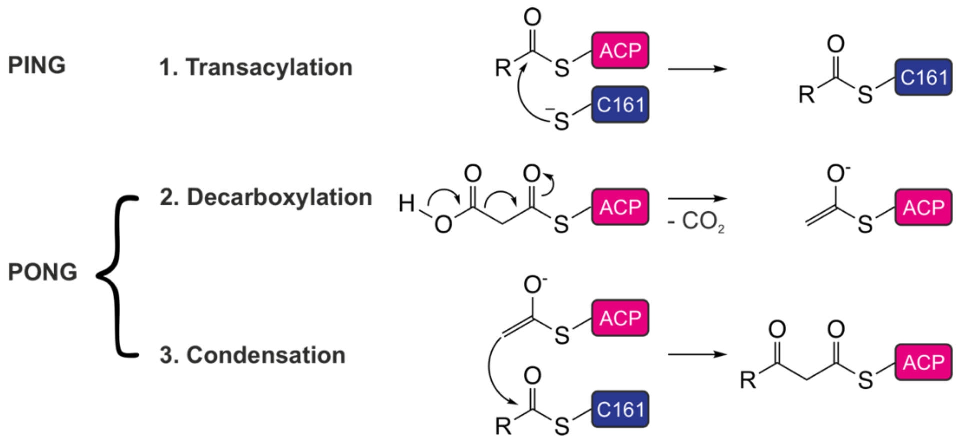

Supplementary Figure 3: Schematic depiction of the KS Ping-Pong mechanism. In the Ping step, the ACP-bound acyl is transacylated onto the active cysteine of the KS domain. The Pong step begins with the decarboxylation of a malonyl unit, which was previously loaded onto the free ACP (after Ping step) via the MAT. The resulting enolate attacks the cysteine-bound acyl chain and releasing the β-ketoacyl as ACP-bound moiety from the KS.

a

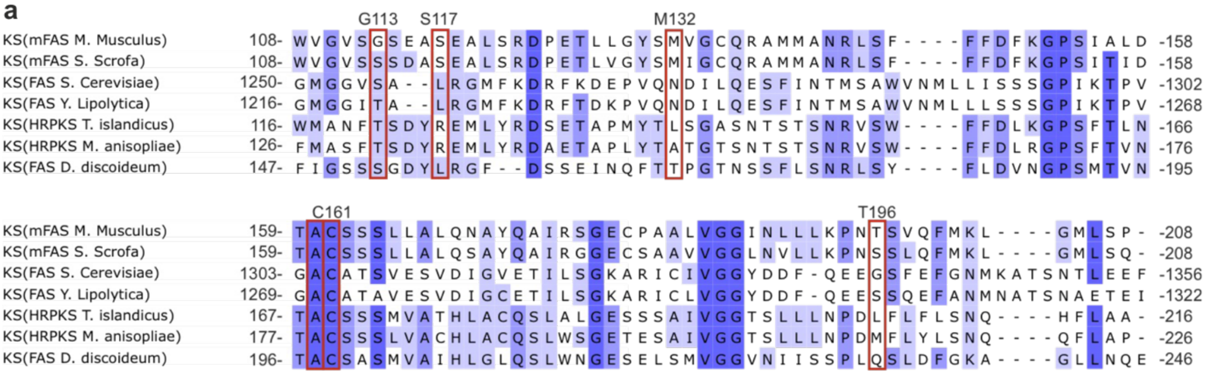

b

	KS(mFAS M. Musculus)	KS(mFAS S. Scrofa)	KS(FAS S. Cerevisiae)	KS(FAS Y. Lipolytica)	KS(HRPKS T. islandicus)	KS(HRPKS M. anisopliae)	KS(FAS D. discoideum)
KS(mFAS M. Musculus)	100%	87%	20%	12%	36%	38%	35%
KS(mFAS S. Scrofa)	89%	100%	21%	13%	38%	38%	35%
KS(FAS S. Cerevisiae)	13%	13%	100%	36%	13%	12%	14%
KS(FAS Y. Lipolytica)	17%	18%	78%	100%	18%	16%	16%
KS(HRPKS T. islandicus)	34%	35%	19%	12%	100%	70%	35%
KS(HRPKS M. anisopliae)	36%	35%	18%	11%	70%	100%	36%
KS(FAS D. discoideum)	31%	31%	19%	11%	33%	34%	100%

Legend: 10% 25% 50% 70% 90%

Supplementary Figure 4: Comparison of selected KS sequences. (a) Sequence alignment. Mutated positions are marked in red and annotated according to murine mFAS numbering. *Mus musculus* (P19096), *Sus scrofa* (A5YV76), *Saccharomyces cerevisiae* (P19097), Yarrowia lipolytica (A0A371C916), *Talaromyces islandicus* (A0A0U1MAK2), *Metarhizium anisopliae* (A0A0D9NM54), *Dictyostelium discoideum* (Q54FI3). (b) Similarity of KS sequences used in the alignment above. (Created with UGENE[2])

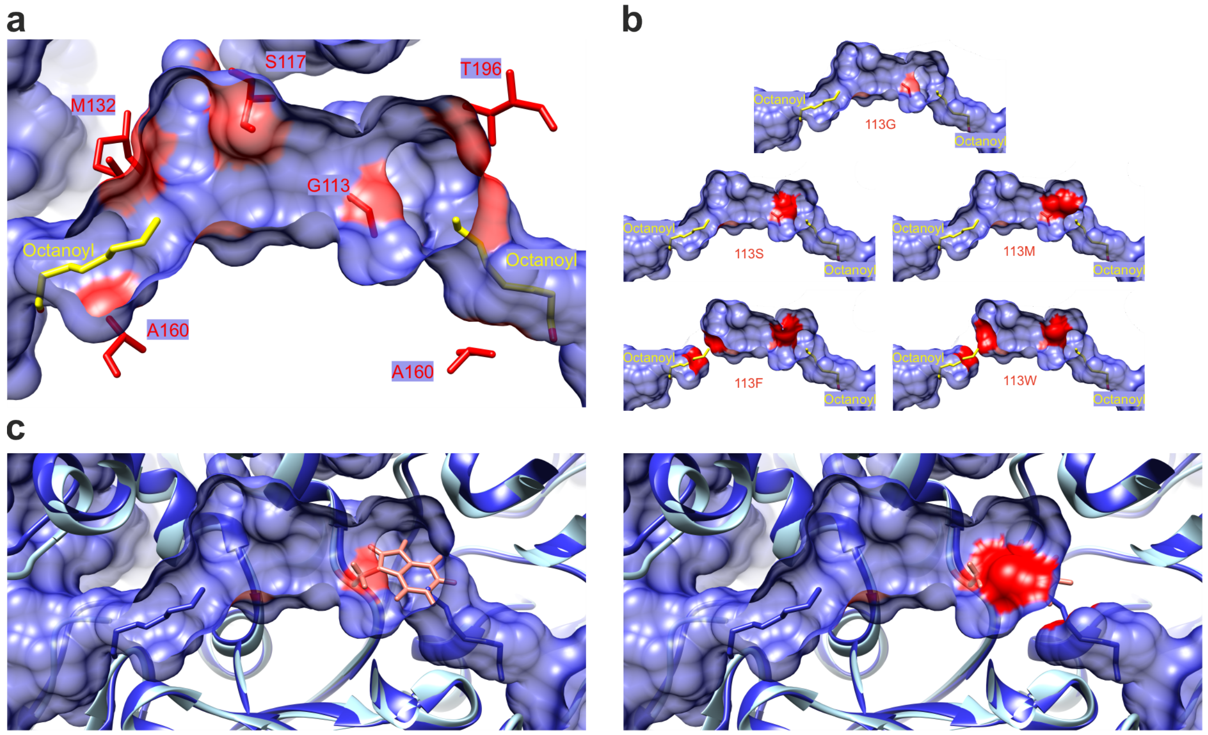

Supplementary Figure 5: The KS binding tunnel. (**a**) Depiction of the KS binding tunnel surface from octanoyl bound structure (PDB IDs: 6rop). Octanoyl chains are colored yellow, residues mutated in the KS mutant screen are colored red. (**b**) Surface depiction of the binding pocket of the mouse KS with an octanoyl chain bound to the active site serine (PDB IDs: 6rop). Position 113 highlighted in red. Residue 113 was exchanged using the rotamers tool from chimera and by selecting the most probable positioning. (**c**) Computational analysis of the sterically most demanding mutation (G113W) in the KS binding pocket. Overlay of the octanoyl-bound crystal structure with tryptophane in position of G113 for clarity in dark blue (PDB: 6ROP) with an energy optimized G113W mutant KS (light blue). Zoom onto tryptophane replacing G113. The two binding tunnels (dark blue KS_WT with C8 and light blue energy optimized G113W mutant without substrate) are depicted as surfaces calculated with chimera. This shows the steric hindrance a growing acyl chain encountered in the G113W mutant. Energy optimization of the G113W mutant was done with Cyrus Bench (ref). The RMSD value between the backbones of the crystal structure and the energy optimized structure is 0.701 Å.

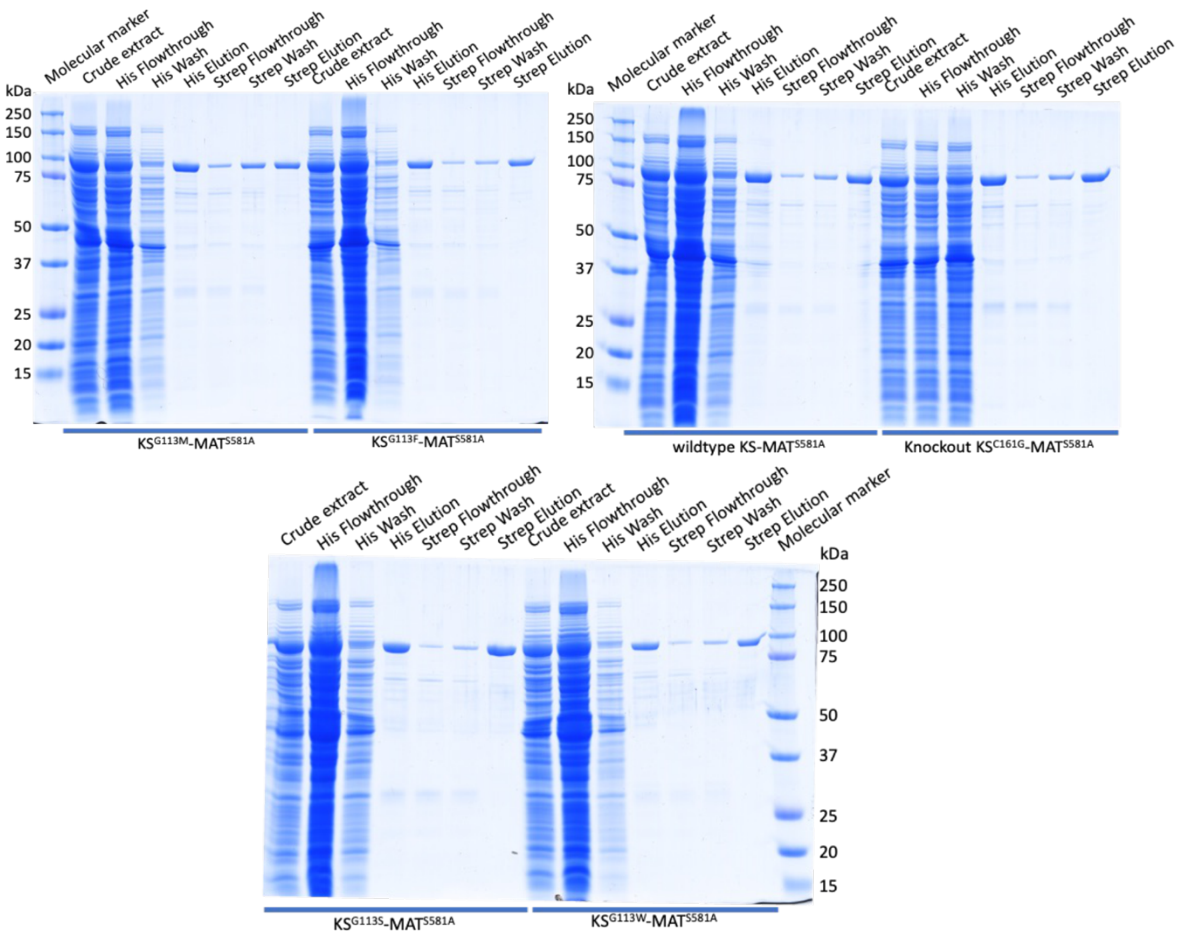

Supplementary Figure 6: Representative SDS-PAGE analysis of the KS^{G113X}-MAT^{S581A} didomain constructs (X refers to M, S, F and W, wildtype for comparison) recombinantly produced in *E. coli* for the detailed enzyme kinetic analysis of the KS. Apparent molecular weights of target proteins agree with calculated molecular weight of 97 kDa.

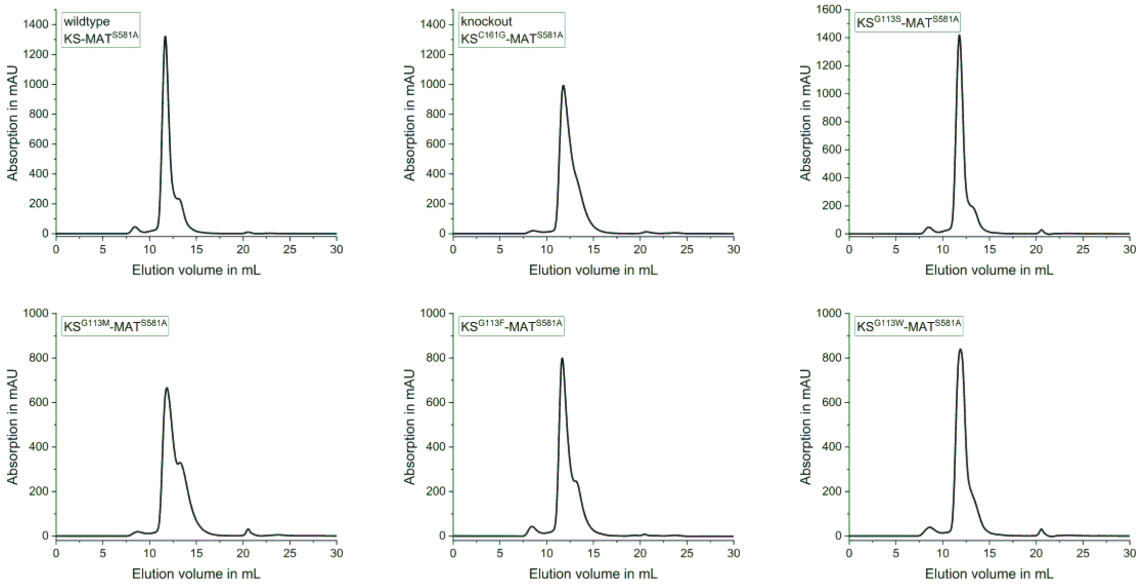

Supplementary Figure 7: Representative size-exclusion chromatograms of KS^{G113X}-MAT^{S581A} mutants expressed for the detailed kinetic analysis of the KS. All chromatograms show a prominent peak at 12 mL elution volume, in line with the preferential dimeric state of the didomain construct, and a lower peak/shoulder at 13 mL of the monomeric species. Fractions of dimeric proteins were collected and used for further experiments.

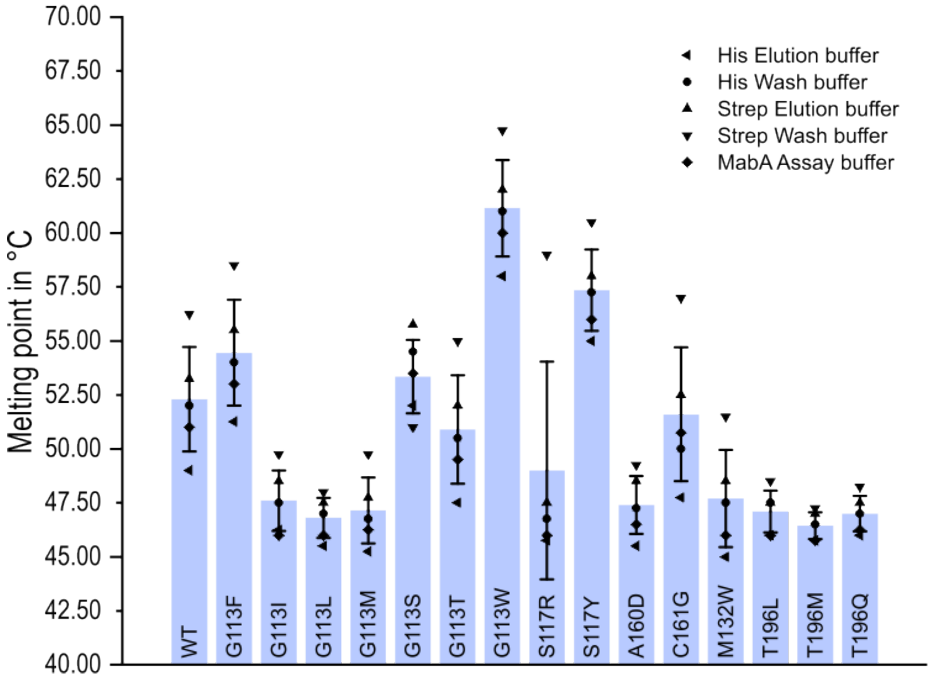

Supplementary Figure 8: Thermal shift assay of KS^{G113X}-MAT^{S581A} mutants, as well as other KS mutants, recorded in in five different buffers (His Wash, His Elution, Strep Wash, Strep Elution, and MabA assay buffer). Data is represented as mean +/- SD of the five different buffers.

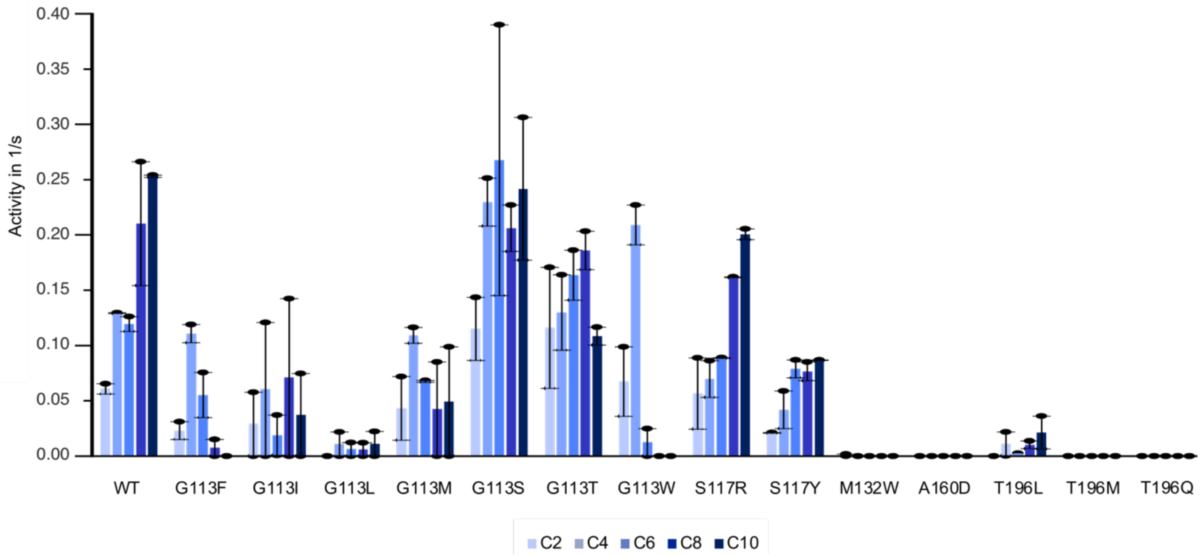

Supplementary Figure 9: Screening of KS mutants. The initial reaction velocity was determined at v_{max} of 20 µM Mal-ACP in technical duplicates of the wildtype and 16 mutants for acetyl- (C2), butyryl- (C4), hexanoyl- (C6), octanoyl- (C8) and decanoyl- (C10) ACP using the MabA assay. Error bars indicate the propagated error of standard deviations of blank measurement (KS knockout) and positive measurement.

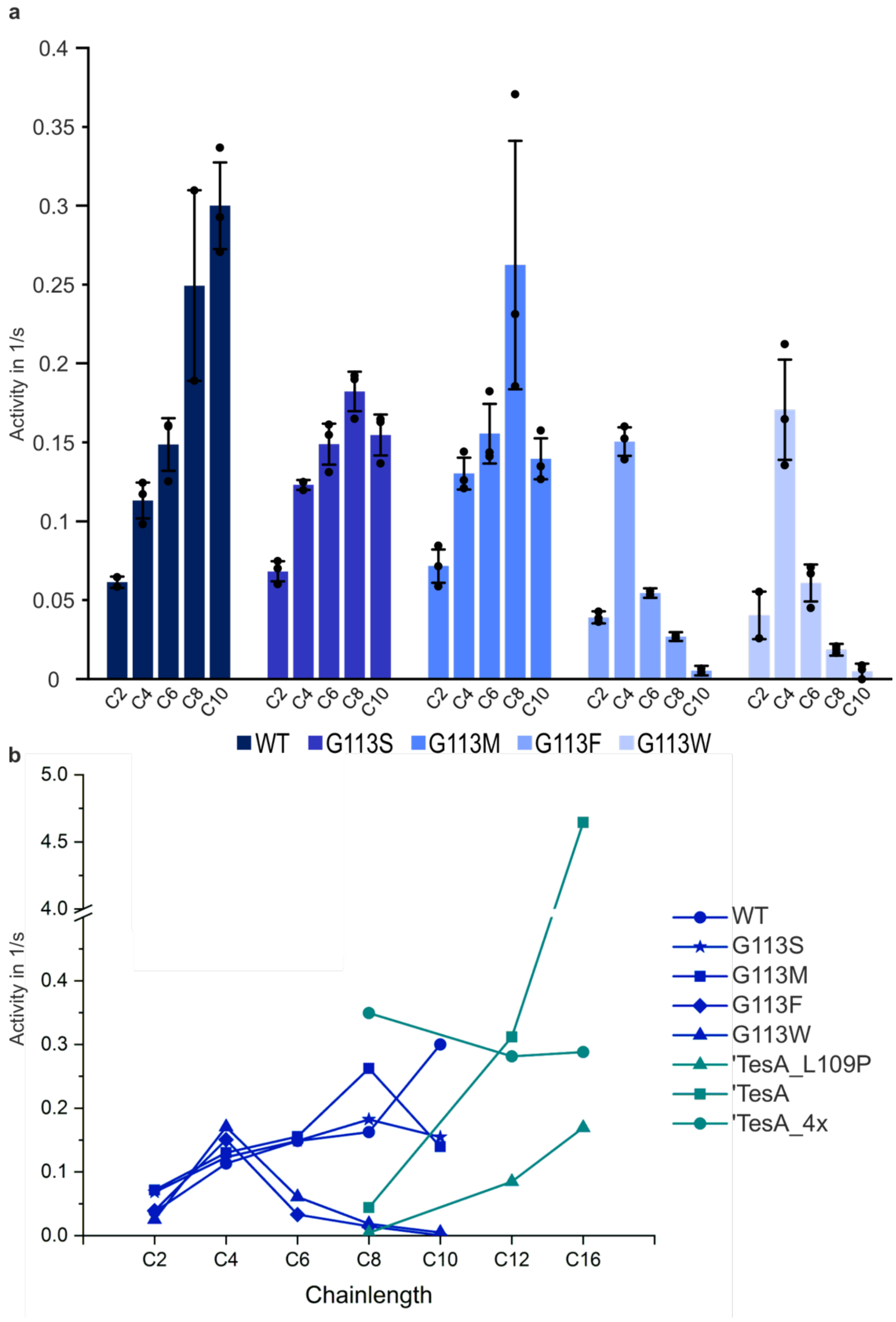

Supplementary Figure 10: KS elongation rates. (a) KS elongation activity of the WT and four G113 point mutations for chain lengths between C2 and C10. The rates represent the mean of three biological replicates and are given in 1/s. The error bars reflect the error propagation of blank (knockout) and positive (mutant) measurements standard deviation. (b) Apparent rates for the substrate octanoyl-ACP of five KS variants (blue) and three 'TesA variants (turquoise) in 1/s. Data for the KS mutations was recorded in this study, data for the 'TesA variants is from Deng et al.[3]

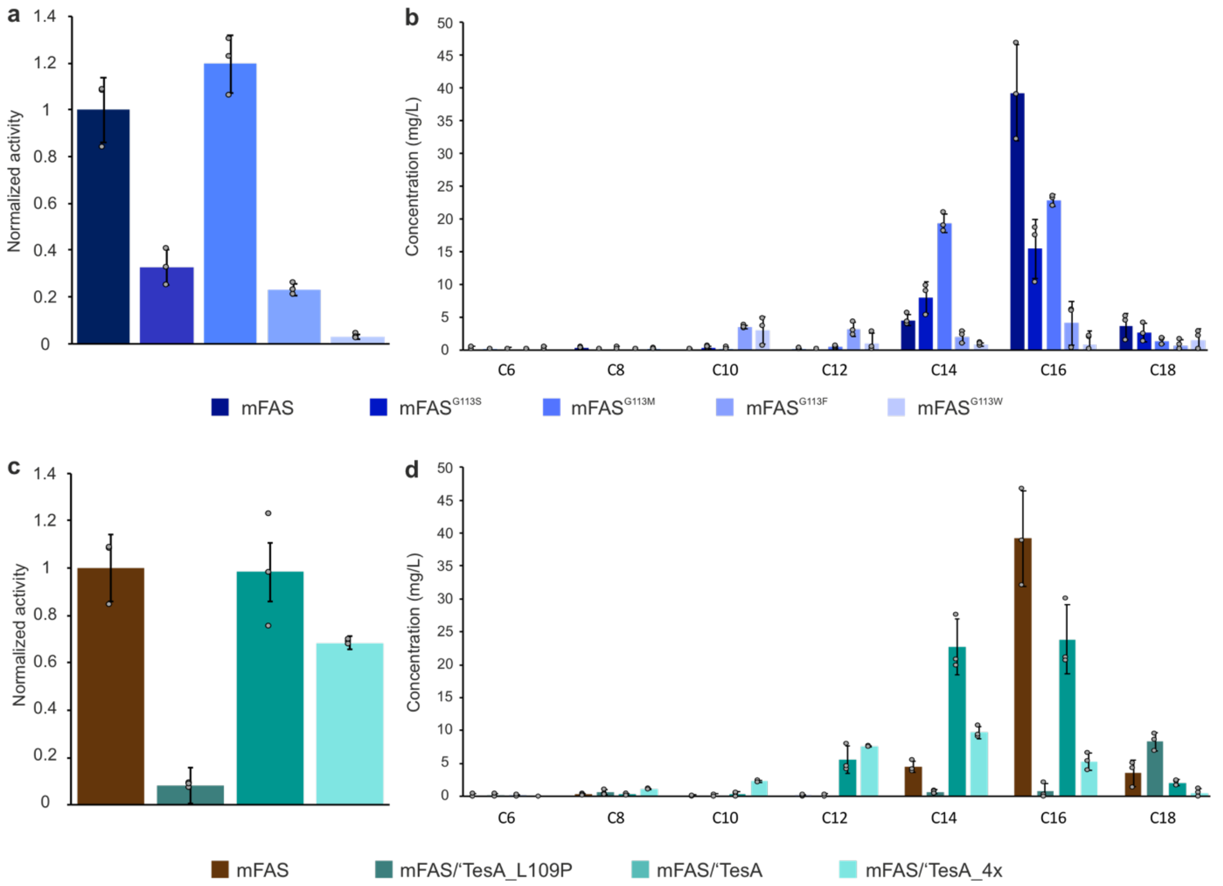

Supplementary Figure 11: *In vitro* assays of mFAS KS-mutants and mFAS/'TesA hybrids. (a) Normalized activity of five mFAS mutated in KS at position 113 (data collected in biological triplicates with three technical replicates each). NADPH decrease was monitored via its fluorescence (excited 348-320 nm, detected 470-420 nm) over time. Activities of variants were normalized to WT activity. This figure panel is also shown in Figure 2e, and here included for clarity. (b) FAs were produced in vitro by incubation of the enzyme, Ac-Coa, Mal-CoA and NADPH overnight and measured vie gas chromatography. This is an accompanying figure to Figure 2f, where FA concentrations are sorted by mFAS variants. (c/d) Normalized activity of different mFAS/'TesA hybrids mutated in 'TesA; including wildtype mFAS (mFAS_WT) as reference. Data collection as described for (a and b) in biological triplicates with three technical replicates each. Data presented as the mean +/- SD of three biological replicates. Figure panels c and d are accompanying figures to Figure 3a and b.

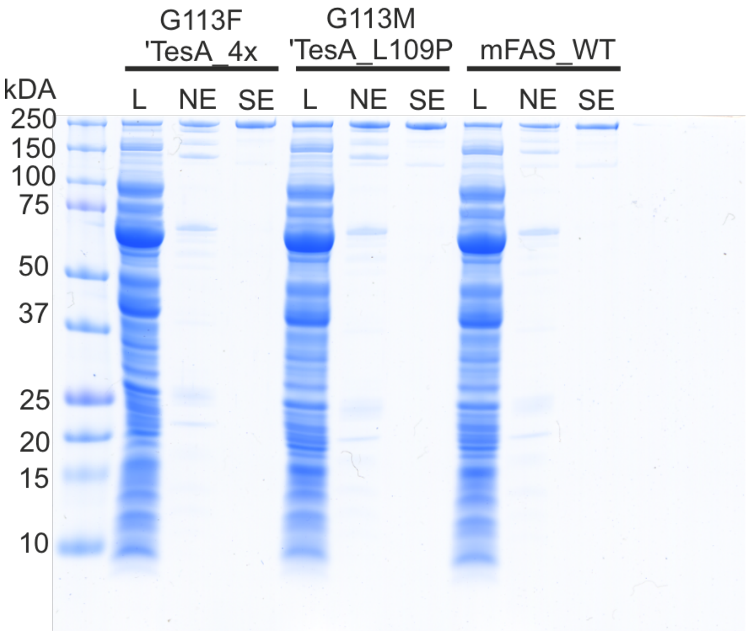

Supplementary Figure 12: Representative analysis of protein purity by SDS-PAGE for selected mFAS/'TesA constructs. A 10% Schägger-gel was used. Samples are lysate (L), elution from the nickel-chelating column (NE) and elution from the STREP-tactin column (SE). Apparent molecular weights of target proteins agree with calculated molecular weight of 270 kDa. Proteins are pure after the STREP column, as judged from SE lanes.

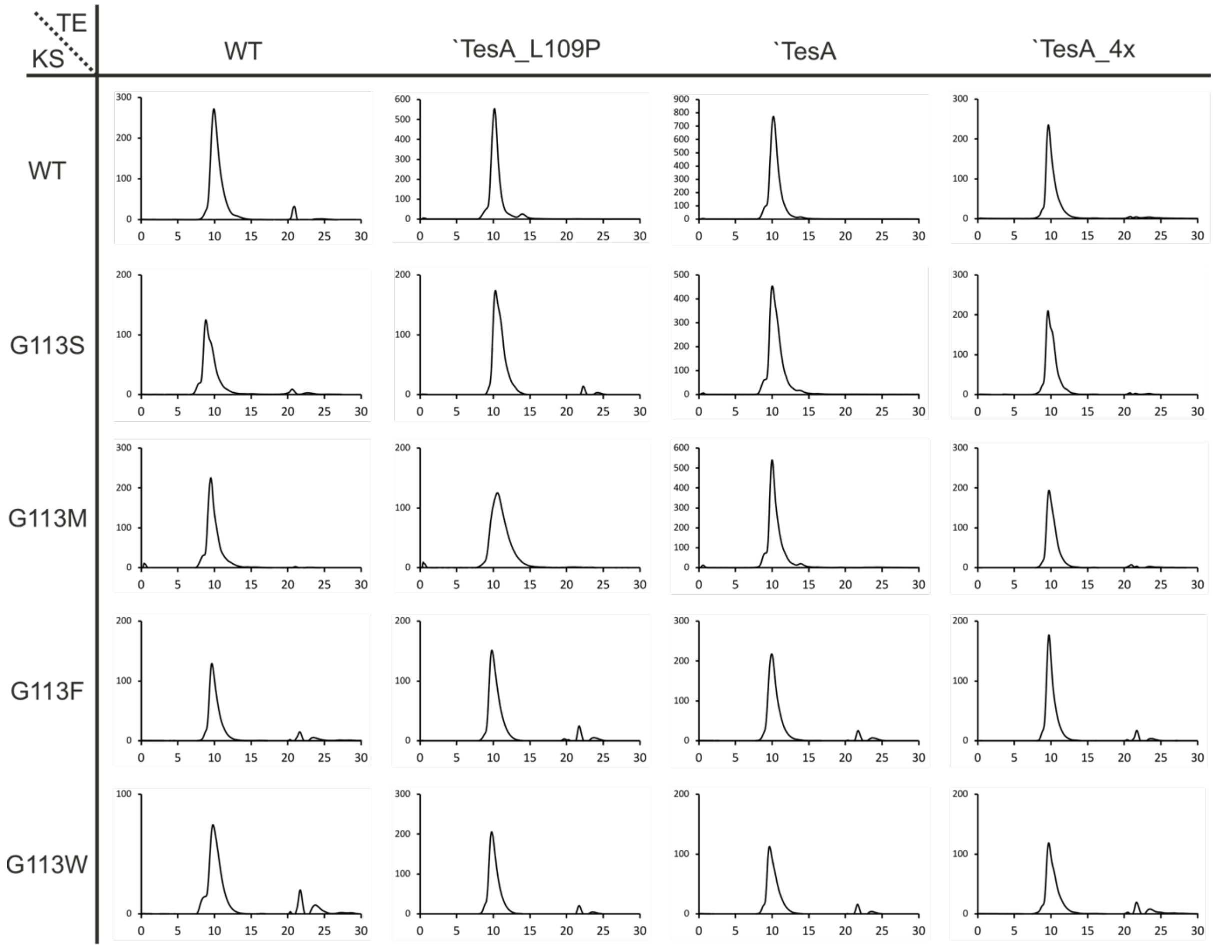

Supplementary Figure 13: Analysis of protein purity by SEC for the 20 mFAS/'TesA constructs used in this study. X-axis shows the elution volume in mL, Y-axis shows the 280 nm absorption in mAU. Proteins elute as dimers mainly, with varying amounts of monomeric species. Just proteins in dimeric state were used for in vitro experiments.

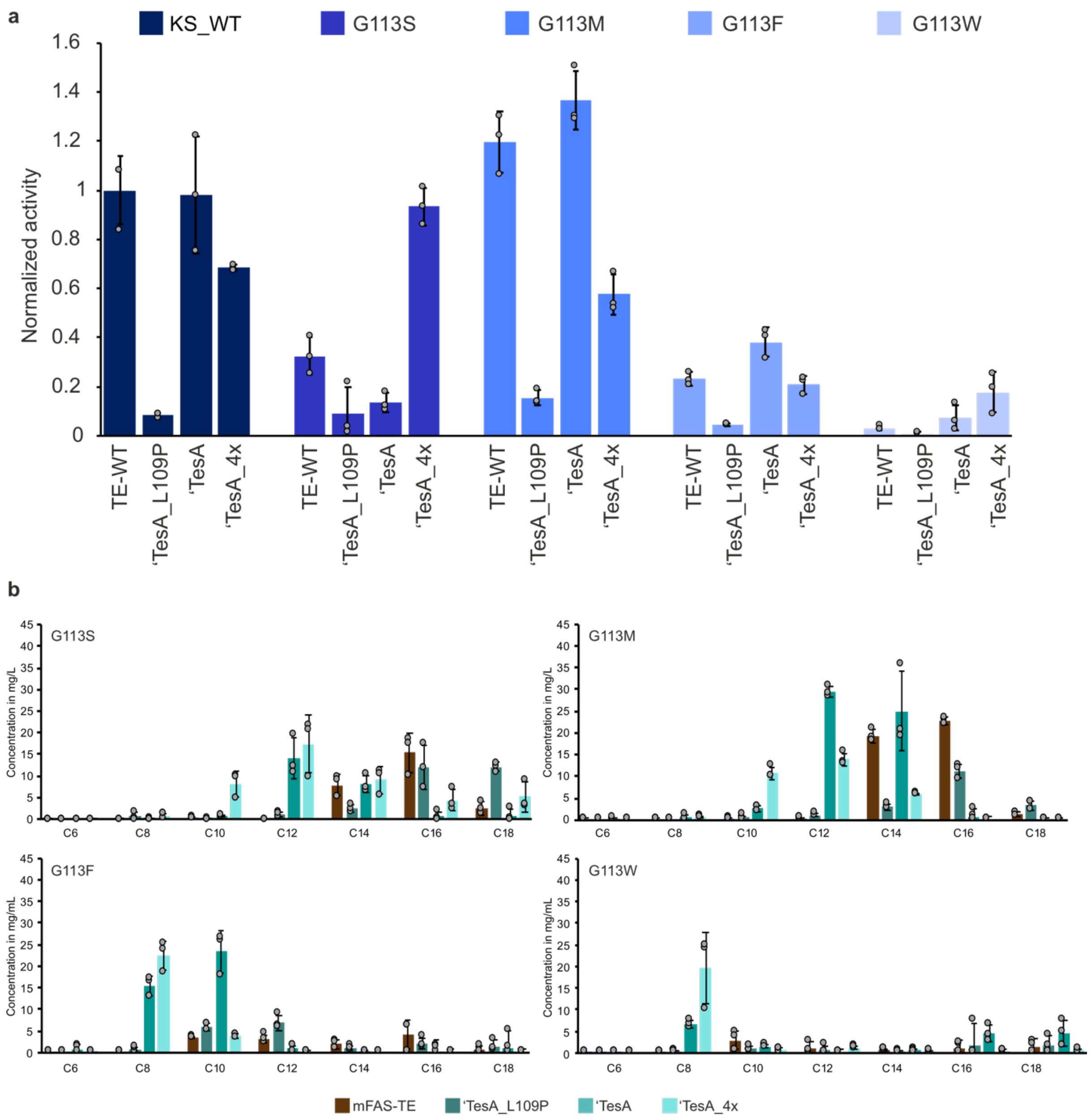

Supplementary Figure 14: Normalized activity and product spectra of mFAS-hybrids. (a) Normalized activity of mFAS variant harboring KS mutated to G113S, G113M, G113F or G113W (or non-mutated WT KS) combined and one of the three 'TesA variants 'TesA, 'TesA_L109P or 'TesA_4x (or mFAS TE mTE). Data are normalized to WT activity (373 nmol/min/mg protein), and were collected in biological triplicates with three technical replicates each. This is an accompanying figure to Figure 3a. (b) Product distributions of mFAS-hybrids grouped by KS mutants (data collected in biological triplicates with three technical replicates each). FAs were produced in vitro by incubation of the enzyme, Ac-Coa, Mal-CoA and NADPH overnight and measured via gas chromatography. Data presented as the mean +/- SD of three biological replicates. This is an accompanying figure to Figure 3b.

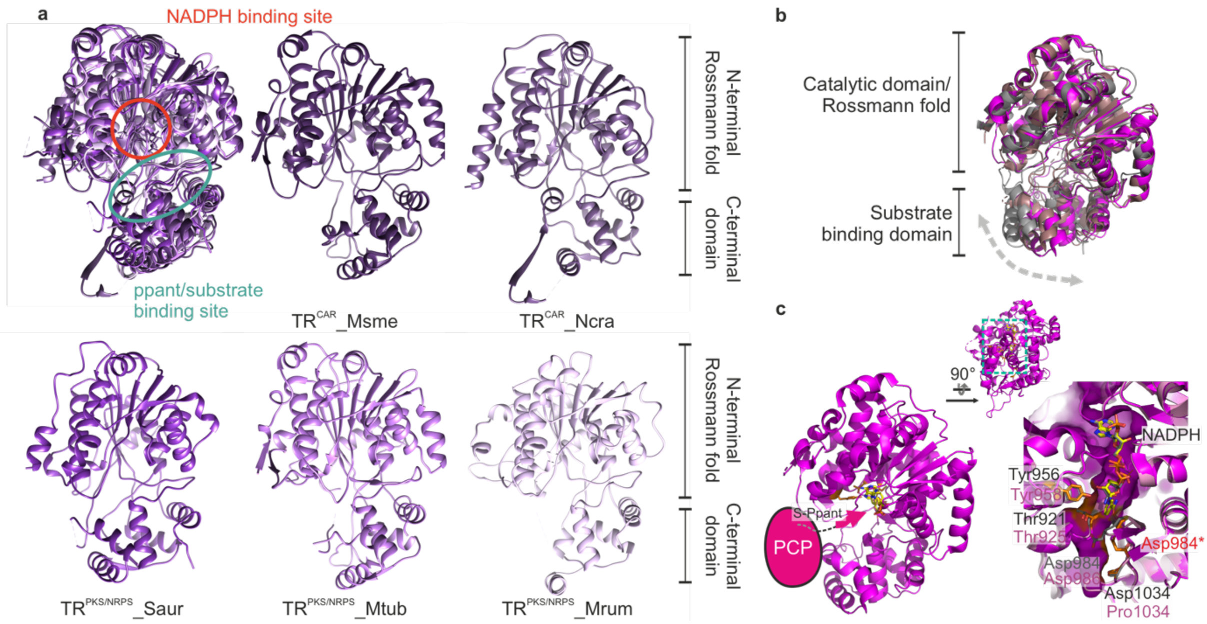

Supplementary Figure 15: Thioreductase domains used in mFAS/TR engineering. (a) Structures of TR domains used in this study for which X-ray structures are available. Overlayx of the five structures (top left) showing a well conserved Rossmann-fold with its NADPH binding site and a more divergent C-terminal domain responsible for substrate recognition. PDB ID codes are: 5MSO (TR^{CAR}_Mmar), 8AEP (TR^{CAR}_Ncra), 4W4T (TR$^{PKS/NRPS}$_Saur), 4DQV (TR$^{PKS/NRPS}$_Mtub) and 6VTZ (TR$^{PKS/NRPS}$ _Mrum). Thioreductase domains used in mFAS/TR engineering. (b) Superposition of TR^{CAR} from Mycobacterium marinum (magenta, PDB ID: 5msu; 60% sequence identical to M. smegmatis TR^{CAR}), and TRs$^{PKS/NRPS}$ from M. tuberculosis (purple, PDB ID: 4u5q, 55%) and Stigmatella aurantiaca (grey, PDB ID: 4u7w, 30%). The overall conserved fold shows rigid body like conformational variability between the catalytic domain and the substrate binding domain that assists substrate binding (grey arrow). (c) M. marinum TR^{CAR} (PDB ID: 5msu) with selected residues in the active site and binding tunnel highlighted in stick representation. Zoom in active site reveals conformational dynamics of the loop carrying Asp984 (M. marinum numbering; M. smegmatis number attached in purple) switching between the active on-state (Asp984*) and the inactive off-state. Active site and binding site residues are highlighted in stick representation. Bound NADPH is shown in yellow.

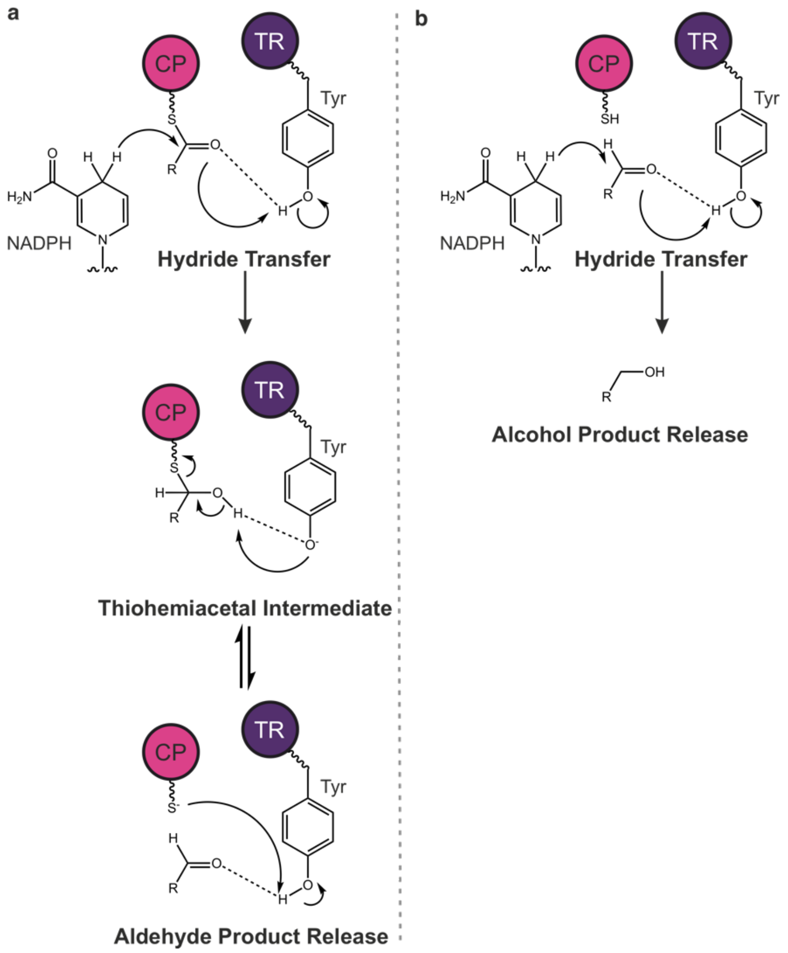

Supplementary Figure 16: Mechanism of TR-mediated reduction and product release. (a) (First) 2-electron reduction. Carrier protein (CP) bound substrate is reduced to the corresponding aldehyde using one NADPH as hydride-donor. (b) Second 2-electron reduction. Formed aldehyde can be reduced to alcohol by a second equivalent of NADPH. Figure adapted from Mullowney et al.[4]

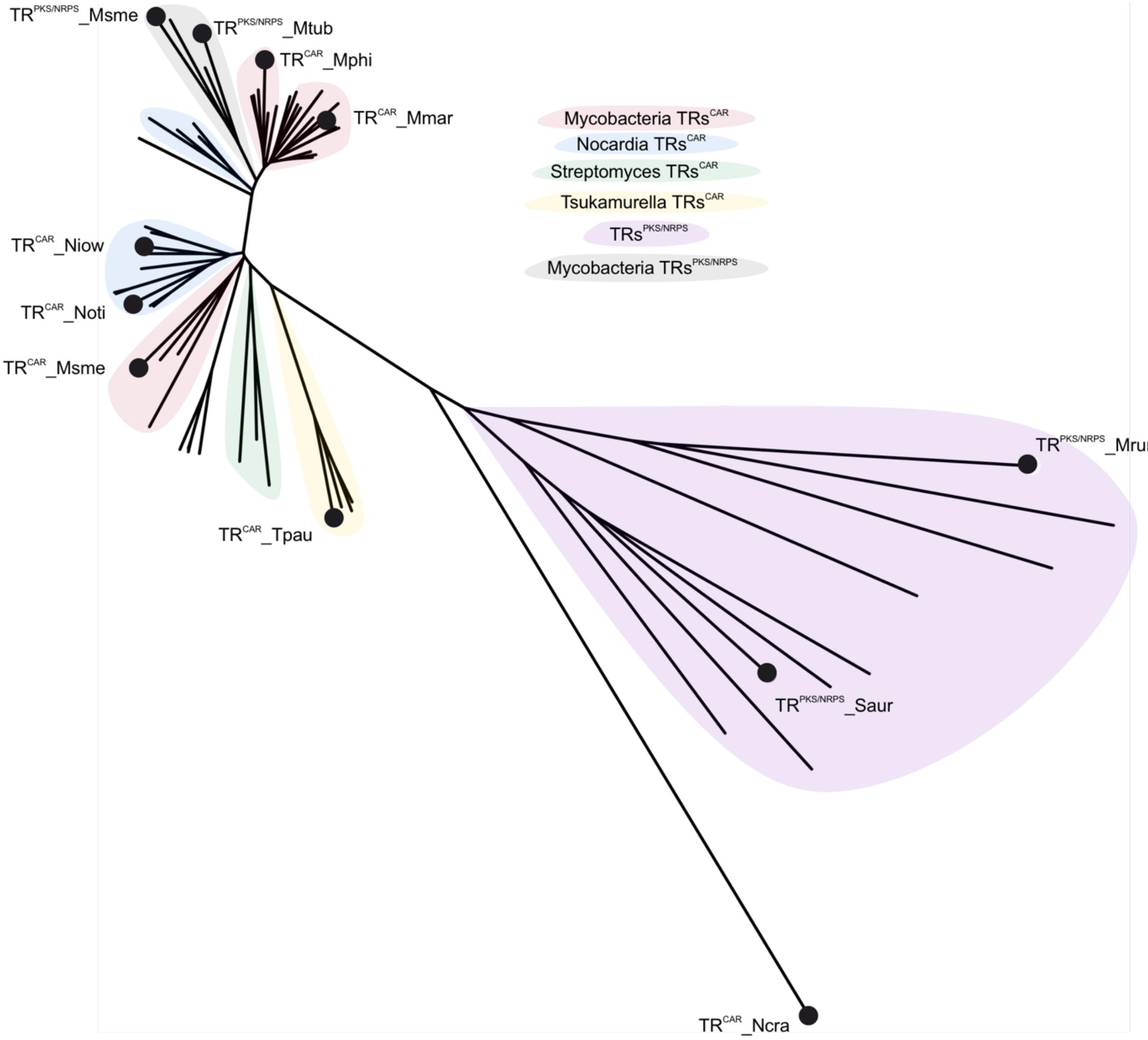

Supplementary Figure 17: Phylogenetic tree of TRs^{CAR} and TRs$^{PKS/NRPS}$. Sequences from the TR^{CAR}s were used from Finnigan et al.[5] and sequences from TRs$^{PKS/NRPS}$ were retrieved from Uniprot and Genbank. Sequence alignment was done using aMAFFT, and the tree was calculated using BLOSUM62. Groups of TRs were colored and annotated. TR^{CAR}-Ncra poses as outlier since it is a fungal TR.

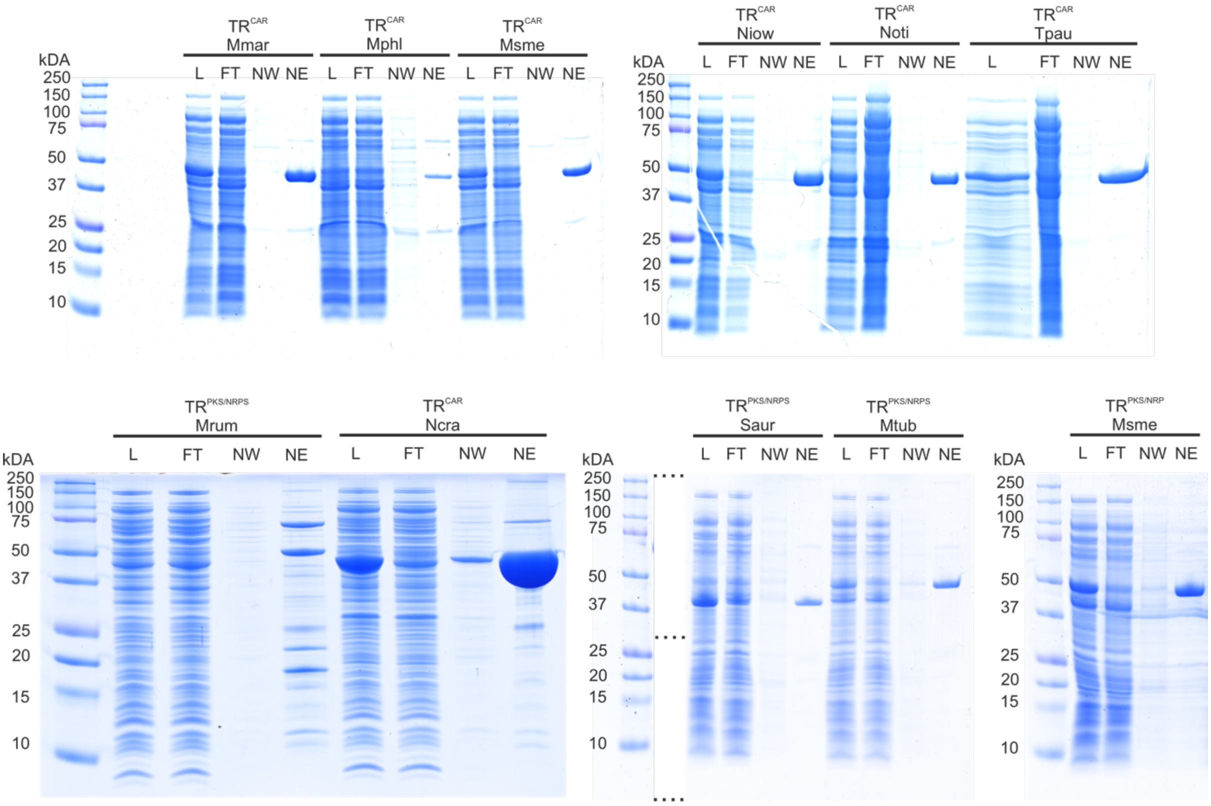

Supplementary Figure 18: SDS-PAGE of TR-domains. A 10% Schägger-gel was used. Samples are lysate (L), flowthrough (FT), wash (NW) and elution from the nickel-column (NE). Apparent molecular weights of target proteins agree with the calculated weights. All proteins are pure after the nickel-chelating column, as judged from NE lanes.

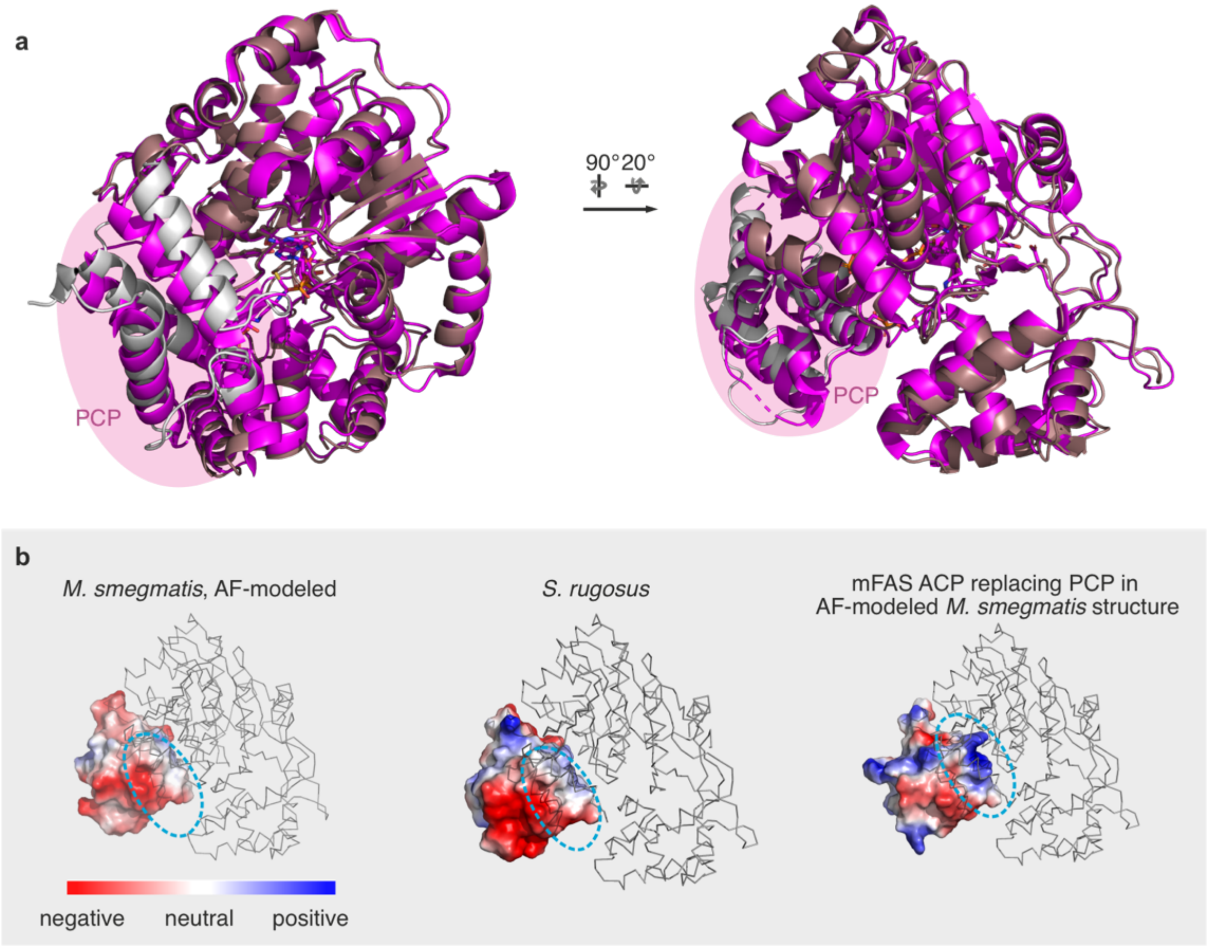

Supplementary Figure 19: Interaction of carrier protein with TR^{CAR}. a PCP-TR^{CAR} from *Segniliparus rugosus* with docked PCP and phosphopantetheine extending into the binding pocket (PDB ID: 5msv) in superposition with the *M. smegmatis* PCP-TR^{CAR} AlphaFold3 model. Structures align with rmsd of 0.85 Å. b Vacuum electrostatics indicated for PCP of modeled *M. smegmatis* PCP-TR^{CAR} (right/top), for *S. rugosus* PCP-TR^{CAR} (middle) and mFAS ACP modeled to the PCP atomic coordinates of *M. smegmatis* PCP-TR^{CAR} AlphaFold3 model with Modeller (bottom). Different surface potentials indicate a potential mismatch in polarity between the mFAS ACP and the TR interface and suboptimal electrostatic complementarity.

```
           ACP α4          10         20         30  TE α1
                           |          |          |
    mFAS  MSSKTDSATDTTA----PKSRSDTSLKQNQLNLSTL-
mFAS_R2   MSSKTDSAAATARRPGDGRPTFAGVHGDDAAEVHARD
mFAS_R3   MSSK---AAAAQRTAGDRRPSFTTVHGADATEIRASE
mFAS_R5   MSSKTDSAAASERDSG-SRPTAATVHGDDGL-LRADD
                                            TR- α1/β1
```

Supplementary Figure 20: Sequence alignment of linkers in mFAS_WT and mFAS/TR hybrids. Alignment was done using MAFFT webserver with default settings. C-terminal structural element of mFAS ACP (pink), N-terminal structural element of mFAS TE (brown) and N-terminal structural element TRs (purple) are highlighted. Linker lengths are 25 (mFAS), 29 (mFAS/TR$^{\#2}$), 26 (mFAS/TR$^{\#3}$) and 27 (mFAS/TR$^{\#5}$).

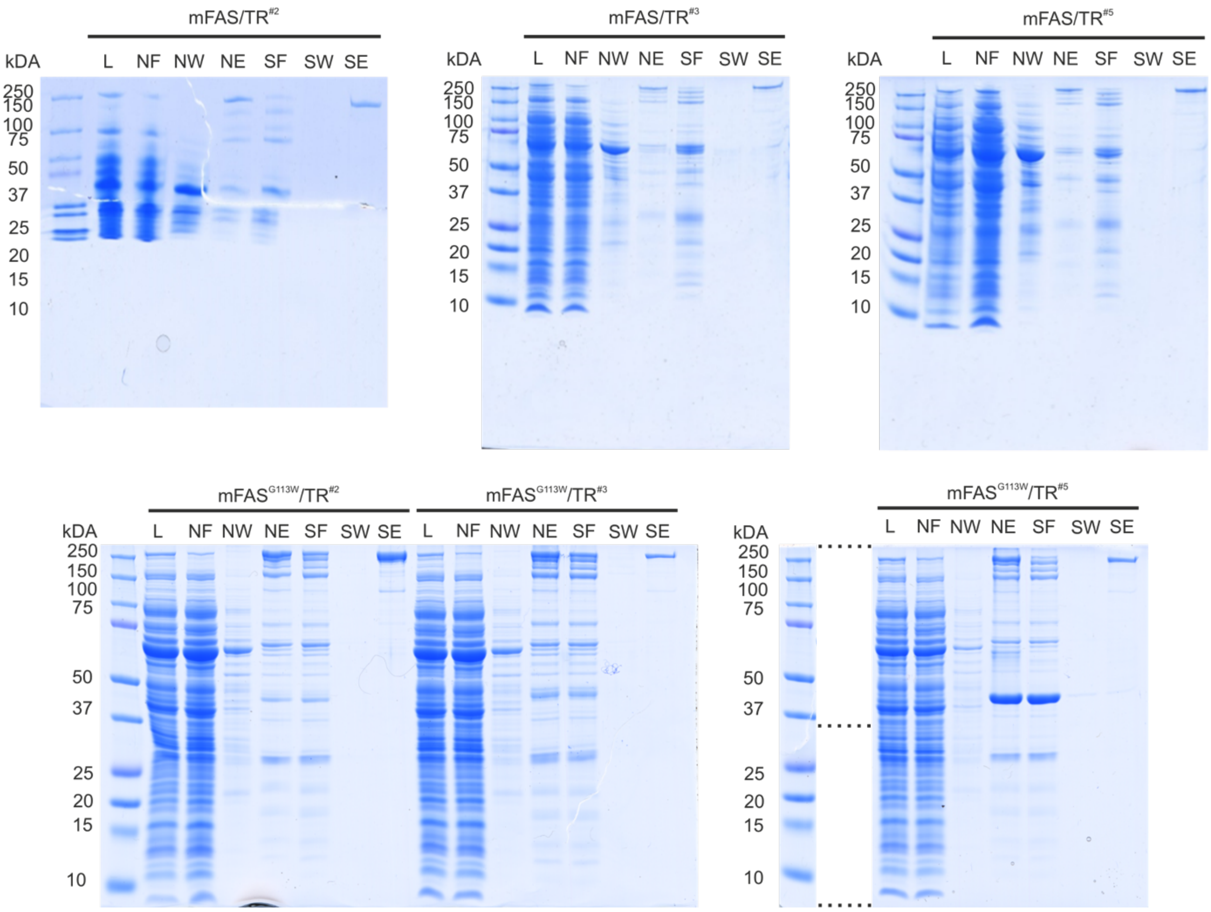

Supplementary Figure 21: SDS-PAGE for mFAS/TR hybrids. A 10% Schägger-gel was used. Samples are lysate (L), flowthrough (NF), wash (NW) and elution from the nickel-column (NE) and flowthrough (SF), wash (SW) and elution from the strep-column (SE). Apparent molecular weights of target proteins agree with the calculated weights of about 270 kDa. All proteins are pure after the STREP-tactin column, as judged from SE lanes.

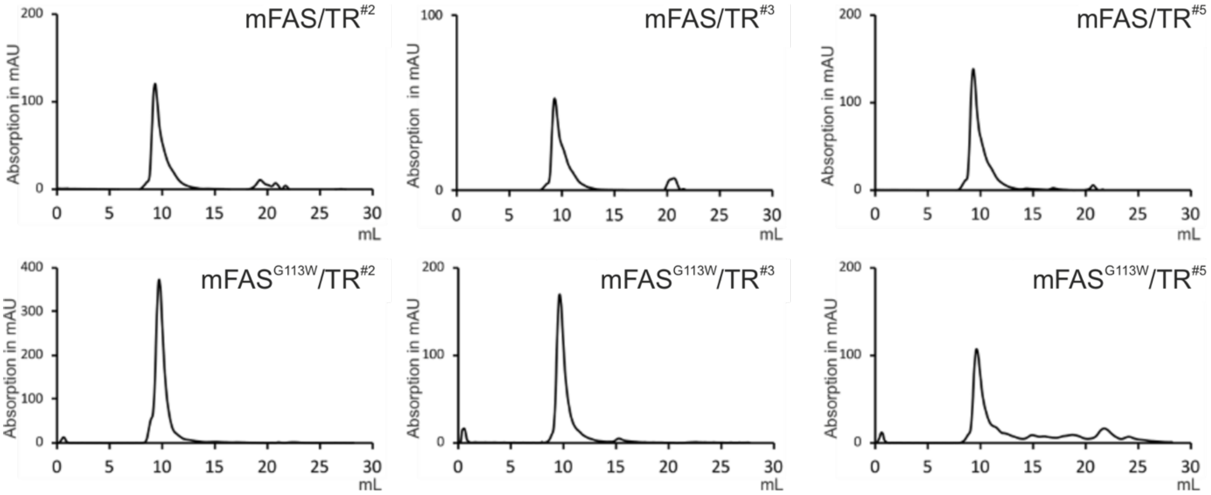

Supplementary Figure 22: Analysis of protein purity by SEC for the mFAS/TR hybrids. All proteins are pure, and mainly in dimeric form. If there was a shoulder present – indicating the monomer – only dimeric fractions were used.

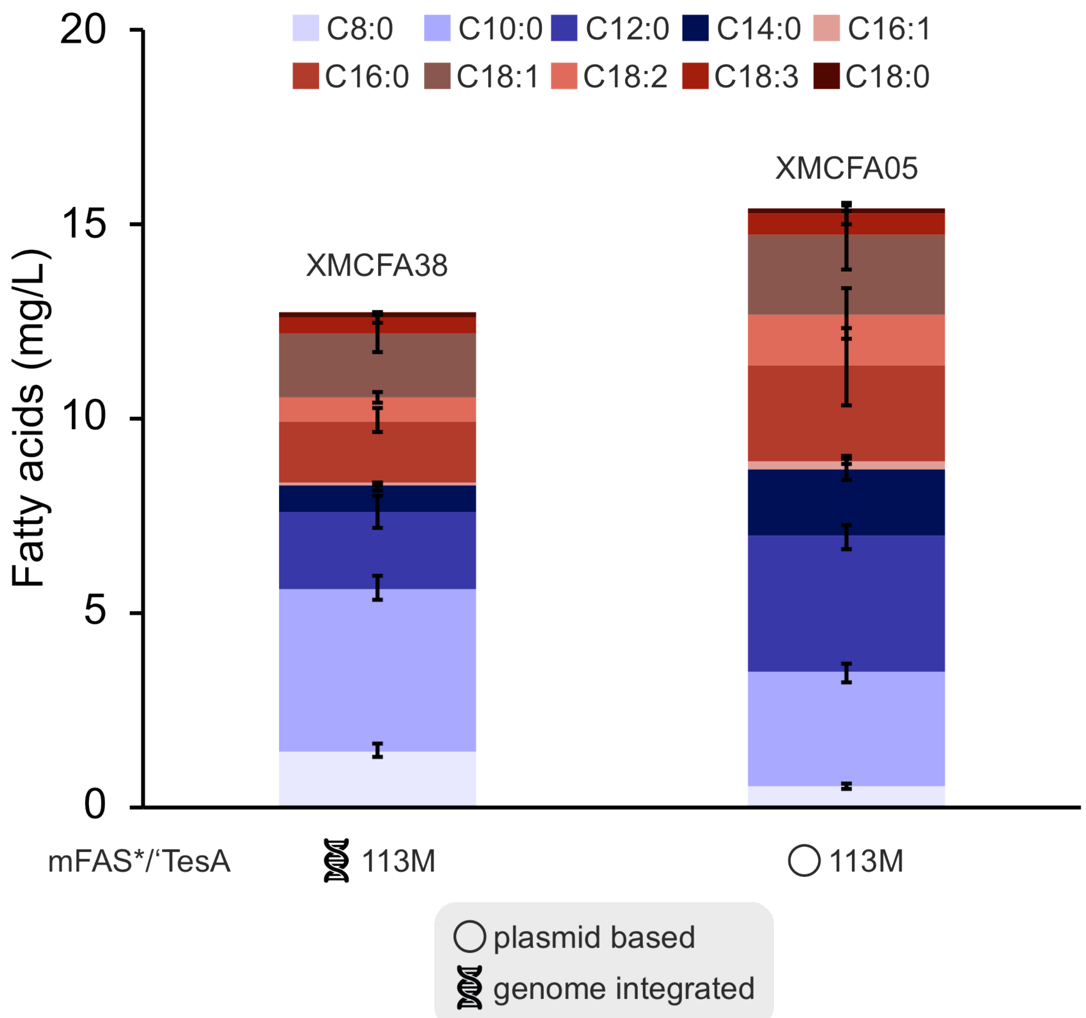

Supplementary Figure 23: Production of free FAs by using mFAS^{G113M}/'TesA hybrids with different overexpression forms. Production of free FAs by a *O. polymorpha* strain with mFAS^{G113M}/'TesA hybrid. mFAS^{G113M}/'TesA hybrid was encoded from plasmid (XMCFA05) and genome (XMCFA38), respectively. *O. polymorpha* strains were cultivated in minimal medium containing 20 g/L glucose. All data presented as the mean ± SD of three yeast clones.

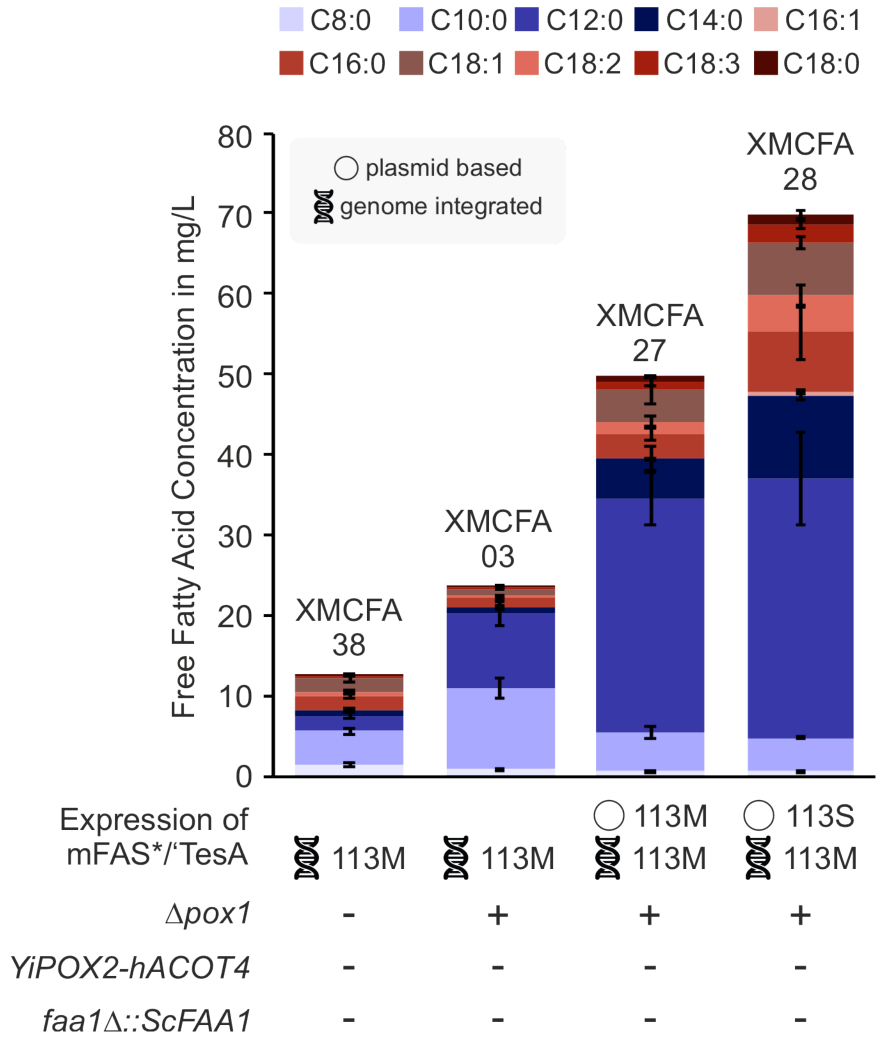

Supplementary Figure 24: Blocking β-oxidation for production of free fatty acids by *O. polymorpha* strains with mFASG113M/'TesA integrated in the genome. The latter three strains carry a POX1 knockout to reduce loss of FAs due to β-oxidation, and the latter two strains (XMCFA27 and XMCFA28) have increased copy of mFAS hybrids through additional plasmid-based expression of mFASG113S/'TesA and mFASG113M/'TesA, respectively. All data presented as the mean ± SD of three yeast clones.

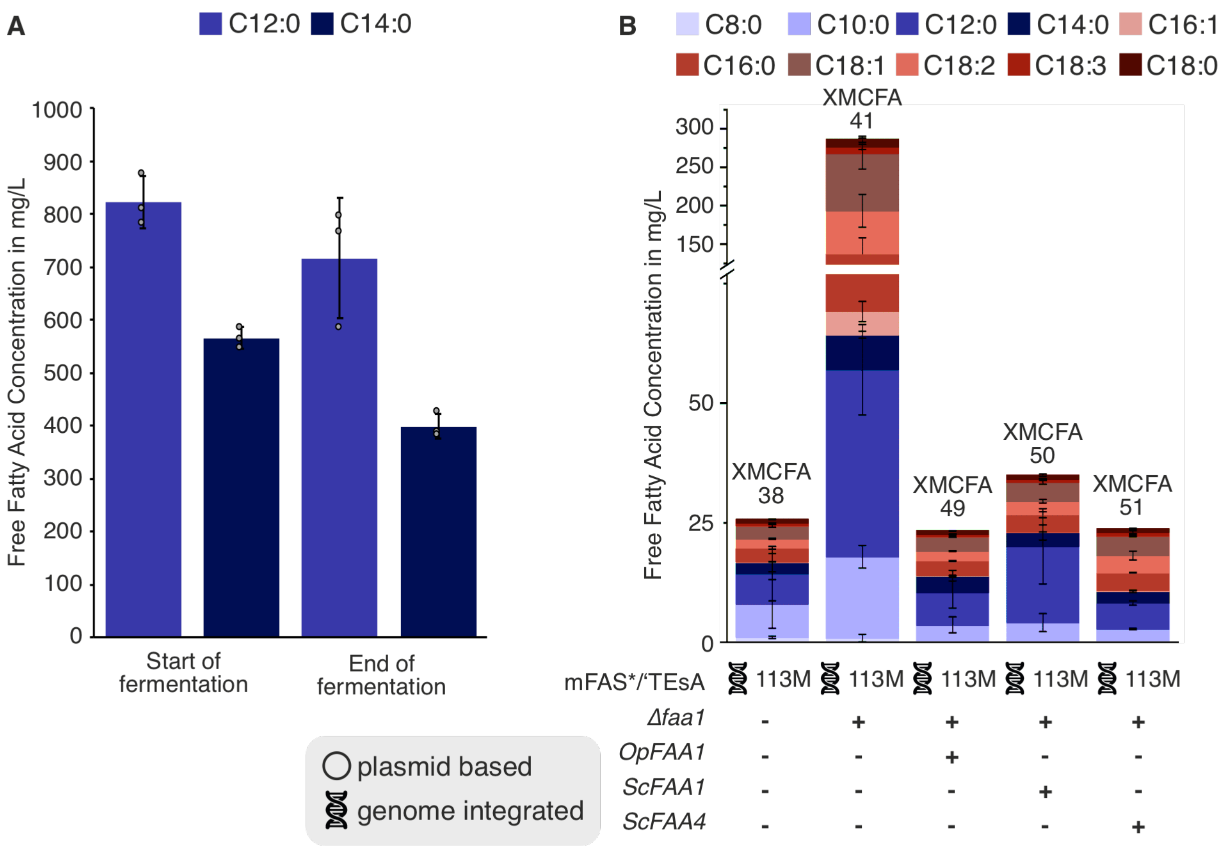

Supplementary Figure 25: Screening long-chain fatty acyl-CoA synthases. **a** Consumption of C12/C14 FAs by wildtype *O. polymorpha* strain. **b** Replacing endogenous *FAA1* with other fatty acyl-CoA synthase genes from *S. cerevisiae* (*ScFAA1* and *ScFAA4*) in engineered *O. polymorpha* strain with genome integrated mFAS^{G113M}/'TesA. The latter four strains carry a *FAA1* knockout to reduce loss of FAs due to β-oxidation, with three strains harboring different fatty acyl-CoA synthases through genome-based expression of *OpFAA1*, *ScFAA1* and *ScFAA4*, respectively. *O. polymorpha* strains were cultivated in minimal medium containing 20 g/L glucose. All data presented as the mean ± SD of three yeast clones.

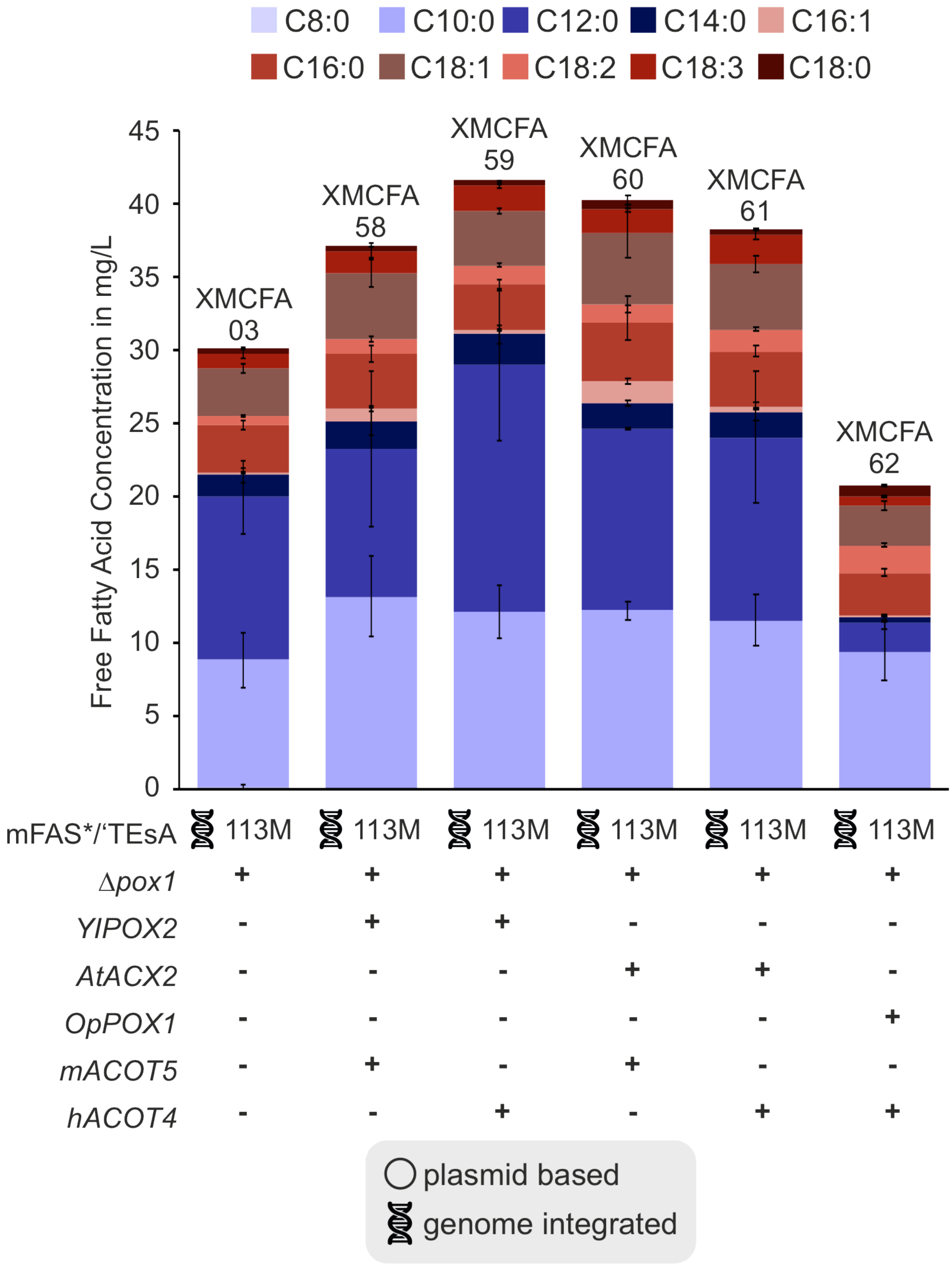

Supplementary Figure 26: Optimizing the combination of various fatty acyl-CoA oxidases and thioesterases. The *O. polymorpha* strains all harbor the mFAS^{G113M}/'TesA encoding gene integrated in the genome and the POX1 gene knockout. The latter five strains have introduced in genome different combinations of genes encoding fatty acyl-CoA oxidase (YlPOX2, AtACX2 and OpPOX1) and thioesterase (mACOT5 and hACOT4) through genome-based expression. O. polymorpha strains were cultivated in minimal medium containing 20 g/L glucose. All data presented as the mean ± SD of three yeast clones.

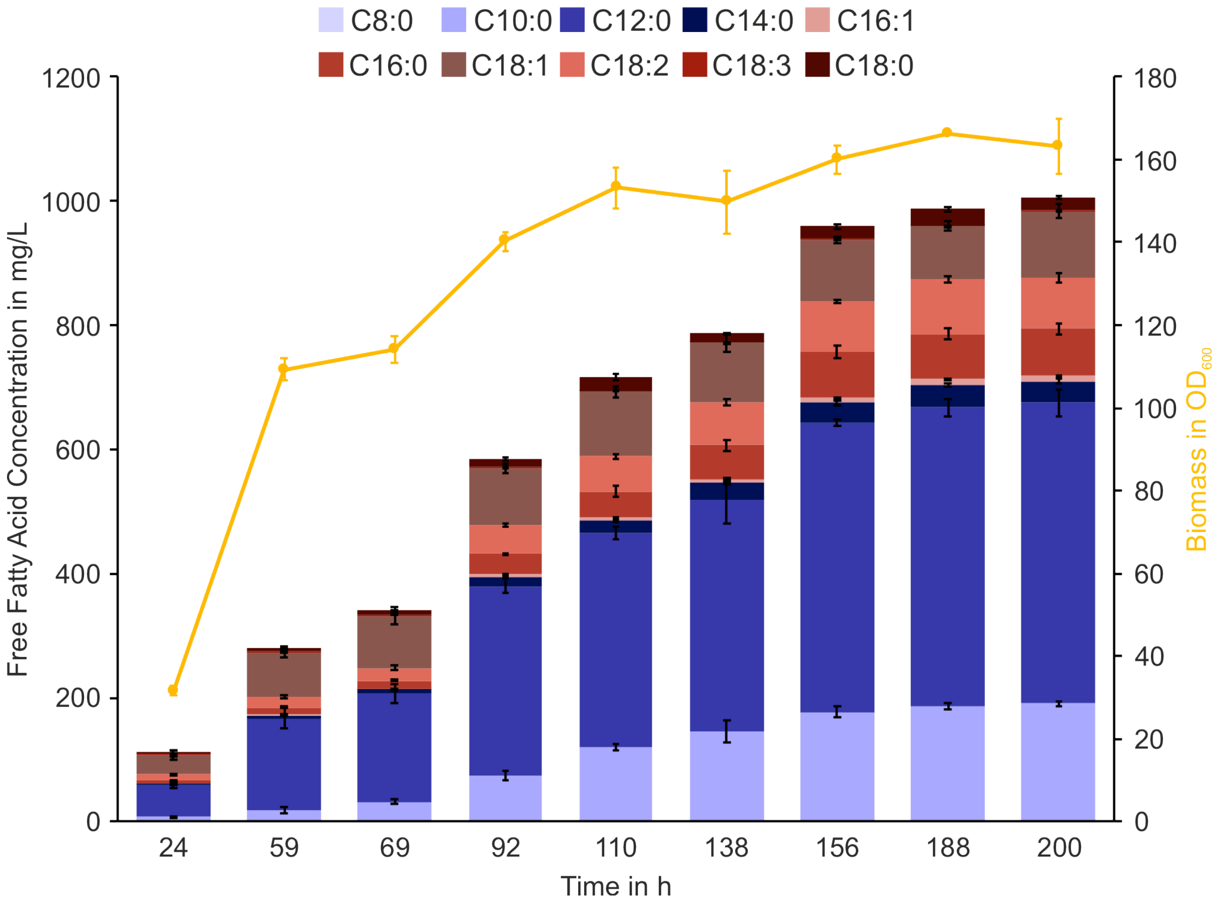

Supplementary Figure 27: Fed-batch fermentation of strain XMCFA69 in 1.5 L bioreactors. Depicted are the FFA fractions and the biomass at different timepoints. All data is the mean from three biological replicates and the depicted errors are the corresponding SD.

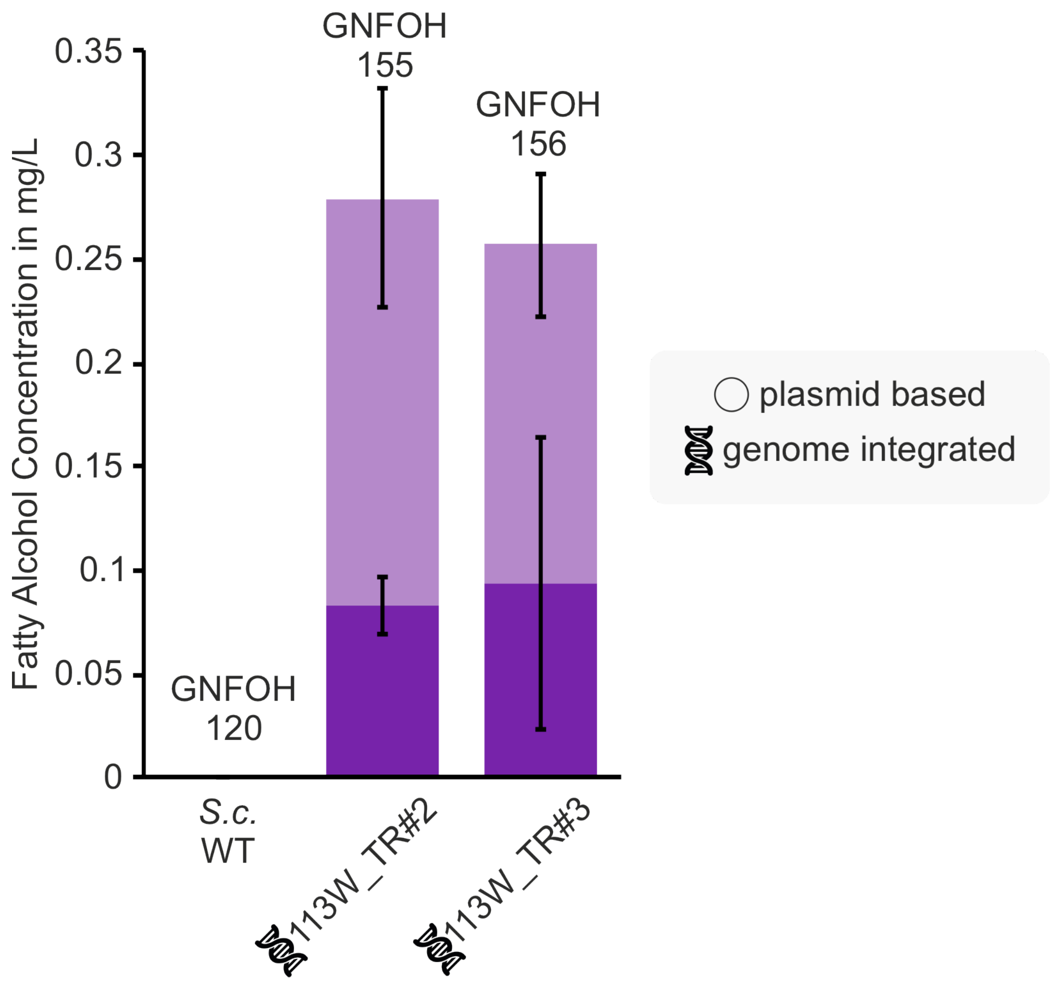

Supplementary Figure 28: Production of fatty alcohols by S. cerevisiae strains. The strains express the hybrids mFAS mFASG113W/TR#2 and mFAS mFASG113W/TR#3, respectively. O. p. and S. c. strains were cultivated in minimal medium containing 20 g/L glucose. All data presented as the mean ± SD of three yeast clones.

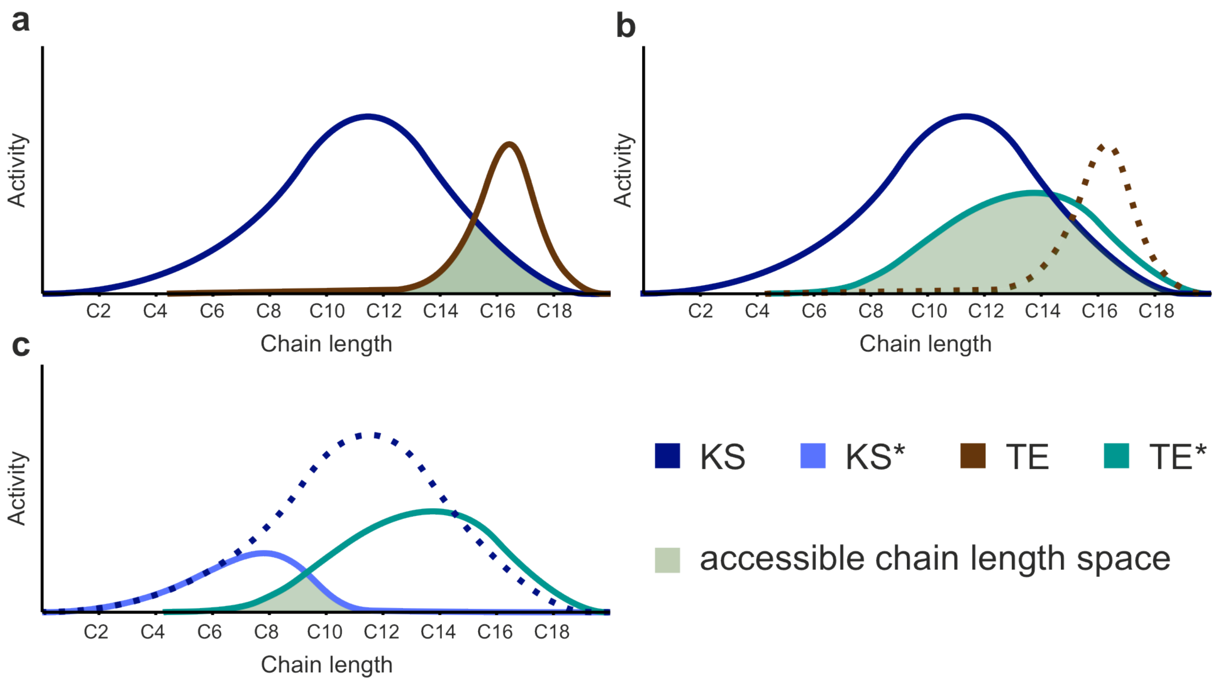

Supplementary Figure 29: Conceptual representation of the putative kinetic behavior of the interplay between KS and TE. Graphs display estimated activities of KS and TE domains for saturated acyl-ACP across different chain lengths, which will also be referred to as specificity in the following. The intersection reflects the product output spectrum. (**a**) Scheme illustrates the kinetic scenario of wildtype mFAS with its domains KS and TE. KS (dark blue) and TE (brown) reach similar peak activities, although at different chain length; KS at C12 and TE at C16. At chain length of C12, the KS-mediated elongation is dominant, while the low activity of TE at C12 prevents the TE to intercept chain extension. There is an inverse situation at C16, meaning that the KS activity is decreased at increased TE activity. Here, the TE outcompetes chain extension leading to C16-FA as main product. The competition for the acyl chain shifts further in favor of the termination reaction by the TE domain for C18 chain length, reflecting the long chain length limit of the wildtype mFAS (**b**) A thioesterase domain (termed TE*) with termination activity at C14 and generally broadened specificity replaces the native mFAS TE. The domains KS and TE share activity for a broad range of chain length; thus they are able to both act on a range of acyl-ACPs, leading to a broadened spectrum. This scenario reflects the generalist mFAS/'TesA (**c**) The generalist mFAS/TesA' exhibits susceptibility to the output chain length when modulated by KS kinetics. The chain length output spectra are shifted towards shorter chains, when the KS of the generalist mFAS/'TesA is engineered to decrease elongation activities for chains longer chains (indicated by KS*). Since elongation is hampered at longer chain length, the intersection is narrowed and product output shifted to FAs of shorter chain lengths. In this work, mFAS/'TesA with KS mutations G113S/F/W exhibit shifted FA output, with the situation depicted in (**c**) illustrating variant mFAS^{G113W}/'TesA.

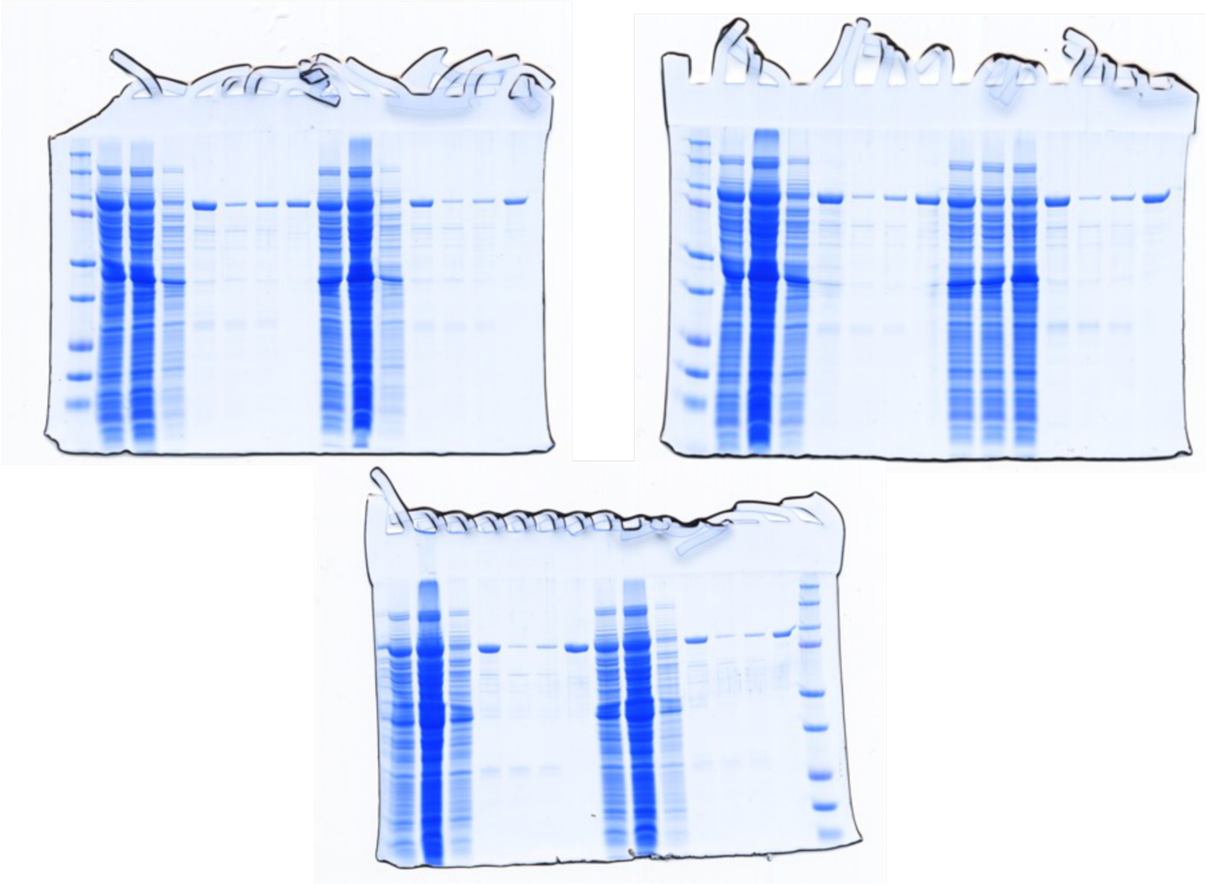

Supplementary Figure 30: Uncropped SDS-Page gels from supplementary figure 6.

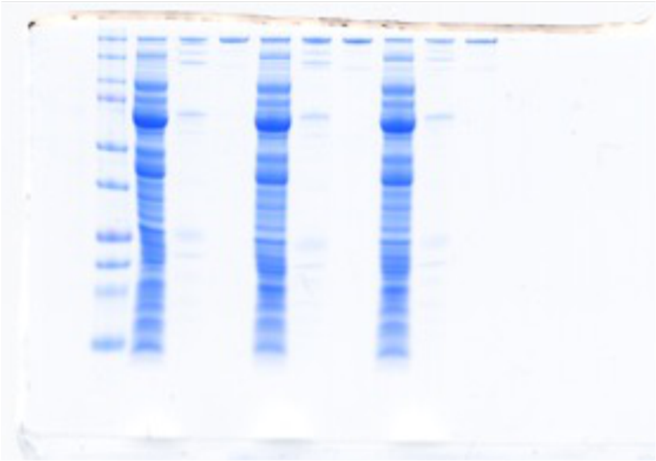

Supplementary Figure 31: Uncropped SDS-Page gels from supplementary figure 6.

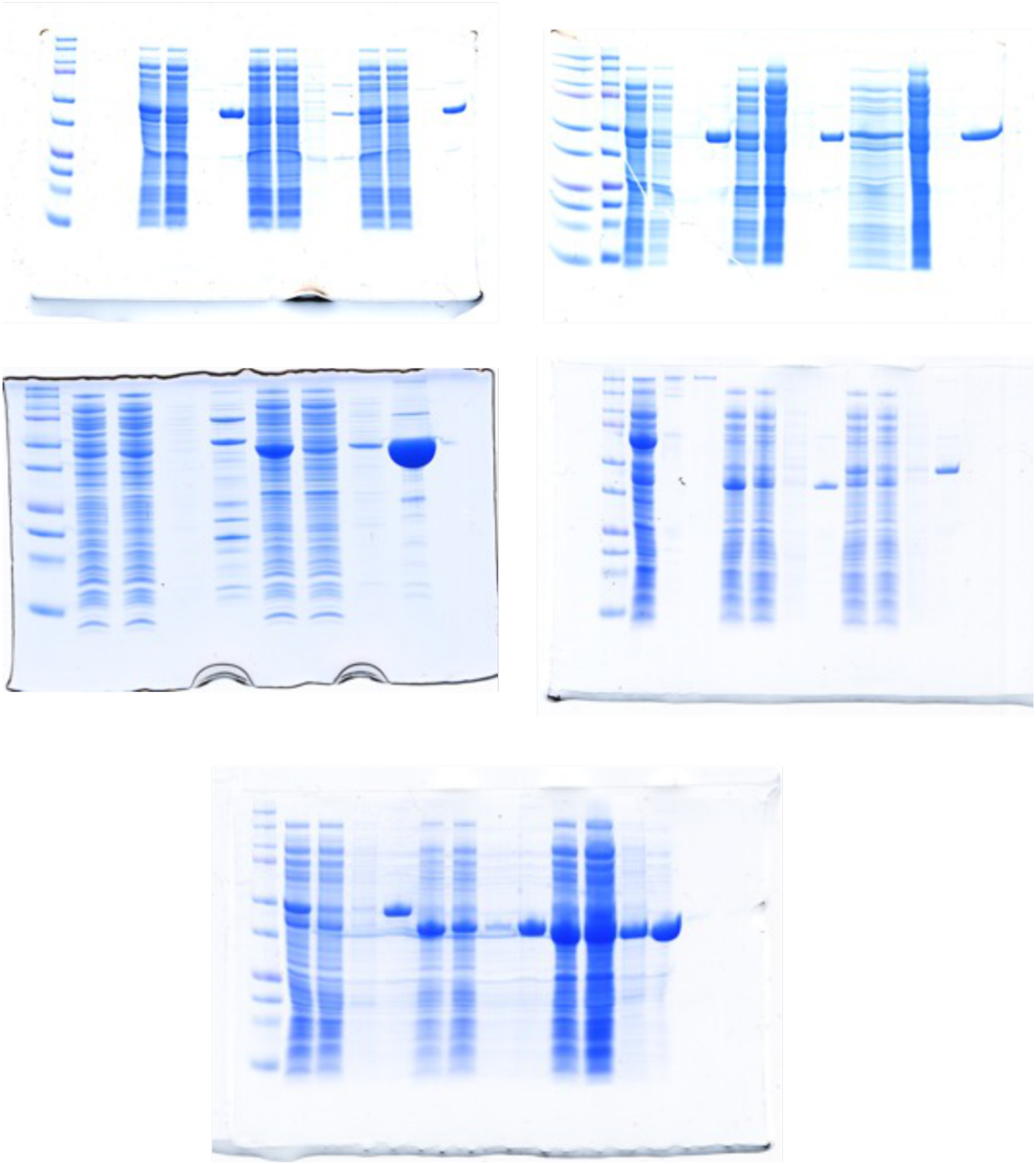

Supplementary Figure 32: Uncropped SDS-Page gels from supplementary figure 18.

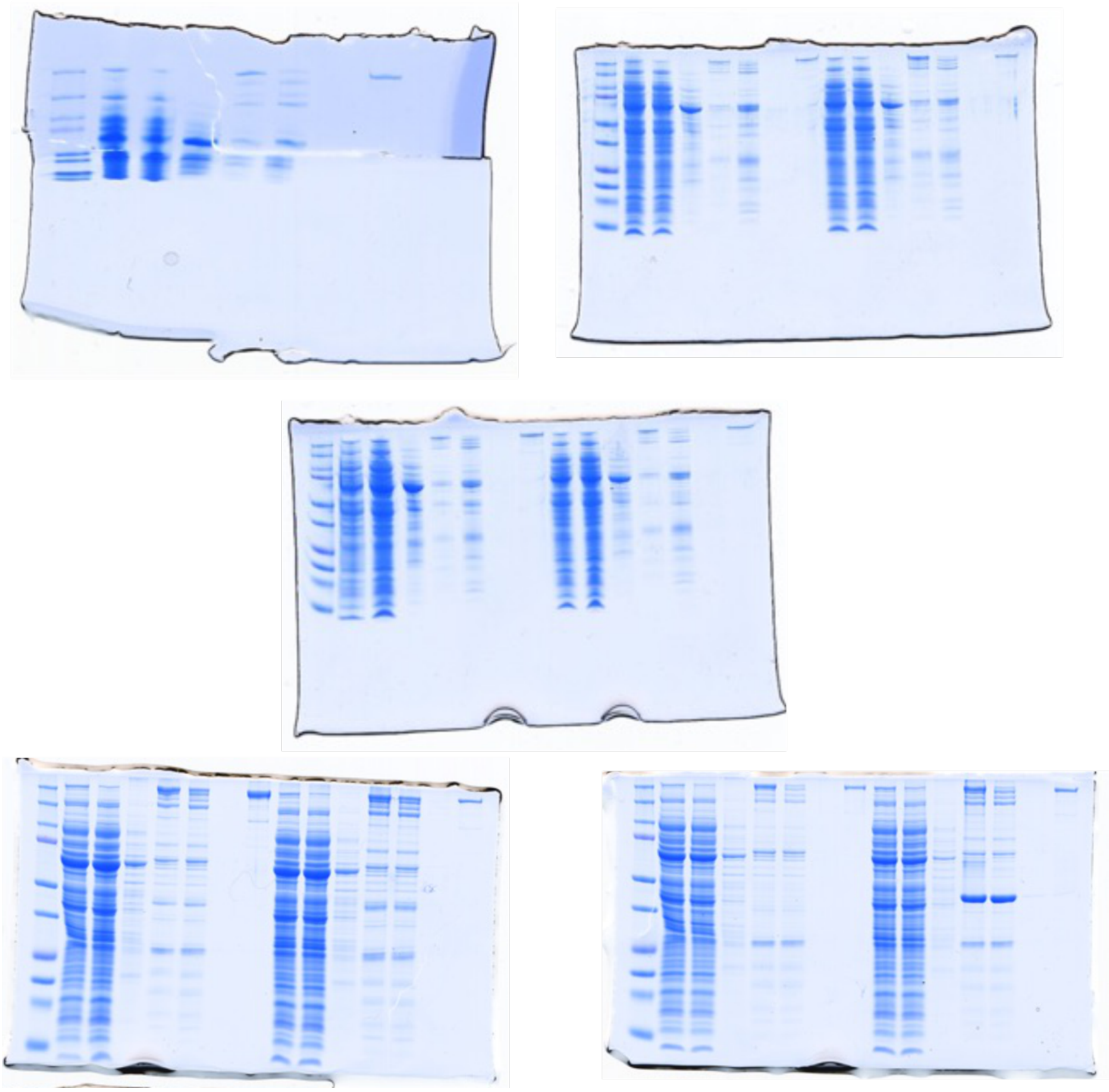

Supplementary Figure 33: Uncropped SDS-Page gels from supplementary figure 23.

Supplementary Methods:

Synthesis of decanoyl-CoA ester

Decanoyl-CoA ester was prepared as done by *Gusenda et al*.[34]

Shortly, the synthesis was performed under argon-atmosphere. 202 mg decanoic acid (1.17 mmol, 6 eq) was dissolved in 6 mL abs. THF and cooled to 0 °C. 90 µL ethyl chloroformate (1.16 mmol, 5.9 eq) and 160 µL triethylamine (TEA) (1.15 mmol, 5.9 eq) were added to the solution and stirred for 45 min at 0 °C. The reaction mixture was transferred to a 2 mL Eppendorf-tube and centrifuged for 5 min at 20000 x g. The supernatant was transferred to a solution of 150 mg CoA (0.195 mmol, 1 eq) in 6 mL 0.1 M $NaHCO_3$ and stirred for 1 h at RT. The solution was divided into two parts and each fraction was poured into 25 mL -20 °C cold acetone. The mixtures were centrifuged for 5 min at 10000 x g and the supernatant was discarded. The crude product was dried under reduced pressure and stored at -20 °C until further purification.

Isolation of the product was performed using a reverse phase (RP) liquid chromatography (LC) column with C18-RP silica gel (part. size 48-65) from Thermo Scientific and MeOH/H2O 1:1 as eluent. The collected fractions were combined and the organic solvent removed under reduced pressure.

Yield: 45% as white solid.

Synthesis of C10-ACP

C10-ACP was biosynthesized as done by *Gusenda et al*.[34]

Shortly, SFP, C10-CoA and apo-ACP were mixed in 5 mL buffer (50 mM Tris, 10 mM $MgCl_2$, pH 7.5) to final concentrations of: SFP (15 µM, 0.05 eq), C10-CoA (1500 µM, 5 eq) and apo-ACP (300 µM, 1 eq). The reaction mixture was incubated for 10 min at 37 °C and 300 x g and the reaction progress was checked via HPLC analysis. Therefore, 5 µL sample were mixed with 25 µL 0.1% TFA solution and injected to the Discovery® BIO Wide Pore C5 LC-column from Supelco and eluted with a H_2O + 0.1%TFA/ACN + 0.1%TFA gradient (20-98 % in 16 min). The reaction mixture was poured onto a Strep Tactin XT column, washed with 3 CV Strep-Wash buffer and eluted with 2.5 CV Strep-Elution buffer. The elution fraction was rebuffered to ACP-buffer (50 mM potassium phosphate, 200 mM KCl, 1 mM EDTA, 10% (v/v) glycerol, pH 7.0) and concentrated using Amicon® Ultra Centrifugal filters from Merck with a 10 kDa cut-off.

Lists:

Supplementary Table 2: Table of TR domains screened for this work. Given is the internal number, the enzyme class, the molecular weight, the type of reduction and their native organism.

Short form	Plasmid number	Enzyme class	Molecular weight in kDa	Type [e$^-$]	Organism
#1	pAR388	CAR	50.9	2	*Mycobacterium marinum*
#2	pAR389	CAR	51.0	2	*Mycobacterium phlei*
#3	pAR390	CAR	50.5	2	*Mycobacterium smegmatis*
#4	pAR391	CAR	50.6	2	*Nocardia iowensis*
#5	pAR392	CAR	50.7	2	*Nocardia otitidiscaviarum*
#6	pAR393	CAR	50.4	2	*Tsukamurella paurometabola*
#7	pMMH12	CAR	46.6	2/4	*Neurospora crassa*
#8	pAR394	PKS/NRPS	44.8	4	*Stigmatella aurantiaca*
#9	pAR395	PKS/NRPS	51.6	4	*Mycobacterium tuberculosis*
#10	pAR396	PKS/NRPS	52.8	4	*Mycobacterium smegmatis*
#11	pMMH05	NRPS	48.8	2/4	*Methanobrevibacter ruminantium*

Supplementary Table3: Cloning strategies and primers (purchased from Sigma Aldrich) for plasmids created in this study. Plasmids were constructed using In-Fusion Cloning (Takara). The DNA encoding for the TR domains was amplified from their genomic DNA and introduced into a pET-22b (+) vector. Other plasmids were derived from plasmids prepared by us and collaboration partners in earlier projects.

**Plasmid**	**Cloning Method**	**Fragments**	**Primer Name**	**Primer Sequence 5‘-3‘/Restriction Enzymes**	**Template**
pAR388 (TR^{CAR}_Mmar)	InFusion	pAR3XX_V	PrAR646	GGAGGTAAGCTTCATCATCACCACCACCACC	pAR18
			PrAR647	TGCGGCCGCCATATGTATATCTCCTTCTTAAAG	
		pAR388_I	PrAR648	CATATGGCGGCCGCAGCTGCTAGAAAGCCAGGTTC	Mmar_CAR_SFP(AKBoles)
			PrAR649	GATGAAGCTTACCTCCCAACAAACCCAACAATCTCAAGTC	
pAR389 (TR^{CAR}_Mphi)	InFusion	pAR3XX_V	PrAR646	GGAGGTAAGCTTCATCATCACCACCACCACC	pAR18
			PrAR647	TGCGGCCGCCATATGTATATCTCCTTCTTAAAG	
		pAR389_I	PrAr650	CATATGGCGGCCGCAACCGCGCGCCGACCAG	Genomic DNA DSM: 43239
			PrAR651	GATGAAGCTTACCTCCGAGCAGCCCGAGCAGC	
pAR390 (TR^{CAR}_Msme)	InFusion	pAR3XX_V	PrAR646	GGAGGTAAGCTTCATCATCACCACCACCACC	pAR18
			PrAR647	TGCGGCCGCCATATGTATATCTCCTTCTTAAAG	
		pAR390_I	PrAR652	CATATGGCGGCCGCAGCGCAGCGCACCGCG	Genomic DNA DSM: 43756
			PrAR653	GATGAAGCTTACCTCCGATCAGACCGAACTCACGC	
pAR391 (TR^{CAR}_Niow)	InFusion	pAR3XX_V	PrAR646	GGAGGTAAGCTTCATCATCACCACCACCACC	pAR18
			PrAR647	TGCGGCCGCCATATGTATATCTCCTTCTTAAAG	
		pAR391_I	PrAR654	CATATGGCGGCCGCAGCGGAACGCAACTCGGG	Genomic DNA DSM: 45107
			PrAR655	GATGAAGCTTACCTCCGAGCAGCTGAAGCAGTTCC	
pAR392 (TR^{CAR}_Noti)	InFusion	pAR3XX_V	PrAR646	GGAGGTAAGCTTCATCATCACCACCACCACC	pAR18
			PrAR647	TGCGGCCGCCATATGTATATCTCCTTCTTAAAG	
		pAR392_I	PrAR656	CATATGGCGGCCGCAAGCGAACGCGATTCCGGC	Genomic DNA DSM: 43242
			PrAR657	GATGAAGCTTACCTCCGCCCTGAATCAACCCCAG	

pAR393 (TR^{CAR}_Tpau)	InFusion	pAR3XX_V	PrAR646	GGAGGTAAGCTTCATCATCACCACCACCACC	pAR18
			PrAR647	TGCGGCCGCCATATGTATATCTCCTTCTTAAAG	
		pAR393_I	PrAR658	CATATGGCGGCCGCAAAGGCTCGATCGGGTGGC	Genomic DNA DSM: 20162
			PrAR659	GATGAAGCTTACCTCCGTCGCGCGCACCGGG	
pAR394 (TR$^{PKS/NRPS}$_Saur)	InFusion	pAR3XX_V	PrAR646	GGAGGTAAGCTTCATCATCACCACCACCACC	pAR18
			PrAR647	TGCGGCCGCCATATGTATATCTCCTTCTTAAAG	
		pAR394_I	PrAR660	CATATGGCGGCCGCAGCCGCGAAGACAGGCTC	Genomic DNA DSM: 17044
			PrAR661	GATGAAGCTTACCTCCCCTCACCTCGGGTGCCTTG	
pAR395 (TR$^{PKS/NRPS}$_Mtub)	InFusion	pAR3XX_V	PrAR646	GGAGGTAAGCTTCATCATCACCACCACCACC	pAR18
			PrAR647	TGCGGCCGCCATATGTATATCTCCTTCTTAAAG	
		pAR395_I	PrAR662	CATATGGCGGCCGCACGAGATGCCCGACCGAC	Genomic DNA
			PrAR663	GATGAAGCTTACCTCCCAGCAGTCCGAGCAGTTGTAG	
pAR396 (TR$^{PKS/NRPS}$_Msme)	InFusion	pAR3XX_V	PrAR646	GGAGGTAAGCTTCATCATCACCACCACCACC	pAR18
			PrAR647	TGCGGCCGCCATATGTATATCTCCTTCTTAAAG	
		pAR396_I	PrAR664	CATATGGCGGCCGCACACGACGCCGGTGAGCG	Genomic DNA DSM: 43756
			PrAR665	GATGAAGCTTACCTCCCAGCAGCCCGAGCAGCTG	
pAR401 (mFAS/TR^{CAR}_Msme)	restriction enzyme	pAR401_V		Not1-HF, HindIII-HF	pAR387
		pAR401_I		Not1-HF, HindIII-HF	pAR390
pAR435 (mFAS/'TesA)	InFusion	pAR435_V	PrAR202	CTCGAGCATCATCACCACCAC	pAR18
			PrAR706	TTGGTTCTGCTTCAGAGACG	
		pAR435_I	PrAR729	CTGAAGCAGAACCAAGCCGCCGCAGCG	Genomic DNA *E. Coli*, selfmade
			PrAR730	GTGATGATGCTCGAGTGAGTCATGATTTACTAAAGGCTG	
pAR436 (mFAS/'TesA_L109P)	InFusion	pAR436_1	PrAR729	CTGAAGCAGAACCAAGCCGCCGCAGCG	pAR435
			PrAR728	GTTTGCAGGCGGACGTATTTGCATTAACAATGGTTC	

		pAR436_2	PrAR727	GCAAATACGTCCGCCTGCAAACTATGGTCG	
			PrAR730	GTGATGATGCTCGAGTGAGTCATGATTTACTAAAGGCTG	
pAR440 (mFAS^{G113M})	InFusion	pAR440_1	PrAR321	TGGGTGGGTGTGAGTATKTCAGAGGCATCCGAGGCC	AR18
			PrAR27	GGTTAGCTCCTTCGGTCCTC	
		pAR440_2	PrAR26	GAGGACCGAAGGAGCTAACC	AR18
			PrAR322	ACTCACACCCACCCAGACG	
pAR442 (mFAS^{G113F})	InFusion	pAR442_1	PrAR323	TGGGTGGGTGTGAGTTWTTCAGAGGCATCCGAGGCC	AR18
			PrAR27	GGTTAGCTCCTTCGGTCCTC	
		pAR442_2	PrAR26	GAGGACCGAAGGAGCTAACC	AR18
			PrAR322	ACTCACACCCACCCAGACG	
pAR445 (mFAS^{G113W})	InFusion	pAR445_1	PrAR324	TGGGTGGGTGTGAGTTKGTCAGAGGCATCCGAGGCC	AR18
			PrAR27	GGTTAGCTCCTTCGGTCCTC	
		pAR445_2	PrAR26	GAGGACCGAAGGAGCTAACC	AR18
			PrAR322	ACTCACACCCACCCAGACG	
pDL007 (mFAS^{G113M}/'TesA)	InFusion	pDL007_1	PrAR321	TGGGTGGGTGTGAGTATKTCAGAGGCATCCGAGGCC	pAR435
			PrAR27	GGTTAGCTCCTTCGGTCCTC	
		pDL007_2	PrAR26	GAGGACCGAAGGAGCTAACC	pAR435
			PrAR322	ACTCACACCCACCCAGACG	
pDL008 (mFAS^{G113F}/'TesA)	InFusion	pDL008_1	PrAR323	TGGGTGGGTGTGAGTTWTTCAGAGGCATCCGAGGCC	pAR435
			PrAR27	GGTTAGCTCCTTCGGTCCTC	
		pDL008_2	PrAR26	GAGGACCGAAGGAGCTAACC	pAR435
			PrAR322	ACTCACACCCACCCAGACG	
pDL010 (mFAS^{G113M}/'TesA_L109P)	InFusion	pDL010_1	PrAR321	TGGGTGGGTGTGAGTATKTCAGAGGCATCCGAGGCC	pAR436
			PrAR27	GGTTAGCTCCTTCGGTCCTC	
		pDL010_2	PrAR26	GAGGACCGAAGGAGCTAACC	pAR436
			PrAR322	ACTCACACCCACCCAGACG	
pDL011 (mFAS^{G113F}/'TesA_L109P)	InFusion	pDL011_1	PrAR323	TGGGTGGGTGTGAGTTWTTCAGAGGCATCCGAGGCC	pAR436
			PrAR27	GGTTAGCTCCTTCGGTCCTC	
		pDL011_2	PrAR26	GAGGACCGAAGGAGCTAACC	pAR436
			PrAR322	ACTCACACCCACCCAGACG	
pDL013	InFusion		PrDL007	GCGACTCCTGCATTAGGAAG	pAR435

		pDL013_1	PrDL014	GACCTCTTCCATAAAAAAGGGCAG	
		pDL013_1	PrDL013	TTTATGGAAGAGGTCGGCAAGAAGCCACAATGGATGCAGGATG	pAR435
			PrDL008	CTTCCTAATGCAGGAGTCGC	
pDL017 (mFAS/'TesA_4x)	InFusion	pDL017_1	PrDL15	CTGCTGCCCTTTTTTcTGGAtGAGGTCggCaagAAGC	pDL013
			PrDL10	AGTCAATTCAGGGTGGTGAATG	
		pDL017_1	PrDL09	CACCCTGAATTGACTCTCTTC	pDL013
			PrDL16	AAAAAAGGGCAGCAGCG	
pDL018 (mFAS^{G113M}/'TesA_4x)	InFusion	pDL018_1	PrAR321	TGGGTGGGTGTGAGTATKTCAGAGGCATCCGAGGCC	pDL017
			PrAR27	GGTTAGCTCCTTCGGTCCTC	
		pDL018_2	PrAR26	GAGGACCGAAGGAGCTAACC	pDL017
			PrAR322	ACTCACACCCACCCAGACG	
pDL019 (mFAS^{G113F}/'TesA_4x)	InFusion	pDL019_1	PrAR323	TGGGTGGGTGTGAGTTWTTCAGAGGCATCCGAGGCC	pDL017
			PrAR27	GGTTAGCTCCTTCGGTCCTC	
		pDL019_2	PrAR26	GAGGACCGAAGGAGCTAACC	pDL017
			PrAR322	ACTCACACCCACCCAGACG	
pDL021 (mFAS^{G113S}/'TesA)	InFusion	pDL021_1	PrDL19	TGGGTGGGTGTGAGTTCGTCAGAGGCATCCGAGGCC	pAR435
			PrAR27	GGTTAGCTCCTTCGGTCCTC	
		pDL021_2	PrAR26	GAGGACCGAAGGAGCTAACC	pAR435
			PrAR322	ACTCACACCCACCCAGACG	
pDL026 (mFAS^{G113W}/'TesA)	InFusion	pDL026_1	PrAR324	TGGGTGGGTGTGAGTTKGTCAGAGGCATCCGAGGCC	pAR435
			PrAR27	GGTTAGCTCCTTCGGTCCTC	
		pDL026_2	PrAR26	GAGGACCGAAGGAGCTAACC	pAR435
			PrAR322	ACTCACACCCACCCAGACG	
pDL027 (mFAS^{G113S})	InFusion	pDL027_1	PrDL19	TGGGTGGGTGTGAGTTCGTCAGAGGCATCCGAGGCC	pAR18
			PrAR27	GGTTAGCTCCTTCGGTCCTC	
		pDL027_2	PrAR26	GAGGACCGAAGGAGCTAACC	pAR18
			PrAR322	ACTCACACCCACCCAGACG	
pDL028 (mFAS^{G113S}/'TesA_L109P)	InFusion	pDL028_1	PrDL19	TGGGTGGGTGTGAGTTCGTCAGAGGCATCCGAGGCC	pAR436
			PrAR27	GGTTAGCTCCTTCGGTCCTC	
		pDL028_2	PrAR26	GAGGACCGAAGGAGCTAACC	pAR436
			PrAR322	ACTCACACCCACCCAGACG	

pDL029 (mFAS^{G113S}/'TesA_4x)	InFusion	pDL029_1	PrDL19	TGGGTGGGTGTGAGTTCGTCAGAGGCATCCGAGGCC	pDL017
			PrAR27	GGTTAGCTCCTTCGGTCCTC	
		pDL029_2	PrAR26	GAGGACCGAAGGAGCTAACC	pDL017
			PrAR322	ACTCACACCCACCCAGACG	
pDL030 (mFAS^{G113W})	InFusion	pDL030_1	PrAR324	TGGGTGGGTGTGAGTTKGTCAGAGGCATCCGAGGCC	pAR18
			PrAR27	GGTTAGCTCCTTCGGTCCTC	
		pDL030_2	PrAR26	GAGGACCGAAGGAGCTAACC	pAR18
			PrAR322	ACTCACACCCACCCAGACG	
pDL031 (mFAS^{G113W}/'TesA_L109P)	InFusion	pDL031_1	PrAR324	TGGGTGGGTGTGAGTTKGTCAGAGGCATCCGAGGCC	s
			PrAR27	GGTTAGCTCCTTCGGTCCTC	
		pDL031_2	PrAR26	GAGGACCGAAGGAGCTAACC	pAR436
			PrAR322	ACTCACACCCACCCAGACG	
pDL032 (mFAS^{G113W}/'TesA_4x)	InFusion	pDL031_1	PrAR324	TGGGTGGGTGTGAGTTKGTCAGAGGCATCCGAGGCC	pDL017
			PrAR27	GGTTAGCTCCTTCGGTCCTC	
		pDL031_2	PrAR26	GAGGACCGAAGGAGCTAACC	pDL017
			PrAR322	ACTCACACCCACCCAGACG	
pMMH01 (mFAS/TR^{CAR}_Mphi)	InFusion	pMMH01_1	PrMMH01	CCGAAAGGAAGCTGAGTTGGC	pAR18
			PrMMH02	CGCGGTTGCGGCCGCCGAGTCAGTCTTGGAGGACATTTC	
		pMMH01_2	PrMMH03	GCGGCCGCAACCGCG	pAR389
			PrMMH04	CTCAGCTTCCTTTCGGGCTTTGTTAG	
pMMH02 (mFAS/TR^{CAR}_Noti)	InFusion	pMMH02_1	PrMMH01	CCGAAAGGAAGCTGAGTTGGC	pAR18
			PrMMH05	TTCGCTTGCGGCCGCCGAGTCAGTCTTGGAGGACATTTC	
		pMMH02_2	PrMMH06	GCGGCCGCAAGCGAAC	pAR392
			PrMMH04	CTCAGCTTCCTTTCGGGCTTTGTTAG	
pMMH05 (TR$^{PKS/NRPS}$_Mrum)	InFusion	pMMH05_V	PrMMH13	TTTGATTATTTTGAAAAGCTTCATCATCACCACCACC	pAR390
			PrMMH14	CTCTTCGCCAATGTCGGCCGCCATATGTATATCTCCTTC	
		pMMH05_I	PrMMH11	GACATTGGCGAAGAGGACTTGG	Genomic DNA DSM: 1093

			PrMMH12	TTCAAAATAATCAAACTTATTCAAATAATCAAACAATCG	
pMM07 (mFAS^{G113W}TR^{CAR}_Mphi)	InFusion	pMMH07_1	PrAR324	TGGGTGGGTGTGAGTTKGTCAGAGGCATCCGAGGCC	pAR420
			PrMMH04	CTCAGCTTCCTTTCGGGCTTTGTTAG	
		pMMH07_2	PrAR322	ACTCACACCCACCCAGACG	pAR420
			PrMMH01	CCGAAAGGAAGCTGAGTTGGC	
pMMH08 (mFAS^{G113W}TR^{CAR}_Noti)	InFusion	pMMH08_1	PrAR324	TGGGTGGGTGTGAGTTKGTCAGAGGCATCCGAGGCC	pAR422
			PrMMH04	CTCAGCTTCCTTTCGGGCTTTGTTAG	
		pMMH08_2	PrAR322	ACTCACACCCACCCAGACG	pAR422
			PrMMH01	CCGAAAGGAAGCTGAGTTGGC	
pMMH10 (mFAS^{G113W}TR^{CAR}_Msme)	InFusion	pMMH10_1	PrAR324	TGGGTGGGTGTGAGTTKGTCAGAGGCATCCGAGGCC	pAR401
			PrMMH04	CTCAGCTTCCTTTCGGGCTTTGTTAG	
		pMMH10_2	PrAR322	ACTCACACCCACCCAGACG	pAR401
			PrMMH01	CCGAAAGGAAGCTGAGTTGGC	
pMMH12 (TR^{CAR}_Ncra)	InFusion	pMMH12_V	PrMMH31	AAGCTTCATCATCACCACCACC	pAR390
			PrMMH32	GGCCGCCATATGTATATCTCCTTC	
		pMMH12_I	PrMMH20	ATACATATGGCGGCCCAGGGCACCCTGGACAATG	NcCAR_R_domain_Nterm_10xHisTag (AK Winkler)
			PrMMH21	GTGATGATGAAGCTTAAAGTTCCATTGGCGACACCAG	
pHp-32	Gibson Assembly	P5/P2	P5	TTTATGATGAGTAAAATGATGTTTTTATTGTGTGAATAGGTG	pTaFAR1 (Ref. 4)
			P2	TTTATGATGAGTAAAATGATGTTTTTATTGTGTGAATAGGTG	pTaFAR1 (Ref. 4)
		P565/P530	P565	GCTCTTTAGTACAAAGATAATATAGAAACAAAATGTCTGCCGGCGCTGGCTC	pmFAS^{G113M}-Op^{OPT1}
			P530	CGATGGGGCAGGGTCCGATG	pmFAS^{G113M}-Op^{OPT1}
		P531/P566	P531	CAGTGGAACCCATCGGACCCTGCCCCATCGTCGTTGGGCGCCTTGGACTTG	pmFAS^{G113M}-Op^{OPT1}
			P566	CAATAAAAACATCATTTTACTCATCATAAATTACGAGTCGTGGTTCACCAGTG	pmFAS^{G113M}-Op^{OPT1}

pHp-33	Gibson Assembly	P5/P537	P5	TTTATGATGAGTAAAATGATGTTTTTATTGTGTGAATAGGTG	pHp-32
			P537	GACAGGGCCTCCGAGGCCTCCGAgaaCGACACGCCCACCCACACGCCGGTG	pHp-32
		P569/P530	P569	GCGTGTGGGTGGGCGTGTCGttcTCGGAGGCCTCGGAGGCCC	pHp-32
			P530	CGATGGGGCAGGGTCCGATG	pHp-32
		P531/P566	P531	CAGTGGAACCCATCGGACCCTGCCCCATCGTCGTTGGGCGCCTTGGACTTG	pHp-32
			P566	CAATAAAAACATCATTTTACTCATCATAAATTACGAGTCGTGGTTCACCAGTG	pHp-32
pOpMCFA-01	Gibson Assembly	P5/079	P5	TTTATGATGAGTAAAATGATGTTTTTATTGTGTGAATAGGTG	pHp-32
			079	CGACACGCCCACCCACACGC	pHp-32
		081/P530	081	GCGTGTGGGTGGGCGTGTCGtcgTCGGAGGCCTCGGAGGC	pHp-32
			P530	CGATGGGGCAGGGTCCGATG	pHp-32
		P531/P566	P531	CAGTGGAACCCATCGGACCCTGCCCCATCGTCGTTGGGCGCCTTGGACTTG	pHp-32
			P566	CAATAAAAACATCATTTTACTCATCATAAATTACGAGTCGTGGTTCACCAGTG	pHp-32

Supplementary Table 3: Cloning strategies and primers for expression cassettes created in this study. Plasmids were constructed using either In-Fusion Cloning (Takara), Gibson Assembly, overlap extension polymerase chain reaction (OE-PCR) and restriction enzyme cloning and labeled accordingly in the tables.

**Expression cassette**	**Cloning Method**	**Fragments**	**Primer Name**	**Primer Sequence 5‘-3‘/Restriction Enzymes**	**Template**
dOp-5-up	SOE-PCR	NS18up	p286	GGCCGAGAAGTATAACGTCAGACC	Genome of *O. polymorpha*
			p648	AGTCACACACCATATCCGAGCAG	
		P_{GAP}-mFAS^{G113M}_TesA-up	p649	GGAAGCTCTGCTCGGATATGGTGTGTGACTCCAATTATCATTAATAATCACTCATGATCCCTGC	pHp-32
			p650	CGGTGTGAACCAGCAGGAAGC	
dOp-5-dn	SOE-PCR	mFAS^{G113M}_TesA-dn-T_{ADH21}	p651	GCAAGCTGCTGTGGAAGGACAAC	pHp-32
			p652	GGCCCCGTCATTGTTTGAAAAAACGCGAAACAGAATTATGTACTGATGCTGCTGGATAC	

		NS18dn	p653	TTTCGCGTTTTTTCAAACAATGACGGG	Genome of *O. polymorpha*
			p314	GCTGGATATGGCAGACAATAAATACTCAC	
dOpMCFA-07	SOE-PCR	NS2up	001	GAGAACGACTGACAACGTGCTAC	Genome of *O. polymorpha*
			002	AATAATTTATGGCTTCTGCGGAGAGAGTGCTGTAGTTGTGATTTTAATGCACACTTGAG	
		$P_{ADH2-1-629}$	003	CACTCTCTCCGCAGAAGCCATAAATTATTTG	
			004	TTTAAATTGATTGATTGATTGATAGAAGGATGCCTAAG	
		SFP	054	TCCTTCTATCAATCAATCAATCAATTTAAAATGAAGATTTACGGAATTTATATGGACCG	SFP-Op^{opt1}
			055	TTACCTATTTACCTATTCAAAATACCACCTTTATAAAAGCTCTTCGTACGAGACCATTG	
		T_{FBA}	018	AGGTGGTATTTTGAATAGGTAAATAGGTAAATCAAC	Genome of *O. polymorpha*
			019	GCCTTCGGATATTTCAGTTATGTCGAC	
		NS2dw	020	CAACGTCGACATAACTGAAATATCCGAAGGCGTTGCTGGTGCCATCTTTTGGC	
			021	CACTTCAAATTTGCTACCACTGAACTGG	
Donor SFP	SOE-PCR	IX-2up	P373(IX-2upF)	GGGGAACTAAAGCACAAGGATTTGAAG	Genome of *S. cerevisiae*
			P431(GAL2p-IX-2upR)	CCCCTGGTCCGTAAACCTCCTTGGATTAGCCTTCCATCGGGATTACTAAGATCTCAAG	
		P_{GAL2}	P432(GAL2p-F)	CTAATCCAAGGAGGTTTACGGACCAG	
			P429(GAL2p-R)	TATGAAAGAATTATTTTTTTTATTATGTTAATCTTGTGTTTACTTAAC	
		SFP	P476(SFP-F)	ACATAATAAAAAAAATAATTCTTTCATAATGAAGATTTACGGAATTTATATGGACCGCC	pCDF-1b-SFP
			P477(SFP-R)	TAAAAATCATAAATCATAAGAAATTCGCTTATAAAAGCTCTTCGTACGAGACCATTGTG	
		T_{ADH1}	P104	GCATATCTACAATTGGGTGAAATGGGG	Genome of *S. cerevisiae*
			P105	GCGAATTTCTTATGATTTATGATTTTTATT	
		IX-2dw	P394(ADH1t-IX-2dwF)	CGCTCCCCATTTCACCCAATTGTAGATATGCCACTGCAGCTCTGCTTTGAAAAC	
			P382(IX-2dwR)	CCTGGTTTAGTTATCTGTAATGGTGGG	
Donor mFAS^{G113W}_MphiR	SOE-PCR	X-3up	P351(X-3upF	GCGCCTTTGATCCTTCCCC	Genome of *S. cerevisiae*
			P438(GAL2	TCCCCTGGTCCGTAAACCTCCTTGGATTAGGCCCGAGAAACGCAAATGC	

			p-X-3upR)		
		P_{GAL2}	P432(GAL2p-F)	CTAATCCAAGGAGGTTTACGGACCAG	
			P429(GAL2p-R)	TATGAAAGAATTATTTTTTTATTATGTTAATCTTGTGTTTACTTAAC	
		$mFAS^{G113W}$_MphiR up	P469(FAS1-F)	TAACATAATAAAAAAAATAATTCTTTCATAATGTCCGAGGAGGTGGTGATAGCC	pMMH07
			P470	CACTTTTCCGCTCACAATCAGATTG	
		$mFAS^{G113W}$_MphiR mid	P471	CACTGGCAATCTGATTGTGAGCG	
			P472	GGCCCATTTACCTGGAATGGC	
		$mFAS^{G113W}$_MphiR dw	P473	TCCCCTGATGCCATTCCAGG	
			P474	GTTGTGTGCTAGTGTCTCCCGTCTTCTGTTTAACCTCCGAGCAGCCCG	
		T_{PRM9}	P247(PRM9t-F)	ACAGAAGACGGGAGACACTAGC	Genome of *S. cerevisiae*
			P248(PRM9t-R)	ATTTTCAACATCGTATTTTCCGAAGCG	
		X-3dw	P404(PRM9t-X-3dwF)	AACGCTTCGGAAAATACGATGTTGAAAATCCCCGCGTAAATACGCGG	
			P361(X-3dwR)	GCAGTAAATGCCGCGCTTAC	
Donor $mFAS^{G113W}$_MsmeR	SOE-PCR	X-3up	P351(X-3upF	GCGCCTTTGATCCTTCCCC	Genome of *S. cerevisiae*
			P438(GAL2p-X-3upR)	TCCCCTGGTCCGTAAACCTCCTTGGATTAGGCCCGAGAAACGCAAATGC	
		P_{GAL2}	P432(GAL2p-F)	CTAATCCAAGGAGGTTTACGGACCAG	
			P429(GAL2p-R)	TATGAAAGAATTATTTTTTTATTATGTTAATCTTGTGTTTACTTAAC	
		$mFAS^{G113W}$_MsmeR up	P469(FAS1-F)	TAACATAATAAAAAAAATAATTCTTTCATAATGTCCGAGGAGGTGGTGATAGCC	pMMH10
			P470	CACTTTTCCGCTCACAATCAGATTG	
		$mFAS^{G113W}$_MsmeR mid	P471	CACTGGCAATCTGATTGTGAGCG	
			P472	GGCCCATTTACCTGGAATGGC	
			P473	TCCCCTGATGCCATTCCAGG	
			P475(FAS2-R)	GTTGTGTGCTAGTGTCTCCCGTCTTCTGTTTAACCTCCGATCAGACCGAACTC	
		T_{PRM9}	P247(PRM9t-F)	ACAGAAGACGGGAGACACTAGC	Genome of *S. cerevisiae*

		X-3dw	P404(PRM9t-X-3dwF)	AACGCTTCGGAAAATACGATGTTGAAAATCCCCGCGTAAATACGCGG	
			P361(X-3dwR)	GCAGTAAATGCCGCGCTTAC	
dOpMCFA-30(ScFAA1)/dOpMCFA-31(ScFAA4)	SOE-PCR	4NS5up	040	GAGTCTCCGAGCGCAGACAAC	Genome of *O. polymorpha*
			181	CTAATTATGGCAACGGTGTCTTTAGGGCTACTCAGATATCGTCTCCAACGACCAC	
		P_{TEF1}	012	TAGCCCTAAAGACACCGTTGCC	
			011	TTTGTACGAATGTACTAATTAAAAGATCAGAAAAAAATCTCATGTGG	
		ScFAA1/ ScFAA4	184/ 186	CTGATCTTTTAATTAGTACATTCGTACAAAATGGTTGCTCAATATACCGTTCCAG/ CTGATCTTTTAATTAGTACATTCGTACAAAATGACCGAACAATATTCCGTTGCAG	Genome of *S. cerevisiae*
			185/ 187	CTTATTCAGTTAGCTAGCTGAGCTCGAGATATTAAGACGAACTATAAACGGCGTCAAC/ CTTATTCAGTTAGCTAGCTGAGCTCGAGATATTAAGTGTTTTCTTTATAAACTCTTTCCACATCTGG	
		T_{YX212}	007	TATCTCGAGCTCAGCTAGCTAACTG	Genome of *O. polymorpha*
			022	CCATTCGCCATTCAGGCTGC	
		4NS5dw	044	CAACAGTTGCGCAGCCTGAATGGCGAATGGCTCCTCTGGGCTGCTGTGGT	
			045	GTTGATCAAACGGGCAATCCCC	
dOpMCFA-42	SOE-PCR	P_{FAA1}	265	GTACCGCTTAATGATCGAGGACTG	Genome of *O. polymorpha*
			267	AGTGATTTACGATCTGCAATAGTAGAAAATAGAGG	
		ScFAA1	263	TATTTTCTACTATTGCAGATCGTAAATCACTATGGTTGCTCAATATACCGTTCCAG	Genome of *S. cerevisiae*
			264	GATCTTATGTGATACATACTTTGAAATCATTAAGACGAACTATAAACGGCGTCAAC	
		T_{FAA1}	268	TGATTTCAAAGTATGTATCACATAAGATCTAATAATAATCTACAG	Genome of *O. polymorpha*
			269	GTGCCGAAGTCATTCAGACAGTC	
donor YlPOX2-mACOT5	SOE-PCR	3NS3up	204	CCGCCTATTGACAAAATTCCGC	Genome of *O. polymorpha*
			219	CTAATTATGGCAACGGTGTCTTTAGGGCTACAAGAACAAAAAAGCCAAATCCTTCAC	
		P_{TEF1}	012	TAGCCCTAAAGACACCGTTGCC	
			011	TTTGTACGAATGTACTAATTAAAAGATCAGAAAAAAATCTCATGTGG	
		YlPOX2	220	CTGATCTTTTAATTAGTACATTCGTACAAAATGAACCCAAACAACACCGG	pYlPOX2-Op^{OPT1}
			221	CTTTGCCTGAGACAACTTAACTGCTGCAGAACCACCACCCTCCTCGTCCAGCTCGCAGATG	

		mACOT5	222	GGTGGTGGTTCTGCAGCAGTTAAGTTGTCTCAGGCAAAGGTGCCAACCGTGTCGCTGGAG	pmACOT5-Op^{OPT1}
			223	CTCAAAAGCGGTATGTCCTTCCACGTCTCCTTACAGCTTGGCAGGCGATGG	
		T_{AOX}	205	GGAGACGTGGAAGGACATACCG	Genome of *O. polymorpha*
			206	CACTCGCTGGCCGGAACGCTCAAGTCGCTTCGCTGTGATGAAGCATACAACCTG	
		3NS3dw	207	GAAGCGACTTGAGCGTTCCG	
			208	GGGGTTCTTGTGTCAAGTGTATTGTAC	
donor YlPOX2-hACOT4 AtACX2-mACOT5 AtACX2-hACOT4 OpPOX1-mACOT5 (Based on donor YlPOX2-mACOT5, different fragments labeled with the same color are combined)		AtACX2	226	CTGATCTTTTAATTAGTACATTCGTACAAAATGGAGTCGAGAAGAGAGAAGAACC	pAtACX2-Op^{OPT1}
			227	CTTTGCCTGAGACAACTTAACTGCTGCAGAACCACCACCGAAGCCCACCACTTGGGTGTAC	
		hACOT4	224	GGTGGTGGTTCTGCAGCAGTTAAGTTGTCTCAGGCAAAGTCGGCCACCCTGATCCTGGAG	phACOT4-Op^{OPT1}
			225	CTCAAAAGCGGTATGTCCTTCCACGTCTCCTTACAGCTTTGGCACGGCGG	
		OpPOX1	228	CTGATCTTTTAATTAGTACATTCGTACAAAATGGACTCGACTATGCCAGAAGTG	Genome of *O. polymorpha*
			229	CTTTGCCTGAGACAACTTAACTGCTGCAGAACCACCACCTAATTTCCGCAAAGTCTGGCTCGTC	

Supplementary Table 4: Table of strains and their genotypes used in this study.

Strain	Gene type	Resource
JQcr03L	*MATa*; *ura3*Δ; T_{AOX1}::(P_{GAP}-*hCAS9*-T_{AOX1}); *ku80*Δ; *NS5*::(P_{TKL1}-*ScSAE2*-T_{URA3}); *OpLEU2*	Ref. 4
JQcr03L(SFP)	*MATa*; *ura3*Δ; T_{AOX1}::(P_{GAP}-*hCAS9*-T_{AOX1}); *ku80*Δ; *NS5*::(P_{TKL1}-*ScSAE2*-T_{URA3}); *OpLEU2*; *NS2*::($P_{ADH2\text{-}1\text{-}629}$-*SFP*-$T_{FBA}$) **=JQcr03L+*NS2*::($P_{ADH2\text{-}1\text{-}629}$-*SFP*-$T_{FBA}$)**	this study
XMCFA20	*MATa*; *ura3*Δ; T_{AOX1}::(P_{GAP}-*hCAS9*-T_{AOX1}); *ku80*Δ; *NS5*::(P_{TKL1}-*ScSAE2*-T_{URA3}); *OpLEU2*; *NS2*::($P_{ADH2\text{-}1\text{-}629}$-*SFP*-$T_{FBA}$); P_{GAP}-*mFAS*^{G113S}-*TesA*-T_{ADH} **=JQcr03L(SFP)+P_{GAP}-*mFAS*^{G113S}-*TesA*-T_{ADH}**	this study
XMCFA05	*MATa*; *ura3*Δ; T_{AOX1}::(P_{GAP}-*hCAS9*-T_{AOX1}); *ku80*Δ; *NS5*::(P_{TKL1}-*ScSAE2*-T_{URA3}); *OpLEU2*; *NS2*::($P_{ADH2\text{-}1\text{-}629}$-*SFP*-$T_{FBA}$); P_{GAP}-*mFAS*^{G113M}-*TesA*-T_{ADH} **=JQcr03L(SFP)+P_{GAP}-*mFAS*^{G113M}-*TesA*-T_{ADH}**	this study
XMCFA06	*MATa*; *ura3*Δ; T_{AOX1}::(P_{GAP}-*hCAS9*-T_{AOX1}); *ku80*Δ; *NS5*::(P_{TKL1}-*ScSAE2*-T_{URA3}); *OpLEU2*; *NS2*::($P_{ADH2\text{-}1\text{-}629}$-*SFP*-$T_{FBA}$); P_{GAP}-*mFAS*^{G113F}-*TesA*-T_{ADH} **=JQcr03L(SFP)+P_{GAP}-*mFAS*^{G113F}-*TesA*-T_{ADH}**	this study
XMCFA38	*MATa*; *ura3*Δ; T_{AOX1}::(P_{GAP}-*hCAS9*-T_{AOX1}); *ku80*Δ; *NS5*::(P_{TKL1}-*ScSAE2*-T_{URA3}); *OpLEU2*; *NS2*::($P_{ADH2\text{-}1\text{-}629}$-*SFP*-$T_{FBA}$); *NS18*::(P_{GAP}-*mFAS*^{G113M}-*TesA*-T_{ADH}) **=JQcr03l(SFP)+*NS18*::(P_{GAP}-*mFAS*^{G113M}-*TesA*-T_{ADH})**	this study
XMCFA03	*MATa*; *ura3*Δ; T_{AOX1}::(P_{GAP}-*hCAS9*-T_{AOX1}); *ku80*Δ; *NS5*::(P_{TKL1}-*ScSAE2*-T_{URA3}); *OpLEU2*; *NS2*::($P_{ADH2\text{-}1\text{-}629}$-*SFP*-$T_{FBA}$); *NS18*::(P_{GAP}-*mFAS*^{G113M}-*TesA*-T_{ADH}); *pox1*Δ **=XMCFA38+*pox1*Δ**	this study
XMCFA41	*MATa*; *ura3*Δ; T_{AOX1}::(P_{GAP}-*hCAS9*-T_{AOX1}); *ku80*Δ; *NS5*::(P_{TKL1}-*ScSAE2*-T_{URA3}); *OpLEU2*; *NS2*::($P_{ADH2\text{-}1\text{-}629}$-*SFP*-$T_{FBA}$); *NS18*::(P_{GAP}-*mFAS*^{G113M}-*TesA*-T_{ADH}); *faa1*Δ **=XMCFA38+*faa1*Δ**	this study
XMCFA27	*MATa*; *ura3*Δ; T_{AOX1}::(P_{GAP}-*hCAS9*-T_{AOX1}); *ku80*Δ; *NS5*::(P_{TKL1}-*ScSAE2*-T_{URA3}); *OpLEU2*; *NS2*::($P_{ADH2\text{-}1\text{-}629}$-*SFP*-$T_{FBA}$); *NS18*::(P_{GAP}-*mFAS*^{G113M}-*TesA*-T_{ADH}); *pox1*Δ; P_{GAP}-*mFAS*^{G113M}-*TesA*-T_{ADH} **=XMCFA03+P_{GAP}-*mFAS*^{G113M}-*TesA*-T_{ADH}**	this study
XMCFA28	J *MATa*; *ura3*Δ; T_{AOX1}::(P_{GAP}-*hCAS9*-T_{AOX1}); *ku80*Δ; *NS5*::(P_{TKL1}-*ScSAE2*-T_{URA3}); *OpLEU2*; *NS2*::($P_{ADH2\text{-}1\text{-}629}$-*SFP*-$T_{FBA}$); *NS18*::(P_{GAP}-*mFAS*^{G113M}-*TesA*-T_{ADH}); *pox1*Δ; P_{GAP}-*mFAS*^{G113S}-*TesA*-T_{ADH} **=XMCFA03+P_{GAP}-*mFAS*^{G113S}-*TesA*-T_{ADH}**	this study
XMCFA50	*MATa*; *ura3*Δ; T_{AOX1}::(P_{GAP}-*hCAS9*-T_{AOX1}); *ku80*Δ; *NS5*::(P_{TKL1}-*ScSAE2*-	this study

	T_{URA3}); OpLEU2; NS2::($P_{ADH2-1-629}$-SFP-T_{FBA}); NS18::(P_{GAP}-mFAS^{G113M}-TesA-T_{ADH}); faa1Δ; 4NS5::(P_{TEF1}-ScFAA1-T_{YX212}) **=XMCFA41+4NS5::(P_{TEF1}-ScFAA1-T_{YX212})**	
XMCFA51	MATa; ura3Δ; T_{AOX1}::(P_{GAP}-hCAS9-T_{AOX1}); ku80Δ; NS5::(P_{TKL1}-ScSAE2-T_{URA3}); OpLEU2; NS2::($P_{ADH2-1-629}$-SFP-T_{FBA}); NS18::(P_{GAP}-mFAS^{G113M}-TesA-T_{ADH}); faa1Δ; 4NS5::(P_{TEF1}-ScFAA4-T_{YX212}) **=XMCFA41+4NS5::(P_{TEF1}-ScFAA4-T_{YX212})**	this study
XMCFA58	MATa; ura3Δ; T_{AOX1}::(P_{GAP}-hCAS9-T_{AOX1}); ku80Δ; NS5::(P_{TKL1}-ScSAE2-T_{URA3}); OpLEU2; NS2::($P_{ADH2-1-629}$-SFP-T_{FBA}); NS18::(P_{GAP}-mFAS^{G113M}-TesA-T_{ADH}); pox1Δ; 3NS3::(P_{TEF1}-YlPOX2-mACOT5-T_{AOX}) **=XMCFA03+3NS3::(P_{TEF1}-YlPOX2-mACOT5-T_{AOX})**	this study
XMCFA59	MATa; ura3Δ; T_{AOX1}::(P_{GAP}-hCAS9-T_{AOX1}); ku80Δ; NS5::(P_{TKL1}-ScSAE2-T_{URA3}); OpLEU2; NS2::($P_{ADH2-1-629}$-SFP-T_{FBA}); NS18::(P_{GAP}-mFAS^{G113M}-TesA-T_{ADH}); pox1Δ; 3NS3::(P_{TEF1}-YlPOX2-hACOT4-T_{AOX}) **=XMCFA03+3NS3::(P_{TEF1}-YlPOX2-hACOT4-T_{AOX})**	this study
XMCFA60	MATa; ura3Δ; T_{AOX1}::(P_{GAP}-hCAS9-T_{AOX1}); ku80Δ; NS5::(P_{TKL1}-ScSAE2-T_{URA3}); OpLEU2; NS2::($P_{ADH2-1-629}$-SFP-T_{FBA}); NS18::(P_{GAP}-mFAS^{G113M}-TesA-T_{ADH}); pox1Δ; 3NS3::(P_{TEF1}-AtACX2-mACOT5-T_{AOX}) **=XMCFA03+3NS3::(P_{TEF1}-AtACX2-mACOT5-T_{AOX})**	this study
XMCFA61	MATa; ura3Δ; T_{AOX1}::(P_{GAP}-hCAS9-T_{AOX1}); ku80Δ; NS5::(P_{TKL1}-ScSAE2-T_{URA3}); OpLEU2; NS2::($P_{ADH2-1-629}$-SFP-T_{FBA}); NS18::(P_{GAP}-mFAS^{G113M}-TesA-T_{ADH}); pox1Δ; 3NS3::(P_{TEF1}-AtACX2-hACOT4-T_{AOX}) **=XMCFA03+3NS3::(3NS3::(P_{TEF1}-AtACX2-hACOT4-T_{AOX})**	this study
XMCFA62	MATa; ura3Δ; T_{AOX1}::(P_{GAP}-hCAS9-T_{AOX1}); ku80Δ; NS5::(P_{TKL1}-ScSAE2-T_{URA3}); OpLEU2; NS2::($P_{ADH2-1-629}$-SFP-T_{FBA}); NS18::(P_{GAP}-mFAS^{G113M}-TesA-T_{ADH}); pox1Δ; 3NS3::(P_{TEF1}-OpPOX1-hACOT4-T_{AOX}) **=XMCFA03+3NS3::3NS3::(P_{TEF1}-OpPOX1-hACOT4-T_{AOX})**	this study
XMCFA65	MATa; ura3Δ; T_{AOX1}::(P_{GAP}-hCAS9-T_{AOX1}); ku80Δ; NS5::(P_{TKL1}-ScSAE2-T_{URA3}); OpLEU2; NS2::($P_{ADH2-1-629}$-SFP-T_{FBA}); NS18::(P_{GAP}-mFAS^{G113M}-TesA-T_{ADH}); pox1Δ; 3NS3::(P_{TEF1}-YlPOX2-hACOT4-T_{AOX}); FAA1::ScFAA1 **=XMCFA59+ FAA1::ScFAA1**	this study
XMCFA69	MATa; ura3Δ; T_{AOX1}::(P_{GAP}-hCAS9-T_{AOX1}); ku80Δ; NS5::(P_{TKL1}-ScSAE2-T_{URA3}); OpLEU2; NS2::($P_{ADH2-1-629}$-SFP-T_{FBA}); NS18::(P_{GAP}-mFAS^{G113M}-TesA-T_{ADH}); pox1Δ; 3NS3::(P_{TEF1}-YlPOX2-hACOT4-T_{AOX}); FAA1::ScFAA1; P_{GAP}-mFAS^{G113S}-TesA-T_{ADH} **=XMCFA65+ P_{GAP}-mFAS^{G113S}-TesA-T_{ADH}**	this study
GNFOH120	MATa; MAL2-8c; SUC2; hfd1Δ; gal80Δ; gal1Δ; gal7Δ; his3Δ::(HIS3-T_{ENO2})+(P_{ADH2}-PEX28-T_{HIS3}); ura3Δ::(P_{TPI1}-RtFAS1-T_{FBA1})+ (P_{TEF1}-RtFAS2-T_{CYC1})+amdSym; X1-5::P_{TEF1}-Cas9-T_{CYC1}; acc1::P_{TEF1}-ACC1; pyc1::P_{TEF1}-PYC1; XI-4::(P_{TPI1}-MPC1)+(P_{PGK1}-MPC3-T_{DIT1}); X-2::(P_{GAL1}-AnACLa-T_{CYC1})+(P_{GAL10}-AnACLb-T_{ADH1}); gal10Δ::(P_{TPI1}-RtCIT1-T_{FBA1})+(P_{TDH3}-IDP2-T_{CYC1})+(P_{TEF1}-YHM2-T_{GAL1}); pgi1Δ::(P_{COX9}-PGI1)+(P_{TDH3}-GND1-T_{CYC1})+(P_{tHXT7}-TKL1-T_{TDH2})+(P_{PGK1}-TAL1-T_{ADH1})+(P_{TEF1}-ZWF1); idh2Δ:: P_{GSY1}-IDH2;XII-4:: (P_{ADH6}-per1ADH5-T_{ADH1})+(P_{ADH6}-per2MaFAR1-T_{CYC1}); VII-2:: (T_{ADH1}-per1ADH5-$P_{GAL1,10}$-per2MaFAR1-T_{CYC1});VIII-2::(P_{BDH2}-per1ADH5-T_{ADH1})+ (P_{BDH2}-per2MaFAR1-T_{CYC1}); XI-2::(P_{HXT7}-PXA1-T_{ADH1})+(P_{HXT7}-PXA2-T_{FBA1}); XI-6::(T_{CPS1}-per2PYC1-$P_{GAL1,10}$-per2RtME-T_{PRM9}); XI-8::(T_{CPS1}-IDP3-$P_{GAL1,10}$-per2IDP2-T_{PRM9})	Ref. 5
GNFOH152	MATa; MAL2-8c; SUC2; hfd1Δ; gal80Δ; gal1Δ; gal7Δ; his3Δ::(HIS3-T_{ENO2})+(P_{ADH2}-PEX28-T_{HIS3}); ura3Δ::(P_{TPI1}-RtFAS1-T_{FBA1})+ (P_{TEF1}-RtFAS2-T_{CYC1})+amdSym; X1-5::P_{TEF1}-Cas9-T_{CYC1}; acc1::P_{TEF1}-ACC1; pyc1::P_{TEF1}-PYC1; XI-4::(P_{TPI1}-MPC1)+(P_{PGK1}-MPC3-T_{DIT1}); X-2::(P_{GAL1}-AnACLa-T_{CYC1})+(P_{GAL10}-AnACLb-T_{ADH1}); gal10Δ::(P_{TPI1}-RtCIT1-T_{FBA1})+(P_{TDH3}-IDP2-T_{CYC1})+(P_{TEF1}-YHM2-T_{GAL1}); pgi1Δ::(P_{COX9}-PGI1)+(P_{TDH3}-GND1-T_{CYC1})+(P_{tHXT7}-TKL1-T_{TDH2})+(P_{PGK1}-TAL1-T_{ADH1})+(P_{TEF1}-ZWF1); idh2Δ:: P_{GSY1}-IDH2;XII-4:: (P_{ADH6}-per1ADH5-T_{ADH1})+(P_{ADH6}-per2MaFAR1-T_{CYC1}); VII-2:: (T_{ADH1}-per1ADH5-$P_{GAL1,10}$-per2MaFAR1-T_{CYC1});VIII-2::(P_{BDH2}-per1ADH5-T_{ADH1})+ (P_{BDH2}-per2MaFAR1-T_{CYC1}); XI-2::(P_{HXT7}-PXA1-T_{ADH1})+(P_{HXT7}-PXA2-T_{FBA1}); XI-6::(T_{CPS1}-per2PYC1-$P_{GAL1,10}$-per2RtME-T_{PRM9}); XI-8::(T_{CPS1}-IDP3-$P_{GAL1,10}$-per2IDP2-T_{PRM9}) IX-2::(P_{GAL2}-SFP- T_{ADH1})**=GNFOH120+ IX-2:: P_{GAL2}-SFP- T_{ADH1}**	this study
GNFOH155	MATa; MAL2-8c; SUC2; hfd1Δ; gal80Δ; gal1Δ; gal7Δ; his3Δ::(HIS3-T_{ENO2})+(P_{ADH2}-PEX28-T_{HIS3}); ura3Δ::(P_{TPI1}-RtFAS1-T_{FBA1})+ (P_{TEF1}-RtFAS2-T_{CYC1})+amdSym; X1-5::P_{TEF1}-Cas9-T_{CYC1}; acc1::P_{TEF1}-ACC1; pyc1::P_{TEF1}-PYC1; XI-4::(P_{TPI1}-MPC1)+(P_{PGK1}-MPC3-T_{DIT1}); X-2::(P_{GAL1}-AnACLa-T_{CYC1})+(P_{GAL10}-AnACLb-T_{ADH1}); gal10Δ::(P_{TPI1}-RtCIT1-T_{FBA1})+(P_{TDH3}-IDP2-T_{CYC1})+(P_{TEF1}-YHM2-T_{GAL1}); pgi1Δ::(P_{COX9}-PGI1)+(P_{TDH3}-GND1-T_{CYC1})+(P_{tHXT7}-TKL1-T_{TDH2})+(P_{PGK1}-TAL1-T_{ADH1})+(P_{TEF1}-ZWF1); idh2Δ:: P_{GSY1}-IDH2;XII-4:: (P_{ADH6}-per1ADH5-T_{ADH1})+(P_{ADH6}-per2MaFAR1-T_{CYC1}); VII-2:: (T_{ADH1}-per1ADH5-$P_{GAL1,10}$-per2MaFAR1-T_{CYC1});VIII-2::(P_{BDH2}-per1ADH5-T_{ADH1})+ (P_{BDH2}-per2MaFAR1-T_{CYC1}); XI-2::(P_{HXT7}-PXA1-T_{ADH1})+(P_{HXT7}-PXA2-T_{FBA1}); XI-6::(T_{CPS1}-per2PYC1-$P_{GAL1,10}$-per2RtME-T_{PRM9}); XI-8::(T_{CPS1}-IDP3-$P_{GAL1,10}$-per2IDP2-	this study

	T_{PRM9}) *IX-2*::(P_{GAL2}-*SFP*- T_{ADH1}); *X-3*:: (P_{GAL2}-*mFAS*$^{G113W-}$*MphiR*-T_{PRM9}) **= GNFOH152+ X-3:: P_{GAL2}-mFAS^{G113W-}MphIR-T_{PRM9}**	
GNFOH156	*MATa*; *MAL2-8c*; *SUC2*; *hfd1*Δ; *gal80*Δ; *gal1*Δ; *gal7*Δ; *his3*Δ::(*HIS3*-T_{ENO2})+(P_{ADH2}-*PEX28*-T_{HIS3}); *ura3*Δ::(P_{TPI1}-*RtFAS1*-T_{FBA1})+ (P_{TEF1}-*RtFAS2*-T_{CYC1})+*amdSym*; *X1-5*::P_{TEF1}-*Cas9*-T_{CYC1}; *acc1*::P_{TEF1}-*ACC1*; *pyc1*::P_{TEF1}-*PYC1*; *XI-4*::(P_{TPI1}-*MPC1*)+(P_{PGK1}-*MPC3*-T_{DIT1}); *X-2*::(P_{GAL1}-*AnACLa*-T_{CYC1})+(P_{GAL10}-*AnACLb*-T_{ADH1}); *gal10*Δ::(P_{TPI1}-*RtCIT1*-T_{FBA1})+(P_{TDH3}-*IDP2*-T_{CYC1})+(P_{TEF1}-*YHM2*-T_{GAL1}); *pgi1*Δ::(P_{COX9}-*PGI1*)+(P_{TDH3}-*GND1*-T_{CYC1})+(P_{tHXT7}-*TKL1*-T_{TDH2})+(P_{PGK1}-*TAL1*-T_{ADH1})+(P_{TEF1}-*ZWF1*); *idh2*Δ:: P_{GSY1}-*IDH2*;*XII-4*:: (P_{ADH6}-*per1ADH5*-T_{ADH1})+(P_{ADH6}-*per2MaFAR1*-T_{CYC1}); *VII-2*:: (T_{ADH1}-*per1ADH5*-$P_{GAL1,10}$-*per2MaFAR1*-T_{CYC1});*VIII-2*::(P_{BDH2}-*per1ADH5*-T_{ADH1})+ (P_{BDH2}-*per2MaFAR1*-T_{CYC1}); *XI-2*::(P_{HXT7}-*PXA1*-T_{ADH1})+(P_{HXT7}-*PXA2*-T_{FBA1}); *XI-6*::(T_{CPS1}-*per2PYC1*-$P_{GAL1,10}$-*per2RtME*-T_{PRM9}); *XI-8*::(T_{CPS1}-*IDP3*-$P_{GAL1,10}$-*per2IDP2*-T_{PRM9}) *IX-2*::(P_{GAL2}-*SFP*- T_{ADH1}); *X-3*:: (P_{GAL2}-*mFAS*^{G113W}-*MsmeR*-T_{PRM9})**= GNFOH152+ X-3:: P_{GAL2}- mFAS^{G113W}-MsmeR -T_{PRM9}**	this study

Supplementary Table 5: Amino acid and DNA sequences of the constructs used in the FA chain length experiments. Mutated positions and the performed mutations are highlighted.

**Construct**	**Sequence form start to stop codon**
pAR18 (WT)<br>pDL027 (G113S)<br>pAR440 (G113M)<br>pAR442 (G113F)<br>pDL030 (G113W)<br>(mouseFAS, Strep-and His-tagged)<br>Position 113 highlighted	MSAWSHPQFEKGGGSGGGSGGSAWSHPQFEKGAGSEEVVIAGMSGKLPESENLQEFWANLIGGVDMVTDDDR<br>RWKAGLYGLPKRSGKLKDLSKFDASFFGVHPKQAHTMDPQLRLLLEVSYEAIVDGGINPASLRGTNTGVWVGV<br>S**G/S/M/F/W**SEASEALSRDPETLLGYSMVGCQRAMMANRLSFFFDFKGPSIALDTACSSSLLALQNAYQAIRS<br>GECPAALVGGINLLLKPNTSVQFMKLGMLSPDGTCRSFDDSGSGYCRSEAVVAVLLTKKSLARRVYATILNAGT<br>NTDGSKEQGVTFPSGEVQEQLICSLYQPAGLAPESLEYIEAHGTGTKVGDPQELNGITRSLCAFRQAPLLIGSTKS<br>NMGHPEPASGLAALTKVLLSLEHGVWAPNLHFHNPNPEIPALLDGRLQVVDRPLPVRGGNVGINSFGFGGSNV<br>HVILQPNTRQAPAPTAHAALPHLLHASGRTLEAVQDLLEQGRQHSQDLAFVSMLNDIAATPTAAMPFRGYTVL<br>GVEGRVQEVQQVSTNKRPLWFICSGMGTQWRGMGLSLMRLDSFRESILRSDEAVKPLGVKVSDLLLSTDERTF<br>DDIVHAFVSLTAIQIALIDLLTSVGLKPDGIIGHSLGEVACGYADGCLSQREAVLAAYWRGQCIKDAHLPPGSMA<br>AVGLSWEECKQRCPAGVVPACHNSEDTVTISGPQAAVNEFVEQLKQEGVFAKEVRTGGLAFHSYFMEGIAPTL<br>LQALKKVIREPRPRSARWLSTSIPEAQWQSSLARTSSAEYNVNNLVSPVLFQEALWHIPEHAVVLEIAPHALLQ<br>AVLKRGVKSSCTIIPLMKRDHKDNLEFFLTNLGKVHLTGINVNPNALFPPVEFPAPRGTPLISPHIKWDHSQTW<br>DVPVAEDFPNGSSSSSATVYSIDASPESPDHYLVDHCIDGRVIFPGTGYLCLVWKTLARSLGLSLEETPVVFENVS<br>FHQATILPKTGTVALEVRLLEASHAFEVSDTGNLIVSGKVYLWEDPNSKLFDHPEVPTPPESASVSRLTQGEVY<br>KELRLRGYDYGPQFQGICEATLEGEQGKLLWKDNWVTFMDTMLQVSILGSSQQSLQLPTRVTAIYIDPATHRQ<br>KVYRLKEDTQVADVTTSRCLGITVSGGIHISRLQTTATSRRQQEQLVPTLEKFVFTPHMEAECLSESTALQKELQ<br>LCKGLARALQTKATQQGLKAAMLGQEDPPQHGLPRLLAAACQLQLNGNLQLELGEALAQERLLLPEDPLISGL<br>LNSQALKACVDTALENLSTLKMKVAEVLAGEGHLYSRIPALLNTQPMLQLEYTATDRHPQALKDVQTKLQQH<br>DVAQGQWNPSDPAPSSLGALDLLVCNCALATLGDPALALDNMVAALKEGGFLLVHTVLKGHALGETLACLPSE<br>VQPAPSLLSQEEWESLFSRKALHLVGLKRSFYGTALFLCRRAIPQEKPIFLSVEDTSFQWVDSLKSTLATSSSQP<br>VWLTAMDCPTSGVVGLVNCLRKEPGGHRIRCILLSNLSNTSHAPKLDPGSPELQQVLKHDLVMNVYRDGAWG<br>AFRHFQLEQDKPKEQTAHAFVNVLTRGDLASIRWVSSPLKHTQPSSSGAQLCTVYYASLNFRDIMLATGKLSPD<br>AIPGKWASRDCMLGMEFSGRDRCGRRVMGLVPAEGLATSVLLSSDFLWDVPSSWTLEEAASVPVVYTTAYYSL<br>VVRGRIQRGETVLIHSGSGGVGQAAISIALSLGCRVFTTVGSAEKRAYLQARFPQLDDTSFANSRDTSFEQHVLL<br>HTGGKGVDLVLNSLAEEKLQASVRCLAQHGRFLEIGKFDLSNNHPLGMAIFLKNVTFHGILLDALFEEANDSW<br>REVAALLKAGIRDGVVKPLKCTVFPKAQVEDAFRYMAQGKHIGKVLVQVREEEPEAVLPGAQPTLISAISKTFC<br>PAHKSYIITGGLGGFGLELARWLVLRGAQRLVLTSRSGIRTGYQAKHIREWRRQGIQVLVSTSNVSSLEGARALI<br>AEATKLGPVGGVFNLAMVLRDAMLENQTPELFQDVNKPKYNGTLNLDRATREACPELDYFVAFSSVSCGRGN<br>AGQTNYGFANSTMERICEQRRHDGLPGLAVQWGAIGDVGIVLEAMGTNDTVIGGTLPQRISSCMEVLDLFLNQ<br>PHAVLSSFVLAEKKAVAHGDGDTQRDLVKAVAHILGIRDLAGINLDSTLADLGLDSLMGVEVRQILEREHDLVL<br>PMREVRQLTLRKLQEMSSKTDSATDTTAPKSRSDTSLKQNQLNLSTLLVNPEGPTLTQLNSVQSSERPLFLVHP<br>IEGSTTVFHSLAAKLSVPTYGLQCTQAAPLDSIPNLAAYYIDCIKQVQPEGPYRIAGYSFGACVAFEMCSQLQAQQ<br>GPAPTHNNLFLFDGSHTYVLAYTQSYRAKMTPGCEAEAEAEALCFFIKQFLDVEHSKVLEALLPLKSLEDRVAA<br>SVDLITKSHHSLDRRELSFAAVSFYHKLRAADQYKPKAKYHGNVTLLRAKTGGTYGEDLGADYNLSQVCDGKV<br>SVHIIEGDHRTLLEGSGLESIINIIHSSLAEPRVSVREGLEHHHHHHHH
pAR18 (WT)<br>pDL027 (G113S)<br>pAR440 (G113M)<br>pAR442 (G113F)<br>pDL030 (G113W)<br>(mouseFAS, Strep-and His-tagged)<br>Position 113 highlighted	ATGAGCGCTTGGAGCCATCCACAATTTGAGAAGGGTGGAGGTTCTGGCGGTGGATCGGGAGGTTCAGCGTGG<br>AGCCACCCGCAGTTCGAAAAAGGCGCCGGATCCGAGGAGGTGGTGATAGCCGGTATGTCGGGGAAGTTGCCCG<br>AGTCAGAGAACCTACAGGAGTTCTGGGCCAACCTCATTGGTGGTGTGGACATGGTCACAGATGATGACAGGA<br>GATGGAAGGCTGGGCTCTATGGATTACCCAAGCGGTCTGGAAAGCTGAAGGATCTCTCCAAGTTCGACGCCTC<br>CTTTTTTGGGGTCCACCCCAAGCAGGCACACACAATGGACCCCCAGCTTCGGCTGCTGTTGGAAGTCAGCTAT<br>GAAGCAATTGTGGATGGAGGTATCAACCCAGCCTCACTCCGAGGAACGAACACTGGCGTCTGGGTGGGTGTGA<br>GT**GGT/TCG/ATG/TTT/TGG**TCAGAGGCATCCGAGGCCCTTAGCAGAGATCCCGAGACGCTTCTGGGCTACA<br>GCATGGTGGGCTGCCAGCGTGCAATGATGGCCAACCGGCTCTCTTTCTTCTTCGACTTCAAAGGACCAAGCAT<br>TGCCCTGGACACAGCCTGCTCCTCCAGCTTGCTGGCACTACAGAATGCCTACCAGGCCATCCGTAGTGGGGAA<br>TGCCCCGCGGCCCTTGTGGGTGGGATCAACCTGCTCCTGAAGCCGAACACCTCTGTGCAGTTCATGAAGCTGG<br>GCATGCTCAGCCCGGACGGCACCTGCAGATCCTTTGATGATTCAGGGAGTGGATATTGTCGCTCTGAGGCTGT<br>TGTAGCAGTTCTGCTGACTAAGAAGTCCCTGGCTCGGCGGGTCTATGCCACGATTCTGAATGCCGGCACCAAT<br>ACAGATGGCAGCAAGGAGCAAGGTGTAACATTCCCCTCTGGAGAAGTCCAAGAACAACTCATCTGCTCTCTGT<br>ATCAGCCAGCTGGTCTGGCCCCGGAGTCGCTTGAGTATATTGAAGCCCATGGCACGGGCACCAAGGTGGGTGA<br>CCCCCAGGAACTGAATGGCATTACTCGGTCCCTGTGCGCCTTCCGCCAGGCCCCTCTGTTAATTGGCTCCACCA<br>AATCCAACATGGGACACCCTGAGCCTGCCTCTGGGCTTGCAGCCCTGACCAAGGTGCTGTTATCCCTGGAGCA<br>TGGGGTCTGGGCCCCTAACCTGCACTTCCACAACCCCAACCCTGAGATCCCAGCACTTCTTGATGGGCGGCTGC<br>AGGTGGTCGATAGGCCCCTGCCTGTTCGTGGTGGCAACGTGGGCATCAACTCATTTGGCTTCGGAGGCTCCAA<br>TGTTCATGTCATCCTCCAGCCCAACACACGGCAGGCCCCTGCGCCCACTGCACACGCTGCCCTTCCCCATTTGC<br>TGCACGCCAGTGGACGCACCTTAGAGGCAGTGCAGGACCTGCTGGAACAGGGCCGCCAGCACAGCCAGGACCT<br>GGCCTTTGTGAGCATGCTCAATGACATTGCGGCAACCCCTACAGCAGCCATGCCCTTCAGGGGTTACACTGTG<br>CTAGGTGTTGAGGGCCGTGTCCAAGAAGTGCAGCAAGTGTCCACCAACAAGCGCCCACTCTGGTTCATCTGCT<br>CAGGGATGGGCACGCAGTGGCGCGGGATGGGGCTGAGCCTCATGCGCCTGGACAGCTTCCGTGAGTCTATCCT<br>GCGCTCCGATGAGGCTGTGAAGCCGTTGGGAGTGAAAGTGTCAGATCTGCTGTTGAGCACAGATGAGCGCACC<br>TTTGATGACATCGTGCATGCCTTTGTGAGCCTCACTGCCATCCAGATTGCCCTCATCGACCTACTGACTTCTGT

Construct	Sequence form start to stop codon
	GGGACTGAAACCTGACGGCATCATTGGGCACTCCTTGGGAGAGGTTGCCTGTGGCTATGCAGATGGCTGTCTC<br>TCCCAGAGAGAGGCTGTGCTTGCAGCTTACTGGCGAGGCCAGTGCATCAAAGATGCCCACCTCCCGCCTGGAT<br>CCATGGCAGCTGTTGGTTTGTCCTGGGAGGAATGTAAACAGCGCTGCCCCGCTGGCGTGGTGCCTGCCTGCCA<br>CAACTCTGAGGACACCGTGACCATCTCTGGACCTCAGGCTGCAGTGAATGAATTTGTGGAGCAGCTAAAGCAA<br>GAAGGTGTGTTTGCCAAGGAGGTACGAACAGGAGGCCTGGCTTTCCACTCCTACTTCATGGAAGGAATTGCCC<br>CCACATTGCTGCAGGCTCTCAAGAAGGTGATCCGGGAACCACGGCCGCGCTCGGCTCGATGGCTCAGCACCTC<br>TATCCCTGAGGCCCAGTGGCAGAGCAGCCTGGCCCGCACATCTTCTGCCGAGTACAATGTCAACAACCTGGTG<br>AGCCCTGTGCTCTTCCAGGAAGCACTGTGGCACATCCCTGAGCATGCCGTGGTGCTGGAGATTGCGCCCCACG<br>CACTGTTGCAGGCTGTCCTGAAGCGAGGCGTGAAGTCCAGCTGCACCATCATTCCCTTGATGAAGAGGGATCA<br>TAAAGATAACTTGGAGTTCTTTCTCACCAACCTTGGCAAGGTGCACCTCACAGGCATCAATGTCAACCCTAAC<br>GCCTTGTTCCCACCTGTGGAGTTCCCGGCTCCCCGAGGGACTCCTCTCATCTCCCCTCACATCAAGTGGGACCA<br>CAGTCAGACTTGGGATGTCCCGGTTGCTGAGGACTTCCCAAACGGCTCCAGCTCCTCCTCTGCTACAGTCTAC<br>AGCATCGACGCCAGTCCTGAGTCGCCCGACCACTACCTGGTAGACCACTGCATTGACGGCCGGGTCATCTTCCC<br>TGGCACTGGCTACCTGTGCCTGGTGTGGAAGACACTGGCTCGCAGCCTGGGCTTGTCCCTAGAAGAGACCCCT<br>GTGGTATTTGAGAATGTGTCGTTTCATCAGGCCACTATACTACCCAAGACAGGAACCGTGGCGCTGGAGGTGA<br>GGCTGCTAGAGGCCTCCCATGCCTTTGAGGTGTCTGACACTGGCAATCTGATTGTGAGCGGAAAAGTGTACCT<br>GTGGGAAGACCCGAACTCCAAGTTATTCGACCACCCAGAAGTCCCAACACCCCCTGAGTCTGCATCGGTCTCC<br>CGCCTGACCCAGGGAGAAGTATACAAGGAGCTGCGGCTGCGTGGCTATGATTATGGCCCTCAGTTCCAGGGCA<br>TCTGTGAGGCCACCCTTGAAGGTGAACAAGGCAAGCTGCTCTGGAAAGATAACTGGGTGACCTTCATGGACAC<br>AATGCTGCAGGTATCCATTCTGGGTTCTAGCCAGCAGAGTCTACAGCTACCTACCCGTGTGACCGCCATCTAT<br>ATCGACCCTGCCACCCACCGTCAGAAGGTGTACAGGCTGAAGGAGGACACTCAAGTGGCTGATGTGACAACGA<br>GCCGCTGTCTGGGCATAACGGTCTCTGGTGGTATCCACATCTCAAGACTACAGACGACAGCAACCTCACGGCG<br>GCAGCAAGAACAGCTGGTCCCCACCTTGGAAAAGTTCGTTTTCACACCGCACATGGAGGCTGAGTGCCTGTCT<br>GAGAGCACTGCCCTGCAGAAGGAGCTGCAACTGTGCAAGGGTCTGGCACGGGCTCTGCAGACCAAGGCCACCC<br>AGCAAGGGCTGAAGGCGGCAATGCTTGGGCAAGAGGACCCTCCACAGCACGGGCTGCCTCGACTCCTGGCAGC<br>TGCTTGCCAGTTGCAGCTCAACGGGAACCTGCAGCTGGAGCTGGGAGAAGCGCTGGCTCAAGAGAGGCTCCTG<br>CTGCCAGAAGACCCTCTGATCAGTGGCCTCCTCAACTCCCAGGCCCTCAAGGCCTGCGTAGACACAGCCCTGGA<br>GAACTTGTCTACTCTCAAGATGAAGGTGGCAGAGGTGCTGGCTGGAGAAGGCCACTTGTATTCCCGAATCCCG<br>GCACTGCTCAACACCCAGCCCATGCTACAACTGGAATACACAGCCACCGACCGGCACCCCCAGGCCCTGAAGG<br>ATGTTCAGACCAAACTGCAGCAGCATGATGTGGCGCAGGGCCAGTGGAACCCTTCCGACCCTGCGCCCAGCAG<br>CCTGGGTGCCCTTGACCTTCTGGTGTGCAACTGTGCATTAGCCACCCTGGGGGATCCAGCCTTGGCCCTGGAC<br>AACATGGTAGCTGCCCTCAAGGAAGGTGGTTTCCTGCTAGTGCACACAGTGCTCAAAGGACATGCCCTTGGGG<br>AGACCCTGGCCTGCCTACCCTCTGAGGTGCAGCCTGCGCCCAGCCTCCTAAGCCAGGAGGAGTGGGAGAGCCT<br>GTTCTCGAGGAAGGCACTACACCTGGTGGGCCTTAAAAGGTCCTTCTACGGTACTGCGCTGTTCCTGTGCCGG<br>CGAGCCATCCCACAGGAGAAACCTATCTTCCTGTCTGTGGAGGATACCAGCTTCCAGTGGGTGGACTCTCTGA<br>AGAGCACTCTGGCCACGTCCTCCTCCCAGCCTGTGTGGCTAACGGCCATGGACTGCCCCACCTCGGGTGTGGTG<br>GGTTTGGTGAATTGTCTCCGAAAAGAGCCGGGTGGACACCGGATTCGGTGTATCCTGCTGTCCAACCTCAGCA<br>ACACATCTCACGCCCCCAAGTTGGACCCTGGCTCTCCAGAGCTACAGCAGGTGCTAAAGCATGACCTCGTGAT<br>GAACGTGTACCGGGACGGGGCCTGGGGTGCCTTCCGTCACTTCCAGTTAGAGCAGGACAAGCCCAAGGAGCAG<br>ACAGCGCATGCCTTTGTAAACGTCCTCACCCGAGGGGACCTCGCCTCCATCCGCTGGGTCTCCTCCCCCCTGAA<br>GCACACGCAGCCCTCGAGCTCAGGAGCACAGCTCTGCACTGTCTACTACGCCTCACTGAACTTCCGAGACATC<br>ATGCTGGCCACGGGCAAGCTGTCCCCTGATGCCATTCCAGGTAAATGGGCCAGCCGAGACTGCATGCTCGGCA<br>TGGAGTTCTCAGGCCGGGATAGGTGTGGCCGGCGTGTGATGGGGCTGGTTCCTGCAGAAGGCCTGGCCACCTC<br>AGTCCTGCTATCATCTGACTTCCTCTGGGATGTACCCTCCAGCTGGACCCTGGAGGAGGCGGCCTCTGTGCCCG<br>TCGTCTATACCACTGCTTACTACTCGTTAGTGGTTCGCGGGCGCATCCAGCGTGGGGAGACCGTGCTCATCCA<br>CTCAGGTTCAGGTGGTGTGGGCCAAGCGGCCATTTCCATTGCCCTCAGTCTGGGCTGCCGCGTCTTCACCACTG<br>TGGGCTCTGCAGAGAAGCGAGCATACCTCCAGGCCAGGTTCCCTCAGCTTGATGACACCAGCTTTGCCAACTC<br>GAGGGACACATCATTTGAGCAGCACGTGTTACTGCACACAGGTGGCAAAGGGGTCGACCTGGTCCTCAACTCA<br>CTGGCAGAAGAGAAGCTGCAGGCCAGTGTGCGGTGCTTGGCTCAGCATGGTCGCTTCTTAGAGATTGGCAAAT<br>TTGATCTTTCTAACAACCACCCTCTGGGCATGGCTATCTTCTTGAAGAACGTCACTTTCCATGGGATCCTGCT<br>GGACGCCCTTTTTGAGGAGGCCAATGACAGCTGGCGGGAGGTGGCGGCACTCCTGAAGGCTGGCATTCGTGAT<br>GGAGTCGTGAAGCCCCTCAAGTGCACAGTGTTTCCCAAGGCCCAGGTGGAAGATGCCTTCCGCTACATGGCTC<br>AGGGGAAACACATTGGCAAAGTCCTTGTCCAGGTACGGGAGGAGGAGCCTGAGGCTGTGCTGCCAGGGGCTCA<br>GCCCACCCTGATTTCTGCCATCTCCAAGACCTTCTGCCCAGCCCATAAGAGTTACATCATCACTGGTGGCCTAG<br>GTGGCTTTGGCCTGGAGCTGGCCCGGTGGCTCGTGCTTCGCGGAGCCCAGAGGCTTGTGCTGACTTCCCGATC<br>TGGAATCCGCACCGGCTACCAAGCCAAGCACATTCGGGAGTGGAGACGCCAGGGCATCCAAGTGCTCGTGTCA<br>ACAAGCAACGTGAGCTCACTGGAGGGGGCCCGTGCTCTCATCGCCGAAGCCACAAAGCTGGGGCCCGTTGGGG<br>GTGTCTTCAACCTGGCCATGGTTTTGAGGGATGCCATGCTGGAGAACCAGACCCCAGAGCTCTTCCAGGATGT<br>CAACAAGCCCAAATACAATGGCACCCTGAACCTTGACAGGGCAACCCGGGAAGCCTGCCCTGAGCTGGACTAC<br>TTTGTGGCCTTCTCCTCTGTAAGCTGCGGGCGTGGTAATGCTGGCCAAACTAACTACGGCTTCGCCAACTCTA<br>CCATGGAGCGTATATGTGAACAGCGCAGGCACGATGGCCTCCCAGGCCTTGCCGTGCAGTGGGGTGCCATTGG<br>TGACGTGGGCATTGTCCTGGAAGCGATGGGCACCAATGACACAGTCATCGGAGGTACGCTGCCTCAGCGCATC<br>TCCTCCTGCATGGAGGTACTGGACCTCTTCCTGAATCAGCCCCACGCAGTCCTGAGCAGCTTTGTGCTGGCAG

Construct	Sequence form start to stop codon
	AGAAGAAAGCTGTGGCCCATGGGGACGGGGACACCCAGAGGGATCTGGTGAAAGCTGTAGCACACATCCTAG<br>GCATCCGAGACCTCGCAGGTATTAACCTGGACAGCACGCTGGCAGACCTCGGCCTGGACTCGCTCATGGGTGT<br>GGAAGTTCGTCAGATCCTGGAACGAGAACACGATCTGGTGCTGCCCATGCGTGAGGTGCGGCAGCTCACGCTG<br>CGGAAACTTCAGGAAATGTCCTCCAAGACTGACTCGGCTACTGACACGACAGCCCCCAAGTCCAGGAGTGACA<br>CGTCTCTGAAGCAGAACCAACTGAACCTGAGCACACTGCTGGTGAACCCTGAGGGTCCTACCCTAACCCAGCT<br>CAACTCGGTGCAGAGCTCTGAGCGGCCTCTGTTCCTTGTGCACCCCATTGAGGGTTCCACCACCGTGTTCCACA<br>GTCTGGCTGCCAAGCTCAGTGTGCCCACCTACGGCCTGCAGTGCACCCAAGCTGCCCCCCTGGATAGCATTCCG<br>AACCTGGCTGCCTACTACATAGATTGCATCAAGCAAGTGCAGCCTGAGGGACCCTACCGCATAGCTGGGTACT<br>CATTTGGAGCCTGTGTAGCCTTCGAGATGTGCTCCCAGCTGCAGGCCCAGCAGGGCCCAGCCCCGACCCACAAC<br>AACCTCTTCCTGTTTGACGGCTCACACACCTACGTGTTGGCCTACACCCAGAGCTACCGGGCAAAGATGACCC<br>CAGGCTGTGAAGCCGAGGCCGAGGCTGAGGCCTTATGCTTCTTCATAAAGCAGTTTCTTGATGTGGAACACAG<br>CAAGGTGCTGGAGGCCCTGCTGCCACTGAAGAGCCTGGAAGATCGGGTGGCTGCCTCCGTGGACCTTATCACT<br>AAGAGTCACCACAGCCTGGACCGCCGAGAGCTGAGCTTTGCTGCCGTGTCCTTCTACCACAAGCTCCGGGCAG<br>CTGATCAGTATAAGCCCAAGGCCAAGTACCATGGCAACGTGACACTGCTGCGTGCCAAGACAGGCGGCACCTA<br>TGGCGAGGACTTGGGTGCTGACTACAACCTCTCCCAGGTGTGTGACGGGAAGGTGTCTGTGCACATCATTGAG<br>GGTGACCACCGCACACTGCTGGAGGGCAGTGGCCTGGAATCCATCATCAACATCATCCATAGCTCCCTGGCTG<br>AGCCACGAGTGAGTGTACGGGAGGGCCTCGAGCATCATCACCACCACCACCACCACTGA
pAR435<br>(mFAS/'TesA)<br>pAR436<br>(mFAS/'TesA_L109P)<br>pDL017<br>(mFAS/'TesA_4x)<br>pDL021<br>(mFAS^{G113S}/'TesA)<br>pDL028<br>(mFAS^{G113S}/'TesA_L109P)<br>pDL029<br>(mFAS^{G113S}/'TesA_4x)<br>pDL007<br>(mFAS^{G113M}/'TesA)<br>pDL010<br>(mFAS^{G113M}/'TesA_L109P)<br>pDL018<br>(mFAS^{G113M}/'TesA_4x)<br>pDL008<br>(mFAS^{G113F}/'TesA)<br>pDL011<br>(mFAS^{G113F}/'TesA_L109P)<br>pDL019<br>(mFAS^{G113F}/'TesA_4x)<br>pDL026<br>(mFAS^{G113W}/'TesA)<br>pDL031<br>(mFAS^{G113W}/'TesA_L109P)<br>pDL032<br>(mFAS^{G113W}/'TesA_4x)<br>(StrepI_mouseFAS/'TesA_H8)<br>Position 113<br>Highlighted in red<br>TesA_L109<br>highlighted in blue<br>Positions for TesA4x<br>highlighted in green	MSAWSHPQFEKGGGSGGGSGGSAWSHPQFEKGAGSEEVVIAGMSGKLPESENLQEFWANLIGGVDMVTDDDR<br>RWKAGLYGLPKRSGKLKDLSKFDASFFGVHPKQAHTMDPQLRLLLEVSYEAIVDGGINPASLRGTNTGVWVGV<br>S**G/S/M/F/W**SEASEALSRDPETLLGYSMVGCQRAMMANRLSFFFDFKGPSIALDTACSSSLLALQNAYQAIRS<br>GECPAALVGGINLLLKPNTSVQFMKLGMLSPDGTCRSFDDSGSGYCRSEAVVAVLLTKKSLARRVYATILNAGT<br>NTDGSKEQGVTFPSGEVQEQLICSLYQPAGLAPESLEYIEAHGTGTKVGDPQELNGITRSLCAFRQAPLLIGSTKS<br>NMGHPEPASGLAALTKVLLSLEHGVWAPNLHFHNPNPEIPALLDGRLQVVDRPLPVRGGNVGINSFGFGGSNV<br>HVILQPNTRQAPAPTAHAALPHLLHASGRTLEAVQDLLEQGRQHSQDLAFVSMLNDIAATPTAAMPFRGYTVL<br>GVEGRVQEVQQVSTNKRPLWFICSGMGTQWRGMGLSLMRLDSFRESILRSDEAVKPLGVKVSDLLLSTDERTF<br>DDIVHAFVSLTAIQIALIDLLTSVGLKPDGIIGHSLGEVACGYADGCLSQREAVLAAYWRGQCIKDAHLPPGSMA<br>AVGLSWEECKQRCPAGVVPACHNSEDTVTISGPQAAVNEFVEQLKQEGVFAKEVRTGGLAFHSYFMEGIAPTL<br>LQALKKVIREPRPRSARWLSTSIPEAQWQSSLARTSSAEYNVNNLVSPVLFQEALWHIPEHAVVLEIAPHALLQ<br>AVLKRGVKSSCTIIPLMKRDHKDNLEFFLTNLGKVHLTGINVNPNALFPPVEFPAPRGTPLISPHIKWDHSQTW<br>DVPVAEDFPNGSSSSSATVYSIDASPESPDHYLVDHCIDGRVIFPGTGYLCLVWKTLARSLGLSLEETPVVFENVS<br>FHQATILPKTGTVALEVRLLEASHAFEVSDTGNLIVSGKVYLWEDPNSKLFDHPEVPTPPESASVSRLTQGEVY<br>KELRLRGYDYGPQFQGICEATLEGEQGKLLWKDNWVTFMDTMLQVSILGSSQQSLQLPTRVTAIYIDPATHRQ<br>KVYRLKEDTQVADVTTSRCLGITVSGGIHISRLQTTATSRRQQEQLVPTLEKFVFTPHMEAECLSESTALQKELQ<br>LCKGLARALQTKATQQGLKAAMLGQEDPPQHGLPRLLAAACQLQLNGNLQLELGEALAQERLLLPEDPLISGL<br>LNSQALKACVDTALENLSTLKMKVAEVLAGEGHLYSRIPALLNTQPMLQLEYTATDRHPQALKDVQTKLQQH<br>DVAQGQWNPSDPAPSSLGALDLLVCNCALATLGDPALALDNMVAALKEGGFLLVHTVLKGHALGETLACLPSE<br>VQPAPSLLSQEEWESLFSRKALHLVGLKRSFYGTALFLCRRAIPQEKPIFLSVEDTSFQWVDSLKSTLATSSSQP<br>VWLTAMDCPTSGVVGLVNCLRKEPGGHRIRCILLSNLSNTSHAPKLDPGSPELQQVLKHDLVMNVYRDGAWG<br>AFRHFQLEQDKPKEQTAHAFVNVLTRGDLASIRWVSSPLKHTQPSSSGAQLCTVYYASLNFRDIMLATGKLSPD<br>AIPGKWASRDCMLGMEFSGRDRCGRRVMGLVPAEGLATSVLLSSDFLWDVPSSWTLEEAASVPVVYTTAYYSL<br>VVRGRIQRGETVLIHSGSGGVGQAAISIALSLGCRVFTTVGSAEKRAYLQARFPQLDDTSFANSRDTSFEQHVLL<br>HTGGKGVDLVLNSLAEEKLQASVRCLAQHGRFLEIGKFDLSNNHPLGMAIFLKNVTFHGILLDALFEEANDSW<br>REVAALLKAGIRDGVVKPLKCTVFPKAQVEDAFRYMAQGKHIGKVLVQVREEEPEAVLPGAQPTLISAISKTFC<br>PAHKSYIITGGLGGFGLELARWLVLRGAQRLVLTSRSGIRTGYQAKHIREWRRQGIQVLVSTSNVSSLEGARALI<br>AEATKLGPVGGVFNLAMVLRDAMLENQTPELFQDVNKPKYNGTLNLDRATREACPELDYFVAFSSVSCGRGN<br>AGQTNYGFANSTMERICEQRRHDGLPGLAVQWGAIGDVGIVLEAMGTNDTVIGGTLPQRISSCMEVLDLFLNQ<br>PHAVFVLAEKKAVAHGDGDTQRDLVKAVAHILGIRDLAGINLDSTLADLGLDSLMGVEVRQILEREHDLVLPM<br>REVRQLTLRKLQEMSSKTDSATDTTAPKSRSDTSLKQNQAAAADTLLILGDSLSAGYRMSASAAWPALLNDK<br>WQSKTSVVNASISGDTSQQGLARLPALLKQHQPRWVLVELGGNDGLRGFQPQQTEQTLRQILQDVKAANAEP<br>LLMQIR**L/P**PANYGRRYNEAFSAIYPKLAKEFDVPLLPFF**M/LE/DEVY/GL/K**KPQWMQDDGIHPNRDAQPF<br>IADWMAKQLQPLVNHDSLEHHHHHHHH
pAR435<br>(mFAS/'TesA)<br>pAR436<br>(mFAS/'TesA_L109P)	ATGAGCGCTTGGAGCCATCCACAATTTGAGAAGGGTGGAGGTTCTGGCGGTGGATCGGGAGGTTCAGCGTGG<br>AGCCACCCGCAGTTCGAAAAAGGCGCCGGATCCGAGGAGGTGGTGATAGCCGGTATGTCGGGGAAGTTGCCCG<br>AGTCAGAGAACCTACAGGAGTTCTGGGCCAACCTCATTGGTGGTGTGGACATGGTCACAGATGATGACAGGA<br>GATGGAAGGCTGGGCTCTATGGATTACCCAAGCGGTCTGGAAAGCTGAAGGATCTCTCCAAGTTCGACGCCTC

Construct	Sequence form start to stop codon
pDL017<br>(mFAS/'TesA_4x)<br>pDL021<br>(mFAS^{G113S}/'TesA)<br>pDL028<br>(mFAS^{G113S}/'TesA_L10<br>9P)<br>pDL029<br>(mFAS^{G113S}/'TesA_4x)<br>pDL007<br>(mFAS^{G113M}/'TesA)<br>pDL010<br>(mFAS^{G113M}/'TesA_L1<br>09P)<br>pDL018<br>(mFAS^{G113M}/'TesA_4x)<br>pDL008<br>(mFAS^{G113F}/'TesA)<br>pDL011<br>(mFAS^{G113F}/'TesA_L10<br>9P)<br>pDL019<br>(mFAS^{G113M}/'TesA_4x)<br>pDL026<br>(mFAS^{G113W}/'TesA)<br>pDL031<br>(mFAS^{G113W}/'TesA_L1<br>09P)<br>pDL032<br>(mFAS^{G113W}/'TesA_4x)<br>(StrepI_mouseFAS/'T<br>esA_H8)<br>Position 113<br>Highlighted in red<br>TesA_L109<br>highlighted in blue<br>Positions for TesA4x<br>highlighted in green	CTTTTTTGGGGTCCACCCCAAGCAGGCACACACAATGGACCCCCAGCTTCGGCTGCTGTTGGAAGTCAGCTAT<br>GAAGCAATTGTGGATGGAGGTATCAACCCAGCCTCACTCCGAGGAACGAACACTGGCGTCTGGGTGGGTGTGA<br>GT**GGT/TCG/ATG/TTT/TGG**TCAGAGGCATCCGAGGCCCTTAGCAGAGATCCCGAGACGCTTCTGGGCTACA<br>GCATGGTGGGCTGCCAGCGTGCAATGATGGCCAACCGGCTCTCTTTCTTCTTCGACTTCAAAGGACCAAGCAT<br>TGCCCTGGACACAGCCTGCTCCTCCAGCTTGCTGGCACTACAGAATGCCTACCAGGCCATCCGTAGTGGGGAA<br>TGCCCCGCGGCCCTTGTGGGTGGGATCAACCTGCTCCTGAAGCCGAACACCTCTGTGCAGTTCATGAAGCTGG<br>GCATGCTCAGCCCGGACGGCACCTGCAGATCCTTTGATGATTCAGGGAGTGGATATTGTCGCTCTGAGGCTGT<br>TGTAGCAGTTCTGCTGACTAAGAAGTCCCTGGCTCGGCGGGTCTATGCCACGATTCTGAATGCCGGCACCAAT<br>ACAGATGGCAGCAAGGAGCAAGGTGTAACATTCCCCTCTGGAGAAGTCCAAGAACAACTCATCTGCTCTCTGT<br>ATCAGCCAGCTGGTCTGGCCCCGGAGTCGCTTGAGTATATTGAAGCCCATGGCACGGGCACCAAGGTGGGTGA<br>CCCCCAGGAACTGAATGGCATTACTCGGTCCCTGTGCGCCTTCCGCCAGGCCCCTCTGTTAATTGGCTCCACCA<br>AATCCAACATGGGACACCCTGAGCCTGCCTCTGGGCTTGCAGCCCTGACCAAGGTGCTGTTATCCCTGGAGCA<br>TGGGGTCTGGGCCCCTAACCTGCACTTCCACAACCCCAACCCTGAGATCCCAGCACTTCTTGATGGGCGGCTGC<br>AGGTGGTCGATAGGCCCCTGCCTGTTCGTGGTGGCAACGTGGGCATCAACTCATTTGGCTTCGGAGGCTCCAA<br>TGTTCATGTCATCCTCCAGCCCAACACACGGCAGGCCCCTGCGCCCACTGCACACGCTGCCCTTCCCCATTTGC<br>TGCACGCCAGTGGACGCACCTTAGAGGCAGTGCAGGACCTGCTGGAACAGGGCCGCCAGCACAGCCAGGACCT<br>GGCCTTTGTGAGCATGCTCAATGACATTGCGGCAACCCCTACAGCAGCCATGCCCTTCAGGGGTTACACTGTG<br>CTAGGTGTTGAGGGCCGTGTCCAAGAAGTGCAGCAAGTGTCCACCAACAAGCGCCCACTCTGGTTCATCTGCT<br>CAGGGATGGGCACGCAGTGGCGCGGGATGGGGCTGAGCCTCATGCGCCTGGACAGCTTCCGTGAGTCTATCCT<br>GCGCTCCGATGAGGCTGTGAAGCCGTTGGGAGTGAAAGTGTCAGATCTGCTGTTGAGCACAGATGAGCGCACC<br>TTTGATGACATCGTGCATGCCTTTGTGAGCCTCACTGCCATCCAGATTGCCCTCATCGACCTACTGACTTCTGT<br>GGGACTGAAACCTGACGGCATCATTGGGCACTCCTTGGGAGAGGTTGCCTGTGGCTATGCAGATGGCTGTCTC<br>TCCCAGAGAGAGGCTGTGCTTGCAGCTTACTGGCGAGGCCAGTGCATCAAAGATGCCCACCTCCCGCCTGGAT<br>CCATGGCAGCTGTTGGTTTGTCCTGGGAGGAATGTAAACAGCGCTGCCCCGCTGGCGTGGTGCCTGCCTGCCA<br>CAACTCTGAGGACACCGTGACCATCTCTGGACCTCAGGCTGCAGTGAATGAATTTGTGGAGCAGCTAAAGCAA<br>GAAGGTGTGTTTGCCAAGGAGGTACGAACAGGAGGCCTGGCTTTCCACTCCTACTTCATGGAAGGAATTGCCC<br>CCACATTGCTGCAGGCTCTCAAGAAGGTGATCCGGGAACCACGGCCGCGCTCGGCTCGATGGCTCAGCACCTC<br>TATCCCTGAGGCCCAGTGGCAGAGCAGCCTGGCCCGCACATCTTCTGCCGAGTACAATGTCAACAACCTGGTG<br>AGCCCTGTGCTCTTCCAGGAAGCACTGTGGCACATCCCTGAGCATGCCGTGGTGCTGGAGATTGCGCCCCACG<br>CACTGTTGCAGGCTGTCCTGAAGCGAGGCGTGAAGTCCAGCTGCACCATCATTCCCTTGATGAAGAGGGATCA<br>TAAAGATAACTTGGAGTTCTTTCTCACCAACCTTGGCAAGGTGCACCTCACAGGCATCAATGTCAACCCTAAC<br>GCCTTGTTCCCACCTGTGGAGTTCCCGGCTCCCCGAGGGACTCCTCTCATCTCCCCTCACATCAAGTGGGACCA<br>CAGTCAGACTTGGGATGTCCCGGTTGCTGAGGACTTCCCAAACGGCTCCAGCTCCTCCTCTGCTACAGTCTAC<br>AGCATCGACGCCAGTCCTGAGTCGCCCGACCACTACCTGGTAGACCACTGCATTGACGGCCGGGTCATCTTCCC<br>TGGCACTGGCTACCTGTGCCTGGTGTGGAAGACACTGGCTCGCAGCCTGGGCTTGTCCCTAGAAGAGACCCCT<br>GTGGTATTTGAGAATGTGTCGTTTCATCAGGCCACTATACTACCCAAGACAGGAACCGTGGCGCTGGAGGTGA<br>GGCTGCTAGAGGCCTCCCATGCCTTTGAGGTGTCTGACACTGGCAATCTGATTGTGAGCGGAAAAGTGTACCT<br>GTGGGAAGACCCGAACTCCAAGTTATTCGACCACCCAGAAGTCCCAACACCCCCTGAGTCTGCATCGGTCTCC<br>CGCCTGACCCAGGGAGAAGTATACAAGGAGCTGCGGCTGCGTGGCTATGATTATGGCCCTCAGTTCCAGGGCA<br>TCTGTGAGGCCACCCTTGAAGGTGAACAAGGCAAGCTGCTCTGGAAAGATAACTGGGTGACCTTCATGGACAC<br>AATGCTGCAGGTATCCATTCTGGGTTCTAGCCAGCAGAGTCTACAGCTACCTACCCGTGTGACCGCCATCTAT<br>ATCGACCCTGCCACCCACCGTCAGAAGGTGTACAGGCTGAAGGAGGACACTCAAGTGGCTGATGTGACAACGA<br>GCCGCTGTCTGGGCATAACGGTCTCTGGTGGTATCCACATCTCAAGACTACAGACGACAGCAACCTCACGGCG<br>GCAGCAAGAACAGCTGGTCCCCACCTTGGAAAAGTTCGTTTTCACACCGCACATGGAGGCTGAGTGCCTGTCT<br>GAGAGCACTGCCCTGCAGAAGGAGCTGCAACTGTGCAAGGGTCTGGCACGGGCTCTGCAGACCAAGGCCACCC<br>AGCAAGGGCTGAAGGCGGCAATGCTTGGGCAAGAGGACCCTCCACAGCACGGGCTGCCTCGACTCCTGGCAGC<br>TGCTTGCCAGTTGCAGCTCAACGGGAACCTGCAGCTGGAGCTGGGAGAAGCGCTGGCTCAAGAGAGGCTCCTG<br>CTGCCAGAAGACCCTCTGATCAGTGGCCTCCTCAACTCCCAGGCCCTCAAGGCCTGCGTAGACACAGCCCTGGA<br>GAACTTGTCTACTCTCAAGATGAAGGTGGCAGAGGTGCTGGCTGGAGAAGGCCACTTGTATTCCCGAATCCCG<br>GCACTGCTCAACACCCAGCCCATGCTACAACTGGAATACACAGCCACCGACCGGCACCCCCAGGCCCTGAAGG<br>ATGTTCAGACCAAACTGCAGCAGCATGATGTGGCGCAGGGCCAGTGGAACCCTTCCGACCCTGCGCCCAGCAG<br>CCTGGGTGCCCTTGACCTTCTGGTGTGCAACTGTGCATTAGCCACCCTGGGGGATCCAGCCTTGGCCCTGGAC<br>AACATGGTAGCTGCCCTCAAGGAAGGTGGTTTCCTGCTAGTGCACACAGTGCTCAAAGGACATGCCCTTGGGG<br>AGACCCTGGCCTGCCTACCCTCTGAGGTGCAGCCTGCGCCCAGCCTCCTAAGCCAGGAGGAGTGGGAGAGCCT<br>GTTCTCGAGGAAGGCACTACACCTGGTGGGCCTTAAAAGGTCCTTCTACGGTACTGCGCTGTTCCTGTGCCGG<br>CGAGCCATCCCACAGGAGAAACCTATCTTCCTGTCTGTGGAGGATACCAGCTTCCAGTGGGTGGACTCTCTGA<br>AGAGCACTCTGGCCACGTCCTCCTCCCAGCCTGTGTGGCTAACGGCCATGGACTGCCCCACCTCGGGTGTGGTG<br>GGTTTGGTGAATTGTCTCCGAAAAGAGCCGGGTGGACACCGGATTCGGTGTATCCTGCTGTCCAACCTCAGCA<br>ACACATCTCACGCCCCCAAGTTGGACCCTGGCTCTCCAGAGCTACAGCAGGTGCTAAAGCATGACCTCGTGAT<br>GAACGTGTACCGGGACGGGGCCTGGGGTGCCTTCCGTCACTTCCAGTTAGAGCAGGACAAGCCCAAGGAGCAG<br>ACAGCGCATGCCTTTGTAAACGTCCTCACCCGAGGGGACCTCGCCTCCATCCGCTGGGTCTCCTCCCCCCTGAA<br>GCACACGCAGCCCTCGAGCTCAGGAGCACAGCTCTGCACTGTCTACTACGCCTCACTGAACTTCCGAGACATC<br>ATGCTGGCCACGGGCAAGCTGTCCCCTGATGCCATTCCAGGTAAATGGGCCAGCCGAGACTGCATGCTCGGCA

Construct	Sequence form start to stop codon
	TGGAGTTCTCAGGCCGGGATAGGTGTGGCCGGCGTGTGATGGGGCTGGTTCCTGCAGAAGGCCTGGCCACCTC<br>AGTCCTGCTATCATCTGACTTCCTCTGGGATGTACCCTCCAGCTGGACCCTGGAGGAGGCGGCCTCTGTGCCCG<br>TCGTCTATACCACTGCTTACTACTCGTTAGTGGTTCGCGGGCGCATCCAGCGTGGGGAGACCGTGCTCATCCA<br>CTCAGGTTCAGGTGGTGTGGGCCAAGCGGCCATTTCCATTGCCCTCAGTCTGGGCTGCCGCGTCTTCACCACTG<br>TGGGCTCTGCAGAGAAGCGAGCATACCTCCAGGCCAGGTTCCCTCAGCTTGATGACACCAGCTTTGCCAACTC<br>GAGGGACACATCATTTGAGCAGCACGTGTTACTGCACACAGGTGGCAAAGGGGTCGACCTGGTCCTCAACTCA<br>CTGGCAGAAGAGAAGCTGCAGGCCAGTGTGCGGTGCTTGGCTCAGCATGGTCGCTTCTTAGAGATTGGCAAAT<br>TTGATCTTTCTAACAACCACCCTCTGGGCATGGCTATCTTCTTGAAGAACGTCACTTTCCATGGGATCCTGCT<br>GGACGCCCTTTTTGAGGAGGCCAATGACAGCTGGCGGGAGGTGGCGGCACTCCTGAAGGCTGGCATTCGTGAT<br>GGAGTCGTGAAGCCCCTCAAGTGCACAGTGTTTCCCAAGGCCCAGGTGGAAGATGCCTTCCGCTACATGGCTC<br>AGGGGAAACACATTGGCAAAGTCCTTGTCCAGGTACGGGAGGAGGAGCCTGAGGCTGTGCTGCCAGGGGCTCA<br>GCCCACCCTGATTTCTGCCATCTCCAAGACCTTCTGCCCAGCCCATAAGAGTTACATCATCACTGGTGGCCTAG<br>GTGGCTTTGGCCTGGAGCTGGCCCGGTGGCTCGTGCTTCGCGGAGCCCAGAGGCTTGTGCTGACTTCCCGATC<br>TGGAATCCGCACCGGCTACCAAGCCAAGCACATTCGGGAGTGGAGACGCCAGGGCATCCAAGTGCTCGTGTCA<br>ACAAGCAACGTGAGCTCACTGGAGGGGGCCCGTGCTCTCATCGCCGAAGCCACAAAGCTGGGGCCCGTTGGGG<br>GTGTCTTCAACCTGGCCATGGTTTTGAGGGATGCCATGCTGGAGAACCAGACCCCAGAGCTCTTCCAGGATGT<br>CAACAAGCCCAAATACAATGGCACCCTGAACCTTGACAGGGCAACCCGGGAAGCCTGCCCTGAGCTGGACTAC<br>TTTGTGGCCTTCTCCTCTGTAAGCTGCGGGCGTGGTAATGCTGGCCAAACTAACTACGGCTTCGCCAACTCTA<br>CCATGGAGCGTATATGTGAACAGCGCAGGCACGATGGCCTCCCAGGCCTTGCCGTGCAGTGGGGTGCCATTGG<br>TGACGTGGGCATTGTCCTGGAAGCGATGGGCACCAATGACACAGTCATCGGAGGTACGCTGCCTCAGCGCATC<br>TCCTCCTGCATGGAGGTACTGGACCTCTTCCTGAATCAGCCCCACGCAGTCCTGAGCAGCTTTGTGCTGGCAG<br>AGAAGAAAGCTGTGGCCCATGGGGACGGGGACACCCAGAGGGATCTGGTGAAAGCTGTAGCACACATCCTAG<br>GCATCCGAGACCTCGCAGGTATTAACCTGGACAGCACGCTGGCAGACCTCGGCCTGGACTCGCTCATGGGTGT<br>GGAAGTTCGTCAGATCCTGGAACGAGAACACGATCTGGTGCTGCCCATGCGTGAGGTGCGGCAGCTCACGCTG<br>CGGAAACTTCAGGAAATGTCCTCCAAGACTGACTCGGCTACTGACACGACAGCCCCCAAGTCCAGGAGTGACA<br>CGTCTCTGAAGCAGAACCAAGCCGCCGCAGCGGACACGTTATTGATTCTGGGTGATAGCCTGAGCGCCGGGTA<br>TCGAATGTCTGCCAGCGCGGCCTGGCCTGCCTTGTTGAATGATAAGTGGCAGAGTAAAACGTCGGTAGTTAAT<br>GCCAGCATCAGCGGCGACACCTCGCAACAAGGACTGGCGCGCCTTCCGGCTCTGCTGAAACAGCATCAGCCGC<br>GTTGGGTGCTGGTTGAACTGGGCGGCAATGACGGTTTGCGTGGTTTTCAGCCACAGCAAACCGAGCAAACGCT<br>GCGCCAGATTTTGCAGGATGTCAAAGCCGCCAACGCTGAACCATTGTTAATGCAAATACGT**CTG**/CCGCCTGC<br>AAACTATGGTCGCCGTTATAATGAAGCCTTTAGCGCCATTTACCCCAAACTCGCCAAAGAGTTTGATGTTCCG<br>CTGCTGCCCTTTTTT**ATG**/CTG**GAA**/GATGAGGTC**TAC**/GGC**CTC**/AAGAAGCCACAATGGATGCAGGATGA<br>CGGTATTCATCCCAACCGCGACGCCCAGCCGTTTATTGCCGACTGGATGGCGAAGCAGTTGCAGCCTTTAGTA<br>AATCATGACTCACTCGAGCATCATCACCACCACCACCACTGA
Codon-optimized sequence for *O. polymorpha* (mFAS^{g113m}_TesA)	ATGTCTGCCGGCGCTGGCTCGGAAGAGGTGGTGATCGCCGGCATGTCGGGCAAGCTGCCTGAGTCGGAGAACC<br>TGCAAGAGTTCTGGGCCAACCTGATCGGCGGCGTGGACATGGTGACCGACGATGACCGCAGATGGAAAGCCGG<br>CCTGTATGGACTGCCAAAGAGATCTGGCAAGTTGAAGGACCTGTCGAAGTTCGACGCCTCGTTCTTCGGCGTG<br>CACCCAAAGCAAGCCCACACCATGGACCCACAGCTGAGACTGTTGCTGGAGGTGTCGTACGAGGCCATCGTGG<br>ACGGCGGCATTAACCCTGCCTCGCTGAGAGGCACCAACACCGGCGTGTGGGTGGGCGTGTCGATGTCGGAGGC<br>CTCGGAGGCCCTGTCGAGAGACCCTGAGACCCTGCTGGGCTACTCGATGGTGGGCTGTCAGAGAGCCATGATG<br>GCCAACAGACTGTCGTTCTTTTTCGACTTCAAGGGCCCATCGATCGCCCTGGACACCGCCTGCTCGTCTTCGCT<br>GCTGGCCCTGCAGAACGCCTACCAAGCCATCAGATCGGGCGAGTGCCCTGCCGCCCTGGTGGGCGGCATCAACC<br>TGTTGCTGAAGCCAAACACCTCGGTGCAGTTCATGAAGCTGGGCATGCTGTCGCCTGACGGCACCTGCAGATC<br>GTTCGACGACTCGGGCTCGGGCTACTGCAGATCGGAGGCCGTGGTGGCCGTGCTGCTGACCAAGAAGTCGTTG<br>GCTAGAAGAGTTTACGCTACCATCCTGAACGCCGGCACCAATACCGACGGCTCGAAGGAGCAAGGCGTGACCT<br>TCCCATCGGGCGAGGTGCAAGAGCAGCTGATCTGCTCGCTGTATCAGCCTGCCGGCCTGGCCCCTGAGTCGCT<br>GGAGTACATCGAGGCCCACGGCACCGGCACCAAGGTGGGCGACCCACAAGAGCTGAATGGCATCACGAGATCG<br>CTGTGCGCCTTCAGACAAGCCCCTTTGTTGATCGGCTCGACCAAGTCTAACATGGGCCACCCTGAGCCTGCCTC<br>GGGCCTGGCCGCCTTGACCAAGGTGCTGCTGTCGCTGGAGCACGGAGTGTGGGCCCCAAACCTGCACTTCCAC<br>AACCCAAACCCTGAGATCCCTGCCCTGCTGGACGGCAGACTGCAAGTGGTGGACAGACCACTGCCTGTGAGAG<br>GCGGCAACGTGGGCATCAACTCGTTCGGCTTCGGCGGCTCGAACGTGCACGTGATCCTGCAGCCTAACACGAG<br>ACAAGCCCCTGCCCCAACCGCCCACGCCGCTCTGCCACACCTGCTGCACGCCTCTGGCAGAACCCTGGAGGCCG<br>TGCAAGACCTGCTGGAGCAAGGCAGACAGCACTCGCAAGACCTGGCTTTCGTGTCGATGCTGAACGACATCGC<br>CGCCACCCCTACCGCCGCCATGCCATTCAGAGGCTACACCGTGCTGGGCGTGGAGGGCAGAGTGCAAGAGGTG<br>CAGCAAGTGTCGACCAACAAGAGACCACTGTGGTTCATCTGCTCGGGCATGGGCACGCAGTGGAGAGGCATGG<br>GCCTGTCGCTGATGAGACTGGACTCGTTCAGAGAGTCGATCCTGAGATCGGACGAGGCCGTGAAGCCACTGGG<br>CGTGAAGGTGTCGGACCTGTTGCTGTCGACCGACGAGAGAACCTTCGACGACATCGTGCACGCCTTCGTGTCG<br>CTGACGGCCATTCAGATCGCCCTGATCGACCTGCTGACCTCGGTGGGCCTGAAGCCTGACGGCATCATCGGCC<br>ACTCGCTGGGCGAGGTTGCCTGCGGCTACGCCGACGGCTGCTTGTCGCAGAGAGAGGCCGTGCTGGCCGCCTA<br>CTGGAGAGGACAGTGCATCAAGGACGCCCACCTGCCACCTGGCTCGATGGCCGCCGTGGGCCTGTCGTGGGAG<br>GAGTGCAAGCAGAGATGCCCTGCCGGCGTGGTGCCTGCCTGCCACAACTCGGAGGACACCGTGACCATCTCGG<br>GCCCACAAGCCGCCGTGAACGAGTTCGTGGAGCAGCTGAAGCAAGAGGGCGTGTTCGCCAAAGAGGTGAGAAC<br>CGGCGGCCTGGCCTTCCACTCGTACTTCATGGAGGGCATCGCCCCAACCCTGCTGCAAGCCCTGAAGAAGGTG

Construct	Sequence form start to stop codon
	ATCAGAGAGCCAAGACCAAGATCGGCTAGATGGCTGTCGACCTCGATCCCTGAGGCTCAGTGGCAGTCGTCGC<br>TGGCCCGCACCTCGTCGGCCGAGTACAACGTGAACAACCTGGTGTCGCCTGTGCTGTTCCAAGAGGCCCTGTG<br>GCACATCCCTGAGCACGCCGTGGTGCTGGAGATCGCCCCACACGCCCTGCTGCAAGCCGTGTTGAAGAGAGGC<br>GTGAAGTCGTCGTGCACCATCATCCCACTGATGAAGAGAGACCACAAGGACAACCTGGAGTTCTTCCTGACCA<br>ACCTGGGCAAGGTGCACCTGACCGGCATCAACGTGAACCCAAACGCCCTGTTCCCACCTGTGGAGTTCCCTGCC<br>CCAAGAGGCACCCCTTTGATCTCGCCACACATCAAGTGGGACCACTCGCAGACCTGGGACGTGCCTGTGGCCG<br>AGGACTTCCCAAACGGCTCGTCTTCGTCTTCGGCCACCGTGTACTCGATCGACGCCTCGCCTGAGTCGCCTGAC<br>CACTACCTGGTGGACCACTGCATCGACGGCAGAGTGATCTTCCCTGGCACCGGCTACCTGTGCCTGGTGTGGA<br>AGACCCTGGCTAGATCGCTGGGCCTGTCTCTGGAGGAGACCCCTGTGGTGTTCGAGAACGTGTCGTTCCACCA<br>AGCCACCATCCTGCCAAAGACCGGCACCGTGGCCCTGGAGGTGAGACTGCTGGAGGCCTCGCACGCCTTCGAG<br>GTGTCGGACACCGGCAACCTGATCGTGTCGGGCAAGGTGTACCTGTGGGAGGACCCAAACTCGAAGCTGTTCG<br>ACCACCCTGAGGTGCCAACCCCACCTGAGTCGGCCTCGGTGTCGAGACTGACCCAAGGCGAGGTGTACAAGGA<br>GCTGAGACTGAGAGGCTACGACTACGGCCCACAGTTCCAAGGCATCTGCGAGGCCACCCTGGAGGGCGAGCAA<br>GGCAAGCTGCTGTGGAAGGACAACTGGGTGACCTTCATGGACACCATGCTGCAAGTGTCGATCCTGGGCTCGT<br>CGCAGCAGTCGCTGCAGCTGCCAACGAGAGTGACCGCCATCTACATCGACCCTGCCACCCATAGACAGAAGGT<br>TTACAGACTGAAGGAGGACACCCAAGTGGCCGACGTGACCACCTCGAGATGCCTGGGCATCACCGTGTCGGGC<br>GGCATCCACATCTCGAGACTGCAGACCACCGCCACCTCGAGAAGACAACAAGAGCAACTGGTTCCAACCCTGG<br>AGAAGTTCGTGTTCACCCCACACATGGAGGCCGAGTGCCTGTCGGAGTCGACCGCCCTGCAGAAGGAGCTGCA<br>GCTGTGCAAGGGCCTGGCTAGAGCCCTGCAGACCAAGGCCACGCAGCAAGGCCTGAAGGCCGCCATGCTGGGC<br>CAAGAGGACCCACCACAGCACGGCCTGCCAAGACTGCTGGCCGCTGCCTGTCAGCTGCAGCTGAACGGCAACC<br>TGCAACTGGAGTTGGGCGAGGCCCTGGCCCAAGAGCGCCTGCTGTTGCCTGAGGACCCACTGATCTCGGGCCT<br>GCTGAACTCGCAAGCCTTGAAGGCCTGCGTGGACACCGCCCTGGAGAACCTGTCGACCCTGAAGATGAAGGTG<br>GCCGAGGTGCTGGCCGGCGAGGGCCACCTGTACTCGAGAATCCCTGCCTTGTTGAATACGCAGCCAATGCTGC<br>AGCTGGAGTACACGGCCACCGACAGACATCCACAAGCCCTGAAAGATGTGCAGACCAAGCTGCAGCAGCACGA<br>CGTGGCCCAAGGACAGTGGAACCCATCGGACCCTGCCCCATCGTCGTTGGGCGCCTTGGACTTGCTGGTGTGC<br>AATTGCGCCCTGGCTACCCTGGGCGACCCTGCCCTGGCCCTGGACAACATGGTGGCCGCCTTGAAGGAAGGCG<br>GCTTCCTGCTGGTTCACACCGTGCTGAAGGGCCACGCCCTGGGAGAAACCCTGGCCTGCTTGCCATCGGAGGT<br>GCAGCCTGCCCCATCGCTGCTGTCGCAAGAGGAGTGGGAGTCGCTGTTCTCGAGAAAGGCCCTGCACCTGGTG<br>GGCCTGAAGAGATCGTTCTACGGCACCGCCCTGTTCCTGTGCAGAAGAGCCATCCCACAAGAGAAGCCAATCT<br>TCCTGTCGGTGGAGGACACGTCGTTTCAGTGGGTGGACTCGTTGAAGTCGACCCTGGCTACGTCGTCTTCGCA<br>GCCTGTGTGGCTGACCGCCATGGACTGCCCAACCTCGGGCGTGGTGGGCCTGGTGAACTGCCTGAGAAAGGAG<br>CCTGGCGGCCACAGAATCAGATGCATCCTGCTGTCGAACCTGTCTAACACCTCTCACGCCCCAAAGCTGGACCC<br>TGGCTCGCCTGAGCTGCAGCAAGTGCTGAAGCACGACCTGGTGATGAACGTGTACAGAGACGGCGCCTGGGGC<br>GCCTTCAGACACTTTCAGCTGGAGCAAGACAAGCCAAAGGAGCAGACCGCCCACGCCTTCGTGAACGTGCTGA<br>CGAGAGGCGACCTGGCCTCGATCAGATGGGTGTCGTCGCCACTGAAGCACACGCAGCCATCGTCTTCGGGCGC<br>TCAGCTGTGCACCGTGTACTACGCCTCGCTGAACTTCAGAGACATCATGCTGGCCACCGGCAAGCTGTCGCCT<br>GACGCCATCCCTGGCAAGTGGGCCTCGAGAGACTGCATGCTGGGCATGGAGTTCTCGGGCAGAGACAGATGCG<br>GCAGAAGAGTGATGGGCCTGGTGCCTGCCGAAGGCCTGGCCACCTCGGTGCTGCTGTCGTCTGACTTCCTGTG<br>GGACGTGCCATCGTCGTGGACCCTGGAGGAGGCCGCCTCGGTGCCTGTGGTGTACACCACCGCCTACTACTCGC<br>TGGTGGTGAGAGGCAGAATTCAGAGAGGCGAGACCGTGCTGATCCACTCGGGCTCTGGCGGCGTGGGCCAAGC<br>TGCCATCTCGATCGCCCTGTCGCTGGGCTGCAGAGTGTTCACCACCGTGGGCTCGGCCGAGAAGAGAGCCTAC<br>CTGCAAGCTAGATTCCCACAGCTGGACGACACCTCGTTTGCTAATTCGAGAGACACCTCGTTCGAACAACACG<br>TGCTGCTGCACACCGGCGGCAAGGGCGTGGACCTGGTGCTGAACTCGCTGGCCGAGGAGAAGCTGCAAGCCTC<br>GGTGAGATGCCTGGCTCAGCACGGCAGATTCCTGGAGATCGGCAAGTTCGACCTGTCGAACAATCACCCACTG<br>GGCATGGCCATCTTCCTGAAGAACGTGACCTTCCACGGCATCCTGCTGGACGCCCTGTTCGAGGAGGCCAACG<br>ACTCGTGGAGAGAGGTGGCTGCCCTGCTGAAGGCCGGAATCAGAGATGGCGTGGTGAAGCCACTGAAGTGCAC<br>CGTGTTCCCAAAGGCCCAAGTGGAGGACGCCTTCAGATACATGGCCCAAGGCAAGCACATCGGCAAGGTGCTG<br>GTGCAAGTGAGAGAGGAAGAGCCTGAGGCCGTGCTGCCTGGCGCTCAGCCAACCCTGATCTCGGCCATCTCGA<br>AGACCTTCTGCCCTGCCCACAAGTCGTACATCATCACCGGCGGCCTGGGCGGCTTCGGCCTGGAGCTGGCTAG<br>ATGGCTGGTGCTGAGAGGAGCTCAGAGACTGGTGCTGACCTCGAGATCGGGCATCAGAACCGGCTACCAAGCC<br>AAGCACATCAGAGAGTGGAGAAGACAAGGCATCCAAGTGCTGGTGTCGACCTCGAATGTGTCGTCGCTGGAG<br>GGCGCTAGAGCCCTGATCGCCGAGGCCACCAAGCTGGGCCCTGTGGGCGGCGTGTTCAACCTGGCCATGGTTC<br>TGAGAGACGCCATGCTGGAGAATCAGACCCCTGAGCTGTTCCAAGACGTGAACAAGCCAAAGTACAACGGCAC<br>CCTGAACCTGGACAGAGCCACGAGAGAGGCCTGCCCTGAGCTGGACTACTTCGTGGCCTTCTCGTCGGTGTCG<br>TGCGGCAGAGGCAACGCCGGACAGACCAACTACGGCTTCGCCAACTCGACCATGGAGAGAATCTGCGAGCAGC<br>GCCGCCACGACGGACTGCCTGGCCTGGCTGTGCAGTGGGGCGCTATCGGCGACGTGGGCATCGTGCTGGAGGC<br>CATGGGCACCAACGACACCGTGATCGGCGGCACCCTGCCACAGAGAATCTCGTCGTGCATGGAGGTGCTGGAC<br>CTGTTCCTGAATCAGCCACACGCCGTGCTGTCGTCTTTTGTGCTGGCCGAGAAGAAGGCTGTGGCTCACGGCG<br>ACGGCGACACGCAGAGAGACCTGGTGAAGGCCGTGGCCCATATCCTGGGCATCAGAGACCTGGCCGGCATCAA<br>CCTGGACTCGACCCTGGCCGATCTGGGCTTGGACTCGCTGATGGGCGTGGAGGTGAGACAAATCCTGGAGAGA<br>GAGCACGATCTGGTGCTGCCAATGAGAGAGGTTAGACAGCTGACCCTGAGAAAGCTGCAAGAGATGTCGTCG<br>AAGACCGACTCGGCCACCGACACCACCGCCCCAAAGTCGAGATCGGACACCTCGCTGAAGCAGAACCAAGCCG<br>CTGCCGCCGACACCCTGCTGATCCTGGGCGACTCGCTGTCGGCCGGCTACAGAATGTCGGCCTCGGCCGCCTGG

Construct	Sequence form start to stop codon
	CCTGCCCTGCTGAACGACAAGTGGCAGTCGAAGACCTCGGTGGTGAACGCCTCGATCTCGGGCGACACCTCGC<br>AGCAAGGCCTGGCTAGACTGCCTGCCTTGCTGAAGCAGCATCAGCCAAGATGGGTGCTGGTGGAGCTGGGCGG<br>CAACGACGGCCTGAGAGGCTTTCAGCCACAGCAGACCGAGCAGACCCTGAGACAGATCCTGCAAGACGTGAAG<br>GCCGCCAACGCCGAGCCACTGCTGATGCAGATCAGACTGCCTGCCAATTACGGCAGAAGATACAACGAGGCCT<br>TCTCGGCCATTTACCCAAAGCTGGCCAAGGAGTTCGACGTGCCACTGCTGCCATTCTTCATGGAAGAGGTGTA<br>CCTGAAGCCACAGTGGATGCAAGACGACGGCATCCACCCAAACAGAGACGCTCAGCCATTCATCGCCGACTGG<br>ATGGCCAAGCAGCTGCAGCCACTGGTGAACCACGACTCGTAA
Codon-optimized sequence for *O. polymorpha* (*SFP*)	ATGAAGATTTACGGAATTTATATGGACCGCCCGCTTTCACAGGAAGAAAATGAACGGTTCATGACTTTCATA<br>TCACCTGAAAAACGGGAGAAATGCCGGAGATTTTATCATAAAGAAGATGCTCACCGCACCCTGCTGGGAGAT<br>GTGCTCGTTCGCTCAGTCATAAGCAGGCAGTATCAGTTGGACAAATCCGATATCCGCTTTAGCACGCAGGAAT<br>ACGGGAAGCCGTGCATCCCTGATCTTCCCGACGCTCATTTCAACATTTCTCACTCCGGCCGCTGGGTCATTGGT<br>GCGTTTGATTCACAGCCGATCGGCATAGATATCGAAAAAACGAAACCGATCAGCCTTGAGATCGCCAAGCGCT<br>TCTTTTCAAAAACAGAGTACAGCGACCTTTTAGCAAAAGACAAGGACGAGCAGACAGACTATTTTTATCATC<br>TATGGTCAATGAAAGAAAGCTTTATCAAACAGGAAGGCAAAGGCTTATCGCTTCCGCTTGATTCCTTTTCAGT<br>GCGCCTGCATCAGGACGGACAAGTATCCATTGAGCTTCCGGACAGCCATTCCCCATGCTATATCAAAACGTAT<br>GAGGTCGATCCCGGCTACAAAATGGCTGTATGCGCCGCACACCCTGATTTCCCCGAGGATATCACAATGGTCT<br>CGTACGAAGAGCTTTTATAA

Supplementary Table 4: Amino acid and DNA sequences of the reductase domains screened for their capability to use C10-mACP as substrates.

**Construct**	**Sequence form start to stop codon**
pAR388 (reductase domain of CAR from *Mycobacterium Marinum*)	MAAAAARKPGSSRPTFASVHGASNGQVTEVHAGDLSLDKFIDAATLAEAPRLPAANTQVRTVLLTGATGFLGRYL<br>ALEWLERMDLVDGKLICLVRAKSDTEARARLDKTFDSGDPELLAHYRALAGDHLEVLAGDKGEADLGLDRQTWQ<br>RLADTVDLIVDPAALVNHVLPYSQLFGPNALGTAELLRLALTSKIKPYSYTSTIGVADQIPPSAFTEDADIRVISATRA<br>VDDSYANGYSNSKWAGEVLLREAHDLCGLPVAVFRCDMILADTTWAGQLNVPDMFTRMILSLAATGIAPGSFYEL<br>AADGARQRAHYDGLPVEFIAEAISTLGAQSQDGFHTYHVMNPYDDGIGLDEFVDWLNESGCPIQRIADYGDWLQR<br>FETALRALPDRQRHSSLLPLLHNYRQPERPVRGSIAPTDRFRAAVQEAKIGPDKDIPHVGAPIIVKYVSDLRLLGLLG<br>GKLHHHHHHHH
pAR388 (reductase domain of CAR from *Mycobacterium Marinum*)	ATGGCGGCCGCAGCTGCTAGAAAGCCAGGTTCTTCTAGACCAACCTTCGCTTCTGTCCACGGTGCTTCTAACGGTC<br>AAGTCACCGAAGTTCACGCTGGTGACTTGTCCTTGGACAAGTTCATCGATGCTGCTACCTTGGCTGAAGCTCCAA<br>GATTGCCAGCTGCTAACACCCAAGTTAGAACCGTTTTGTTGACCGGTGCTACCGGTTTCTTGGGTAGATACTTGG<br>CTTTGGAATGGTTGGAAAGAATGGACTTGGTCGACGGTAAGTTGATCTGTTTGGTCAGAGCTAAGTCCGACACCG<br>AAGCTAGAGCTAGATTGGACAAGACCTTCGACTCTGGTGACCCAGAATTGTTGGCTCACTACAGAGCTTTGGCTG<br>GTGACCACTTGGAAGTTTTGGCTGGTGACAAGGGTGAAGCTGACTTGGGTTTGGACAGACAAACCTGGCAAAGAT<br>TGGCTGACACCGTCGACTTGATCGTCGACCCAGCTGCTTTGGTCAACCACGTTTTGCCATACTCTCAATTGTTCGG<br>TCCAAACGCTTTGGGTACCGCTGAATTGTTGAGATTGGCTTTGACCTCCAAGATCAAGCCATACTCTTACACCTCT<br>ACCATCGGTGTCGCTGACCAAATCCCACCATCTGCTTTCACCGAAGACGCTGACATCAGAGTCATCTCTGCTACCA<br>GAGCTGTCGACGACTCTTACGCTAACGGTTACTCTAACTCTAAGTGGGCTGGTGAAGTTTTGTTGAGAGAAGCTC<br>ACGACTTGTGTGGTTTGCCAGTTGCTGTTTTCAGATGTGACATGATCTTGGCTGACACCACCTGGGCTGGTCAAT<br>TGAACGTTCCAGACATGTTCACCAGAATGATCTTGTCTTTGGCTGCTACCGGTATCGCTCCAGGTTCTTTCTACGA<br>ATTGGCTGCTGACGGTGCTAGACAAAGAGCTCACTACGACGGTTTGCCAGTCGAATTCATCGCTGAAGCTATTTC<br>TACTTTGGGTGCTCAATCTCAAGATGGTTTCCACACCTACCACGTTATGAACCCATACGACGACGGTATCGGTTT<br>GGACGAATTCGTCGACTGGTTGAACGAATCCGGTTGTCCAATCCAAAGAATCGCTGACTACGGTGACTGGTTGCA<br>AAGATTCGAAACCGCTTTGAGAGCTTTGCCAGATAGACAAAGACACTCTTCTTTGTTGCCATTGTTGCACAACTA<br>CAGACAACCAGAAAGACCAGTCAGAGGTTCTATCGCTCCAACCGATAGATTCAGAGCTGCTGTTCAAGAAGCTAA<br>GATCGGTCCAGACAAGGACATTCCACACGTCGGTGCTCCAATCATCGTTAAGTACGTCTCTGACTTGAGATTGTT<br>GGGTTTGTTGGGAGGTAAGCTTCATCATCACCACCACCACCACCAC
pAR389 (reductase domain of CAR from *Mycobacterium phlei*)	MAAATARRPGDGRPTFAGVHGDDAAEVHARDLTLDRFLDDATLAAATALPGPAPEIRTVLLTGATGFLGRYLALE<br>WLERMAMVGGTLICLVRGRDDAAARARLDQIFDSGDPELLRHYRELADRHLEVIAGDKSDAVLGLDRRTWQRLA<br>DTVDLIVDPAALVNHVLPYRELFGPNVVGTAELIRLALTGRLKPYLYTSTIAVGAGIAPGQFTEDADIRQISATRTLD<br>DSYANGYATSKWAGEVLLREAHDLCGLPVAVFRCDMILADTSYAGQLNVPDMFTRLILSLVATGIAPLSFYELDAA<br>GHRQRAHYDGLPVEFVAEAVSALGLDVAEDGGFATYHVMNPYDDGIGLDEFVDWLTGAGYPIERVNDYGTWFQR<br>FETAIRGLPERQRQASLLPLLHNYQRPQPPIRGSAAPTDRFRSAVQDAKIGPDKDIPHITPEVIVKYVTDLRLLGLLG<br>GKLHHHHHHHH
pAR389 (reductase domain of CAR from *Mycobacterium phlei*)	ATGGCGGCCGCAACCGCGCGCCGACCAGGCGACGGACGGCCCACCTTCGCCGGGGTCCACGGCGACGACGCCGCCG<br>AGGTGCACGCCCGCGATCTGACCCTGGACAGGTTCCTCGACGACGCGACGCTGGCCGCCGCGACCGCTCTGCCCGG<br>TCCGGCGCCGGAGATCCGCACGGTGCTGCTGACCGGGGCGACCGGATTCCTCGGCCGCTACCTGGCGCTGGAGTGG<br>CTGGAGCGGATGGCGATGGTCGGCGGCACCCTGATCTGCCTGGTGCGCGGCCGCGACGACGCCGCCGCCCGGGCCC<br>GGCTGGATCAGATCTTCGACTCCGGCGATCCCGAACTGCTGCGCCACTACCGGGAGTTGGCCGACCGCCATCTCGA<br>GGTCATCGCCGGCGACAAGAGCGACGCCGtCCTCGGCCTGGACCGCCGGACCTGGCAGCGGCTGGCCGATACCGTC<br>GACCTGATCGTCGACCCCGCCGCCCTGGTCAACCATGTGCTGCCGTACCGGGAGTTGTTCGGGCCCAACGTGGTCG<br>GAACCGCCGAGCTGATCCGTCTCGCGCTCACCGGCAGGCTCAAGCCGTATCTGTACACCTCGACGATCGCTGTGGG<br>CGCGGGGATTGCGCCCGGGCAGTTCACCGAGGACGCCGACATCCGGCAGATCAGCGCCACCCGGACACTCGATGAC<br>AGCTACGCCAACGGCTACGCCACCAGCAAGTGGGCCGGCGAGGTGCTGCTGCGGGAGGCACACGATCTGTGCGGCC<br>TGCCGGTGGCGGTGTTCCGGTGCGACATGATCCTGGCCGACACCAGCTACGCCGGTCAGCTCAACGTGCCGGACAT<br>GTTCACCCGGCTGATCCTGAGCCTGGTGGCCACCGGGATCGCGCCGCTGTCGTTCTACGAACTCGACGCCGCCGGG<br>CACCGGCAGCGGGCGCACTACGACGGTCTGCCGGTGGAGTTCGTCGCCGAGGCCGTCTCCGCACTCGGTCTCGACG<br>TGGCCGAGGACGGCGGCTTCGCGACCTACCACGTGATGAACCCGTACGACGACGGCATCGGGCTCGACGAGTTCGT<br>CGACTGGCTGACCGGGGCCGGcTATCCGATCGAGCggGTCAACGACTACGGCACaTGGTTTCAGCGGTTCGAGACC<br>GCGATCCGCGGCCTGCCCGAACGGCAGCGGCAGGCGTCGCTGCTTCCGCTGCTGCACAaCTACCAGCGGCCGCAGC<br>CGCCGATCCGCGGGTCGGCCGCCCCGACCGATCGGTTCCGCAGCGCGGTGCAGGACGCGAAGATCGGCCCGGACAA<br>GGACATTCCGCACATCACCCCGGAGGTGATCGTCAAGTACGTCACCGATCTGCGGCTGCTCGGGCTGCTCGGAGGT<br>AAGCTTCATCATCACCACCACCACCACCAC

pAR390 (reductase domain of CAR from *Mycobacterium smegmatis*)	MAAAAQRTAGDRRPSFTTVHGADATEIRASELTLDKFIDAETLQAAPGLPKVTTEPRTVLLSGANGWLGRFLTLQ<br>WLERLAPVGGTLITIVRGRDDAAARARLTQAYDTDPELSRRFAELADRHLRVVAGDIGDPNLGLTPEIWHRLAAE<br>VDLVVHPAALVNHVLPYRQLFGPNVVGTAEVIKLALTERIKPVTYLSTVSVAMGIPDFEEDGDIRTVSPVRPLDGGY<br>ANGYGNSKWAGEVLLREAHDLCGLPVATFRSDMILAHPRYRGQVNVPDMFTRLLLSLLITGVAPRSFYIGDGERPR<br>AHYPGLTVDFVAEAVTTLGAQQREGYVSYDVMNPHDDGISLDVFVDWLIRAGHPIDRVDDYDDWVRRFETALTA<br>LPEKRRAQTVLPLLHAFRAPQAPLRGAPEPTEVFHAAVRTAKVGPGDIPHLDEALIDKYIRDLREFGLIGGKLHHH<br>HHHHH
pAR390 (reductase domain of CAR from *Mycobacterium smegmatis*)	ATGGCGGCCGCAGCGCAGCGCACCGCGGGTGACCGCAGGCCGAGTTTCACCACCGTGCACGGCGCGGACGCCACCG<br>AGATCCGGGCGAGTGAGCTGACCCTGGACAAGTTCATCGACGCCGAAACGCTCCaGGCCGCACCGGGTCTGCCCAA<br>GGTCACCACCGAGCCACGGACGGTGTTGCTCTCGGGCGCCAACGGCTGGCTGGGCCGGTTCCTCACGTTGCAGTGG<br>CTGGAACGCCTGGCACCTGTCGGCGGCACCCTCATCACGATCGTGCGGGGCCGCGACGACGCCGCGGCCCGCGCAC<br>GGCTGACCCAGGCCTACGACACCGATCCCGAGTTGTCCCGCCGCTTCGCCGAGCTGGCCGACCGCCACCTGCGGGT<br>GGTCGCCGGTGACATCGGCGACCCGAATCTGGGCCTCACACCCGAGATCTGGCACCGGCTCGCCGCCGAGGTCGAC<br>CTGGTGGTGCATCCGGCAGCGCTGGTCAACCACGTGCTCCCCTACCGGCAGCTGTTCGGCCCCAACGTCGTGGGCA<br>CGGCCGAGGTGATCAAGCTGGCCCTCACCGAACGGATCAAGCCCGTCACGTACCTGTCCACCGTGTCGGTGGCCAT<br>GGGGATCCCCGACTTCGAGGAGGACGGCGACATCCGGACCGTGAGCCCGGTGCGCCCGCTCGACGGCGGATACGCC<br>AACGGCTACGGCAACAGCAAGTGGGCCGGCGAGGTGCTGCTGCGGGAGGCCCACGATCTGTGCGGGCTGCCCGTG<br>GCGACGTTCCGCTCGGACATGATCCTGGCGCATCCGCGCTACCGCGGTCAGGTCAACGTGCCAGACATGTTCACGC<br>GACTCCTGTTGAGCCTCTTGATCACCGGCGTCGCGCCGCGGTCGTTCTACATCGGAGACGGTGAGCGCCCGCGGGC<br>GCACTACCCCGGCCTGACGGTCGATTTCGTGGCCGAGGCGGTCACGACGCTCGGCGCGCAGCAGCGCGAGGGATAC<br>GTGTCCTACGACGTGATGAACCCGCACGACGACGGGATCTCCCTGGATGTGTTCGTGGACTGGCTGATCCGGGCG<br>GGCCATCCGATCGACCGGGTCGACGACTACGACGACTGGGTGCGTCGGTTCGAGACCGCGTTGACCGCGCTTCCCG<br>AGAAGCGCCGCGCACAGACCGTACTGCCGCTGCTGCACGCGTTCCGCGCTCCGCAGGCACCGTTGCGCGGCGCACC<br>CGAACCCACGGAGGTGTTCCACGCCGCGGTGCGCACCGCGAAGGTGGGCCCGGGAGACATCCCGCACCTCGACGAG<br>GCGCTGATCGACAAGTACATACGCGATCTGCGTGAGTTCGGTCTGATCGGAGGTAAGCTTCATCATCACCACCAC<br>CACCACCAC
pAR391 (reductase domain of CAR from *Nocardia iowensis*)	MAAAAERNSGAKRPTFTSVHGGGSEIRAADLTLDKFIDARTLAAADSIPHAPVPAQTVLLTGANGYLGRFLCLEWL<br>ERLDKTGGTLICVVRGSDAAAARKRLDSAFDSGDPGLLEHYQQLAARTLEVLAGDIGDPNLGLDDATWQRLAETV<br>DLIVHPAALVNHVLPYTQLFGPNVVGTAEIVRLAITARRKPVTYLSTVGVADQVDPAEYQEDSDVREMSAVRVVRE<br>SYANGYGNSKWAGEVLLREAHDLCGLPVAVFRSDMILAHSRYAGQLNVQDVFTRLILSLVATGIAPYSFYRTDADG<br>NRQRAHYDGLPADFTAAAITALGIQATEGFRTYDVLNPYDDGISLDEFVDWLVESGHPIQRITDYSDWFHRFETAI<br>RALPEKQRQASVLPLLDAYRNPCPAVRGAILPAKEFQAAVQTAKIGPEQDIPHLSAPLIDKYVSDLELLQLLGGKLH<br>HHHHHHH
pAR391 (reductase domain of CAR from *Nocardia iowensis*)	ATGGCGGCCGCAGCGGAACGCAACTCGGGCGCGAAGCGTCCCACCTTCACCTCGGTGCACGGCGGCGGTTCCGAGA<br>TCCGCGCCGCCGATCTGACCCTCGACAAGTTCATCGATGCCCGCACCCTGGCCGCCGCCGACAGCATTCCGCACGC<br>GCCGGTGCCAGCGCAGACGGTGCTGCTGACCGGCGCGAACGGCTACCTCGGCCGGTTCCTGTGCCTGGAATGGCTG<br>GAGCGGCTGGACAAGACGGGTGGCACGCTGATCTGCGTCGTGCGCGGTAGTGACGCGGCCGCGGCCCGTAAACGG<br>CTGGACTCGGCGTTCGACAGCGGCGATCCCGGCCTGCTCGAGCACTACCAGCAACTGGCCGCACGGACCCTGGAAG<br>TCCTCGCCGGTGATATCGGCGACCCGAATCTCGGTCTGGACGACGCGACTTGGCAGCGGTTGGCCGAAACCGTCGA<br>CCTGATCGTCCATCCCGCCGCGTTGGTCAACCACGTCCTTCCCTACACCCAGCTGTTCGGCCCCAATGTCGTCGGC<br>ACCGCCGAAATCGTCCGGTTGGCGATCACGGCGCGGCGCAAGCCGGTCACCTACCTGTCGACCGTCGGAGTGGCCG<br>ACCAGGTCGACCCGGCGGAGTATCAGGAGGACAGCGACGTCCGCGAGATGAGCGCGGTGCGCGTCGTGCGCGAGA<br>GTTACGCCAACGGCTACGGCAACAGCAAGTGGGCGGGGGAGGTCCTGCTGCGCGAAGCACACGATCTGTGTGGCT<br>TGCCGGTCGCGGTGTTCCGTTCGGACATGATCCTGGCGCACAGCCGGTACGCGGGTCAGCTCAACGTCCAGGACGT<br>GTTCACCCGGCTGATCCTCAGCCTGGTCGCCACCGGCATCGCGCCGTACTCGTTCTACCGAACCGACGCGGACGGC<br>AACCGGCAGCGGGCCCACTATGACGGCTTGCCGGCGGACTTCACGGCGGCGGCGATCACCGCGCTCGGCATCCAAG<br>CCACCGAAGGCTTCCGGACCTACGACGTGCTCAATCCGTACGACGATGGCATCTCCCTCGATGAATTCGTCGACTG<br>GCTCGTCGAATCCGGCCACCCGATCCAGCGCATCACCGACTACAGCGACTGGTTCCACCGTTTCGAGACGGCGATC<br>CGCGCGCTGCCGGAAAAGCAACGCCAGGCCTCGGTGCTGCCGTTGCTGGACGCCTACCGCAACCCCTGCCCGGCGG<br>TCCGCGGCGCGATACTCCCGGCCAAGGAGTTCCAAGCGGCGGTGCAAACAGCCAAAATCGGTCCGGAACAGGACA<br>TCCCGCATTTGTCCGCGCCACTGATCGATAAGTACGTCAGCGATCTGGAACTGCTTCAGCTGCTCGGAGGTAAGCT<br>TCATCATCACCACCACCACCACCAC
pAR392 (reductase domain of CAR from *Nocardia otitidiscaviarum*)	MAAASERDSGSRPTAATVHGDDGLLRADDLALEAFLDPATLDAAAHLPSALEPPRTVLLTGANGYLGRFLALEWL<br>QRLDVSGGTLICLIRGSDADSARRRLDAVFATGDPELEAHYRELAERRLRVLPGDIGEPNLGLREQDWRDLAETVD<br>LIVHPAALVNHVLPYAQLFGPNVVGTAEVIRLALTSRLKPVTYLSTVAVSAGIDPETFTEDGDIREISPVRRLDDGYA<br>NGYGNSKWAGEVLLRNAHDRFGLPVAVFRSDMILAHSRYAGQLNVPDMFTRLLLSVLATGLAPGSFHDAHGERH<br>RAHYDGLPADFTAAAVTTLGSRVTSGYETYDVLNPHDDGISLDTFVDWLIEAGHPIDRIDDYAEWFARFDTALRAL<br>PEHQRQHSLLPLLHAYRRPTPPLHGVALPAKHFRAAVQQAKLGPDGDIPHVTRELIEKYASDLRLLGLIQGGGKLH<br>HHHHHHH

pAR392 (reductase domain of CAR from *Nocardia otitidiscaviarum*)	ATGGCGGCCGCAAGCGAACGCGATTCCGGCAGTCGTCCGACCGCCGCCACGGTGCACGGCGACGACGGCCTGCTGC<br>GGGCCGACGATCTCGCCCTGGAGGCATTCCTCGACCCGGCGACCCTGGACGCGGCCGCGCATCTGCCGAGCGCGCT<br>GGAGCCGCCGCGCACGGTTCTGCTGACCGGCGCCAACGGCTACCTCGGCCGCTTCCTCGCCCTGGAATGGCTACAG<br>CGCCTGGACGTCTCGGGCGGTACTCTGATCTGCCTGATCCGCGGCAGTGACGCCGACAGTGCTCGGCGCCGTCTCG<br>ACGCGGTCTTCGCCACCGGTGATCCGGAGCTGGAAGCCCACTACCGCGAACTCGCCGAGCGGCGGCTGCGTGTCCT<br>GCCCGGCGATATCGGTGAACCGAATCTCGGCCTGCGCGAACAGGATTGGCGCGATCTGGCCGAGACCGTGGACCT<br>GATCGTGCATCCGGCCGCCCTGGTCAACCATGTGCTGCCCTACGCCCAGTTGTTCGGCCCCAATGTTGTCGGTACC<br>GCCGAGGTGATCCGTCTCGCGCTCACCTCCCGGCTGAAGCCGGTCACCTACCTGTCCACCGTGGCGGTATCCGCCG<br>GTATCGACCCGGAGACCTTCACCGAGGACGGCGATATCCGCGAGATCAGCCCCGTGCGCCGGCTCGACGACGGCTA<br>CGCCAATGGCTACGGCAACAGCAAATGGGCCGGCGAGGTGCTGTTGCGCAATGCGCACGACCGCTTCGGTCTCCCG<br>GTGGCGGTCTTCCGCTCCGACATGATCCTGGCCCACAGCCGCTACGCCGGACAGCTCAACGTGCCCGACATGTTCA<br>CCCGCCTGCTGCTGAGCGTGCTGGCGACCGGCCTGGCCCCCGGCTCCTTCCACGATGCCCACGGCGAGCGCCACCGC<br>GCCCACTACGACGGTCTCCCAGCCGATTTCACCGCCGCGGCCGTCACCACCCTGGGCAGCCGGGTGACCAGTGGTT<br>ACGAGACCTACGACGTGCTCAACCCGCACGACGACGGGATCTCCCTGGACACCTTCGTCGACTGGCTCATCGAGGC<br>GGGCCACCCCATCGACCGCATCGACGATTACGCCGAGTGGTTCGCCCGCTTCGACACCGCCCTGCGCGCCCTCCCCG<br>AACACCAACGCCAACACTCGCTGCTACCCCTGCTGCACGCCTACCGTCGCCCCACCCCTCCCCTGCACGGAGTCGCC<br>CTCCCCGCCAAGCACTTCCGCGCCGCCGTACAGCAGGCGAAACTCGGCCCGGACGGGGATATCCCGCACGTGACGC<br>GGGAGCTGATCGAGAAGTACGCGAGTGATCTGCGGTTACTGGGGTTGATTCAGGGCGGAGGTAAGCTTCATCATC<br>ACCACCACCACCACCAC
pAR393 (reductase domain of CAR from *Tsukamurella paurometabola*)	MAAAKARSGGVAAPTADSVHGVGASVARATDLTLEKFIDPELLALAPTLPAATGEPNTVLLTGSTGYLGRFLLLDW<br>LRRVAPHGGTVIALVRGADADDARRRVTAAIGDSDPDLTQEFTSLAEHHLHVIAGDFGSPALGLDDATWSDLAGR<br>VDHVVHCGALVNHVLPYDQLFGPNVVATGEVVRLALTTRRKSVDYVSTVAVVPQDDGRVLVEDDDVRELGAERRI<br>GADAYANGYAVSKWAGEVLLHEAADLADLPVRVFRSDMILAHSRFHGQFNEVDQFTRLLLSIAETGLAPASFYTPD<br>PSGHRPHYDGLPVDFTAEAITTLSAAGRSGYRTFHVLNANDDGVSLDSFVDWIAASGRSIERIDDYDTWFARFEQA<br>LQQLPDEARQRSVLPLLHAVREPAPAAGTSALSVDRFRGAVRETGVGPGDIPVLDRALIEKYLRDFETAGWLAPGA<br>RDGGKLHHHHHHHH
pAR393 (reductase domain of CAR from *Tsukamurella paurometabola*)	ATGGCGGCCGCAAAGGCTCGATCGGGTGGCGTCGCGGCACCGACCGCCGACTCGGTGCACGGCGTGGGTGCGAGCG<br>TCGCCCGGGCCACCGACCTGACGCTGGAGAAGTTCATCGACCCCGAGCTCCTCGCGCTCGCGCCGACGCTTCCCGC<br>GGCGACCGGTGAGCCGAACACCGTGCTGCTCACCGGATCCACCGGCTACCTCGGCCGCTTCCTGCTGCTGGACTGG<br>TTGCGACGGGTCGCTCCGCACGGCGGCACCGTGATCGCGCTGGTGCGCGGCGCCGACGCCGACGATGCGCGACGCC<br>GCGTCACGGCCGCGATCGGTGACTCGGATCCTGACCTGACACAAGAGTTCACGTCACTCGCGGAGCATCACCTCCA<br>CGTGATCGCCGGTGACTTCGGCAGCCCCGCACTCGGACTCGACGATGCCACCTGGAGCGATCTCGCCGGGCGAGTC<br>GATCACGTGGTGCACTGCGGCGCGCTCGTCAACCACGTGCTGCCCTACGACCAACTGTTCGGTCCCAATGTGGTGG<br>CCACCGGCGAAGTGGTGCGACTCGCACTCACCACGCGCCGCAAGTCCGTGGATTACGTCTCCACGGTGGCTGTGGT<br>TCCGCAGGATGACGGCCGCGTCCTGGTCGAGGACGACGATGTTCGCGAGCTCGGCGCCGAACGGCGCATCGGGGCC<br>GATGCCTACGCGAACGGCTACGCCGTGAGCAAATGGGCGGGCGAAGTGCTGTTGCATGAGGCAGCCGACCTGGCG<br>GACCTGCCGGTGCGGGTGTTCCGCTCCGATATGATCTTGGCGCACAGTCGATTCCACGGACAGTTCAACGAGGTCG<br>ACCAGTTCACCCGCCTGCTCCTGAGTATCGCCGAGACCGGACTGGCGCCGGCGTCGTTCTACACGCCGGATCCGAG<br>TGGACACCGCCCGCACTACGACGGGCTGCCGGTGGACTTCACCGCCGAAGCGATCACCACGCTCAGCGCCGCGGGG<br>CGTTCGGGGTACCGGACCTTCCACGTGCTCAACGCCAACGATGACGGCGTGAGCCTGGACAGCTTCGTCGACTGGA<br>TCGCCGCCTCGGGCCGGAGCATCGAACGGATCGACGACTACGACACCTGGTTCGCCCGGTTCGAGCAGGCGCTCCA<br>GCAGCTCCCCGATGAGGCGCGCCAGCGGTCGGTGCTGCCCCTGCTGCACGCGGTGCGCGAGCCGGCTCCGGCCGCC<br>GGGACCTCCGCGCTGTCGGTGGACCGGTTCCGTGGTGCGGTGCGTGAGACCGGAGTAGGACCGGGGGACATCCCG<br>GTGCTCGATCGCGCCCTGATCGAGAAGTACCTGCGCGACTTCGAGACCGCGGGCTGGCTCGCGCCCGGTGCGCGCG<br>ACGGAGGTAAGCTTCATCATCACCACCACCACCACCAC
pMMH12 (reductase domain of CAR from *Neurospora crassa*)	MAAQGTLDNEHHVMEALVEKYTRDLPTPKQNKPAPADEGQVVVITGTTGGIGSYLIDICSSSSRVSKIICLNRSEDG<br>KARQTASSSGRGLSTDFSKCEFYHADMSRADLGLGPEVYSRLLSEVDRVIHNQWPVNFNIAVESFEPHIRGCRNLV<br>DFSYKADKNVPIVFVSSIGTVDRWHDEDRIVPEASLDDLSLAAGGYGQSKLVSSLIFDKAAEVSGVPTEVVRVGQVA<br>GPSSEKGYWNKQEWLPSIVASSAYLGVLPDSLGQMTTIDWTPIEAIAKLLLEVSGVIDNVPLDKINGYFHGVNPERT<br>SWSALAPAVQEYYGDRIQKIVPLDEWLEALEKSQEKAEDVTRNPGIKLIDTYRTWSEGYKKGTKFVPLDMTRTKE<br>YSKTMREMHAVTPELMKNWCRQWNFKLHHHHHHHH
pMMH12 (reductase domain of CAR from *Neurospora crassa*)	ATGGCGGCCCAGGGCACCCTGGACAATGAACATCACGTGATGGAAGCACTGGTTGAAAAATATACCCGTGATCTG<br>CCGACGCCGAAACAGAACAAACCGGCTCCGGCGGACGAAGGTCAAGTCGTGGTTATTACCGGTACCACGGGTGGC<br>ATCGGCTCCTACCTGATTGATATCTGCTCCTCATCGAGCCGCGTGTCCAAAATTATCTGTCTGAATCGTTCAGAAG<br>ATGGCAAAGCGCGCCAGACGGCCTCTAGTTCCGGTCGTGGCCTGAGCACCGACTTTTCTAAATGCGAATTCTATCA<br>TGCAGATATGAGCCGCGCTGACCTGGGTCTGGGTCCGGAAGTGTACAGTCGTCTGCTGTCCGAAGTGGATCGCGT<br>TATTCATAACCAGTGGCCGGTCAACTTTAATATTGCGGTGGAATCTTTCGAACCGCACATCCGTGGTTGTCGCAA<br>TCTGGTGGATTTTAGTTATAAAGCCGACAAAAACGTTCCGATTGTTTTCGTCTCATCGATCGGCACCGTCGATCG<br>TTGGCACGATGAAGACCGCATTGTGCCGGAAGCGTCACTGGATGACCTGTCGCTGGCCGCCGGTGGCTACGGCCA<br>GTCCAAACTGGTGAGCTCTCTGATCTTTGATAAAGCAGCTGAAGTCTCAGGCGTGCCGACCGAAGTCGTGCGTGT<br>TGGTCAGGTTGCGGGTCCGAGTTCCGAAAAAGGTTATTGGAATAAACAAGAATGGCTGCCGTCGATTGTCGCATC

	ATCGGCTTACCTGGGTGTGCTGCCGGATAGCCTGGGCCAGATGACCACGATCGACTGGACGCCGATTGAAGCCAT<br>CGCAAAACTGCTGCTGGAAGTGTCTGGCGTTATTGATAACGTGCCGCTGGACAAAATCAATGGTTATTTTCATGG<br>CGTTAACCCGGAACGTACCAGTTGGAGCGCTCTGGCCCCGGCAGTCCAGGAATATTACGGTGATCGCATTCAAAA<br>AATCGTTCCGCTGGACGAATGGCTGGAAGCGCTGGAAAAATCTCAGGAAAAAGCCGAAGATGTGACCCGTAACCC<br>GGGCATTAAACTGATCGACACCTATCGCACGTGGTCTGAAGGTTACAAGAAAGGCACGAAATTTGTTCCGCTGGA<br>TATGACCCGCACGAAAGAATATAGTAAAACCATGCGTGAAATGCACGCTGTGACGCCGGAACTGATGAAAAACTG<br>GTGTCGCCAATGGAACTTTAAGCTTCATCATCACCACCACCACCACCAC
pAR394 (reductase domain of PKS/NRPS from *Stigmatella aurantiaca*)	MAAAAAKTGSLPAHDVTVEMEADAVLDAEIALGKALPPVTGALRTILLTGATGFLGAFLLEELCRRTDARIYCLVR<br>SKTEQEGMNRIRKNLESYSLWNEALAPRIVPVRGDIGQPLLGLSEKEFQRLSEEIDAIYHNGALVNFLYPYESMRAA<br>NVLGTREILRLATRTRIKPLHYVSTVSVLPLGRKAPIREDEPLEGPSSLVGGYAQSKWVAEKLVREASRRGLPVTILR<br>PGRVTGHSRTGAWNTDDLVCRTLKGCVRMGVAPSVDALLDLTPVDYVSSAIVDLSMRPESIGQTYHLVNPQFVRA<br>DEMWNYMRAFGYGLRVLPYDQWLSELGSAASSDSELGDLLMFLQQVPPEDRSVGGPRMVVCDSGDTLKALGGTG<br>TSCPSVDASLISTYLSSLVHRGFLKAPEVRGGKLHHHHHHHH
pAR394 (reductase domain of PKS/NRPS from *Stigmatella aurantiaca*)	ATGGCGGCCGCAGCCGCGAAGACAGGCTCGCTGCCAGCCCACGATGTGACGGTGGAGATGGAAGCAGATGCTGTG<br>CTGGATGCCGAGATCGCGCTGGGCAAGGCACTTCCGCCCGTGACGGGGGCTTTGCGCACCATCCTGCTGACCGGCG<br>CCACGGGCTTCCTCGGTGCTTTCTTGCTGGAGGAACTCTGCCGCAGGACGGACGCGCGCATTTATTGCTTGGTGCG<br>CTCCAAGACAGAGCAGGAGGGGATGAACAGGATTCGCAAGAACCTGGAGAGCTACTCCCTGTGGAACGAGGCTCT<br>GGCGCCTCGCATCGTTCCTGTCCGAGGAGATATTGGCCAACCGTTGTTGGGACTCTCGGAGAAGGAGTTCCAGCG<br>GCTCTCGGAAGAGATTGACGCCATTTATCACAATGGCGCGCTCGTCAATTTCCTCTACCCCTACGAGTCCATGCGA<br>GCGGCCAACGTGCTTGGCACGCGGGAGATCCTCCGGCTGGCTACGCGGACGCGCATCAAACCGTTGCACTACGTTT<br>CGACTGTTTCGGTATTGCCCCTGGGACGAAAGGCTCCGATCCGCGAGGATGAGCCTTTGGAAGGGCCCTCAAGTT<br>TGGTGGGAGGCTACGCACAGAGCAAGTGGGTTGCCGAGAAGCTTGTGAGAGAGGCTTCTCGGCGGGGGCTCCCGG<br>TGACCATCCTCCGGCCGGGACGGGTGACCGGCCACAGCCGCACGGGGGCCTGGAACACGGACGATCTGGTGTGCAG<br>GACCCTCAAGGGGTGCGTCCGGATGGGAGTGGCTCCCAGCGTCGATGCGCTGCTTGACCTGACGCCCGTCGATTAC<br>GTCAGCAGTGCCATCGTGGATCTGTCCATGCGCCCGGAGTCGATTGGCCAGACGTATCATCTCGTCAATCCGCAGT<br>TCGTGCGCGCTGATGAGATGTGGAATTACATGCGAGCCTTTGGGTACGGATTGCGAGTCCTCCCGTATGACCAAT<br>GGCTTTCGGAATTGGGCTCCGCGGCATCGTCCGACAGCGAGCTGGGCGATCTTCTCATGTTCCTCCAGCAGGTTCC<br>TCCAGAGGACCGGAGCGTTGGCGGACCGCGGATGGTGGTTTGCGACAGTGGCGACACGCTGAAGGCCTTGGGAGG<br>AACGGGAACGTCCTGCCCGTCGGTGGACGCCTCTTTGATTTCAACCTACCTCTCGTCGCTCGTTCACCGCGGCTTT<br>CTCAAGGCACCCGAGGTGAGGGGAGGTAAGCTTCATCATCACCACCACCACCACCAC
pAR395 (reductase domain of PKS/NRPS from *Mycobacterium tuberculosis*)	MAAARDARPTSDPRLVSVHGDNPTEVHASDLTLDRFIDADTLATAVNLPGPSPELRTVLLTGATGFLGRYLVLELL<br>RRLDVDGRLICLVRAESDEDARRRLEKTFDSGDPELLRHFKELAADRLEVVAGDKSEPDLGLDQPMWRRLAETVD<br>LIVDSAAMVNAFPYHELFGPNVAGTAELIRIALTTKLKPFTYVSTADVGAAIEPSAFTEDADIRVISPTRTVDGGWA<br>GGYGTSKWAGEVLLREANDLCALPVAVFRCGMILADTSYAGQLNMSDWVTRMVLSLMATGIAPRSFYEPDSEGN<br>RQRAHFDGLPVTFVAEAIAVLGARVAGSSLAGFATYHVMNPHDDGIGLDEYVDWLIEAGYPIRRIDDFAEWLQRFE<br>ASLGALPDRQRRHSVLPMLLASNSQRLQPLKPTRGCSAPTDRFRAAVRAAKVGSDKDNPDIPHVSAPTIINYVTNL<br>QLLGLLGGKLHHHHHHHH
pAR395 (reductase domain of PKS/NRPS from *Mycobacterium tuberculosis*)	ATGGCGGCCGCACGAGATGCCCGACCGACCAGCGATCCGCGCTTGGTGTCTGTGCACGGCGACAACCCCACCGAGG<br>TGCATGCCAGCGACCTCACGCTGGACCGGTTCATCGACGCCGACACGCTGGCCACCGCCGTCAACCTGCCGGGCCC<br>GAGCCCCGAGCTACGGACGGTCCTGCTGACGGGCGCGACGGGTTTCCTCGGACGGTATCTGGTCCTTGAATTGCTG<br>CGGCGGCTGGACGTCGACGGCAGGCTGATCTGTTTGGTGCGGGCGGAGTCCGACGAGGATGCGCGGCGTCGTCTG<br>GAGAAGACCTTCGATAGCGGTGACCCGGAATTGCTGCGGCACTTCAAGGAGCTTGCCGCCGACCGGCTGGAGGTC<br>GTCGCAGGCGACAAGAGCGAACCCGACCTGGGCCTGGACCAACCGATGTGGCGGCGGCTGGCCGAAACCGTGGAT<br>TTGATTGTCGATTCCGCGGCGATGGTCAACGCGTTTCCCTACCACGAATTGTTCGGGCCCAACGTCGCGGGCACCG<br>CCGAGCTGATCCGAATCGCGCTTACCACCAAGCTCAAACCCTTCACCTACGTGTCAACCGCCGACGTGGGTGCTGC<br>GATCGAGCCGTCGGCGTTCACCGAGGACGCCGACATCCGGGTAATCAGCCCCACCCGCACCGTCGACGGCGGCTGG<br>GCTGGCGGCTACGGCACCAGCAAGTGGGCCGGTGAGGTGCTGCTGCGCGAGGCCAACGACCTGTGCGCGCTGCCGG<br>TCGCGGTGTTTCGCTGCGGGATGATCCTGGCCGACACCAGCTATGCCGGACAGCTCAACATGTCGGACTGGGTCAC<br>CCGGATGGTGTTGAGCTTGATGGCTACCGGCATCGCGCCTCGTTCGTTCTACGAACCGGACTCCGAGGGCAATCGG<br>CAACGCGCGCACTTCGACGGGCTGCCAGTCACCTTCGTTGCCGAGGCGATCGCGGTGCTGGGCGCGCGGGTGGCCG<br>GCTCATCGTTGGCGGGATTTGCGACCTATCACGTGATGAACCCGCACGACGACGGTATCGGGCTCGATGAGTATG<br>TGGACTGGCTGATTGAGGCCGGCTACCCGATACGCCGCATCGATGACTTTGCGGAGTGGTTGCAGCGGTTTGAGG<br>CCAGCCTGGGCGCTCTGCCGGATCGGCAACGCCGGCACTCGGTGCTGCCGATGCTGCTGGCGAGCAATTCCCAGCG<br>ATTGCAGCCGCTTAAGCCGACCAGGGGGTGCTCCGCGCCGACCGACCGATTCCGTGCCGCGGTGCGAGCGGCGAAA<br>GTCGGCTCCGACAAGGACAATCCAGACATCCCGCACGTGTCGGCGCCGACCATCATCAACTACGTCACCAACCTAC<br>AACTGCTCGGACTGCTGGGAGGTAAGCTTCATCATCACCACCACCACCACCAC

pAR396 (reductase domain of PKS/NRPS from *Mycobacterium smegmatis*)	MAAAHDAGERTDRVSFAAVHGSDVTEVHARDLTLDKFIDAPTLRTATTLPRPDGAVQTVLLTGATGFLGRYLLLE<br>WLRQLRRVDDKVICLVRGKSDEDARRRLEATFDTDPLLRKHFNELATERLQVVAGDKGQPNLGLDEQTWQRLAE<br>SVDLIVDSAAFVNSVLPYSELFGPNVVGTAELIRFALTSKLKPFNFVSTSDVGRQIEPSRFTEQADIRLVSATRKIEVG<br>YANGYGNSKWAGEVLLREAHDHCGLPVAVFRSGMIMVDPTYAGQLNVTDTVSRMVLSIVATGVAPGSFYQRGDN<br>GERQRAHFDGLPVDFVAQAITKLGWQVARSVTDSTASGFETYHVMNPHDDGIGIDTYIDWLIEAGYPIERIEDFGE<br>WLQRFEAALQGLSDQQRQNSVLQMLTLLKQQAGELQPPVPTRGSFAPADRFQAAVRDANIGVEGEIPHVTREVIV<br>KYVTDLQLLGLLGGKLHHHHHHHH
pAR396 (reductase domain of PKS/NRPS from *Mycobacterium smegmatis*)	ATGGCGGCCGCACACGACGCCGGTGAGCGGACCGACAGGGTCAGCTTCGCAGCGGTGCACGGCAGCGACGTCACCG<br>AGGTGCATGCGCGCGATCTGACACTGGACAAGTTCATCGACGCTCCGACACTGCGGACCGCGACGACCCTGCCCCG<br>ACCCGACGGCGCCGTGCAGACGGTCCTCCTGACCGGGGCCACCGGATTCCTAGGGCGCTACCTGCTGCTGGAATGG<br>CTGAGGCAGTTGCGGCGTGTCGACGACAAGGTGATCTGCCTGGTGCGCGGAAAGTCCGACGAGGACGCCCGCAGG<br>CGCCTCGAGGCCACTTTCGACACAGATCCGTTGCTACGCAAGCATTTCAACGAACTGGCCACCGAGCGCCTGCAGG<br>TCGTCGCGGGCGACAAGGGACAACCGAACCTCGGGCTCGACGAACAGACCTGGCAGCGGCTGGCCGAAAGTGTCG<br>ACCTGATCGTCGATTCGGCGGCATTCGTCAACAGTGTGCTGCCCTACAGCGAACTGTTCGGACCCAACGTGGTGGG<br>CACGGCCGAGCTGATCCGGTTCGCGCTGACCTCGAAACTCAAGCCGTTCAACTTCGTGTCGACGTCGGACGTCGGC<br>AGGCAGATCGAGCCGTCGCGGTTCACCGAGCAGGCCGACATCCGGTTGGTCAGCGCGACCCGCAAGATCGAGGTC<br>GGGTATGCCAACGGCTACGGCAACAGCAAGTGGGCAGGCGAGGTGCTGCTGCGCGAGGCCCACGACCACTGCGGC<br>CTGCCGGTGGCGGTGTTCCGGTCGGGCATGATCATGGTCGACCCGACCTACGCCGGGCAGCTGAACGTCACGGACA<br>CGGTGTCGCGCATGGTGCTCAGCATCGTGGCCACCGGTGTCGCACCCGGGTCGTTCTATCAGCGCGGTGACAACGG<br>TGAGCGGCAGCGGGCACACTTCGACGGCTTGCCCGTCGACTTCGTCGCCCAGGCCATCACCAAGCTCGGCTGGCAG<br>GTCGCGAGGTCGGTGACCGATTCCACCGCAAGCGGTTTCGAGACTTATCACGTGATGAACCCGCATGACGACGGC<br>ATCGGCATCGACACCTACATCGACTGGCTCATCGAGGCCGGATACCCGATCGAACGCATCGAGGACTTCGGAGAG<br>TGGTTGCAGCGGTTCGAAGCGGCCCTGCAGGGGCTGTCGGACCAACAGCGGCAGAACTCCGTGCTGCAGATGCTG<br>ACGTTGCTCAAGCAGCAGGCGGGGGAGCTGCAGCCACCAGTGCCCACCCGGGGCTCGTTTGCGCCGGCAGACCGGT<br>TCCAGGCCGCGGTCCGCGATGCGAACATCGGTGTGGAAGGCGAGATTCCGCATGTCACGCGGGAGGTCATCGTCA<br>AGTACGTGACCGATCTGCAGCTGCTCGGGCTGCTGGGAGGTAAGCTTCATCATCACCACCACCACCACCAC
pMMH05 (reductase domain of NRPS from *Methanobrevibacter ruminantium*)	MAADIGEEDLDLTDDIIKNYNYGEINELLQENTWENFFDGENLELGNVLLTGATGFLGIHILYEFIKSEEGKIYCMLR<br>KGKFDSCQERLIDVMNDYFDEDFTDLVGSRIIPIEGDITEIDDFKQLEDEPIDTVINSAALVKHYTADDYIFRVNVDG<br>VINGLKFAQTRNNIKYVQISTISVLSSYSLNEEAYPNQEYDERTLYYEQDLENKYVCSKFLAERAVLQAATKGLPVKI<br>IRVGNLMSRYSDGVFQKNYDTNAFLNNIKTIKKLGAMNPAMASEKVDMSQIDYVAKGILALSKTPEKSRVFHCMN<br>NHYISHRDIVDALNTYGYGIEEVDFEEFKQIYEQNMNENIQGIITADFSIDDFDEEDDFEENVEIEQTVDILHSLGFD<br>WPEADEEYLKRLFDYLNKFDYFEKLHHHHHHHH
pMMH05 (reductase domain of NRPS from *Methanobrevibacter ruminantium*)	ATGGCGGCCGACATTGGCGAAGAGGACTTGGATTTAACTGATGATATCATCAAAAACTACAATTATGGCGAAATC<br>AATGAGCTTCTCCAAGAGAATACATGGGAAAACTTCTTTGATGGTGAAAATCTGGAATTGGGCAATGTCCTATTG<br>ACTGGAGCTACAGGATTTTTAGGAATCCATATCCTATATGAATTCATCAAAAGTGAGGAAGGAAAGATCTATTG<br>TATGTTAAGGAAAGGCAAGTTTGATTCCTGTCAGGAACGGTTGATTGATGTGATGAATGATTACTTTGATGAGG<br>ATTTCACTGATCTCGTCGGCTCACGCATCATTCCAATCGAAGGGGACATAACTGAAATCGATGATTTTAAACAGC<br>TTGAGGATGAGCCTATTGATACGGTGATAAATTCGGCCGCACTTGTAAAGCATTATACTGCCGATGATTATATCT<br>TCAGGGTGAATGTCGATGGAGTAATCAATGGACTTAAATTTGCCCAAACTAGAAATAACATAAAATATGTACAG<br>ATATCAACAATCAGCGTTCTTTCATCATACTCCTTAAATGAAGAGGCATATCCGAATCAGGAATATGATGAAAGG<br>ACATTATATTATGAGCAGGATCTAGAAAACAAATATGTATGCAGTAAGTTTTTAGCTGAAAGGGCGGTTCTGCA<br>AGCTGCAACTAAAGGGCTTCCAGTAAAGATAATCAGAGTTGGAAATCTTATGAGCCGTTATTCAGATGGTGTGTT<br>CCAGAAAAATTATGATACAAATGCATTTTTAAATAACATTAAAACCATTAAAAAATTAGGAGCTATGAATCCTG<br>CAATGGCTAGTGAAAAGGTTGACATGAGTCAAATAGATTATGTTGCAAAGGGAATACTTGCATTGTCTAAGACT<br>CCAGAGAAATCCAGGGTATTCCATTGTATGAATAATCATTATATTTCCCATAGGGATATTGTTGATGCATTGAAT<br>ACATACGGTTACGGAATTGAAGAAGTGGATTTCGAAGAATTCAAACAGATTTACGAACAAAACATGAATGAGAA<br>TATACAGGGTATAATAACTGCCGATTTTTCAATTGATGACTTTGATGAGGAAGACGATTTCGAGGAGAATGTGG<br>AAATTGAACAGACTGTTGATATTCTTCATTCGTTAGGATTTGATTGGCCTGAAGCAGATGAAGAGTATCTTAAAC<br>GATTGTTTGATTATTTGAATAAGTTTGATTATTTTGAAAAGCTTCATCATCACCACCACCACCACCAC

Supplementary Table 5: Amino acid and DNA sequences of the constructs used in the fatty aldehydes/alcohol experiments. Mutated positions and the performed mutations are highlighted.

**Construct**	**Sequence form start to stop codon**
pMMH01 (mouseFAS_ΔTE _CAR-TR_Mphi, Strep-and His-tagged)<br>Position 113 highlighted	MSAWSHPQFEKGGGSGGGSGGSAWSHPQFEKGAGSEEVVIAGMSGKLPESENLQEFWANLIGGVDMVTDDDR<br>RWKAGLYGLPKRSGKLKDLSKFDASFFGVHPKQAHTMDPQLRLLLEVSYEAIVDGGINPASLRGTNTGVWVGV<br>S**G/W**SEASEALSRDPETLLGYSMVGCQRAMMANRLSFFFDFKGPSIALDTACSSSLLALQNAYQAIRSGECPAA<br>LVGGINLLLKPNTSVQFMKLGMLSPDGTCRSFDDSGSGYCRSEAVVAVLLTKKSLARRVYATILNAGTNTDGSK<br>EQGVTFPSGEVQEQLICSLYQPAGLAPESLEYIEAHGTGTKVGDPQELNGITRSLCAFRQAPLLIGSTKSNMGHP<br>EPASGLAALTKVLLSLEHGVWAPNLHFHNPNPEIPALLDGRLQVVDRPLPVRGGNVGINSFGFGGSNVHVILQP<br>NTRQAPAPTAHAALPHLLHASGRTLEAVQDLLEQGRQHSQDLAFVSMLNDIAATPTAAMPFRGYTVLGVEGR<br>VQEVQQVSTNKRPLWFICSGMGTQWRGMGLSLMRLDSFRESILRSDEAVKPLGVKVSDLLLSTDERTFDDIVH<br>AFVSLTAIQIALIDLLTSVGLKPDGIIGHSLGEVACGYADGCLSQREAVLAAYWRGQCIKDAHLPPGSMAAVGLS<br>WEECKQRCPAGVVPACHNSEDTVTISGPQAAVNEFVEQLKQEGVFAKEVRTGGLAFHSYFMEGIAPTLLQALK<br>KVIREPRPRSARWLSTSIPEAQWQSSLARTSSAEYNVNNLVSPVLFQEALWHIPEHAVVLEIAPHALLQAVLKR<br>GVKSSCTIIPLMKRDHKDNLEFFLTNLGKVHLTGINVNPNALFPPVEFPAPRGTPLISPHIKWDHSQTWDVPVA<br>EDFPNGSSSSSATVYSIDASPESPDHYLVDHCIDGRVIFPGTGYLCLVWKTLARSLGLSLEETPVVFENVSFHQAT<br>ILPKTGTVALEVRLLEASHAFEVSDTGNLIVSGKVYLWEDPNSKLFDHPEVPTPPESASVSRLTQGEVYKELRLR<br>GYDYGPQFQGICEATLEGEQGKLLWKDNWVTFMDTMLQVSILGSSQQSLQLPTRVTAIYIDPATHRQKVYRLK<br>EDTQVADVTTSRCLGITVSGGIHISRLQTTATSRRQQEQLVPTLEKFVFTPHMEAECLSESTALQKELQLCKGLA<br>RALQTKATQQGLKAAMLGQEDPPQHGLPRLLAAACQLQLNGNLQLELGEALAQERLLLPEDPLISGLLNSQAL<br>KACVDTALENLSTLKMKVAEVLAGEGHLYSRIPALLNTQPMLQLEYTATDRHPQALKDVQTKLQQHDVAQGQ<br>WNPSDPAPSSLGALDLLVCNCALATLGDPALALDNMVAALKEGGFLLVHTVLKGHALGETLACLPSEVQPAPS<br>LLSQEEWESLFSRKALHLVGLKRSFYGTALFLCRRAIPQEKPIFLSVEDTSFQWVDSLKSTLATSSSQPVWLTA<br>MDCPTSGVVGLVNCLRKEPGGHRIRCILLSNLSNTSHAPKLDPGSPELQQVLKHDLVMNVYRDGAWGAFRHF<br>QLEQDKPKEQTAHAFVNVLTRGDLASIRWVSSPLKHTQPSSSGAQLCTVYYASLNFRDIMLATGKLSPDAIPGK<br>WASRDCMLGMEFSGRDRCGRRVMGLVPAEGLATSVLLSSDFLWDVPSSWTLEEAASVPVVYTTAYYSLVVRG<br>RIQRGETVLIHSGSGGVGQAAISIALSLGCRVFTTVGSAEKRAYLQARFPQLDDTSFANSRDTSFEQHVLLHTGG<br>KGVDLVLNSLAEEKLQASVRCLAQHGRFLEIGKFDLSNNHPLGMAIFLKNVTFHGILLDALFEEANDSWREVA<br>ALLKAGIRDGVVKPLKCTVFPKAQVEDAFRYMAQGKHIGKVLVQVREEEPEAVLPGAQPTLISAISKTFCPAHK<br>SYIITGGLGGFGLELARWLVLRGAQRLVLTSRSGIRTGYQAKHIREWRRQGIQVLVSTSNVSSLEGARALIAEAT<br>KLGPVGGVFNLAMVLRDAMLENQTPELFQDVNKPKYNGTLNLDRATREACPELDYFVAFSSVSCGRGNAGQT<br>NYGFANSTMERICEQRRHDGLPGLAVQWGAIGDVGIVLEAMGTNDTVIGGTLPQRISSCMEVLDLFLNQPHAV<br>LSSFVLAEKKAVAHGDGDTQRDLVKAVAHILGIRDLAGINLDSTLADLGLDSLMGVEVRQILEREHDLVLPMRE<br>VRQLTLRKLQEMSSKTDSAAATARRPGDGRPTFAGVHGDDAAEVHARDLTLDRFLDDATLAAATALPGPAPE<br>IRTVLLTGATGFLGRYLALEWLERMAMVGGTLICLVRGRDDAAARARLDQIFDSGDPELLRHYRELADRHLEVI<br>AGDKSDADLGLDRRTWQRLADTVDLIVDPAALVNHVLPYRELFGPNVVGTAELIRLALTGRLKPYLYTSTIAVG<br>AGIAPGQFTEDADIRQISATRTLDDSYANGYATSKWAGEVLLREAHDLCGLPVAVFRCDMILADTSYAGQLNVP<br>DMFTRLILSLVATGIAPLSFYELDAAGHRQRAHYDGLPVEFVAEAVSALGLDVAEDGGFATYHVMNPYDDGIGL<br>DEFVDWLTGAGYPIEHVNDYGTWFQRFETAIRGLPERQRQASLLPLLHSYQRPQPPIRGSAAPTDRFRSAVQD<br>AKIGPDKDIPHITPEVIVKYVTDLRLLGLLGGKLHHHHHHHH
pMMH01 (mouseFAS_ΔTE _CAR-TR_Mphi, Strep-and His-tagged)<br>Position 113 highlighted	ATGAGCGCTTGGAGCCATCCACAATTTGAGAAGGGTGGAGGTTCTGGCGGTGGATCGGGAGGTTCAGCGTGG<br>AGCCACCCGCAGTTCGAAAAAGGCGCCGGATCCGAGGAGGTGGTGATAGCCGGTATGTCGGGGAAGTTGCCCG<br>AGTCAGAGAACCTACAGGAGTTCTGGGCCAACCTCATTGGTGGTGTGGACATGGTCACAGATGATGACAGGA<br>GATGGAAGGCTGGGCTCTATGGATTACCCAAGCGGTCTGGAAAGCTGAAGGATCTCTCCAAGTTCGACGCCTC<br>CTTTTTTGGGGTCCACCCCAAGCAGGCACACACAATGGACCCCCAGCTTCGGCTGCTGTTGGAAGTCAGCTAT<br>GAAGCAATTGTGGATGGAGGTATCAACCCAGCCTCACTCCGAGGAACGAACACTGGCGTCTGGGTGGGTGTGA<br>GT**GGT/TGG**TCAGAGGCATCCGAGGCCCTTAGCAGAGATCCCGAGACGCTTCTGGGCTACAGCATGGTGGGCT<br>GCCAGCGTGCAATGATGGCCAACCGGCTCTCTTTCTTCTTCGACTTCAAAGGACCAAGCATTGCCCTGGACAC<br>AGCCTGCTCCTCCAGCTTGCTGGCACTACAGAATGCCTACCAGGCCATCCGTAGTGGGGAATGCCCCGCGGCCC<br>TTGTGGGTGGGATCAACCTGCTCCTGAAGCCGAACACCTCTGTGCAGTTCATGAAGCTGGGCATGCTCAGCCC<br>GGACGGCACCTGCAGATCCTTTGATGATTCAGGGAGTGGATATTGTCGCTCTGAGGCTGTTGTAGCAGTTCTG<br>CTGACTAAGAAGTCCCTGGCTCGGCGGGTCTATGCCACGATTCTGAATGCCGGCACCAATACAGATGGCAGCA<br>AGGAGCAAGGTGTAACATTCCCCTCTGGAGAAGTCCAAGAACAACTCATCTGCTCTCTGTATCAGCCAGCTGG<br>TCTGGCCCCGGAGTCGCTTGAGTATATTGAAGCCCATGGCACGGGCACCAAGGTGGGTGACCCCCAGGAACTG<br>AATGGCATTACTCGGTCCCTGTGCGCCTTCCGCCAGGCCCCTCTGTTAATTGGCTCCACCAAATCCAACATGGG<br>ACACCCTGAGCCTGCCTCTGGGCTTGCAGCCCTGACCAAGGTGCTGTTATCCCTGGAGCATGGGGTCTGGCCC<br>CTAACCTGCACTTCCACAACCCCAACCCTGAGATCCCAGCACTTCTTGATGGGCGGCTGCAGGTGGTCGATAG<br>GCCCCTGCCTGTTCGTGGTGGCAACGTGGGCATCAACTCATTTGGCTTCGGAGGCTCCAATGTTCATGTCATC<br>CTCCAGCCCAACACACGGCAGGCCCCTGCGCCCACTGCACACGCTGCCCTTCCCCATTTGCTGCACGCCAGTGG<br>ACGCACCTTAGAGGCAGTGCAGGACCTGCTGGAACAGGGCCGCCAGCACAGCCAGGACCTGGCCTTTGTGAGC<br>ATGCTCAATGACATTGCGGCAACCCCTACAGCAGCCATGCCCTTCAGGGGTTACACTGTGCTAGGTGTTGAGG

Construct	Sequence form start to stop codon
	GCCGTGTCCAAGAAGTGCAGCAAGTGTCCACCAACAAGCGCCCACTCTGGTTCATCTGCTCAGGGATGGGCAC<br>GCAGTGGCGCGGGATGGGGCTGAGCCTCATGCGCCTGGACAGCTTCCGTGAGTCTATCCTGCGCTCCGATGAG<br>GCTGTGAAGCCGTTGGGAGTGAAAGTGTCAGATCTGCTGTTGAGCACAGATGAGCGCACCTTTGATGACATCG<br>TGCATGCCTTTGTGAGCCTCACTGCCATCCAGATTGCCCTCATCGACCTACTGACTTCTGTGGGACTGAAACC<br>TGACGGCATCATTGGGCACTCCTTGGGAGAGGTTGCCTGTGGCTATGCAGATGGCTGTCTCTCCCAGAGAGAG<br>GCTGTGCTTGCAGCTTACTGGCGAGGCCAGTGCATCAAAGATGCCCACCTCCCGCCTGGATCCATGGCAGCTG<br>TTGGTTTGTCCTGGGAGGAATGTAAACAGCGCTGCCCCGCTGGCGTGGTGCCTGCCTGCCACAACTCTGAGGA<br>CACCGTGACCATCTCTGGACCTCAGGCTGCAGTGAATGAATTTGTGGAGCAGCTAAAGCAAGAAGGTGTGTTT<br>GCCAAGGAGGTACGAACAGGAGGCCTGGCTTTCCACTCCTACTTCATGGAAGGAATTGCCCCCACATTGCTGC<br>AGGCTCTCAAGAAGGTGATCCGGGAACCACGGCCGCGCTCGGCTCGATGGCTCAGCACCTCTATCCCTGAGGC<br>CCAGTGGCAGAGCAGCCTGGCCCGCACATCTTCTGCCGAGTACAATGTCAACAACCTGGTGAGCCCTGTGCTC<br>TTCCAGGAAGCACTGTGGCACATCCCTGAGCATGCCGTGGTGCTGGAGATTGCGCCCCACGCACTGTTGCAGG<br>CTGTCCTGAAGCGAGGCGTGAAGTCCAGCTGCACCATCATTCCCTTGATGAAGAGGGATCATAAAGATAACTT<br>GGAGTTCTTTCTCACCAACCTTGGCAAGGTGCACCTCACAGGCATCAATGTCAACCCTAACGCCTTGTTCCCAC<br>CTGTGGAGTTCCCGGCTCCCCGAGGGACTCCTCTCATCTCCCCTCACATCAAGTGGGACCACAGTCAGACTTGG<br>GATGTCCCGGTTGCTGAGGACTTCCCAAACGGCTCCAGCTCCTCCTCTGCTACAGTCTACAGCATCGACGCCAG<br>TCCTGAGTCGCCCGACCACTACCTGGTAGACCACTGCATTGACGGCCGGGTCATCTTCCCTGGCACTGGCTACC<br>TGTGCCTGGTGTGGAAGACACTGGCTCGCAGCCTGGGCTTGTCCCTAGAAGAGACCCCTGTGGTATTTGAGAA<br>TGTGTCGTTTCATCAGGCCACTATACTACCCAAGACAGGAACCGTGGCGCTGGAGGTGAGGCTGCTAGAGGCC<br>TCCCATGCCTTTGAGGTGTCTGACACTGGCAATCTGATTGTGAGCGGAAAAGTGTACCTGTGGGAAGACCCGA<br>ACTCCAAGTTATTCGACCACCCAGAAGTCCCAACACCCCCTGAGTCTGCATCGGTCTCCCGCCTGACCCAGGGA<br>GAAGTATACAAGGAGCTGCGGCTGCGTGGCTATGATTATGGCCCTCAGTTCCAGGGCATCTGTGAGGCCACCC<br>TTGAAGGTGAACAAGGCAAGCTGCTCTGGAAAGATAACTGGGTGACCTTCATGGACACAATGCTGCAGGTAT<br>CCATTCTGGGTTCTAGCCAGCAGAGTCTACAGCTACCTACCCGTGTGACCGCCATCTATATCGACCCTGCCACC<br>CACCGTCAGAAGGTGTACAGGCTGAAGGAGGACACTCAAGTGGCTGATGTGACAACGAGCCGCTGTCTGGGCA<br>TAACGGTCTCTGGTGGTATCCACATCTCAAGACTACAGACGACAGCAACCTCACGGCGGCAGCAAGAACAGCT<br>GGTCCCCACCTTGGAAAAGTTCGTTTTCACACCGCACATGGAGGCTGAGTGCCTGTCTGAGAGCACTGCCCTG<br>CAGAAGGAGCTGCAACTGTGCAAGGGTCTGGCACGGGCTCTGCAGACCAAGGCCACCCAGCAAGGGCTGAAGG<br>CGGCAATGCTTGGGCAAGAGGACCCTCCACAGCACGGGCTGCCTCGACTCCTGGCAGCTGCTTGCCAGTTGCA<br>GCTCAACGGGAACCTGCAGCTGGAGCTGGGAGAAGCGCTGGCTCAAGAGAGGCTCCTGCTGCCAGAAGACCCT<br>CTGATCAGTGGCCTCCTCAACTCCCAGGCCCTCAAGGCCTGCGTAGACACAGCCCTGGAGAACTTGTCTACTCT<br>CAAGATGAAGGTGGCAGAGGTGCTGGCTGGAGAAGGCCACTTGTATTCCCGAATCCCGGCACTGCTCAACACC<br>CAGCCCATGCTACAACTGGAATACACAGCCACCGACCGGCACCCCCAGGCCCTGAAGGATGTTCAGACCAAAC<br>TGCAGCAGCATGATGTGGCGCAGGGCCAGTGGAACCCTTCCGACCCTGCGCCCAGCAGCCTGGGTGCCCTTGA<br>CCTTCTGGTGTGCAACTGTGCATTAGCCACCCTGGGGGATCCAGCCTTGGCCCTGGACAACATGGTAGCTGCC<br>CTCAAGGAAGGTGGTTTCCTGCTAGTGCACACAGTGCTCAAAGGACATGCCCTTGGGGAGACCCTGGCCTGCC<br>TACCCTCTGAGGTGCAGCCTGCGCCCAGCCTCCTAAGCCAGGAGGAGTGGGAGAGCCTGTTCTCGAGGAAGGC<br>ACTACACCTGGTGGGCCTTAAAAGGTCCTTCTACGGTACTGCGCTGTTCCTGTGCCGGCGAGCCATCCCACAG<br>GAGAAACCTATCTTCCTGTCTGTGGAGGATACCAGCTTCCAGTGGGTGGACTCTCTGAAGAGCACTCTGGCCA<br>CGTCCTCCTCCCAGCCTGTGTGGCTAACGGCCATGGACTGCCCCACCTCGGGTGTGGTGGGTTTGGTGAATTG<br>TCTCCGAAAAGAGCCGGGTGGACACCGGATTCGGTGTATCCTGCTGTCCAACCTCAGCAACACATCTCACGCC<br>CCCAAGTTGGACCCTGGCTCTCCAGAGCTACAGCAGGTGCTAAAGCATGACCTCGTGATGAACGTGTACCGGG<br>ACGGGGCCTGGGGTGCCTTCCGTCACTTCCAGTTAGAGCAGGACAAGCCCAAGGAGCAGACAGCGCATGCCTT<br>TGTAAACGTCCTCACCCGAGGGGACCTCGCCTCCATCCGCTGGGTCTCCTCCCCCCTGAAGCACACGCAGCCCT<br>CGAGCTCAGGAGCACAGCTCTGCACTGTCTACTACGCCTCACTGAACTTCCGAGACATCATGCTGGCCACGGG<br>CAAGCTGTCCCCTGATGCCATTCCAGGTAAATGGGCCAGCCGAGACTGCATGCTCGGCATGGAGTTCTCAGGC<br>CGGGATAGGTGTGGCCGGCGTGTGATGGGGCTGGTTCCTGCAGAAGGCCTGGCCACCTCAGTCCTGCTATCAT<br>CTGACTTCCTCTGGGATGTACCCTCCAGCTGGACCCTGGAGGAGGCGGCCTCTGTGCCCGTCGTCTATACCACT<br>GCTTACTACTCGTTAGTGGTTCGCGGGCGCATCCAGCGTGGGGAGACCGTGCTCATCCACTCAGGTTCAGGTG<br>GTGTGGGCCAAGCGGCCATTTCCATTGCCCTCAGTCTGGGCTGCCGCGTCTTCACCACTGTGGGCTCTGCAGAG<br>AAGCGAGCATACCTCCAGGCCAGGTTCCCTCAGCTTGATGACACCAGCTTTGCCAACTCGAGGGACACATCAT<br>TTGAGCAGCACGTGTTACTGCACACAGGTGGCAAAGGGGTCGACCTGGTCCTCAACTCACTGGCAGAAGAGAA<br>GCTGCAGGCCAGTGTGCGGTGCTTGGCTCAGCATGGTCGCTTCTTAGAGATTGGCAAATTTGATCTTTCTAAC<br>AACCACCCTCTGGGCATGGCTATCTTCTTGAAGAACGTCACTTTCCATGGGATCCTGCTGGACGCCCTTTTTG<br>AGGAGGCCAATGACAGCTGGCGGGAGGTGGCGGCACTCCTGAAGGCTGGCATTCGTGATGGAGTCGTGAAGCC<br>CCTCAAGTGCACAGTGTTTCCCAAGGCCCAGGTGGAAGATGCCTTCCGCTACATGGCTCAGGGGAAACACATT<br>GGCAAAGTCCTTGTCCAGGTACGGGAGGAGGAGCCTGAGGCTGTGCTGCCAGGGGCTCAGCCCACCCTGATTT<br>CTGCCATCTCCAAGACCTTCTGCCCAGCCCATAAGAGTTACATCATCACTGGTGGCCTAGGTGGCTTTGGCCT<br>GGAGCTGGCCCGGTGGCTCGTGCTTCGCGGAGCCCAGAGGCTTGTGCTGACTTCCCGATCTGGAATCCGCACC<br>GGCTACCAAGCCAAGCACATTCGGGAGTGGAGACGCCAGGGCATCCAAGTGCTCGTGTCAACAAGCAACGTGA<br>GCTCACTGGAGGGGGCCCGTGCTCTCATCGCCGAAGCCACAAAGCTGGGGCCCGTTGGGGGTGTCTTCAACCT<br>GGCCATGGTTTTGAGGGATGCCATGCTGGAGAACCAGACCCCAGAGCTCTTCCAGGATGTCAACAAGCCCAAA<br>TACAATGGCACCCTGAACCTTGACAGGGCAACCCGGGAAGCCTGCCCTGAGCTGGACTACTTTGTGGCCTTCT

Construct	Sequence form start to stop codon
	CCTCTGTAAGCTGCGGGCGTGGTAATGCTGGCCAAACTAACTACGGCTTCGCCAACTCTACCATGGAGCGTAT<br>ATGTGAACAGCGCAGGCACGATGGCCTCCCAGGCCTTGCCGTGCAGTGGGGTGCCATTGGTGACGTGGGCATT<br>GTCCTGGAAGCGATGGGCACCAATGACACAGTCATCGGAGGTACGCTGCCTCAGCGCATCTCCTCCTGCATGG<br>AGGTACTGGACCTCTTCCTGAATCAGCCCCACGCAGTCCTGAGCAGCTTTGTGCTGGCAGAGAAGAAAGCTGT<br>GGCCCATGGGGACGGGGACACCCAGAGGGATCTGGTGAAAGCTGTAGCACACATCCTAGGCATCCGAGACCTC<br>GCAGGTATTAACCTGGACAGCACGCTGGCAGACCTCGGCCTGGACTCGCTCATGGGTGTGGAAGTTCGTCAGA<br>TCCTGGAACGAGAACACGATCTGGTGCTGCCCATGCGTGAGGTGCGGCAGCTCACGCTGCGGAAACTTCAGGA<br>AATGTCCTCCAAGACTGACTCGGCGGCCGCAACCGCGCGCCGACCAGGCGACGGACGGCCCACCTTCGCCGGGG<br>TCCACGGCGACGACGCCGCCGAGGTGCACGCCCGCGATCTGACCCTGGACAGGTTCCTCGACGACGCGACGCTG<br>GCCGCCGCGACCGCTCTGCCCGGTCCGGCGCCGGAGATCCGCACGGTGCTGCTGACCGGGGCGACCGGATTCCT<br>CGGCCGCTACCTGGCGCTGGAGTGGCTGGAGCGGATGGCGATGGTCGGCGGCACCCTGATCTGCCTGGTGCGC<br>GGCCGCGACGACGCCGCCGCCCGGGCCCGGCTGGATCAGATCTTCGACTCCGGCGATCCCGAACTGCTGCGCCA<br>CTACCGGGAGTTGGCCGACCGCCATCTCGAGGTCATCGCCGGCGACAAGAGCGACGCCGACCTCGGCCTGGAC<br>CGCCGGACCTGGCAGCGGCTGGCCGATACCGTCGACCTGATCGTCGACCCCGCCGCCCTGGTCAACCATGTGCT<br>GCCGTACCGGGAGTTGTTCGGGCCCAACGTGGTCGGAACCGCCGAGCTGATCCGTCTCGCGCTCACCGGCAGG<br>CTCAAGCCGTATCTGTACACCTCGACGATCGCTGTGGGCGCGGGGATTGCGCCCGGGCAGTTCACCGAGGACG<br>CCGACATCCGGCAGATCAGCGCCACCCGGACACTCGATGACAGCTACGCCAACGGCTACGCCACCAGCAAGTG<br>GGCCGGCGAGGTGCTGCTGCGGGAGGCACACGATCTGTGCGGCCTGCCGGTGGCGGTGTTCCGGTGCGACATG<br>ATCCTGGCCGACACCAGCTACGCCGGTCAGCTCAACGTGCCGGACATGTTCACCCGGCTGATCCTGAGCCTGGT<br>GGCCACCGGGATCGCGCCGCTGTCGTTCTACGAACTCGACGCCGCCGGGCACCGGCAGCGGGCGCACTACGACG<br>GTCTGCCGGTGGAGTTCGTCGCCGAGGCCGTCTCCGCACTCGGTCTCGACGTGGCCGAGGACGGCGGCTTCGCG<br>ACCTACCACGTGATGAACCCGTACGACGACGGCATCGGGCTCGACGAGTTCGTCGACTGGCTGACCGGGGCCG<br>GGTATCCGATCGAGCACGTCAACGACTACGGCACCTGGTTTCAGCGGTTCGAGACCGCGATCCGCGGCCTGCC<br>CGAACGGCAGCGGCAGGCGTCGCTGCTTCCGCTGCTGCACAGCTACCAGCGGCCGCAGCCGCCGATCCGCGGGT<br>CGGCCGCCCCGACCGATCGGTTCCGCAGCGCGGTGCAGGACGCGAAGATCGGCCCGGACAAGGACATTCCGCA<br>CATCACCCCGGAGGTGATCGTCAAGTACGTCACCGATCTGCGGCTGCTCGGGCTGCTCGGAGGTAAGCTTCAT<br>CATCACCACCACCACCACCAC
pMMH02 (mouseFAS_ΔTE _CAR-TR_Noti, Strep-and His-tagged)<br>Position 113 highlighted	MSAWSHPQFEKGGGSGGGSGGSAWSHPQFEKGAGSEEVVIAGMSGKLPESENLQEFWANLIGGVDMVTDDDR<br>RWKAGLYGLPKRSGKLKDLSKFDASFFGVHPKQAHTMDPQLRLLLEVSYEAIVDGGINPASLRGTNTGVWVGV<br>S**G/W**SEASEALSRDPETLLGYSMVGCQRAMMANRLSFFFDFKGPSIALDTACSSSLLALQNAYQAIRSGECPAA<br>LVGGINLLLKPNTSVQFMKLGMLSPDGTCRSFDDSGSGYCRSEAVVAVLLTKKSLARRVYATILNAGTNTDGSK<br>EQGVTFPSGEVQEQLICSLYQPAGLAPESLEYIEAHGTGTKVGDPQELNGITRSLCAFRQAPLLIGSTKSNMGHP<br>EPASGLAALTKVLLSLEHGVWAPNLHFHNPNPEIPALLDGRLQVVDRPLPVRGGNVGINSFGFGGSNVHVILQP<br>NTRQAPAPTAHAALPHLLHASGRTLEAVQDLLEQGRQHSQDLAFVSMLNDIAATPTAAMPFRGYTVLGVEGR<br>VQEVQQVSTNKRPLWFICSGMGTQWRGMGLSLMRLDSFRESILRSDEAVKPLGVKVSDLLLSTDERTFDDIVH<br>AFVSLTAIQIALIDLLTSVGLKPDGIIGHSLGEVACGYADGCLSQREAVLAAYWRGQCIKDAHLPPGSMAAVGLS<br>WEECKQRCPAGVVPACHNSEDTVTISGPQAAVNEFVEQLKQEGVFAKEVRTGGLAFHSYFMEGIAPTLLQALK<br>KVIREPRPRSARWLSTSIPEAQWQSSLARTSSAEYNVNNLVSPVLFQEALWHIPEHAVVLEIAPHALLQAVLKR<br>GVKSSCTIIPLMKRDHKDNLEFFLTNLGKVHLTGINVNPNALFPPVEFPAPRGTPLISPHIKWDHSQTWDVPVA<br>EDFPNGSSSSSATVYSIDASPESPDHYLVDHCIDGRVIFPGTGYLCLVWKTLARSLGLSLEETPVVFENVSFHQAT<br>ILPKTGTVALEVRLLEASHAFEVSDTGNLIVSGKVYLWEDPNSKLFDHPEVPTPPESASVSRLTQGEVYKELRLR<br>GYDYGPQFQGICEATLEGEQGKLLWKDNWVTFMDTMLQVSILGSSQQSLQLPTRVTAIYIDPATHRQKVYRLK<br>EDTQVADVTTSRCLGITVSGGIHISRLQTTATSRRQQEQLVPTLEKFVFTPHMEAECLSESTALQKELQLCKGLA<br>RALQTKATQQGLKAAMLGQEDPPQHGLPRLLAAACQLQLNGNLQLELGEALAQERLLLPEDPLISGLLNSQAL<br>KACVDTALENLSTLKMKVAEVLAGEGHLYSRIPALLNTQPMLQLEYTATDRHPQALKDVQTKLQQHDVAQGQ<br>WNPSDPAPSSLGALDLLVCNCALATLGDPALALDNMVAALKEGGFLLVHTVLKGHALGETLACLPSEVQPAPS<br>LLSQEEWESLFSRKALHLVGLKRSFYGTALFLCRRAIPQEKPIFLSVEDTSFQWVDSLKSTLATSSSQPVWLTA<br>MDCPTSGVVGLVNCLRKEPGGHRIRCILLSNLSNTSHAPKLDPGSPELQQVLKHDLVMNVYRDGAWGAFRHF<br>QLEQDKPKEQTAHAFVNVLTRGDLASIRWVSSPLKHTQPSSSGAQLCTVYYASLNFRDIMLATGKLSPDAIPGK<br>WASRDCMLGMEFSGRDRCGRRVMGLVPAEGLATSVLLSSDFLWDVPSSWTLEEAASVPVVYTTAYYSLVVRG<br>RIQRGETVLIHSGSGGVGQAAISIALSLGCRVFTTVGSAEKRAYLQARFPQLDDTSFANSRDTSFEQHVLLHTGG<br>KGVDLVLNSLAEEKLQASVRCLAQHGRFLEIGKFDLSNNHPLGMAIFLKNVTFHGILLDALFEEANDSWREVA<br>ALLKAGIRDGVVKPLKCTVFPKAQVEDAFRYMAQGKHIGKVLVQVREEEPEAVLPGAQPTLISAISKTFCPAHK<br>SYIITGGLGGFGLELARWLVLRGAQRLVLTSRSGIRTGYQAKHIREWRRQGIQVLVSTSNVSSLEGARALIAEAT<br>KLGPVGGVFNLAMVLRDAMLENQTPELFQDVNKPKYNGTLNLDRATREACPELDYFVAFSSVSCGRGNAGQT<br>NYGFANSTMERICEQRRHDGLPGLAVQWGAIGDVGIVLEAMGTNDTVIGGTLPQRISSCMEVLDLFLNQPHAV<br>LSSFVLAEKKAVAHGDGDTQRDLVKAVAHILGIRDLAGINLDSTLADLGLDSLMGVEVRQILEREHDLVLPMRE<br>VRQLTLRKLQEMSSKTDSAAASERDSGSRPTAATVHGDDGLLRADDLALEAFLDPATLDAAAHLPSALEPPRT<br>VLLTGANGYLGRFLALEWLQRLDVSGGTLICLIRGSDADSARRRLDAVFATGDPELEAHYRELAERRLRVLPGD<br>IGEPNLGLREQDWRDLAETVDLIVHPAALVNHVLPYAQLFGPNVVGTAEVIRLALTSRLKPVTYLSTVAVSAGI<br>DPETFTEDGDIREISPVRRLDDGYANGYGNSKWAGEVLLRNAHDRFGLPVAVFRSDMILAHSRYAGQLNVPD<br>MFTRLLLSVLATGLAPGSFHDAHGERHRAHYDGLPADFTAAAVTTLGSRVTSGYETYDVLNPHDDGISLDTFV<br>DWLIEAGHPIDRIDDYAEWFARFDTALRALPEHQRQHSLLPLLHAYRRPTPPLHGVALPAKHFRAAVQQAKL

Construct	Sequence form start to stop codon
	GPDGDIPHVTRELIEKYASDLRLLGLIQGGGKLHHHHHHHH
pMMH02<br>(mouseFAS_∆TE<br>_CAR-TR_Noti,<br>Strep-and His-<br>tagged)<br>Position 113<br>highlighted	ATGAGCGCTTGGAGCCATCCACAATTTGAGAAGGGTGGAGGTTCTGGCGGTGGATCGGGAGGTTCAGCGTGG<br>AGCCACCCGCAGTTCGAAAAAGGCGCCGGATCCGAGGAGGTGGTGATAGCCGGTATGTCGGGGAAGTTGCCCG<br>AGTCAGAGAACCTACAGGAGTTCTGGGCCAACCTCATTGGTGGTGTGGACATGGTCACAGATGATGACAGGA<br>GATGGAAGGCTGGGCTCTATGGATTACCCAAGCGGTCTGGAAAGCTGAAGGATCTCTCCAAGTTCGACGCCTC<br>CTTTTTTGGGGTCCACCCCAAGCAGGCACACACAATGGACCCCCAGCTTCGGCTGCTGTTGGAAGTCAGCTAT<br>GAAGCAATTGTGGATGGAGGTATCAACCCAGCCTCACTCCGAGGAACGAACACTGGCGTCTGGGTGGGTGTGA<br>GT**GGT/TGG**TCAGAGGCATCCGAGGCCCTTAGCAGAGATCCCGAGACGCTTCTGGGCTACAGCATGGTGGGCT<br>GCCAGCGTGCAATGATGGCCAACCGGCTCTCTTTCTTCTTCGACTTCAAAGGACCAAGCATTGCCCTGGACAC<br>AGCCTGCTCCTCCAGCTTGCTGGCACTACAGAATGCCTACCAGGCCATCCGTAGTGGGGAATGCCCCGCGGCCC<br>TTGTGGGTGGGATCAACCTGCTCCTGAAGCCGAACACCTCTGTGCAGTTCATGAAGCTGGGCATGCTCAGCCC<br>GGACGGCACCTGCAGATCCTTTGATGATTCAGGGAGTGGATATTGTCGCTCTGAGGCTGTTGTAGCAGTTCTG<br>CTGACTAAGAAGTCCCTGGCTCGGCGGGTCTATGCCACGATTCTGAATGCCGGCACCAATACAGATGGCAGCA<br>AGGAGCAAGGTGTAACATTCCCCTCTGGAGAAGTCCAAGAACAACTCATCTGCTCTCTGTATCAGCCAGCTGG<br>TCTGGCCCCGGAGTCGCTTGAGTATATTGAAGCCCATGGCACGGGCACCAAGGTGGGTGACCCCCAGGAACTG<br>AATGGCATTACTCGGTCCCTGTGCGCCTTCCGCCAGGCCCCTCTGTTAATTGGCTCCACCAAATCCAACATGGG<br>ACACCCTGAGCCTGCCTCTGGGCTTGCAGCCCTGACCAAGGTGCTGTTATCCCTGGAGCATGGGGTCTGGGCCC<br>CTAACCTGCACTTCCACAACCCCAACCCTGAGATCCCAGCACTTCTTGATGGGCGGCTGCAGGTGGTCGATAG<br>GCCCCTGCCTGTTCGTGGTGGCAACGTGGGCATCAACTCATTTGGCTTCGGAGGCTCCAATGTTCATGTCATC<br>CTCCAGCCCAACACACGGCAGGCCCCTGCGCCCACTGCACACGCTGCCCTTCCCCATTTGCTGCACGCCAGTGG<br>ACGCACCTTAGAGGCAGTGCAGGACCTGCTGGAACAGGGCCGCCAGCACAGCCAGGACCTGGCCTTTGTGAGC<br>ATGCTCAATGACATTGCGGCAACCCCTACAGCAGCCATGCCCTTCAGGGGTTACACTGTGCTAGGTGTTGAGG<br>GCCGTGTCCAAGAAGTGCAGCAAGTGTCCACCAACAAGCGCCCACTCTGGTTCATCTGCTCAGGGATGGGCAC<br>GCAGTGGCGCGGGATGGGGCTGAGCCTCATGCGCCTGGACAGCTTCCGTGAGTCTATCCTGCGCTCCGATGAG<br>GCTGTGAAGCCGTTGGGAGTGAAAGTGTCAGATCTGCTGTTGAGCACAGATGAGCGCACCTTTGATGACATCG<br>TGCATGCCTTTGTGAGCCTCACTGCCATCCAGATTGCCCTCATCGACCTACTGACTTCTGTGGGACTGAAACC<br>TGACGGCATCATTGGGCACTCCTTGGGAGAGGTTGCCTGTGGCTATGCAGATGGCTGTCTCTCCCAGAGAGAG<br>GCTGTGCTTGCAGCTTACTGGCGAGGCCAGTGCATCAAAGATGCCCACCTCCCGCCTGGATCCATGGCAGCTG<br>TTGGTTTGTCCTGGGAGGAATGTAAACAGCGCTGCCCCGCTGGCGTGGTGCCTGCCTGCCACAACTCTGAGGA<br>CACCGTGACCATCTCTGGACCTCAGGCTGCAGTGAATGAATTTGTGGAGCAGCTAAAGCAAGAAGGTGTGTTT<br>GCCAAGGAGGTACGAACAGGAGGCCTGGCTTTCCACTCCTACTTCATGGAAGGAATTGCCCCCACATTGCTGC<br>AGGCTCTCAAGAAGGTGATCCGGGAACCACGGCCGCGCTCGGCTCGATGGCTCAGCACCTCTATCCCTGAGGC<br>CCAGTGGCAGAGCAGCCTGGCCCGCACATCTTCTGCCGAGTACAATGTCAACAACCTGGTGAGCCCTGTGCTC<br>TTCCAGGAAGCACTGTGGCACATCCCTGAGCATGCCGTGGTGCTGGAGATTGCGCCCCACGCACTGTTGCAGG<br>CTGTCCTGAAGCGAGGCGTGAAGTCCAGCTGCACCATCATTCCCTTGATGAAGAGGGATCATAAAGATAACTT<br>GGAGTTCTTTCTCACCAACCTTGGCAAGGTGCACCTCACAGGCATCAATGTCAACCCTAACGCCTTGTTCCCAC<br>CTGTGGAGTTCCCGGCTCCCCGAGGGACTCCTCTCATCTCCCCTCACATCAAGTGGGACCACAGTCAGACTTGG<br>GATGTCCCGGTTGCTGAGGACTTCCCAAACGGCTCCAGCTCCTCCTCTGCTACAGTCTACAGCATCGACGCCAG<br>TCCTGAGTCGCCCGACCACTACCTGGTAGACCACTGCATTGACGGCCGGGTCATCTTCCCTGGCACTGGCTACC<br>TGTGCCTGGTGTGGAAGACACTGGCTCGCAGCCTGGGCTTGTCCCTAGAAGAGACCCCTGTGGTATTTGAGAA<br>TGTGTCGTTTCATCAGGCCACTATACTACCCAAGACAGGAACCGTGGCGCTGGAGGTGAGGCTGCTAGAGGCC<br>TCCCATGCCTTTGAGGTGTCTGACACTGGCAATCTGATTGTGAGCGGAAAAGTGTACCTGTGGGAAGACCCGA<br>ACTCCAAGTTATTCGACCACCCAGAAGTCCCAACACCCCCTGAGTCTGCATCGGTCTCCCGCCTGACCCAGGGA<br>GAAGTATACAAGGAGCTGCGGCTGCGTGGCTATGATTATGGCCCTCAGTTCCAGGGCATCTGTGAGGCCACCC<br>TTGAAGGTGAACAAGGCAAGCTGCTCTGGAAAGATAACTGGGTGACCTTCATGGACACAATGCTGCAGGTAT<br>CCATTCTGGGTTCTAGCCAGCAGAGTCTACAGCTACCTACCCGTGTGACCGCCATCTATATCGACCCTGCCACC<br>CACCGTCAGAAGGTGTACAGGCTGAAGGAGGACACTCAAGTGGCTGATGTGACAACGAGCCGCTGTCTGGGCA<br>TAACGGTCTCTGGTGGTATCCACATCTCAAGACTACAGACGACAGCAACCTCACGGCGGCAGCAAGAACAGCT<br>GGTCCCCACCTTGGAAAAGTTCGTTTTCACACCGCACATGGAGGCTGAGTGCCTGTCTGAGAGCACTGCCCTG<br>CAGAAGGAGCTGCAACTGTGCAAGGGTCTGGCACGGGCTCTGCAGACCAAGGCCACCCAGCAAGGGCTGAAGG<br>CGGCAATGCTTGGGCAAGAGGACCCTCCACAGCACGGGCTGCCTCGACTCCTGGCAGCTGCTTGCCAGTTGCA<br>GCTCAACGGGAACCTGCAGCTGGAGCTGGGAGAAGCGCTGGCTCAAGAGAGGCTCCTGCTGCCAGAAGACCCT<br>CTGATCAGTGGCCTCCTCAACTCCCAGGCCCTCAAGGCCTGCGTAGACACAGCCCTGGAGAACTTGTCTACTCT<br>CAAGATGAAGGTGGCAGAGGTGCTGGCTGGAGAAGGCCACTTGTATTCCCGAATCCCGGCACTGCTCAACACC<br>CAGCCCATGCTACAACTGGAATACACAGCCACCGACCGGCACCCCCAGGCCCTGAAGGATGTTCAGACCAAAC<br>TGCAGCAGCATGATGTGGCGCAGGGCCAGTGGAACCCTTCCGACCCTGCGCCCAGCAGCCTGGGTGCCCTTGA<br>CCTTCTGGTGTGCAACTGTGCATTAGCCACCCTGGGGGATCCAGCCTTGGCCCTGGACAACATGGTAGCTGCC<br>CTCAAGGAAGGTGGTTTCCTGCTAGTGCACACAGTGCTCAAAGGACATGCCCTTGGGGAGACCCTGGCCTGCC<br>TACCCTCTGAGGTGCAGCCTGCGCCCAGCCTCCTAAGCCAGGAGGAGTGGGAGAGCCTGTTCTCGAGGAAGGC<br>ACTACACCTGGTGGGCCTTAAAAGGTCCTTCTACGGTACTGCGCTGTTCCTGTGCCGGCGAGCCATCCCACAG

Construct	Sequence form start to stop codon
	GAGAAACCTATCTTCCTGTCTGTGGAGGATACCAGCTTCCAGTGGGTGGACTCTCTGAAGAGCACTCTGGCCA<br>CGTCCTCCTCCCAGCCTGTGTGGCTAACGGCCATGGACTGCCCCACCTCGGGTGTGGTGGGTTTGGTGAATTG<br>TCTCCGAAAAGAGCCGGGTGGACACCGGATTCGGTGTATCCTGCTGTCCAACCTCAGCAACACATCTCACGCC<br>CCCAAGTTGGACCCTGGCTCTCCAGAGCTACAGCAGGTGCTAAAGCATGACCTCGTGATGAACGTGTACCGGG<br>ACGGGGCCTGGGGTGCCTTCCGTCACTTCCAGTTAGAGCAGGACAAGCCCAAGGAGCAGACAGCGCATGCCTT<br>TGTAAACGTCCTCACCCGAGGGGACCTCGCCTCCATCCGCTGGGTCTCCTCCCCCCTGAAGCACACGCAGCCCT<br>CGAGCTCAGGAGCACAGCTCTGCACTGTCTACTACGCCTCACTGAACTTCCGAGACATCATGCTGGCCACGGG<br>CAAGCTGTCCCCTGATGCCATTCCAGGTAAATGGGCCAGCCGAGACTGCATGCTCGGCATGGAGTTCTCAGGC<br>CGGGATAGGTGTGGCCGGCGTGTGATGGGGCTGGTTCCTGCAGAAGGCCTGGCCACCTCAGTCCTGCTATCAT<br>CTGACTTCCTCTGGGATGTACCCTCCAGCTGGACCCTGGAGGAGGCGGCCTCTGTGCCCGTCGTCTATACCACT<br>GCTTACTACTCGTTAGTGGTTCGCGGGCGCATCCAGCGTGGGGAGACCGTGCTCATCCACTCAGGTTCAGGTG<br>GTGTGGGCCAAGCGGCCATTTCCATTGCCCTCAGTCTGGGCTGCCGCGTCTTCACCACTGTGGGCTCTGCAGAG<br>AAGCGAGCATACCTCCAGGCCAGGTTCCCTCAGCTTGATGACACCAGCTTTGCCAACTCGAGGGACACATCAT<br>TTGAGCAGCACGTGTTACTGCACACAGGTGGCAAAGGGGTCGACCTGGTCCTCAACTCACTGGCAGAAGAGAA<br>GCTGCAGGCCAGTGTGCGGTGCTTGGCTCAGCATGGTCGCTTCTTAGAGATTGGCAAATTTGATCTTTCTAAC<br>AACCACCCTCTGGGCATGGCTATCTTCTTGAAGAACGTCACTTTCCATGGGATCCTGCTGGACGCCCTTTTTG<br>AGGAGGCCAATGACAGCTGGCGGGAGGTGGCGGCACTCCTGAAGGCTGGCATTCGTGATGGAGTCGTGAAGCC<br>CCTCAAGTGCACAGTGTTTCCCAAGGCCCAGGTGGAAGATGCCTTCCGCTACATGGCTCAGGGGAAACACATT<br>GGCAAAGTCCTTGTCCAGGTACGGGAGGAGGAGCCTGAGGCTGTGCTGCCAGGGGCTCAGCCCACCCTGATTT<br>CTGCCATCTCCAAGACCTTCTGCCCAGCCCATAAGAGTTACATCATCACTGGTGGCCTAGGTGGCTTTGGCCT<br>GGAGCTGGCCCGGTGGCTCGTGCTTCGCGGAGCCCAGAGGCTTGTGCTGACTTCCCGATCTGGAATCCGCACC<br>GGCTACCAAGCCAAGCACATTCGGGAGTGGAGACGCCAGGGCATCCAAGTGCTCGTGTCAACAAGCAACGTGA<br>GCTCACTGGAGGGGGCCCGTGCTCTCATCGCCGAAGCCACAAAGCTGGGGCCCGTTGGGGGTGTCTTCAACCT<br>GGCCATGGTTTTGAGGGATGCCATGCTGGAGAACCAGACCCCAGAGCTCTTCCAGGATGTCAACAAGCCCAAA<br>TACAATGGCACCCTGAACCTTGACAGGGCAACCCGGGAAGCCTGCCCTGAGCTGGACTACTTTGTGGCCTTCT<br>CCTCTGTAAGCTGCGGGCGTGGTAATGCTGGCCAAACTAACTACGGCTTCGCCAACTCTACCATGGAGCGTAT<br>ATGTGAACAGCGCAGGCACGATGGCCTCCCAGGCCTTGCCGTGCAGTGGGGTGCCATTGGTGACGTGGGCATT<br>GTCCTGGAAGCGATGGGCACCAATGACACAGTCATCGGAGGTACGCTGCCTCAGCGCATCTCCTCCTGCATGG<br>AGGTACTGGACCTCTTCCTGAATCAGCCCCACGCAGTCCTGAGCAGCTTTGTGCTGGCAGAGAAGAAAGCTGT<br>GGCCCATGGGGACGGGGACACCCAGAGGGATCTGGTGAAAGCTGTAGCACACATCCTAGGCATCCGAGACCTC<br>GCAGGTATTAACCTGGACAGCACGCTGGCAGACCTCGGCCTGGACTCGCTCATGGGTGTGGAAGTTCGTCAGA<br>TCCTGGAACGAGAACACGATCTGGTGCTGCCCATGCGTGAGGTGCGGCAGCTCACGCTGCGGAAACTTCAGGA<br>AATGTCCTCCAAGACTGACTCGGCGGCCGCAAGCGAACGCGATTCCGGCAGTCGTCCGACCGCCGCCACGGTG<br>CACGGCGACGACGGCCTGCTGCGGGCCGACGATCTCGCCCTGGAGGCATTCCTCGACCCGGCGACCCTGGACGC<br>GGCCGCGCATCTGCCGAGCGCGCTGGAGCCGCCGCGCACGGTTCTGCTGACCGGCGCCAACGGCTACCTCGGCC<br>GCTTCCTCGCCCTGGAATGGCTACAGCGCCTGGACGTCTCGGGCGGTACTCTGATCTGCCTGATCCGCGGCAGT<br>GACGCCGACAGTGCTCGGCGCCGTCTCGACGCGGTCTTCGCCACCGGTGATCCGGAGCTGGAAGCCCACTACCG<br>CGAACTCGCCGAGCGGCGGCTGCGTGTCCTGCCCGGCGATATCGGTGAACCGAATCTCGGCCTGCGCGAACAG<br>GATTGGCGCGATCTGGCCGAGACCGTGGACCTGATCGTGCATCCGGCCGCCCTGGTCAACCATGTGCTGCCCT<br>ACGCCCAGTTGTTCGGCCCCAATGTTGTCGGTACCGCCGAGGTGATCCGTCTCGCGCTCACCTCCCGGCTGAAG<br>CCGGTCACCTACCTGTCCACCGTGGCGGTATCCGCCGGTATCGACCCGGAGACCTTCACCGAGGACGGCGATAT<br>CCGCGAGATCAGCCCCGTGCGCCGGCTCGACGACGGCTACGCCAATGGCTACGGCAACAGCAAATGGGCCGGC<br>GAGGTGCTGTTGCGCAATGCGCACGACCGCTTCGGTCTCCCGGTGGCGGTCTTCCGCTCCGACATGATCCTGGC<br>CCACAGCCGCTACGCCGGACAGCTCAACGTGCCCGACATGTTCACCCGCCTGCTGCTGAGCGTGCTGGCGACCG<br>GCCTGGCCCCCGGCTCCTTCCACGATGCCCACGGCGAGCGCCACCGCGCCCACTACGACGGTCTCCCAGCCGAT<br>TTCACCGCCGCGGCCGTCACCACCCTGGGCAGCCGGGTGACCAGTGGTTACGAGACCTACGACGTGCTCAACCC<br>GCACGACGACGGGATCTCCCTGGACACCTTCGTCGACTGGCTCATCGAGGCGGGCCACCCCATCGACCGCATCG<br>ACGATTACGCCGAGTGGTTCGCCCGCTTCGACACCGCCCTGCGCGCCCTCCCCGAACACCAACGCCAACACTCG<br>CTGCTACCCCTGCTGCACGCCTACCGTCGCCCCACCCCTCCCCTGCACGGAGTCGCCCTCCCCGCCAAGCACTTC<br>CGCGCCGCCGTACAGCAGGCGAAACTCGGCCCGGACGGGGATATCCCGCACGTGACGCGGGAGCTGATCGAGA<br>AGTACGCGAGTGATCTGCGGTTACTGGGGTTGATTCAGGGCGGAGGTAAGCTTCATCATCACCACCACCACCA<br>CCAC
pAR401 (mouseFAS_ΔTE_CAR-TR_Msme, Strep-and His-tagged) Position 113 highlighted	MSAWSHPQFEKGGGSGGGSGGSAWSHPQFEKGAGSEEVVIAGMSGKLPESENLQEFWANLIGGVDMVTDDDR<br>RWKAGLYGLPKRSGKLKDLSKFDASFFGVHPKQAHTMDPQLRLLLEVSYEAIVDGGINPASLRGTNTGVWVGV<br>S**G/W**SEASEALSRDPETLLGYSMVGCQRAMMANRLSFFFDFKGPSIALDTACSSSLLALQNAYQAIRSGECPAA<br>LVGGINLLLKPNTSVQFMKLGMLSPDGTCRSFDDSGSGYCRSEAVVAVLLTKKSLARRVYATILNAGTNTDGSK<br>EQGVTFPSGEVQEQLICSLYQPAGLAPESLEYIEAHGTGTKVGDPQELNGITRSLCAFRQAPLLIGSTKSNMGHP<br>EPASGLAALTKVLLSLEHGVWAPNLHFHNPNPEIPALLDGRLQVVDRPLPVRGGNVGINSFGFGGSNVHVILQP<br>NTRQAPAPTAHAALPHLLHASGRTLEAVQDLLEQGRQHSQDLAFVSMLNDIAATPTAAMPFRGYTVLGVEGR<br>VQEVQQVSTNKRPLWFICSGMGTQWRGMGLSLMRLDSFRESILRSDEAVKPLGVKVSDLLLSTDERTFDDIVH<br>AFVSLTAIQIALIDLLTSVGLKPDGIIGHSLGEVACGYADGCLSQREAVLAAYWRGQCIKDAHLPPGSMAAVGLS<br>WEECKQRCPAGVVPACHNSEDTVTISGPQAAVNEFVEQLKQEGVFAKEVRTGGLAFHSYFMEGIAPTLLQALK<br>KVIREPRPRSARWLSTSIPEAQWQSSLARTSSAEYNVNNLVSPVLFQEALWHIPEHAVVLEIAPHALLQAVLKR

Construct	Sequence form start to stop codon
	GVKSSCTIIPLMKRDHKDNLEFFLTNLGKVHLTGINVNPNALFPPVEFPAPRGTPLISPHIKWDHSQTWDVPVA<br>EDFPNGSSSSSATVYSIDASPESPDHYLVDHCIDGRVIFPGTGYLCLVWKTLARSLGLSLEETPVVFENVSFHQAT<br>ILPKTGTVALEVRLLEASHAFEVSDTGNLIVSGKVYLWEDPNSKLFDHPEVPTPPESASVSRLTQGEVYKELRLR<br>GYDYGPQFQGICEATLEGEQGKLLWKDNWVTFMDTMLQVSILGSSQQSLQLPTRVTAIYIDPATHRQKVYRLK<br>EDTQVADVTTSRCLGITVSGGIHISRLQTTATSRRQQEQLVPTLEKFVFTPHMEAECLSESTALQKELQLCKGLA<br>RALQTKATQQGLKAAMLGQEDPPQHGLPRLLAAACQLQLNGNLQLELGEALAQERLLLPEDPLISGLLNSQAL<br>KACVDTALENLSTLKMKVAEVLAGEGHLYSRIPALLNTQPMLQLEYTATDRHPQALKDVQTKLQQHDVAQGQ<br>WNPSDPAPSSLGALDLLVCNCALATLGDPALALDNMVAALKEGGFLLVHTVLKGHALGETLACLPSEVQPAPS<br>LLSQEEWESLFSRKALHLVGLKRSFYGTALFLCRRAIPQEKPIFLSVEDTSFQWVDSLKSTLATSSSQPVWLTA<br>MDCPTSGVVGLVNCLRKEPGGHRIRCILLSNLSNTSHAPKLDPGSPELQQVLKHDLVMNVYRDGAWGAFRHF<br>QLEQDKPKEQTAHAFVNVLTRGDLASIRWVSSPLKHTQPSSSGAQLCTVYYASLNFRDIMLATGKLSPDAIPGK<br>WASRDCMLGMEFSGRDRCGRRVMGLVPAEGLATSVLLSSDFLWDVPSSWTLEEAASVPVVYTTAYYSLVVRG<br>RIQRGETVLIHSGSGGVGQAAISIALSLGCRVFTTVGSAEKRAYLQARFPQLDDTSFANSRDTSFEQHVLLHTGG<br>KGVDLVLNSLAEEKLQASVRCLAQHGRFLEIGKFDLSNNHPLGMAIFLKNVTFHGILLDALFEEANDSWREVA<br>ALLKAGIRDGVVKPLKCTVFPKAQVEDAFRYMAQGKHIGKVLVQVREEEPEAVLPGAQPTLISAISKTFCPAHK<br>SYIITGGLGGFGLELARWLVLRGAQRLVLTSRSGIRTGYQAKHIREWRRQGIQVLVSTSNVSSLEGARALIAEAT<br>KLGPVGGVFNLAMVLRDAMLENQTPELFQDVNKPKYNGTLNLDRATREACPELDYFVAFSSVSCGRGNAGQT<br>NYGFANSTMERICEQRRHDGLPGLAVQWGAIGDVGIVLEAMGTNDTVIGGTLPQRISSCMEVLDLFLNQPHAV<br>LSSFVLAEKKAVAHGDGDTQRDLVKAVAHILGIRDLAGINLDSTLADLGLDSLMGVEVRQILEREHDLVLPMRE<br>VRQLTLRKLQEMSSKAAAAQRTAGDRRPSFTTVHGADATEIRASELTLDKFIDAETLRAAPGLPKVTTEPRTVL<br>LSGANGWLGRFLTLQWLERLAPVGGTLITIVRGRDDAAARARLTQAYDTDPELSRRFAELADRHLRVVAGDIG<br>DPNLGLTPEIWHRLAAEVDLVVHPAALVNHVLPYRQLFGPNVVGTAEVIKLALTERIKPVTYLSTVSVAMGIPD<br>FEEDGDIRTVSPVRPLDGGYANGYGNSKWAGEVLLREAHDLCGLPVATFRSDMILAHPRYRGQVNVPDMFTR<br>LLLSLLITGVAPRSFYIGDGERPRAHYPGLTVDFVAEAVTTLGAQQREGYVSYDVMNPHDDGISLDVFVDWLIR<br>AGHPIDRVDDYDDWVRRFETALTALPEKRRAQTVLPLLHAFRAPQAPLRGAPEPTEVFHAAVRTAKVGPGDIP<br>HLDEALIDKYIRDLREFGLIGGKLHHHHHHHH
pAR401 (mouseFAS_ΔTE _CAR-TR_Msme, Strep-and His-tagged)<br>Position 113 highlighted	ATGAGCGCTTGGAGCCATCCACAATTTGAGAAGGGTGGAGGTTCTGGCGGTGGATCGGGAGGTTCAGCGTGG<br>AGCCACCCGCAGTTCGAAAAAGGCGCCGGATCCGAGGAGGTGGTGATAGCCGGTATGTCGGGGAAGTTGCCCG<br>AGTCAGAGAACCTACAGGAGTTCTGGGCCAACCTCATTGGTGGTGTGGACATGGTCACAGATGATGACAGGA<br>GATGGAAGGCTGGGCTCTATGGATTACCCAAGCGGTCTGGAAAGCTGAAGGATCTCTCCAAGTTCGACGCCTC<br>CTTTTTTGGGGTCCACCCCAAGCAGGCACACACAATGGACCCCCAGCTTCGGCTGCTGTTGGAAGTCAGCTAT<br>GAAGCAATTGTGGATGGAGGTATCAACCCAGCCTCACTCCGAGGAACGAACACTGGCGTCTGGGTGGGTGTGA<br>GT**GGT/TGG**TCAGAGGCATCCGAGGCCCTTAGCAGAGATCCCGAGACGCTTCTGGGCTACAGCATGGTGGGCT<br>GCCAGCGTGCAATGATGGCCAACCGGCTCTCTTTCTTCTTCGACTTCAAAGGACCAAGCATTGCCCTGGACAC<br>AGCCTGCTCCTCCAGCTTGCTGGCACTACAGAATGCCTACCAGGCCATCCGTAGTGGGGAATGCCCCGCGGCCC<br>TTGTGGGTGGGATCAACCTGCTCCTGAAGCCGAACACCTCTGTGCAGTTCATGAAGCTGGGCATGCTCAGCCC<br>GGACGGCACCTGCAGATCCTTTGATGATTCAGGGAGTGGATATTGTCGCTCTGAGGCTGTTGTAGCAGTTCTG<br>CTGACTAAGAAGTCCCTGGCTCGGCGGGTCTATGCCACGATTCTGAATGCCGGCACCAATACAGATGGCAGCA<br>AGGAGCAAGGTGTAACATTCCCCTCTGGAGAAGTCCAAGAACAACTCATCTGCTCTCTGTATCAGCCAGCTGG<br>TCTGGCCCCGGAGTCGCTTGAGTATATTGAAGCCCATGGCACGGGCACCAAGGTGGGTGACCCCCAGGAACTG<br>AATGGCATTACTCGGTCCCTGTGCGCCTTCCGCCAGGCCCCTCTGTTAATTGGCTCCACCAAATCCAACATGGG<br>ACACCCTGAGCCTGCCTCTGGGCTTGCAGCCCTGACCAAGGTGCTGTTATCCCTGGAGCATGGGGTCTGGGCCC<br>CTAACCTGCACTTCCACAACCCCAACCCTGAGATCCCAGCACTTCTTGATGGGCGGCTGCAGGTGGTCGATAG<br>GCCCCTGCCTGTTCGTGGTGGCAACGTGGGCATCAACTCATTTGGCTTCGGAGGCTCCAATGTTCATGTCATC<br>CTCCAGCCCAACACACGGCAGGCCCCTGCGCCCACTGCACACGCTGCCCTTCCCCATTTGCTGCACGCCAGTGG<br>ACGCACCTTAGAGGCAGTGCAGGACCTGCTGGAACAGGGCCGCCAGCACAGCCAGGACCTGGCCTTTGTGAGC<br>ATGCTCAATGACATTGCGGCAACCCCTACAGCAGCCATGCCCTTCAGGGGTTACACTGTGCTAGGTGTTGAGG<br>GCCGTGTCCAAGAAGTGCAGCAAGTGTCCACCAACAAGCGCCCACTCTGGTTCATCTGCTCAGGGATGGGCAC<br>GCAGTGGCGCGGGATGGGGCTGAGCCTCATGCGCCTGGACAGCTTCCGTGAGTCTATCCTGCGCTCCGATGAG<br>GCTGTGAAGCCGTTGGGAGTGAAAGTGTCAGATCTGCTGTTGAGCACAGATGAGCGCACCTTTGATGACATCG<br>TGCATGCCTTTGTGAGCCTCACTGCCATCCAGATTGCCCTCATCGACCTACTGACTTCTGTGGGACTGAAACC<br>TGACGGCATCATTGGGCACTCCTTGGGAGAGGTTGCCTGTGGCTATGCAGATGGCTGTCTCTCCCAGAGAGAG<br>GCTGTGCTTGCAGCTTACTGGCGAGGCCAGTGCATCAAAGATGCCCACCTCCCGCCTGGATCCATGGCAGCTG<br>TTGGTTTGTCCTGGGAGGAATGTAAACAGCGCTGCCCCGCTGGCGTGGTGCCTGCCTGCCACAACTCTGAGGA<br>CACCGTGACCATCTCTGGACCTCAGGCTGCAGTGAATGAATTTGTGGAGCAGCTAAAGCAAGAAGGTGTGTTT<br>GCCAAGGAGGTACGAACAGGAGGCCTGGCTTTCCACTCCTACTTCATGGAAGGAATTGCCCCCACATTGCTGC<br>AGGCTCTCAAGAAGGTGATCCGGGAACCACGGCCGCGCTCGGCTCGATGGCTCAGCACCTCTATCCCTGAGGC<br>CCAGTGGCAGAGCAGCCTGGCCCGCACATCTTCTGCCGAGTACAATGTCAACAACCTGGTGAGCCCTGTGCTC<br>TTCCAGGAAGCACTGTGGCACATCCCTGAGCATGCCGTGGTGCTGGAGATTGCGCCCCACGCACTGTTGCAGG<br>CTGTCCTGAAGCGAGGCGTGAAGTCCAGCTGCACCATCATTCCCTTGATGAAGAGGGATCATAAAGATAACTT<br>GGAGTTCTTTCTCACCAACCTTGGCAAGGTGCACCTCACAGGCATCAATGTCAACCCTAACGCCTTGTTCCCAC<br>CTGTGGAGTTCCCGGCTCCCCGAGGGACTCCTCTCATCTCCCCTCACATCAAGTGGGACCACAGTCAGACTTGG<br>GATGTCCCGGTTGCTGAGGACTTCCCAAACGGCTCCAGCTCCTCCTCTGCTACAGTCTACAGCATCGACGCCAG

Construct	Sequence form start to stop codon
	TCCTGAGTCGCCCGACCACTACCTGGTAGACCACTGCATTGACGGCCGGGTCATCTTCCCTGGCACTGGCTACC<br>TGTGCCTGGTGTGGAAGACACTGGCTCGCAGCCTGGGCTTGTCCCTAGAAGAGACCCCTGTGGTATTTGAGAA<br>TGTGTCGTTTCATCAGGCCACTATACTACCCAAGACAGGAACCGTGGCGCTGGAGGTGAGGCTGCTAGAGGCC<br>TCCCATGCCTTTGAGGTGTCTGACACTGGCAATCTGATTGTGAGCGGAAAAGTGTACCTGTGGGAAGACCCGA<br>ACTCCAAGTTATTCGACCACCCAGAAGTCCCAACACCCCCTGAGTCTGCATCGGTCTCCCGCCTGACCCAGGGA<br>GAAGTATACAAGGAGCTGCGGCTGCGTGGCTATGATTATGGCCCTCAGTTCCAGGGCATCTGTGAGGCCACCC<br>TTGAAGGTGAACAAGGCAAGCTGCTCTGGAAAGATAACTGGGTGACCTTCATGGACACAATGCTGCAGGTAT<br>CCATTCTGGGTTCTAGCCAGCAGAGTCTACAGCTACCTACCCGTGTGACCGCCATCTATATCGACCCTGCCACC<br>CACCGTCAGAAGGTGTACAGGCTGAAGGAGGACACTCAAGTGGCTGATGTGACAACGAGCCGCTGTCTGGGCA<br>TAACGGTCTCTGGTGGTATCCACATCTCAAGACTACAGACGACAGCAACCTCACGGCGGCAGCAAGAACAGCT<br>GGTCCCCACCTTGGAAAAGTTCGTTTTCACACCGCACATGGAGGCTGAGTGCCTGTCTGAGAGCACTGCCCTG<br>CAGAAGGAGCTGCAACTGTGCAAGGGTCTGGCACGGGCTCTGCAGACCAAGGCCACCCAGCAAGGGCTGAAGG<br>CGGCAATGCTTGGGCAAGAGGACCCTCCACAGCACGGGCTGCCTCGACTCCTGGCAGCTGCTTGCCAGTTGCA<br>GCTCAACGGGAACCTGCAGCTGGAGCTGGGAGAAGCGCTGGCTCAAGAGAGGCTCCTGCTGCCAGAAGACCCT<br>CTGATCAGTGGCCTCCTCAACTCCCAGGCCCTCAAGGCCTGCGTAGACACAGCCCTGGAGAACTTGTCTACTCT<br>CAAGATGAAGGTGGCAGAGGTGCTGGCTGGAGAAGGCCACTTGTATTCCCGAATCCCGGCACTGCTCAACACC<br>CAGCCCATGCTACAACTGGAATACACAGCCACCGACCGGCACCCCCAGGCCCTGAAGGATGTTCAGACCAAAC<br>TGCAGCAGCATGATGTGGCGCAGGGCCAGTGGAACCCTTCCGACCCTGCGCCCAGCAGCCTGGGTGCCCTTGA<br>CCTTCTGGTGTGCAACTGTGCATTAGCCACCCTGGGGGATCCAGCCTTGGCCCTGGACAACATGGTAGCTGCC<br>CTCAAGGAAGGTGGTTTCCTGCTAGTGCACACAGTGCTCAAAGGACATGCCCTTGGGGAGACCCTGGCCTGCC<br>TACCCTCTGAGGTGCAGCCTGCGCCCAGCCTCCTAAGCCAGGAGGAGTGGGAGAGCCTGTTCTCGAGGAAGGC<br>ACTACACCTGGTGGGCCTTAAAAGGTCCTTCTACGGTACTGCGCTGTTCCTGTGCCGGCGAGCCATCCCACAG<br>GAGAAACCTATCTTCCTGTCTGTGGAGGATACCAGCTTCCAGTGGGTGGACTCTCTGAAGAGCACTCTGGCCA<br>CGTCCTCCTCCCAGCCTGTGTGGCTAACGGCCATGGACTGCCCCACCTCGGGTGTGGTGGGTTTGGTGAATTG<br>TCTCCGAAAAGAGCCGGGTGGACACCGGATTCGGTGTATCCTGCTGTCCAACCTCAGCAACACATCTCACGCC<br>CCCAAGTTGGACCCTGGCTCTCCAGAGCTACAGCAGGTGCTAAAGCATGACCTCGTGATGAACGTGTACCGGG<br>ACGGGGCCTGGGGTGCCTTCCGTCACTTCCAGTTAGAGCAGGACAAGCCCAAGGAGCAGACAGCGCATGCCTT<br>TGTAAACGTCCTCACCCGAGGGGACCTCGCCTCCATCCGCTGGGTCTCCTCCCCCCTGAAGCACACGCAGCCCT<br>CGAGCTCAGGAGCACAGCTCTGCACTGTCTACTACGCCTCACTGAACTTCCGAGACATCATGCTGGCCACGGG<br>CAAGCTGTCCCCTGATGCCATTCCAGGTAAATGGGCCAGCCGAGACTGCATGCTCGGCATGGAGTTCTCAGGC<br>CGGGATAGGTGTGGCCGGCGTGTGATGGGGCTGGTTCCTGCAGAAGGCCTGGCCACCTCAGTCCTGCTATCAT<br>CTGACTTCCTCTGGGATGTACCCTCCAGCTGGACCCTGGAGGAGGCGGCCTCTGTGCCCGTCGTCTATACCACT<br>GCTTACTACTCGTTAGTGGTTCGCGGGCGCATCCAGCGTGGGGAGACCGTGCTCATCCACTCAGGTTCAGGTG<br>GTGTGGGCCAAGCGGCCATTTCCATTGCCCTCAGTCTGGGCTGCCGCGTCTTCACCACTGTGGGCTCTGCAGAG<br>AAGCGAGCATACCTCCAGGCCAGGTTCCCTCAGCTTGATGACACCAGCTTTGCCAACTCGAGGGACACATCAT<br>TTGAGCAGCACGTGTTACTGCACACAGGTGGCAAAGGGGTCGACCTGGTCCTCAACTCACTGGCAGAAGAGAA<br>GCTGCAGGCCAGTGTGCGGTGCTTGGCTCAGCATGGTCGCTTCTTAGAGATTGGCAAATTTGATCTTTCTAAC<br>AACCACCCTCTGGGCATGGCTATCTTCTTGAAGAACGTCACTTTCCATGGGATCCTGCTGGACGCCCTTTTTG<br>AGGAGGCCAATGACAGCTGGCGGGAGGTGGCGGCACTCCTGAAGGCTGGCATTCGTGATGGAGTCGTGAAGCC<br>CCTCAAGTGCACAGTGTTTCCCAAGGCCCAGGTGGAAGATGCCTTCCGCTACATGGCTCAGGGGAAACACATT<br>GGCAAAGTCCTTGTCCAGGTACGGGAGGAGGAGCCTGAGGCTGTGCTGCCAGGGGCTCAGCCCACCCTGATTT<br>CTGCCATCTCCAAGACCTTCTGCCCAGCCCATAAGAGTTACATCATCACTGGTGGCCTAGGTGGCTTTGGCCT<br>GGAGCTGGCCCGGTGGCTCGTGCTTCGCGGAGCCCAGAGGCTTGTGCTGACTTCCCGATCTGGAATCCGCACC<br>GGCTACCAAGCCAAGCACATTCGGGAGTGGAGACGCCAGGGCATCCAAGTGCTCGTGTCAACAAGCAACGTGA<br>GCTCACTGGAGGGGGCCCGTGCTCTCATCGCCGAAGCCACAAAGCTGGGGCCCGTTGGGGGTGTCTTCAACCT<br>GGCCATGGTTTTGAGGGATGCCATGCTGGAGAACCAGACCCCAGAGCTCTTCCAGGATGTCAACAAGCCCAAA<br>TACAATGGCACCCTGAACCTTGACAGGGCAACCCGGGAAGCCTGCCCTGAGCTGGACTACTTTGTGGCCTTCT<br>CCTCTGTAAGCTGCGGGCGTGGTAATGCTGGCCAAACTAACTACGGCTTCGCCAACTCTACCATGGAGCGTAT<br>ATGTGAACAGCGCAGGCACGATGGCCTCCCAGGCCTTGCCGTGCAGTGGGGTGCCATTGGTGACGTGGGCATT<br>GTCCTGGAAGCGATGGGCACCAATGACACAGTCATCGGAGGTACGCTGCCTCAGCGCATCTCCTCCTGCATGG<br>AGGTACTGGACCTCTTCCTGAATCAGCCCCACGCAGTCCTGAGCAGCTTTGTGCTGGCAGAGAAGAAAGCTGT<br>GGCCCATGGGGACGGGGACACCCAGAGGGATCTGGTGAAAGCTGTAGCACACATCCTAGGCATCCGAGACCTC<br>GCAGGTATTAACCTGGACAGCACGCTGGCAGACCTCGGCCTGGACTCGCTCATGGGTGTGGAAGTTCGTCAGA<br>TCCTGGAACGAGAACACGATCTGGTGCTGCCCATGCGTGAGGTGCGGCAGCTCACGCTGCGGAAACTTCAGGA<br>AATGTCCTCCAAGGCGGCCGCAGCGCAGCGCACCGCGGGTGACCGCAGGCCGAGTTTCACCACCGTGCACGGC<br>GCGGACGCCACCGAGATCCGGGCGAGTGAGCTGACCCTGGACAAGTTCATCGACGCCGAAACGCTCCGGGCCG<br>CACCGGGTCTGCCCAAGGTCACCACCGAGCCACGGACGGTGTTGCTCTCGGGCGCCAACGGCTGGCTGGGCCG<br>GTTCCTCACGTTGCAGTGGCTGGAACGCCTGGCACCTGTCGGCGGCACCCTCATCACGATCGTGCGGGGCCGCG<br>ACGACGCCGCGGCCCGCGCACGGCTGACCCAGGCCTACGACACCGATCCCGAGTTGTCCCGCCGCTTCGCCGAG<br>CTGGCCGACCGCCACCTGCGGGTGGTCGCCGGTGACATCGGCGACCCGAATCTGGGCCTCACACCCGAGATCTG<br>GCACCGGCTCGCCGCCGAGGTCGACCTGGTGGTGCATCCGGCAGCGCTGGTCAACCACGTGCTCCCCTACCGGC<br>AGCTGTTCGGCCCCAACGTCGTGGGCACGGCCGAGGTGATCAAGCTGGCCCTCACCGAACGGATCAAGCCCGT<br>CACGTACCTGTCCACCGTGTCGGTGGCCATGGGGATCCCCGACTTCGAGGAGGACGGCGACATCCGGACCGTG

Construct	Sequence form start to stop codon
	AGCCCGGTGCGCCCGCTCGACGGCGGATACGCCAACGGCTACGGCAACAGCAAGTGGGCCGGCGAGGTGCTGC<br>TGCGGGAGGCCCACGATCTGTGCGGGCTGCCCGTGGCGACGTTCCGCTCGGACATGATCCTGGCGCATCCGCGC<br>TACCGCGGTCAGGTCAACGTGCCAGACATGTTCACGCGACTCCTGTTGAGCCTCTTGATCACCGGCGTCGCGCC<br>GCGGTCGTTCTACATCGGAGACGGTGAGCGCCCGCGGGCGCACTACCCCGGCCTGACGGTCGATTTCGTGGCC<br>GAGGCGGTCACGACGCTCGGCGCGCAGCAGCGCGAGGGATACGTGTCCTACGACGTGATGAACCCGCACGACG<br>ACGGGATCTCCCTGGATGTGTTCGTGGACTGGCTGATCCGGGCGGGCCATCCGATCGACCGGGTCGACGACTA<br>CGACGACTGGGTGCGTCGGTTCGAGACCGCGTTGACCGCGCTTCCCGAGAAGCGCCGCGCACAGACCGTACTG<br>CCGCTGCTGCACGCGTTCCGCGCTCCGCAGGCACCGTTGCGCGGCGCACCCGAACCCACGGAGGTGTTCCACGC<br>CGCGGTGCGCACCGCGAAGGTGGGCCCGGGAGACATCCCGCACCTCGACGAGGCGCTGATCGACAAGTACATA<br>CGCGATCTGCGTGAGTTCGGTCTGATCGGAGGTAAGCTTCATCATCACCACCACCACCACCAC

Supplementary References

1. Gusenda, C., Calixto, A. R., Da Silva, J. R., Fernandes, P. A. & Grininger, M. The Kinetics of Carbon-Carbon Bond Formation in Metazoan Fatty Acid Synthase and Its Impact on Product Fidelity. *Angew. Chem. Int. Ed.* e202412195 (2024) doi:10.1002/anie.202412195.
2. Okonechnikov, K., Golosova, O., Fursov, M., & the UGENE team. Unipro UGENE: a unified bioinformatics toolkit. *Bioinformatics* **28**, 1166–1167 (2012).
3. Deng, X. *et al.* Structure-guided reshaping of the acyl binding pocket of 'TesA thioesterase enhances octanoic acid production in E. coli. *Metab. Eng.* **61**, 24–32 (2020).
4. Mullowney, M. W., McClure, R. A., Robey, M. T., Kelleher, N. L. & Thomson, R. J. Natural products from thioester reductase containing biosynthetic pathways. *Nat. Prod. Rep.* **35**, 847–878 (2018).
5. Finnigan, W. *et al.* Characterization of Carboxylic Acid Reductases as Enzymes in the Toolbox for Synthetic Chemistry. *ChemCatChem* **9**, 1005–1017 (2017).
